# Multiple Introductions Followed by Ongoing Community Spread of SARS-CoV-2 at One of the Largest Metropolitan Areas of Northeast Brazil

**DOI:** 10.3390/v12121414

**Published:** 2020-12-09

**Authors:** Marcelo Henrique Santos Paiva, Duschinka Ribeiro Duarte Guedes, Cássia Docena, Matheus Filgueira Bezerra, Filipe Zimmer Dezordi, Laís Ceschini Machado, Larissa Krokovsky, Elisama Helvecio, Alexandre Freitas da Silva, Luydson Richardson Silva Vasconcelos, Antonio Mauro Rezende, Severino Jefferson Ribeiro da Silva, Kamila Gaudêncio da Silva Sales, Bruna Santos Lima Figueiredo de Sá, Derciliano Lopes da Cruz, Claudio Eduardo Cavalcanti, Armando de Menezes Neto, Caroline Targino Alves da Silva, Renata Pessôa Germano Mendes, Maria Almerice Lopes da Silva, Tiago Gräf, Paola Cristina Resende, Gonzalo Bello, Michelle da Silva Barros, Wheverton Ricardo Correia do Nascimento, Rodrigo Moraes Loyo Arcoverde, Luciane Caroline Albuquerque Bezerra, Sinval Pinto Brandão-Filho, Constância Flávia Junqueira Ayres, Gabriel Luz Wallau

**Affiliations:** 1 Núcleo de Ciências da Vida, Universidade Federal de Pernambuco (UFPE), Centro Acadêmico do Agreste-Rodovia BR-104, km 59-Nova Caruaru, Caruaru 55002-970, Brazil; marcelo.paiva@ufpe.br; 2 Departamento de Entomologia, Instituto Aggeu Magalhães (IAM)-Fundação Oswaldo Cruz-FIOCRUZ, Recife 50670-420, Brazil; dguedes@cpqam.fiocruz.br (D.R.D.G.); zimmer.filipe@gmail.com (F.Z.D.); laisceschini@gmail.com (L.C.M.); lkrokovsky@gmail.com (L.K.); elisama_helvecio@hotmail.com (E.H.); alexfreitasbiotec@gmail.com (A.F.d.S.); dercylopes10@gmail.com (D.L.d.C.); tans@cpqam.fiocruz.br (C.F.J.A.); 3 Núcleo de Plataformas Tecnológicas (NPT), Instituto Aggeu Magalhães (IAM)-Fundação Oswaldo Cruz-FIOCRUZ, Recife 50670-420, Brazil; cassiadc@cpqam.fiocruz.br; 4 Departamento de Microbiologia, Instituto Aggeu Magalhães (IAM)-Fundação Oswaldo Cruz-FIOCRUZ, Recife 50670-420, Brazil; matheus.bezerra@cpqam.fiocruz.br (M.F.B.); antonio.rezende@cpqam.fiocruz.br (A.M.R.); bruna@cpqam.fiocruz.br (B.S.L.F.d.S.); 5 Núcleo de Bioinformática (NBI), Instituto Aggeu Magalhães (IAM)-Fundação Oswaldo Cruz-FIOCRUZ, Recife 50670-420, Brazil; 6 Departamento de Parasitologia, Instituto Aggeu Magalhães (IAM)-Fundação Oswaldo Cruz-FIOCRUZ, Recife 50670-420, Brazil; luydson.vasconcelos@cpqam.fiocruz.br (L.R.S.V.); armandomenezes@gmail.com (A.d.M.N.); carolinetargino.as@gmail.com (C.T.A.d.S.); renata_pessoa100@hotmail.com (R.P.G.M.); almerice@cpqam.fiocruz.br (M.A.L.d.S.); wheverthon.nascimento@cpqam.fiocruz.br (W.R.C.d.N.); rodrigo.loyo@cpqam.fiocruz.br (R.M.L.A.); sinval@cpqam.fiocruz.br (S.P.B.-F.); 7 Laboratório de Virologia e Terapia Experimental (LAVITE), Instituto Aggeu Magalhães (IAM)-Fundação Oswaldo Cruz-FIOCRUZ, Recife 50670-420, Brazil; jeffersonbiotecviro@gmail.com (S.J.R.d.S.); claudio.cavalcanti@cpqam.fiocruz.br (C.E.C.); 8 Departamento de Imunologia, Instituto Aggeu Magalhães (IAM)-Fundação Oswaldo Cruz-FIOCRUZ, Recife 50670-420, Brazil; kamilasalesg@gmail.com (K.G.d.S.S.); msb.michelle@gmail.com (M.d.S.B.); 9 Instituto Gonçalo Moniz, Fundação Oswaldo Cruz, Salvador 40296-710, Brazil; tiago.graf@fiocruz.br; 10 Laboratory of Respiratory Viruses and Measles, Oswaldo Cruz Institute (IOC), Oswaldo Cruz Foundation (FIOCRUZ), Rio de Janeiro 21040-900, Brazil; paola@ioc.fiocruz.br; 11 Laboratório de AIDS e Imunologia Molecular, Instituto Oswaldo Cruz, FIOCRUZ, Rio de Janeiro 21040-900, Brazil; gbellobr@gmail.com; 12 Departamento de Medicina Tropical, Centro de Ciências Médicas, Universidade Federal de Pernambuco (UFPE), Recife 50670-901, Brazil; 13 Secretaria de Saúde de Pernambuco (SES-PE), Secretaria Executiva de Vigilância em Saúde (SEVS-PE), Recife 50751-530, Brazil; lua_cad@yahoo.com.br

**Keywords:** SARS-CoV2, coronavirus, outbreak, Brazil, phylogenetics

## Abstract

Multiple epicenters of the SARS-CoV-2 pandemic have emerged since the first pneumonia cases in Wuhan, China, such as Italy, USA, and Brazil. Brazil is the third-most affected country worldwide, but genomic sequences of SARS-CoV-2 strains are mostly restricted to states from the Southeast region. Pernambuco state, located in the Northeast region, is the sixth most affected Brazilian state, but very few genomic sequences from the strains circulating in this region are available. We sequenced 101 strains of SARS-CoV-2 from patients presenting Covid-19 symptoms that reside in Pernambuco. Phylogenetic reconstructions revealed that all genomes belong to the B lineage and most of the samples (88%) were classified as lineage B.1.1. We detected multiple viral introductions from abroad (likely from Europe) as well as six local B.1.1 clades composed by Pernambuco only strains. Local clades comprise sequences from the capital city (Recife) and other country-side cities, corroborating the community spread between different municipalities of the state. These findings demonstrate that different from Southeastern Brazilian states where the epidemics were majorly driven by one dominant lineage (B.1.1.28 or B.1.1.33), the early epidemic phase at the Pernambuco state was driven by multiple B.1.1 lineages seeded through both national and international traveling.

## 1. Introduction

Acute respiratory infection is caused by several viral pathogens such as influenza, rhinoviruses, and coronaviruses (CoV), that can infect the upper and lower respiratory tract of humans leading to compromised oxygenation and eventually multiple organ failure and death [1]. Two coronaviruses, SARS-CoV in 2002 and MERS-CoV, in 2012, spilled over to the human population affecting thousands of people, but these viruses were not well adapted to human-to-human transmission and the outbreaks came into control before massive human spread [2]. However, in December 2019, a new pneumonia-like illness was reported in the Wuhan municipality in China. A few weeks later the genome of the etiological agent was sequenced and revealed a new coronavirus named SARS-CoV-2 [3]. Different from the SARS-CoV-1 and MERS-CoV, SARS-CoV-2 is particularly well adapted to sustained human-to-human transmission. It is highly infectious, spreading through symptomatic and asymptomatic individuals [4]. Due to widespread human mobility, this virus reached all continents in less than three months after the initial reports from Wuhan (https://www.who.int/emergencies/diseases/novel-coronavirus-2019/situation-reports/). This pandemic is still unfolding, and more than 30 million people have already been infected by the virus, and more than 1 million have died from it, highlighting that humankind is facing one of the largest and most challenging human pandemics of all times.

Since the initial spread of SARS-CoV-2, there were two well recognized epicenters of the pandemic outside China: Northern Italy, which reported the peak of the epidemic around late March to mid-April 2020 [5,6], and the United States of America (USA), which reached the first peak around April 2020, with most cases concentrating in the New York City area [7]. Currently, the USA is the most affected country in the world, accounting for more than 7 million infected people. Large-scale SARS-CoV-2 diagnostic performed in several countries are revealing a complex picture of infection waves due to relaxing of non-pharmaceutical interventions associated with the low level of acquired immunity [8] (https://coronavirus.jhu.edu/map.html, https://www.who.int/emergencies/diseases/novel-coronavirus-2019/situation-reports/). SARS-CoV-2 human infections have been increasing at a rapid pace in South America as well, led mostly by Brazil—the most populous country of the continent with more than 211.8 million people (Instituto Brasileiro de Geografia e Estatística, IBGE—http://www.ibge.gov.br). The first SARS-CoV-2 infection in Brazil was reported on 25 February 2020, and now (September 2020), Brazil is the third-most affected country in the world, with more than 5 million cases and 150 thousand deaths [9] (https://coronavirus.jhu.edu/map.html—https://covid.saude.gov.br/). Recent publications based on the sequencing and characterization of SARS-CoV-2 genomes from Minas Gerais and São Paulo (southeast region of Brazil) revealed the introduction of different SARS-CoV-2 lineages from European countries, which were associated with recent travel history of the patients [9,10,11]. Resende et al. 2020 [12], sequenced 95 SARS-CoV-2 genomes from 10 Brazilian states and Candido et al. 2020 sequenced 427 genomes from 21 Brazilian states and both identified more than 100 independent introductions of SARS-CoV-2 in Brazil and the extensive community spread of prevailing lineages (B.1.1.28 and B.1.1.33) in some Southeast Brazilian states [12,13].

Pernambuco is the seventh most populous state in Brazil and the eighth state regarding the number of infected patients with 147.872 confirmed cases, but the fourth in terms of deaths (8.279 deaths) and the second considering lethality (5.6%) (last accessed September 2020—CIEVS PE—https://www.cievspe.com/). So far, no genomic epidemiology study [14] was performed to sequence SARS-CoV-2 viral genomes circulating in the state. In this study, we sequenced 101 SARS-CoV-2 genomes from the Pernambuco state, Northeastern Brazil during an early pandemic phase in order to evaluate the emergence and community spread of this virus in one of the most affected Brazilian states.

## 2. Materials and Methods

### 2.1. Patient Samples and SARS-CoV-2 Molecular Detection

**Figure 1:**
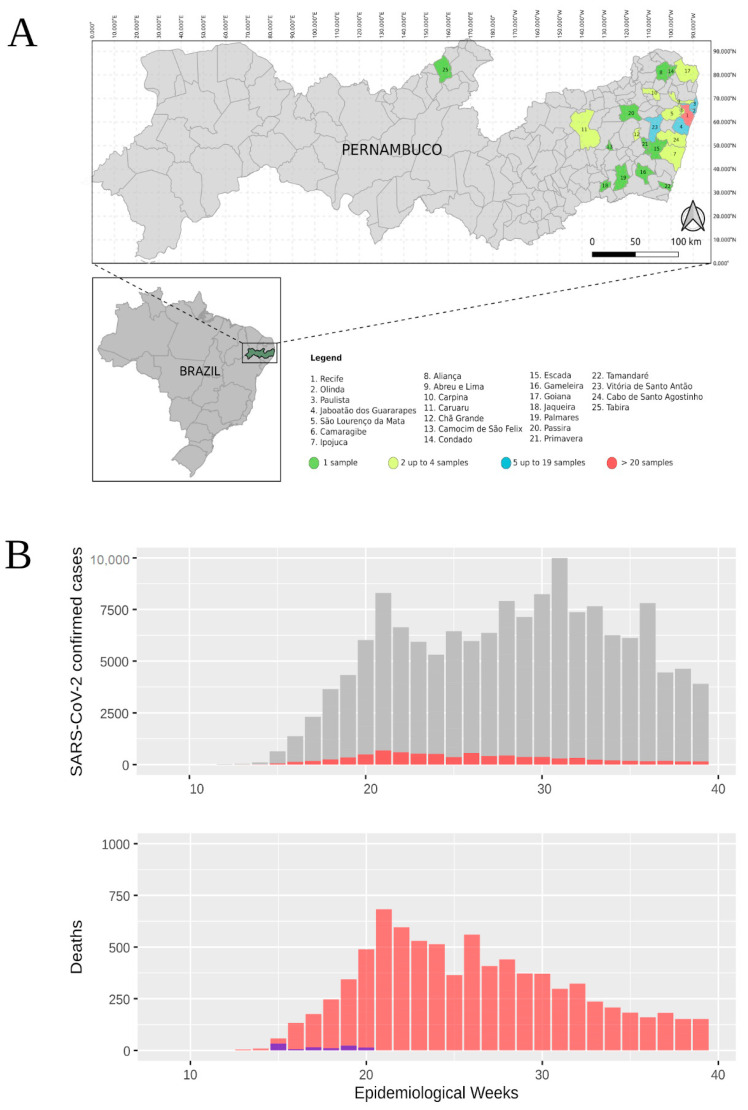
Sample distribution of the sequenced SARS-CoV-2 genomes among the Pernambuco state municipalities and epidemiological curves of SARS-CoV-2 confirmed patients from Pernambuco per epidemiological week. (**A**) Pernambuco state map showing the number of genomes sequenced in this study distributed by Pernambuco municipalities. Municipality colors represents the number of the genomes obtained from a given municipality (green—1 genome, yellow—2 up to 4 genomes, blue—5 to nineteen genomes and red—more than 20 genomes) and the number over a geographical area indicates the municipality name as shown in the numerical legend below the map. (**B**) SARS-CoV-2 epidemiological data from Pernambuco state—number of confirmed SARS-CoV-2 cases (grey) and deaths (red) per epidemiological week. Number of genomes sequence (blue bars) obtained in this study. Epidemiological week 10 refers to the first week of March 2020 and epidemiological week 11 includes the first official case reported in Pernambuco on 12 March 2020.

Samples were obtained from nasopharyngeal and oropharyngeal swabs from symptomatic patients of different ambulatory facilities from Pernambuco (Northeast Brazil) (Figure 1A). Samples metadata can be found in Table S1. These samples are a part of the COVID-19 biorepository from the Aggeu Magalhães Institute (IAM), an Oswaldo Cruz Foundation (FIOCRUZ) unit. All standard operating procedures (SOPs) established by the World Health Organization guidelines were employed [15]. Rigorous biosafety measures were employed as all samples were manipulated in the BSL-3 facility laboratory. RNA extractions were performed using the robotic platform using the Maxwell^®^ 16 Viral Total Nucleic Acid Purification Kit (Promega, Wisconsin-USA), following the manufacturer’s protocol. The molecular detection was performed using the Kit Molecular Bio Manguinhos SARS-CoV-2 (E/RP): A single-step reaction for detecting the virus envelope gene (E) and the Ribonuclease P housekeeping control gene (RNAse P) [16]. The study was approved by the Aggeu Magalhaes Institute Ethical Committee—CAAE 32333120.4.0000.5190.

### 2.2. Genomic Sequencing

Total RNA was used for single strand cDNA generation using FIREScript—Solis Biodyne kit (*Sinapse inc*) following manufacturer’s instruction. cDNA generated was subjected to multiplex PCR reactions using Q5 High Fidelity Hot-Start DNA Polymerase (New England Biolabs) and a set of specific primers, designed by (https://www.protocols.io/view/ncov-2019-sequencing-protocol-bbmuik6w). Cycling conditions were: 98 °C at 30 s, 98 °C at 15 s, 62 °C at 30 s, and 65 °C at 5 min during 35 cycles. Amplified PCR products were purified using AMPure XP Beads (Beckman Coulter) and quantified using the Qubit® dsDNA HS Assay Kits (Invitrogen) following the manufacturer’s instruction. Sequence libraries were prepared with Nextera XT Library Prep Kit (Illumina, San Diego, CA, USA) using 1.5 ng of PCR products following the manufacturer’s instructions. Sequencing was performed in the MiSeq (Illumina) machine using MiSeq Reagent kit V3 of 150 cycles employing a paired-end strategy.

### 2.3. Viral Strain Isolation and Resequencing

A virus (hCoV-19/Brazil/PE-IAM19/2020) containing a rare mutation N501Y in the spike protein was sent to Laboratory of Respiratory Viruses and Measles, the WHO Regional Reference Laboratory in Americas for SARS-CoV-2, for viral isolation and whole genome resequencing. The viral isolation was conducted using 200 µL of the clinical sample inoculated in VERO CCL-81 cells and after the third passage the cytopathic effect (CE) was observed. The supernatant was recovered, and the viral RNA was extracted using the QIAamp Viral RNA Mini Kit (Qiagen). These experiments were performed at the BSL3 facility. The viral isolation was confirmed by the real time RT-PCR test targeting the gene E Kit Molecular Bio Manguinhos SARS-CoV-2 (E/RP). The RNA extracted from clinical sample and from isolated viruses from the third passage were submitted to whole genome sequencing using the RT and PCR multiplex protocol [12]. Amplified PCR products were purified using AMPure XP Beads (Beckman Coulter) following and quantified using the Qubit® dsDNA HS Assay Kits (Invitrogen) following the manufacturer’s instruction. The library was prepared using the Nextera XT Library Prep Kit (Illumina, San Diego, CA, USA) and sequencing was performed in the Illumina MiSeq machine using MiSeq Reagent Kit v2 Micro employing a paired-end strategy.

### 2.4. Genome Assembly and Annotation

Low quality raw sequencing reads, and primers sequences were removed using Trimmomatic 0.36 with default parameters except that the primers sequences used for amplification were passed in the ILLUMINACLIP parameter. Based on the knowledge that epidemic viruses sampled at short time frames does not accumulate a substantial number of mutations, we performed a reference-based assembly strategy using the first published SARS-CoV-2 genome as reference (NC_045512.2) using Bowtie2 software [17] with default parameters. Following, we generated a .bed file using bedtools v 2.15.0 [18], samtools 1.5 [19], vcf-annotate (parameters-filter Qual = 20/MinDP = 100/SnpGap = 20) from vcftools v 0.1.13 [20] and genomeCoverageBed from bedtools v 2.15.0 [18] keeping only position with >5x of coverage. Lastly, we used bedtools maskfasta [18] to generate the final consensus sequences.

The N regions and coverage values for each genome were plotted using karyoploteR [21]. All in house scripts used in the following sections are deposited on https://github.com/dezordi/SARS-CoV-2_tools [22].

### 2.5. Evolutionary Analysis

46,638 genomes of SARS-CoV-2 from GISAID (https://www.gisaid.org/) available up to 24 Jul 2020 were retrieved, this number represents the 45,498 SARS-CoV-2 genomes identified in human samples, with complete and sequenced with high coverage from all continent except South America plus 1140 genomes with no quality filter from South America countries. Sequences from other continents except South America with less than 29,400 pb were removed with fasta_cleaner.py resulting in 39,467 genomes. The genomes were aligned with the reference genome NC_045512.2 using MAFFT add v7.310 [23] with the -keep-length parameter. The 3’ and 5’ ends were removed from alignment as indicated by [24]. Sequences from the edited alignment were submitted to a step to remove redundancy with cluster_gisaid.py, where sequences with 100% of identity from the same country were clusterized with cd-hit-est [25]. After the first step of clusterization, some countries were still overrepresented, for example, USA and England with more than 3000 genomes remaining after clusterization. For these (see Table S2), a second step of clusterization was performed with a minimum threshold of percentage identity (99.6%) to obtain a number of genomes per country close to the mean number of genomes from other countries. Finally, the 101 genomes sequenced in our study are aligned with the final dataset of 7264 genomes and the final alignment was visualized and checked with Aliview [26].

SARS-CoV-2 lineages were assigned with pangolin (https://pangolin.cog-uk.io/) updated at 14 August 2020. Phylogenetic trees were constructed with IQ-TREE 2.1.1 [27] using the ultrafast bootstrap method [28] with 10,000 replicates and with the ModelFinder [29] for two datasets, the first one with 7264 genomes encompassing all genomes that passed from aforementioned filters, and the second one with this previously dataset sampled by country and week (or by state and week for Brazilian genomes) with gisaid_sampler.py. Genomes without a complete collection date information were removed, which resulted in 983 genomes. Branch support was recalculated with the Transfer Bootstrap Expectation (TBE) [30] using 1000 randomly selected bootstrap replicates. The iTOL [31] was used to annotate the phylogenetic trees, tree branches were colored by region and tip colors were colored by pangolin lineage signatures, the root was set between lineages A and B, as proposed by [24]. After tree reconstruction, genomes from GISAID that clustered with IAM genomes with ultrafast bootstrap branch support ≥40 were evaluated regarding the synapomorphic SNPs supporting each cluster.

For the Bayesian analysis, we firstly used the dataset generated with gisaid_sampler.py comprising sequences from A Lineage, B Lineage and B1 Lineage including all SARS-CoV-2 genomes sequenced in this study in a maximum likelihood analysis. After the ML reconstruction with IQ-TREE the tree was evaluated in Tempest 1.5.3 [32] to check the root-to-tip temporal signal. The outlier sequences were removed before the phylogenetic analysis performed in BEAST 1.10.4 [33]. Bayesian time-scaled trees were inferred for each dataset using a strict clock applying a fixed mean clock rate of 0.8 × 10^−3^ as used by other authors [7,34]. We also applied the Bayesian coalescent Skyline model and the GTR + G4 + I nucleotide substitution model. These analyses were based on three independent runs of 100 million MCMC generation sampling in every 10,000 steps. To gain more insights about local SARS-CoV-2 transmission, the identified local clusters were analyzed together in a separate run in Beast. Clock prior was allowed to vary uniformly between 0.8 × 10^−3^–1.0 × 10^−3^ and other models were as above. The run convergence was evaluated with Tracer 1.7 and all parameters showed ESS values >200. The generated trees were combined using LogCombiner and the Maximum clade credibility tree was obtained using the treeannotator. The time-scaled trees were visualized and edited on Figtree 1.4.4 (http://tree.bio.ed.ac.uk/software/figtree/) and Inkscape 0.92 (https://inkscape.org/).

### 2.6. Single Nucleotide Polymorphism Analysis

Single nucleotide polymorphism (SNPs) were evaluated using the snp-sites tool [35], using as input the alignments between each IAM genome and the NC_045512.2 as the reference, the positions with SNPs were retrieved with BCFtools [36]. The SNPs positions were crossed with the genome assembly depth and nucleotide diversity per position accessed with bam-readcount tool [37] (https://github.com/genome/bam-readcount), the outputs from bcftools and bam-readcount were crossed with snp_div.py to access the metrics and nucleotide diversity of SNPs by genomic region (Tables S3 and S4). Owing to the importance of Spike protein in SARS-CoV-2 biology, single amino acid polymorphisms (SAPs) and regions were deletions were found in other SARS-CoV-2 genomes [38] were carefully analyzed using Aliview and karyoploteR.

### 2.7. Epidemiological Data

SARS-CoV-2 confirmed infections and deaths cases from Pernambuco state were obtained from from the Brazilian Ministry of Health coronavirus website (https://covid.saude.gov.br/). We collected the number of cases and deaths per day and epidemiological week since the first case reported in the state (12 March 2020). Plots were performed using the ggplot2 package of the R statistical language (https://www.r-project.org/).

## 3. Results and Discussion

### 3.1. Epidemiological Data from Pernambuco State

The first confirmed SARS-CoV-2 infection in Pernambuco was reported in the second week of March (12 March 2020) in the 11th epidemiological week (Figure 1B) comprising an elderly couple returning from Italy on 28 February [39], only 16 after the first confirmed case in Brazil (25 February 2020) (Figure 1B—top panel). In the 21st epidemiological week, the Pernambuco state reached the first peak of cases followed by a second peak at the 31st epidemiological week (Figure 1B—top panel). An increasing number of deaths followed in which the largest number occurred at the 21st epidemiological with 683 deaths and an average of 97.57 deaths per day (Figure 1B—lower panel). In this study, 101 samples from the beginning of the SARS-CoV-2 spread at the Pernambuco state were processed for whole genome amplification and sequencing. Samples were obtained from twenty-five municipalities of the Pernambuco state (Figure 1A) and were distributed across the 15th (33 samples), 16th (six samples), 17th (14 samples), 18th (11 samples), 19th (23 samples), and 20th (14 samples) epidemiological weeks (blue bars in Figure 1B—lower panel) representing the beginning of the SARS-CoV-2 spread at the Pernambuco state.

### 3.2. Genome Sequencing and Variability

One hundred and one SARS-CoV-2 genomes were obtained with a coverage breadth ranging from 66.47 to 99.89 and an average coverage breadth of 94. We also observed a high average coverage depth of 1826× with a standard deviation of 770× (Table S1).

Coverage depth was mostly uniform with few amplicons showing a much higher depth and few systematic gaps between the 7th and 8th kb position from the ORF1ab (Figure S1), the ORF that generate all non-structural proteins of SARS-CoV-2, and between 20–21 kb of the Spike protein ORF (S), the outermost protein in the coronavirus crown responsible for the cell surface receptor binding [40]. These systematic gaps are probably related to primer competition during the PCR reaction, which probably lead to a higher abundance of some amplicons in detriment of others. It can be clearly seen in the coverage depth plot for each genome in Supplementary File S1. On the other hand, Liu et al. 2020 [38] found recurrent deletions in the coding region of the Spike protein that may restrict late phase viral replication both in clinical and in vitro isolated viral strains [38]. In order to investigate if these natural occurring deletions could be responsible for such gap patterns seen in the Spike protein of the genomes sequenced here, we manually checked the alignment of all genomes from Pernambuco and probed our raw sequencing paired-end data. We could not detect any evidence of deletion in the Spike protein region (NSPRRAR) or (QTQTN) in ninety-six genomes where these regions were well sequenced (Supplementary File S2) identified by Liu et al. 2020 suggesting that the gap regions found in the genomes sequenced here were likely a result of primer competition and the low number of sequenced amplicons corresponding to those regions.

**Figure 2:**
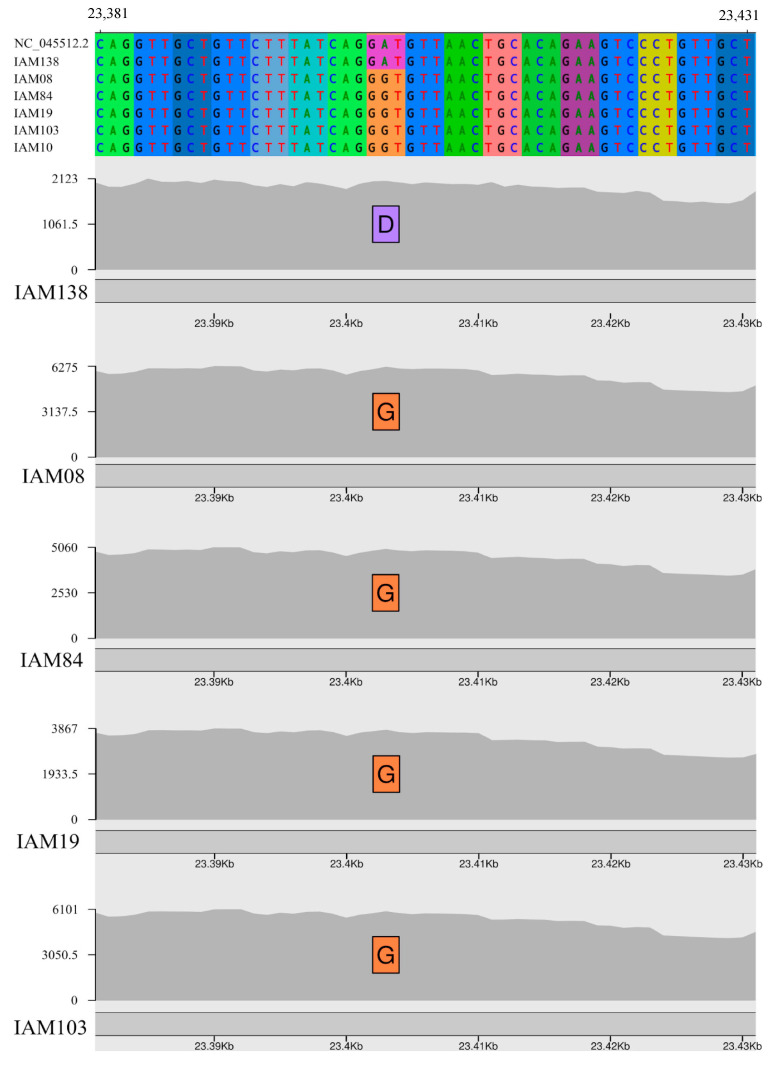
Spike protein amino acid change D614G found in the genomes sequenced in this study. Codon alignment of SARS-CoV-2 Wuhan-1 (NC_045512.2) Spike coding region showing the reference D amino acid in the IAM138 genome and the G amino acid in other four representative genomes sequenced in this study. *x* axis represents Wuhan-1 genomic coordinates and the *y* axis the coverage depth at each position.

Regarding amino acid mutations, new evidence recently emerged showing a number of important amino acid changes in the Spike protein. An amino acid change (D614G) is of particular interest since G614 lineages have consistently replaced well established D614 strains and could confer a fitness advantage of the former strains leading to a higher viral load in infected patients and higher mortality rate [41,42,43,44] although it is not clear yet if this mutation has any impact on virus transmissibility and disease progression [45]. This amino acid change is almost always accompanied by three other mutations: A non-coding nucleotide change (C-to-T) in the 5’UTR, a synonymous nucleotide mutation (C-to-T) at position 3037 and a non-synonymous mutation (C-to-T) at position 14,408 that generated an amino acid change in the RNA-dependent RNA polymerase (RdRp P323L). All genomes sequenced from the Pernambuco state showed the G614 amino acid change and the three accompanying nucleotide mutations described above, except the IAM138 strain which has all four positions identical to Wuhan-1 reference sequence (Figure 2, Supplementary File S3). Recent experimental evidence, in different human cell lines, confirmed that D614G is a key amino acid change that increases SARS-CoV-2 infectivity and when associated with I472V may increase the resistance to neutralizing antibodies [41]. Interestingly, we screened 479 SARS-CoV-2 high-quality genomes available from Brazil for such mutations (up to 1st October 2020) and 469 (98%) of those contain the D614G mutation (Table S5). Additional genomic data will be necessary to evaluate if variant G614 also replaced D614 strains in a similar way as it happened in other countries or its high prevalence is a result of a founder effect based on the importation of primarily G614 variants to Brazil.

Additionally, a rare mutation N501Y in spike protein was found in one sample (hCoV-19/Brazil/PE-IAM19/2020), this mutation is located in the domain that is responsible for ACE2 receptor binding. This amino acid substitution was reported 38 times around the world (Europe, North America and Oceania) being most frequently reported (34 x) in Victoria, Australia. Minor variants were observed in this residue G23063T, which is a nucleotide supported by a substantial fraction of the sequenced reads (>10%) but that account for less than 50% of the reads in a giving position and is not included in the final majority rule consensus. This variant was supported by roughly 21% of the reads (421/1924 reads) in the first Illumina sequencing experiment (Figure S2). Then we took two approaches to better characterize this strain. First, we sequenced the SARS-CoV-2 genome directly from the clinical sample using a different primer set [12] and we obtained similar results where G23063T was supported by around 15% (193/1271) of the reads (Figure S2). Second, we attempted to isolate this virus after the third passage in VERO CCL-81 cells and we managed to detect 75% (620/816 reads) G23073T of the reads supporting the N501Y mutation (Table S7). Interestingly, this sample was not clustered phylogenetically with other sequences containing this amino acid substitution.

The full set of nucleotide changes found in the genomes sequenced in this study can be found in Tables S3 and S7).

### 3.3. SARS-CoV-2 Lineages in Pernambuco

Comprehensive phylogenetic analysis with thousands of genomes sampled worldwide have identified two main SARS-CoV-2 lineages called A and B, which emerged during the beginning of the pandemic in the Hubei province—China [25]. Besides, a subdivision of these lineages was also proposed with five and nine sub lineages within A and B, respectively. Both A and B major lineages spread worldwide, but ongoing studies have been showing that the lineage B (particularly B1) spread and replaced the lineage A in several different countries [42]. The lineage B1, that was responsible for the Italian outbreak [46,47,48], and it later spread to other western countries in Europe and the Americas, including Brazil [13]. Up to now, few studies have investigated the SARS-CoV-2 lineages imported to Brazil and its further spread into the country. The largest study sequenced 427 genomes and analyzed 490 genomes from 21 Brazilian states detecting that only five strains belong to the lineage A while the remaining 485 belong to the B lineage [13]. These authors estimated that more than 100 international introductions of the virus occurred in Brazil. Resende et al. 2020 sequenced 95 genomes from 10 Brazilian states characterizing six SARS-CoV-2 lineages (A.2, B.1, B.1.1, B.2.1, B.2.2, and B.6). Moreover, the majority of the strains characterized were classified as clade B.1 (95%) and 92% of those belong to the sub-clade B.1.1 [12]. Lastly, Xavier et al. 2020 sequenced the genome of 40 strains of SARS-CoV-2 from Minas Gerais state where 85% of these belong to the B lineage in which most of those falls within B.1.1 and only one genome belongs to the A lineage [11].

**Figure 3:**
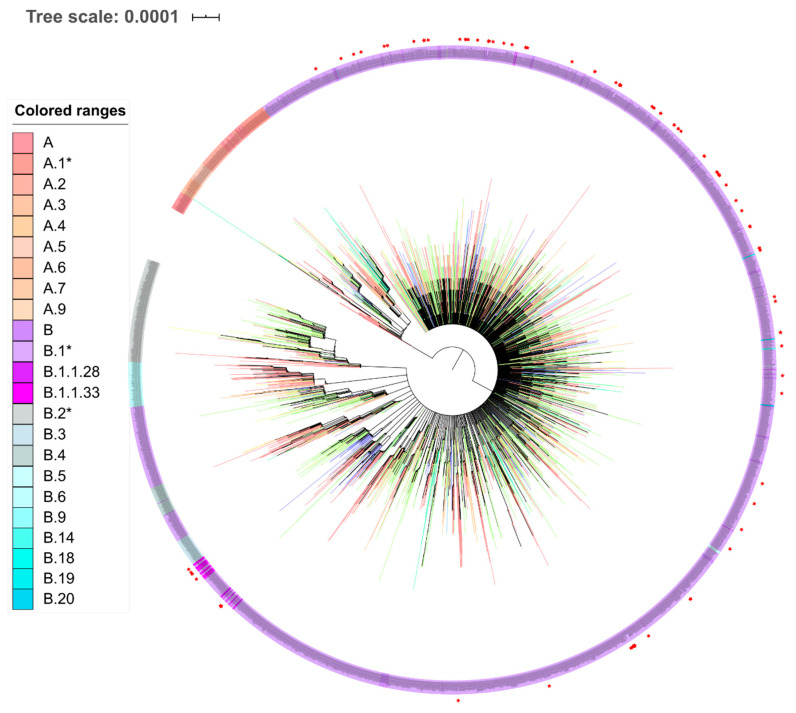
Maximum likelihood phylogenetic tree using 7264 genomes available from GISAID plus 101 genomes generated in this study (red stars) rooted between group A and B. Pangolin lineage assignments are denoted by the tip colors: Reddish are A lineages and bluish are B lineages. Branch colors follows continent of samples origin: Red—Asia; Orange—Africa; Yellow—Australia and Zealandia; Green- Europe; Pink—North America; Blue—Central America; Purple—South America.

In order to evaluate which lineages belong the genomes sequenced in this study, we performed lineages and sublineages assignment with Pangolin following Rambout et al. 2020 [25] dynamic nomenclature for SARS-CoV-2 which was depicted onto a ML phylogenetic tree reconstructed with 7264 SARS-CoV-2 reference genomes (Figure 3). All 101 viral genomes obtained from Pernambuco belong to the B lineage, one strain (IAM138) showed several nucleotide and amino acid polymorphisms that are characteristic of early diverging B lineages that circulated in China, such as the reference Wuhan-1 genome sequencing (see Genome sequencing and variability section above, Figure 2 and Figure 3 and Table S5) suggesting that this lineage is likely a representative of the early divergent SARS-CoV-2 lineages emerged from China. 

**Figure 4:**
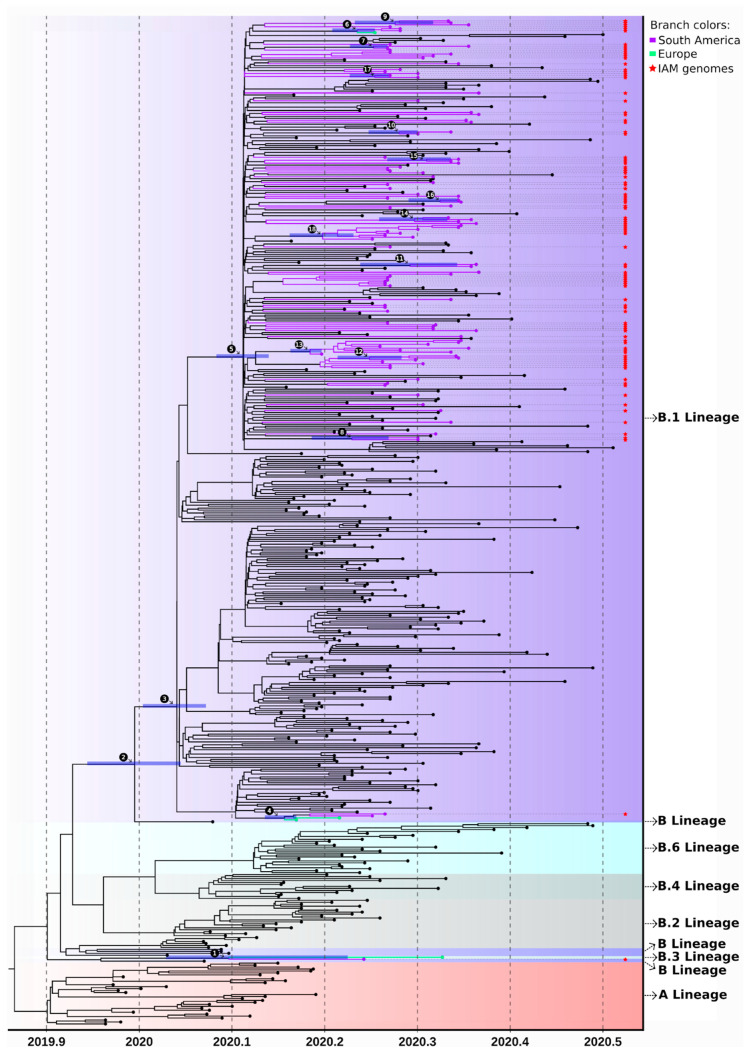
Time-resolved tree reconstructed with a Bayesian framework. Numbers above the branches are specific clades showing posterior probability higher than 70 depicted in Table S2 and horizontal bar represents the HPD95% credible interval of the estimated tMRCA. Branch colors follow continent order as in Figure 3, but only highly supported clades containing genomes obtained in this study were colored. Red stars show the SARS-CoV-2 genomes from Pernambuco state.

A second strain (IAM19) was assigned to the B.1 lineage and grouped with high branch support with two SARS-CoV-2 genomes from France and with genomes from Ceara and Minas Gerais states (ultrafast bootstrap = 55, TBE = 0.80, PP = 0.98, Figure 4, Table S6). The remaining 99 strains grouped in the B1.1 sub-lineage (Table S1 and Figure 3 and Figure 4). Sixteen of those were clustered with another single Pernambuco genome and were placed directly at a polytomic branch showing no specific clustering with other samples from the GISAID dataset. The proofreading correction of the RNA polymerase of SARS-CoV-2 genome results in a low mutation rate of this virus compared to other RNA viruses [49]. Such low variability of densely sequenced genomes from a short period of time (early pandemic phase) likely hindered the positioning of those samples since there is a low number of synapomorphic mutations for a confident phylogenetic clustering. We also found five clades supported by ultrafast bootstrap and/or TBE containing Pernambuco genomes mixed with sequences from other countries and six clades having more than 2 sequences from Pernambuco state alone (Table S6). Several sequences from Pernambuco (cluster 7—IAM17/IAM291/EPI_ISL_476371, cluster 11—IAM109 and cluster 12—IAM766) grouped with references sequence from Europe (ultrafast bootstrap = 96, 83 and 100 respectively), while IAM08 grouped with five genomes from African continent (cluster 9—ultrafast bootstrap = 97) and IAM1264 grouped with other two sequences from Brazil and a genome from Chile (cluster 8—ultrafast bootstrap = 83) (Table S6).

Time-resolved Bayesian tree reconstructions confirmed all clades (Figure 4) and the polytomic positioning of several of the genomes investigated here as shown in the ML tree including the basal positioning of IAM138 samples although with low branch support (PP = 0.75 clustering with EPI_ISL_457352 genome from Europe—Figure 4 clade 1). Besides, the estimated most recent common ancestors (tMRCA) of A and B lineages are in agreement to the emergence of the virus around December 2019. The IAM19 genome clustered with two samples from France with an estimated the tMRCA between late February and mid-March which coincides with the first official case reported (March 12). Moreover, all other sequenced strains belonging to the B1.1 lineage that were clustered in highly supported clades showed overlapping tMRCA between mid-March and mid-April (Table S2) supporting that these lineages that emerged slightly later were the most successful ones spreading in the state (Figure 4).

**Figure 5:**
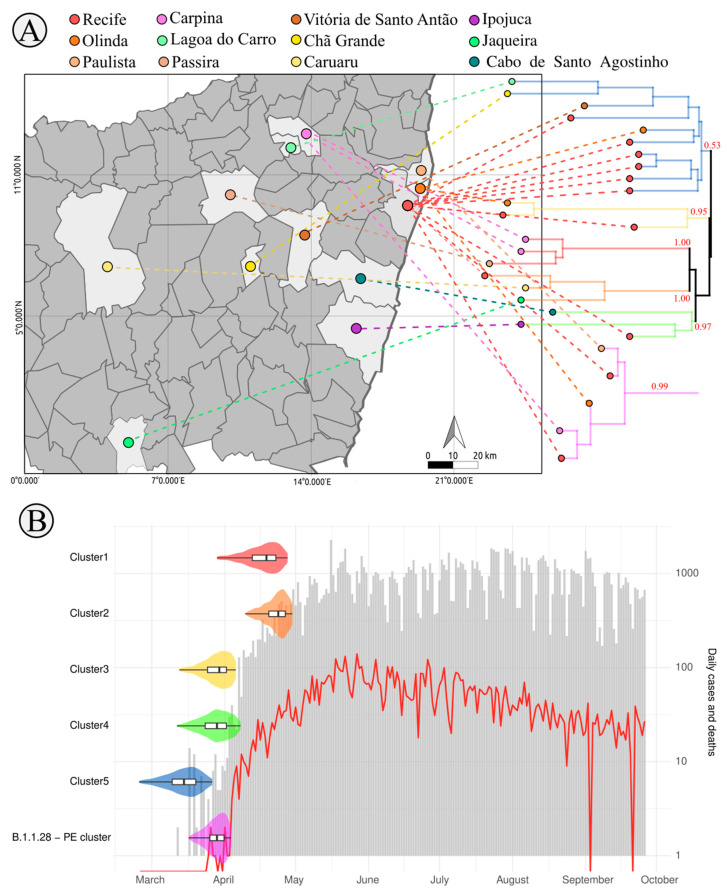
Time origin and geographical spread of local SARS-CoV-2 clusters identified in Pernambuco state. (**A**) Partial map of Pernambuco showing the municipality of origin of patients belonging to the identified clusters in the phylogeny. Circles in the map and in the tips of the phylogeny are colored according to the municipality name in the top legend. Branches in the tree are colored to distinguish each cluster whose posterior probability support is shown. (**B**) Violin plots representing each cluster tMRCA median and 95% HPD (left *y*-axis) superimposed to the daily number of new COVID-19 cases (grey bars) and deaths (red line) (right *y*-axis, log10 scale). Violin plots are colored according to the phylogeny in panel A.

Then we further accessed the phylogenetic clustering and dating of Pernambuco only clusters (six clades) showing higher branch support (ultrafast bootstrap or TBE > 40) and with more than 3 genomes suggesting successful community transmission (data showing the clusters, branch support and synapomorphies can be seen in Table S6). We performed a Bayesian phylogenetic analysis on those clades in order to better understand the SARS-CoV-2 timing and spread through different municipalities of the Pernambuco state (Figure 5A,B). Firstly, the largest Cluster 5 (composed of 10 SARS-CoV-2 genomes) have several early diverging samples from the Recife metropolitan area, including six samples from Recife and one sample from Olinda, which further seeded the transmission to small country-side cities of Vitoria de Santo Antao, Lagoa do Carro e Chã Grande. The tMRCA of this clade dated between late February to late March (Figure 5B) in line with the first cases reported in the state (12th March). Other four clusters (Clusters 1,3,4 and B.1.1.28 PE subcluster) are composed of genomes from geographically close municipalities (Figure 5A), with exception of Cluster 2 which is composed of Jaqueira, Recife and Caruaru, the last two cities are separated from each other around 135km (Figure 5A). The tMRCA of clusters 3, 4 and B.1.1.28 PE sub-cluster was dated between mid-March and early April, while the tMRCA of clusters 1 and 2 was dated between early and late April (Figure 5B). Interestingly, both B.1.1.28 PE subcluster and genomes from B.1.1.33 lineages found in our study represents less than 5% of the lineages characterized at Pernambuco while for other states they accounted to between 10 up to 80% (Parana—10, Sao Paulo—17, Sergipe—19, Minas Gerais—25, Para—33, Rio Grande do Sul—38, Santa Catarina—39, Rio de Janeiro—80%) except for Ceara state where it accounted for only 3% [12]. These lineages described for the first time in this study, suggests that the epidemic at Pernambuco was mainly driven by multiple lineages emerging locally after international introduction at the Recife metropolitan region with further spread to the state country-side cities. Recife city and its metropolitan area receive thousands of Brazilian and foreign tourists every year through the largest national and international airport of the Northeast region besides the intense road flux between Northeast state capitals. Such intense and clustered human mobility hub probably contributed to the emergence of several different lineages of SARS-CoV-2 in the state before non-pharmaceutical interventions were set. Such lineages established transmission chains between municipalities of the Pernambuco state corroborating the exponential spread within the state soon after the first cases were reported (Figure 5B).

Overall, our results showed five new international introduction events of the SARS-CoV-2 into Pernambuco that originated local clusters that were successful in spreading throughout several cities of the state. Although not formally tested, the origin of such strains is likely Europe, whose sequences dominated the base of the B.1.1 cluster. Simultaneously to the arrival of international strains, national lineages (e.g., B.1.1.28 and B.1.1.33) also reached Pernambuco in the beginning of the epidemic, highlighting the fast dissemination capacity of SARS-CoV-2 and bringing new insights about the importance of restrictions to people movement within the country. Besides, the lineage assignment further supports that the B lineage is prevailing through community spread in Brazil in line with other SARS-CoV-2 studies.

## 4. Conclusions

The genomic analysis of 101 SARS-CoV-2 genomes from the beginning of the epidemic at Pernambuco state, Brazil, revealed at least five independent international importation events of SARS-CoV-2 strains, that effectively seeded community transmission, occurred from late February to mid-April which is in line with studies focusing on other Brazilian states. Moreover, we also found evidence of community spread of at least six local lineages, defined here as highly supported clades with synapomorphic mutations and with three or more local sequences, among Recife city and other country-side municipalities. One of these was the Brazilian B.1.1.28 lineage, whereas five were newly detected in this study. Interestingly, all genomes belong to the B lineage, with a higher prevalence of B1.1 lineage, which was shown to be the most prevalent currently circulating in Brazil. All except one genome have the G614 amino acid change which has been suggested to increase the viral fitness allowing to reach a higher viral load in an experimental setting and likely in human patients. G614 strains have replaced D614 strains in several regions around the globe where the second was first established. The high prevalence of G614 strains in our dataset suggests two possible explanations: I—most strains that entered Brazil belonged to G614 strains or II—D614 and G614 were equally imported into Brazil but G614 became prevalent as it occurred in European and North American countries. Our study highlights the importance of international traveling in seeding SARS-CoV-2 in the state of Pernambuco, fostering the emergence of local lineages and the community spread. Continued genomic-based surveillance of SARS-CoV-2 in a much broader set of samples is needed in order to access the viral mutational spectra and provide data to tease apart those hypotheses that will likely impact the control measures set to curb the epidemic.

## Data availability

All genomes generated in this study are deposited on GISAID under the accessions EPI_ISL_500460-500486 and EPI_ISL_500865-500875.

## Supplementary Materials

The following are available online at https://www.mdpi.com/1999-4915/12/12/1414/s1, Figure S1: SARS-CoV-2 genome coverage breadth of each sample sequenced in this study, Figure S2: Integrated Genome Viewer screenshot of minor variants found in the sample IAM19, Table S1: General information about the 101 sequenced genomes, Table S2: Number of GISAID genomes by country after clusterization, Table S3: SNPs found and coverage depth, Table S4: General metrics of SNPs by genomic regions, Table S5: Annoation of mutations on high quality Brazilian genomes, Table S6: Synapomorphic SNPs found in monophyletic clusters, Table S7: Minor variants identified in the 101 new genomes sequenced, File S1: Sequencing coverage depth plot for each sample studied, File S2: Sequencing coverage depth of the 23-24kb SARS-CoV-2 region (Spike protein coding region) where Liu et al. 2020 found recurrent deletions, File S3: Nucleotide and amino acid alignment highlighting the characteristic changes that a accompany the D614G Spike amino acid change, File S4: Acknowledge of authors and originating laboratories that shared SARS-CoV-2 genomes in GISAID database.
