# Supplementary material for: Multiple Introductions Followed by Ongoing Community Spread of SARS-CoV-2 at One of the Largest Metropolitan Areas of Northeast Brazil
Source: Viruses. 2020 Dec 9;12(12):1414. doi: 10.3390/v12121414 (PMC7763515; doi:10.3390/v12121414)
Supplement: Supplementary file 1 [file viruses-12-01414-s001.zip › Supplementary_Material/Supplementary_File_3.pdf]

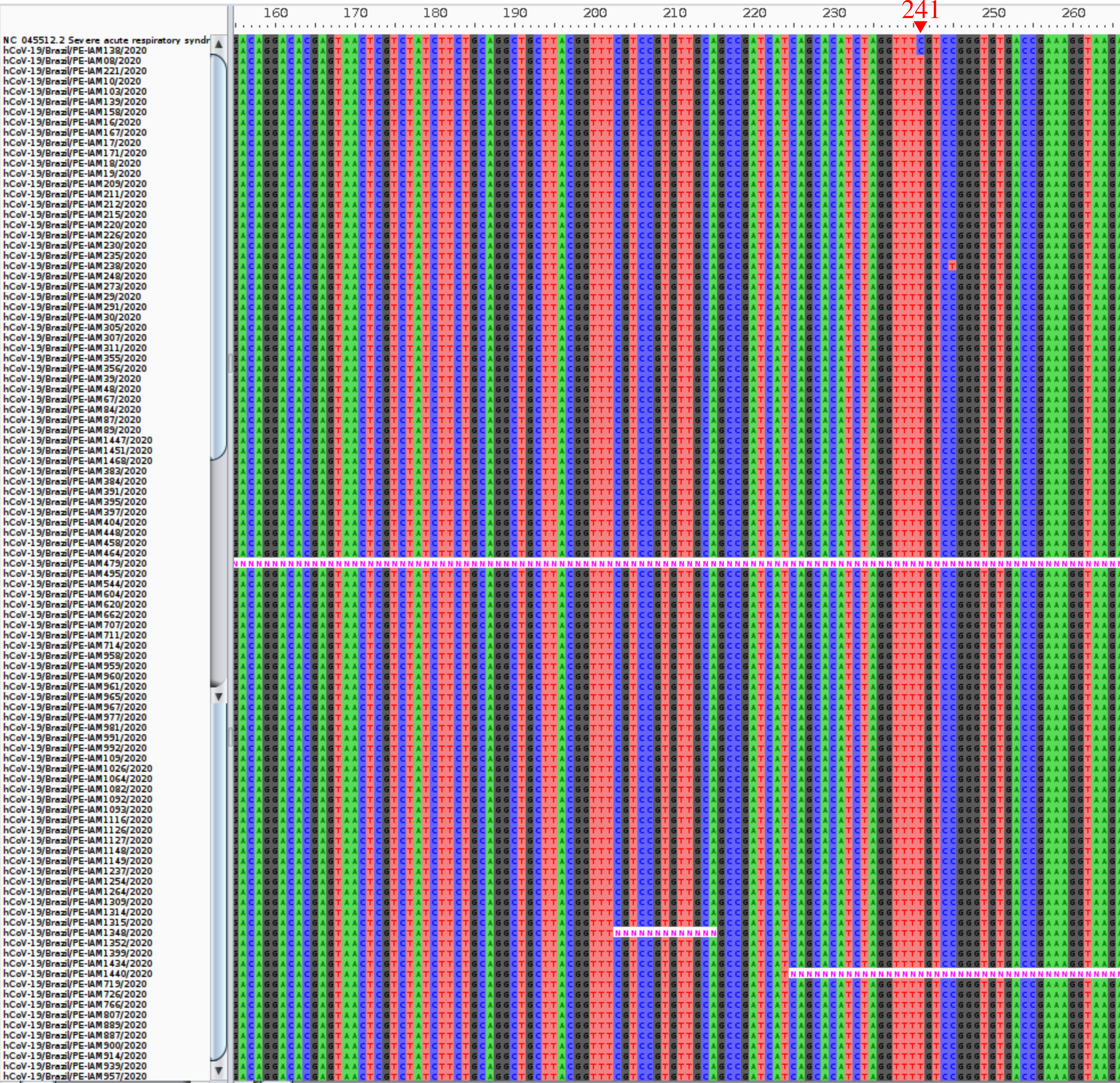
160
170
180
190
200
210
220
230
241
250
260
NC 045512.2 Severe acute respiratory syndr
hCoV-19/Brazil/PE-IAM138/2020
hCoV-19/Brazil/PE-IAM08/2020
hCoV-19/Brazil/PE-IAM221/2020
hCoV-19/Brazil/PE-IAM10/2020
hCoV-19/Brazil/PE-IAM103/2020
hCoV-19/Brazil/PE-IAM139/2020
hCoV-19/Brazil/PE-IAM158/2020
hCoV-19/Brazil/PE-IAM16/2020
hCoV-19/Brazil/PE-IAM167/2020
hCoV-19/Brazil/PE-IAM17/2020
hCoV-19/Brazil/PE-IAM171/2020
hCoV-19/Brazil/PE-IAM18/2020
hCoV-19/Brazil/PE-IAM19/2020
hCoV-19/Brazil/PE-IAM209/2020
hCoV-19/Brazil/PE-IAM211/2020
hCoV-19/Brazil/PE-IAM212/2020
hCoV-19/Brazil/PE-IAM215/2020
hCoV-19/Brazil/PE-IAM220/2020
hCoV-19/Brazil/PE-IAM226/2020
hCoV-19/Brazil/PE-IAM230/2020
hCoV-19/Brazil/PE-IAM235/2020
hCoV-19/Brazil/PE-IAM238/2020
hCoV-19/Brazil/PE-IAM248/2020
hCoV-19/Brazil/PE-IAM273/2020
hCoV-19/Brazil/PE-IAM29/2020
hCoV-19/Brazil/PE-IAM291/2020
hCoV-19/Brazil/PE-IAM30/2020
hCoV-19/Brazil/PE-IAM305/2020
hCoV-19/Brazil/PE-IAM307/2020
hCoV-19/Brazil/PE-IAM311/2020
hCoV-19/Brazil/PE-IAM355/2020
hCoV-19/Brazil/PE-IAM356/2020
hCoV-19/Brazil/PE-IAM39/2020
hCoV-19/Brazil/PE-IAM48/2020
hCoV-19/Brazil/PE-IAM67/2020
hCoV-19/Brazil/PE-IAM84/2020
hCoV-19/Brazil/PE-IAM87/2020
hCoV-19/Brazil/PE-IAM89/2020
hCoV-19/Brazil/PE-IAM1447/2020
hCoV-19/Brazil/PE-IAM1451/2020
hCoV-19/Brazil/PE-IAM1468/2020
hCoV-19/Brazil/PE-IAM383/2020
hCoV-19/Brazil/PE-IAM384/2020
hCoV-19/Brazil/PE-IAM391/2020
hCoV-19/Brazil/PE-IAM395/2020
hCoV-19/Brazil/PE-IAM397/2020
hCoV-19/Brazil/PE-IAM404/2020
hCoV-19/Brazil/PE-IAM448/2020
hCoV-19/Brazil/PE-IAM458/2020
hCoV-19/Brazil/PE-IAM464/2020
hCoV-19/Brazil/PE-IAM479/2020
hCoV-19/Brazil/PE-IAM495/2020
hCoV-19/Brazil/PE-IAM544/2020
hCoV-19/Brazil/PE-IAM604/2020
hCoV-19/Brazil/PE-IAM620/2020
hCoV-19/Brazil/PE-IAM662/2020
hCoV-19/Brazil/PE-IAM707/2020
hCoV-19/Brazil/PE-IAM711/2020
hCoV-19/Brazil/PE-IAM714/2020
hCoV-19/Brazil/PE-IAM958/2020
hCoV-19/Brazil/PE-IAM959/2020
hCoV-19/Brazil/PE-IAM960/2020
hCoV-19/Brazil/PE-IAM961/2020
hCoV-19/Brazil/PE-IAM965/2020
hCoV-19/Brazil/PE-IAM967/2020
hCoV-19/Brazil/PE-IAM977/2020
hCoV-19/Brazil/PE-IAM981/2020
hCoV-19/Brazil/PE-IAM991/2020
hCoV-19/Brazil/PE-IAM992/2020
hCoV-19/Brazil/PE-IAM109/2020
hCoV-19/Brazil/PE-IAM1026/2020
hCoV-19/Brazil/PE-IAM1064/2020
hCoV-19/Brazil/PE-IAM1082/2020
hCoV-19/Brazil/PE-IAM1092/2020
hCoV-19/Brazil/PE-IAM1093/2020
hCoV-19/Brazil/PE-IAM1116/2020
hCoV-19/Brazil/PE-IAM1126/2020
hCoV-19/Brazil/PE-IAM1127/2020
hCoV-19/Brazil/PE-IAM1148/2020
hCoV-19/Brazil/PE-IAM1149/2020
hCoV-19/Brazil/PE-IAM1237/2020
hCoV-19/Brazil/PE-IAM1254/2020
hCoV-19/Brazil/PE-IAM1264/2020
hCoV-19/Brazil/PE-IAM1309/2020
hCoV-19/Brazil/PE-IAM1314/2020
hCoV-19/Brazil/PE-IAM1315/2020
hCoV-19/Brazil/PE-IAM1348/2020
hCoV-19/Brazil/PE-IAM1352/2020
hCoV-19/Brazil/PE-IAM1399/2020
hCoV-19/Brazil/PE-IAM1434/2020
hCoV-19/Brazil/PE-IAM1440/2020
hCoV-19/Brazil/PE-IAM719/2020
hCoV-19/Brazil/PE-IAM726/2020
hCoV-19/Brazil/PE-IAM766/2020
hCoV-19/Brazil/PE-IAM807/2020
hCoV-19/Brazil/PE-IAM889/2020
hCoV-19/Brazil/PE-IAM887/2020
hCoV-19/Brazil/PE-IAM900/2020
hCoV-19/Brazil/PE-IAM914/2020
hCoV-19/Brazil/PE-IAM939/2020
hCoV-19/Brazil/PE-IAM957/2020

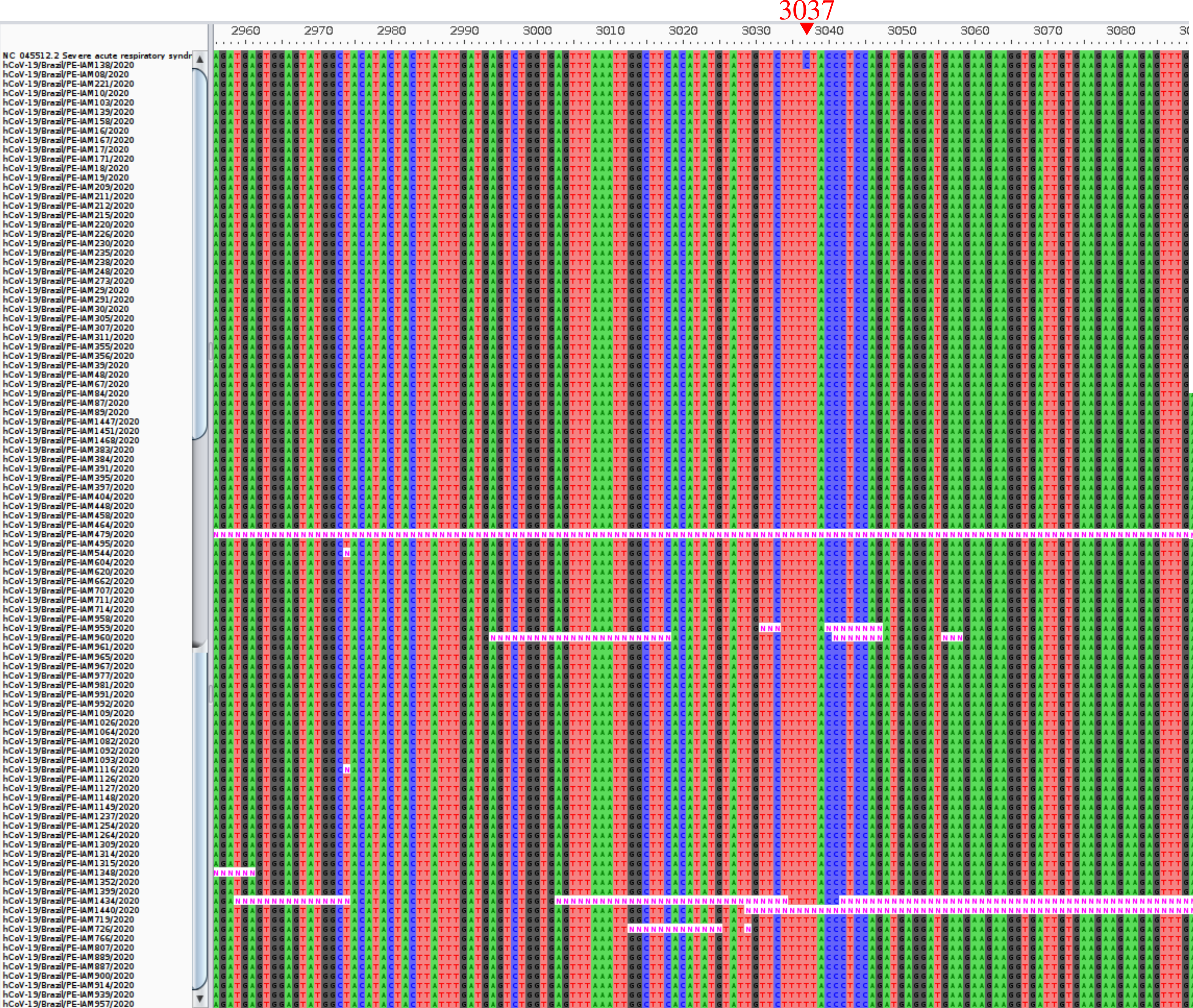
3037
2960
2970
2980
2990
3000
3010
3020
3030
3040
3050
3060
3070
3080
NC 045512.2 Severe acute respiratory syndr

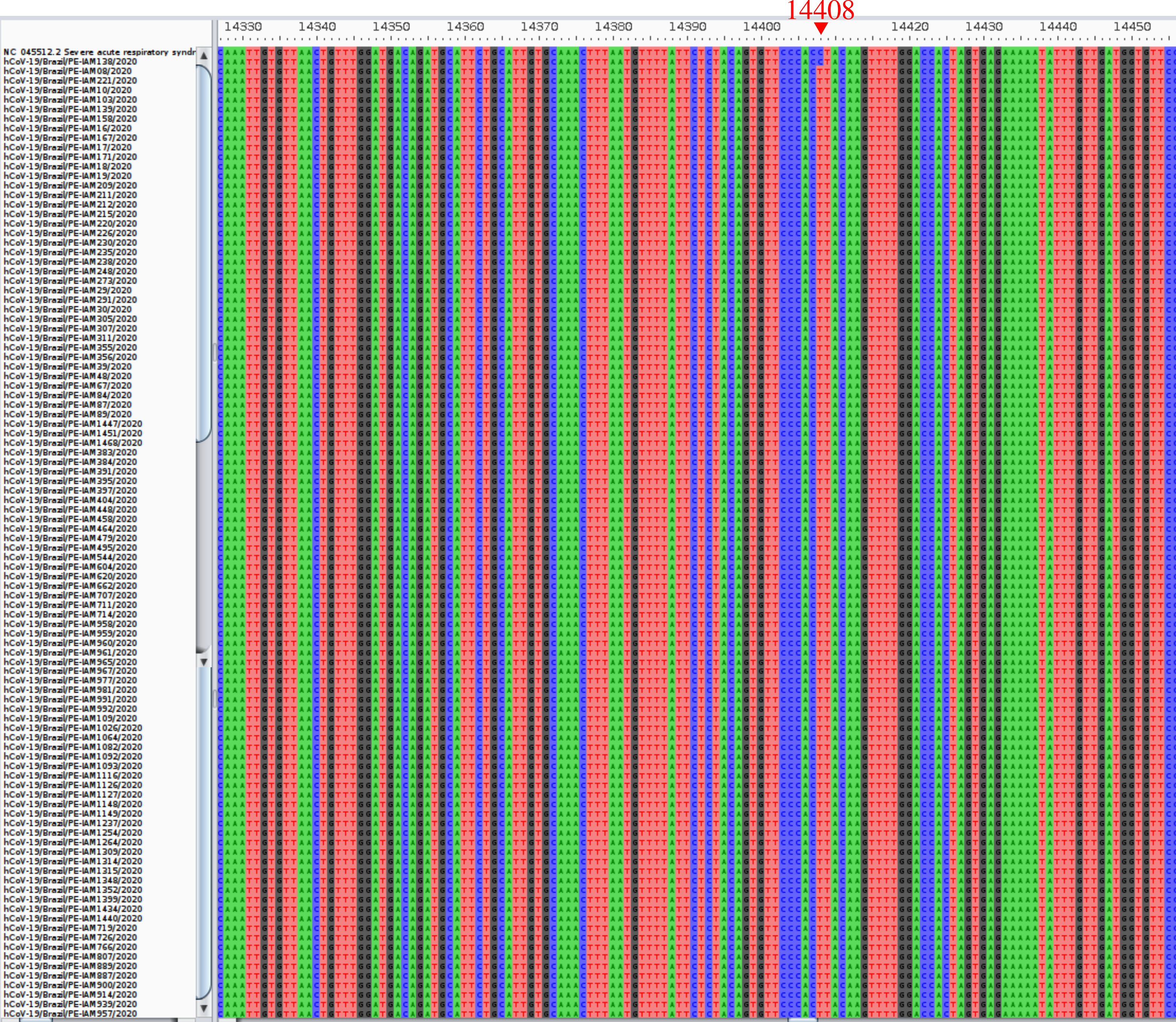
14408
14330
14340
14350
14360
14370
14380
14390
14400
14420
14430
14440
14450
NC 045512.2 Severe acute respiratory syndr
hCoV-19/Brazil/PE-IAM138/2020
hCoV-19/Brazil/PE-IAM08/2020
hCoV-19/Brazil/PE-IAM221/2020
hCoV-19/Brazil/PE-IAM10/2020
hCoV-19/Brazil/PE-IAM103/2020
hCoV-19/Brazil/PE-IAM139/2020
hCoV-19/Brazil/PE-IAM158/2020
hCoV-19/Brazil/PE-IAM16/2020
hCoV-19/Brazil/PE-IAM167/2020
hCoV-19/Brazil/PE-IAM17/2020
hCoV-19/Brazil/PE-IAM171/2020
hCoV-19/Brazil/PE-IAM18/2020
hCoV-19/Brazil/PE-IAM19/2020
hCoV-19/Brazil/PE-IAM209/2020
hCoV-19/Brazil/PE-IAM211/2020
hCoV-19/Brazil/PE-IAM212/2020
hCoV-19/Brazil/PE-IAM215/2020
hCoV-19/Brazil/PE-IAM220/2020
hCoV-19/Brazil/PE-IAM226/2020
hCoV-19/Brazil/PE-IAM230/2020
hCoV-19/Brazil/PE-IAM235/2020
hCoV-19/Brazil/PE-IAM238/2020
hCoV-19/Brazil/PE-IAM248/2020
hCoV-19/Brazil/PE-IAM273/2020
hCoV-19/Brazil/PE-IAM29/2020
hCoV-19/Brazil/PE-IAM291/2020
hCoV-19/Brazil/PE-IAM30/2020
hCoV-19/Brazil/PE-IAM305/2020
hCoV-19/Brazil/PE-IAM307/2020
hCoV-19/Brazil/PE-IAM311/2020
hCoV-19/Brazil/PE-IAM355/2020
hCoV-19/Brazil/PE-IAM356/2020
hCoV-19/Brazil/PE-IAM39/2020
hCoV-19/Brazil/PE-IAM48/2020
hCoV-19/Brazil/PE-IAM67/2020
hCoV-19/Brazil/PE-IAM84/2020
hCoV-19/Brazil/PE-IAM87/2020
hCoV-19/Brazil/PE-IAM89/2020
hCoV-19/Brazil/PE-IAM1447/2020
hCoV-19/Brazil/PE-IAM1451/2020
hCoV-19/Brazil/PE-IAM1468/2020
hCoV-19/Brazil/PE-IAM383/2020
hCoV-19/Brazil/PE-IAM384/2020
hCoV-19/Brazil/PE-IAM391/2020
hCoV-19/Brazil/PE-IAM395/2020
hCoV-19/Brazil/PE-IAM397/2020
hCoV-19/Brazil/PE-IAM404/2020
hCoV-19/Brazil/PE-IAM448/2020
hCoV-19/Brazil/PE-IAM458/2020
hCoV-19/Brazil/PE-IAM464/2020
hCoV-19/Brazil/PE-IAM479/2020
hCoV-19/Brazil/PE-IAM495/2020
hCoV-19/Brazil/PE-IAM544/2020
hCoV-19/Brazil/PE-IAM604/2020
hCoV-19/Brazil/PE-IAM620/2020
hCoV-19/Brazil/PE-IAM662/2020
hCoV-19/Brazil/PE-IAM707/2020
hCoV-19/Brazil/PE-IAM711/2020
hCoV-19/Brazil/PE-IAM714/2020
hCoV-19/Brazil/PE-IAM958/2020
hCoV-19/Brazil/PE-IAM959/2020
hCoV-19/Brazil/PE-IAM960/2020
hCoV-19/Brazil/PE-IAM961/2020
hCoV-19/Brazil/PE-IAM965/2020
hCoV-19/Brazil/PE-IAM967/2020
hCoV-19/Brazil/PE-IAM977/2020
hCoV-19/Brazil/PE-IAM981/2020
hCoV-19/Brazil/PE-IAM991/2020
hCoV-19/Brazil/PE-IAM992/2020
hCoV-19/Brazil/PE-IAM109/2020
hCoV-19/Brazil/PE-IAM1026/2020
hCoV-19/Brazil/PE-IAM1064/2020
hCoV-19/Brazil/PE-IAM1082/2020
hCoV-19/Brazil/PE-IAM1092/2020
hCoV-19/Brazil/PE-IAM1093/2020
hCoV-19/Brazil/PE-IAM1116/2020
hCoV-19/Brazil/PE-IAM1126/2020
hCoV-19/Brazil/PE-IAM1127/2020
hCoV-19/Brazil/PE-IAM1148/2020
hCoV-19/Brazil/PE-IAM1149/2020
hCoV-19/Brazil/PE-IAM1237/2020
hCoV-19/Brazil/PE-IAM1254/2020
hCoV-19/Brazil/PE-IAM1264/2020
hCoV-19/Brazil/PE-IAM1309/2020
hCoV-19/Brazil/PE-IAM1314/2020
hCoV-19/Brazil/PE-IAM1315/2020
hCoV-19/Brazil/PE-IAM1348/2020
hCoV-19/Brazil/PE-IAM1352/2020
hCoV-19/Brazil/PE-IAM1399/2020
hCoV-19/Brazil/PE-IAM1434/2020
hCoV-19/Brazil/PE-IAM1440/2020
hCoV-19/Brazil/PE-IAM719/2020
hCoV-19/Brazil/PE-IAM726/2020
hCoV-19/Brazil/PE-IAM766/2020
hCoV-19/Brazil/PE-IAM807/2020
hCoV-19/Brazil/PE-IAM889/2020
hCoV-19/Brazil/PE-IAM887/2020
hCoV-19/Brazil/PE-IAM900/2020
hCoV-19/Brazil/PE-IAM914/2020
hCoV-19/Brazil/PE-IAM939/2020
hCoV-19/Brazil/PE-IAM957/2020

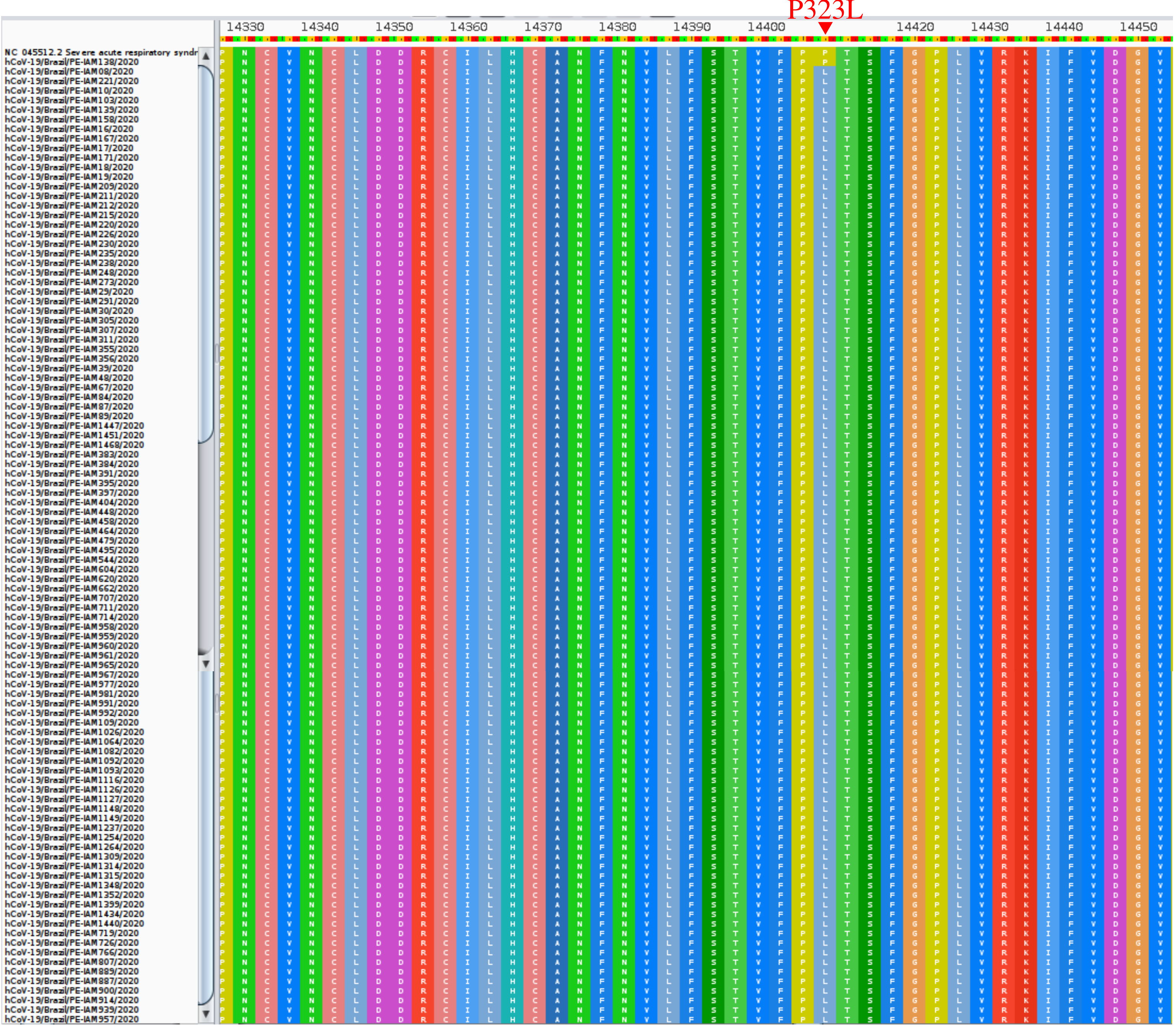
P323L
14330
14340
14350
14360
14370
14380
14390
14400
14420
14430
14440
14450
NC 045512.2 Severe acute respiratory syndr
