# Supplementary figures and images for: Multiple Introductions Followed by Ongoing Community Spread of SARS-CoV-2 at One of the Largest Metropolitan Areas of Northeast Brazil
Source: Viruses. 2020 Dec 9;12(12):1414. doi: 10.3390/v12121414 (PMC7763515; doi:10.3390/v12121414)

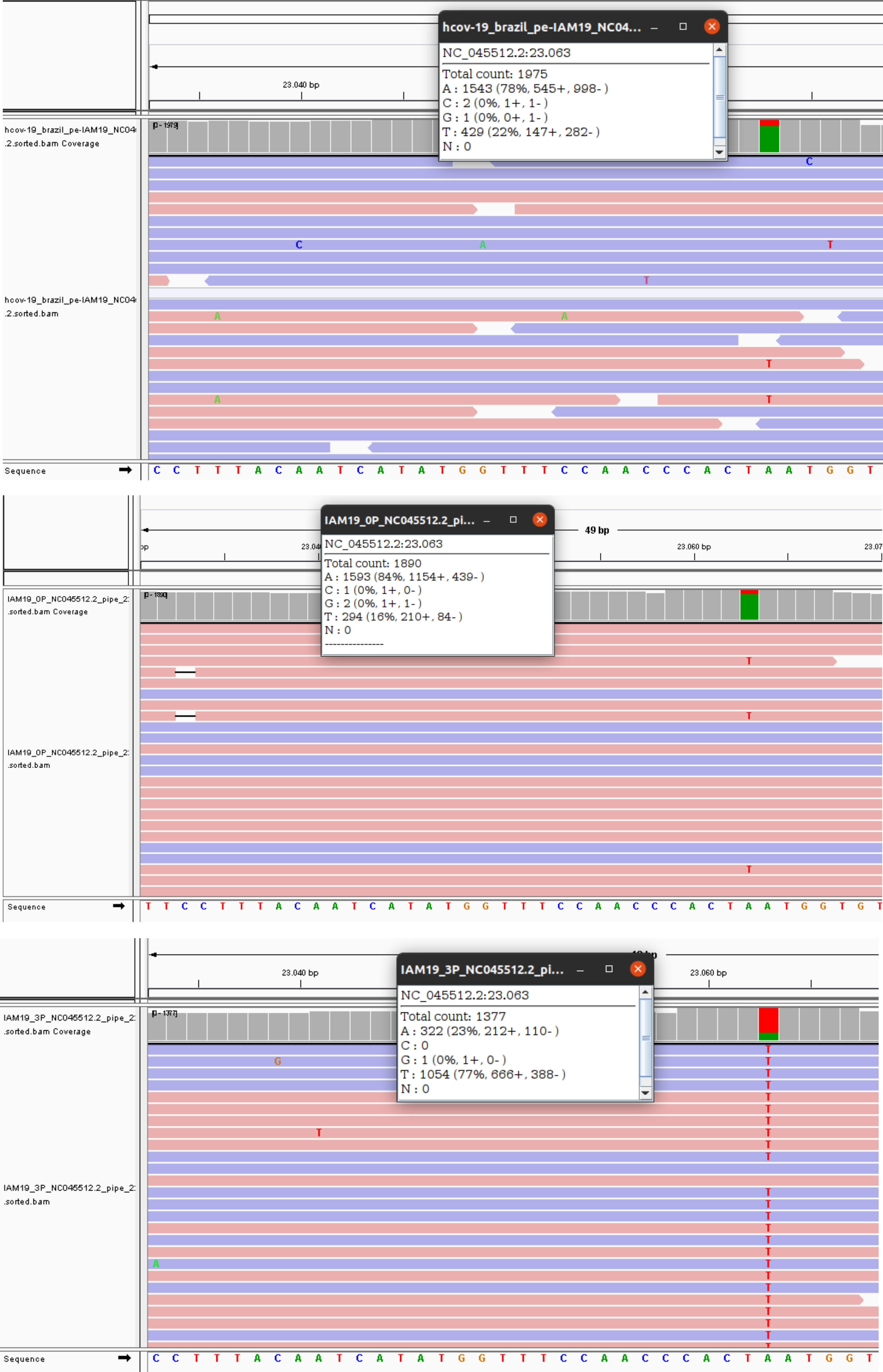

Supplement: Supplementary file 1 [file viruses-12-01414-s001.zip › Supplementary_Material/Figure_S2.png]

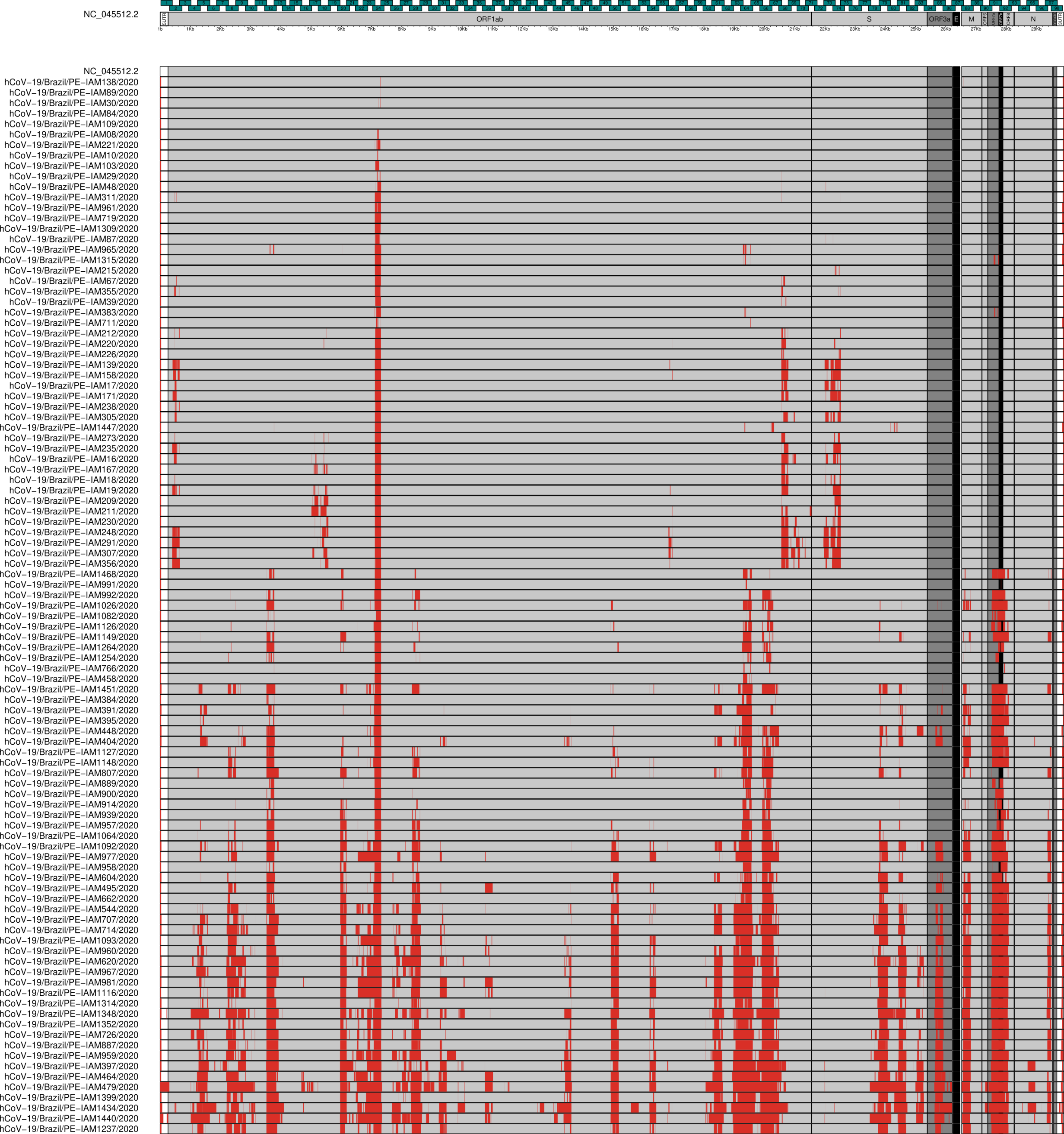

Supplement: Supplementary file 1 [file viruses-12-01414-s001.zip › Supplementary_Material/Figure_S1.png]

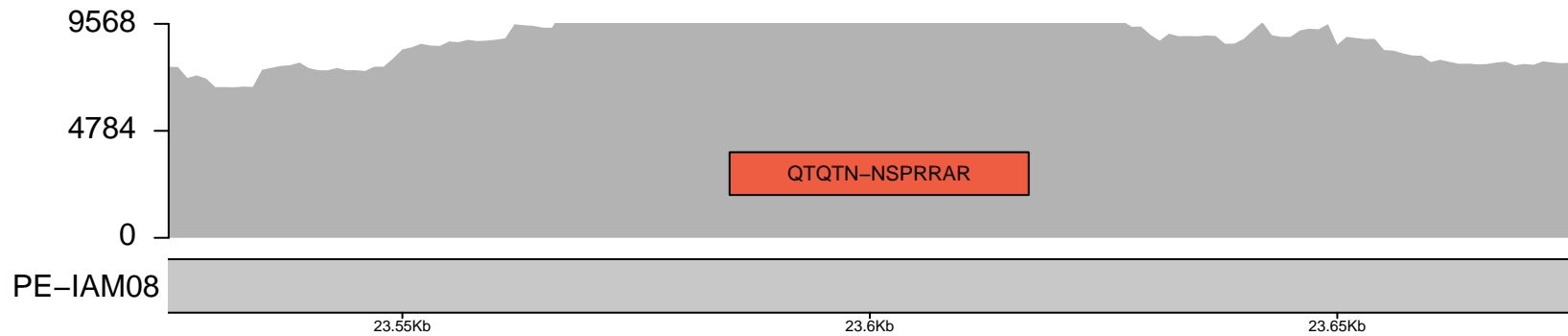

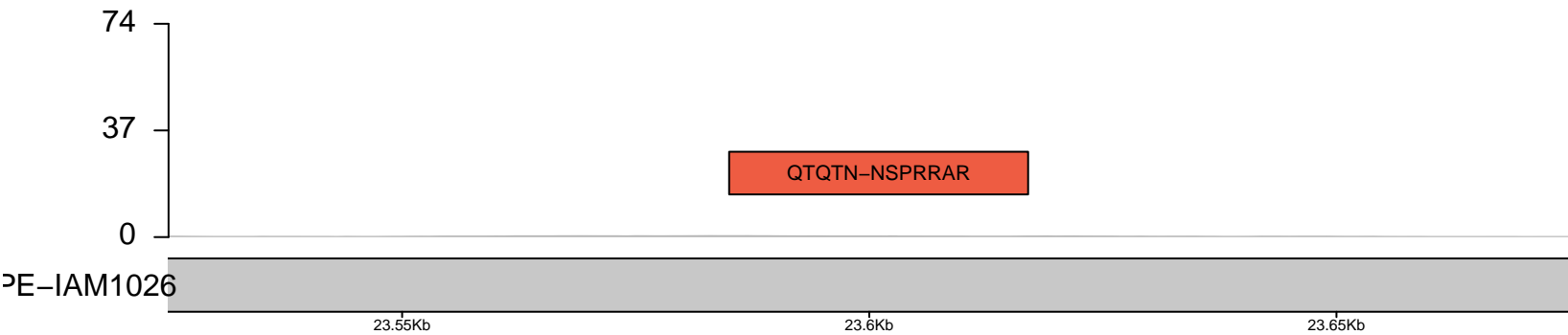

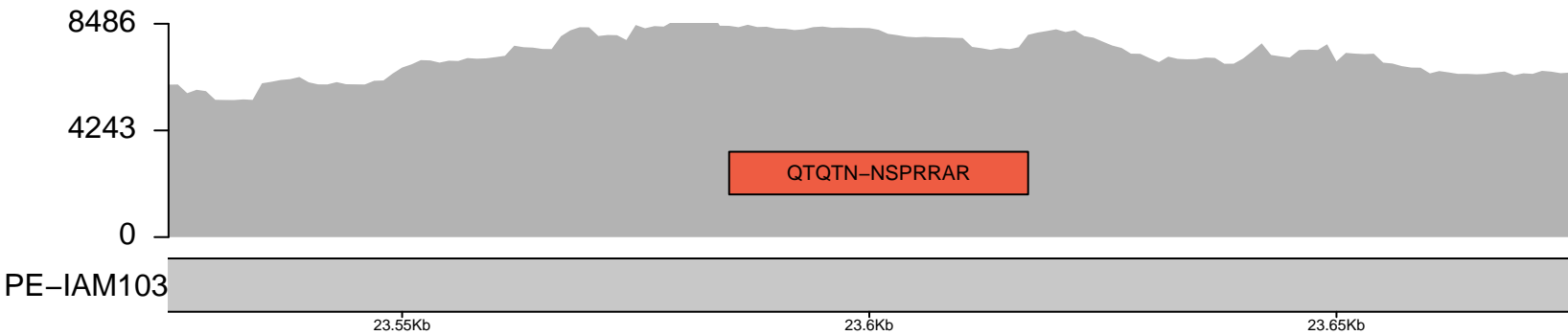

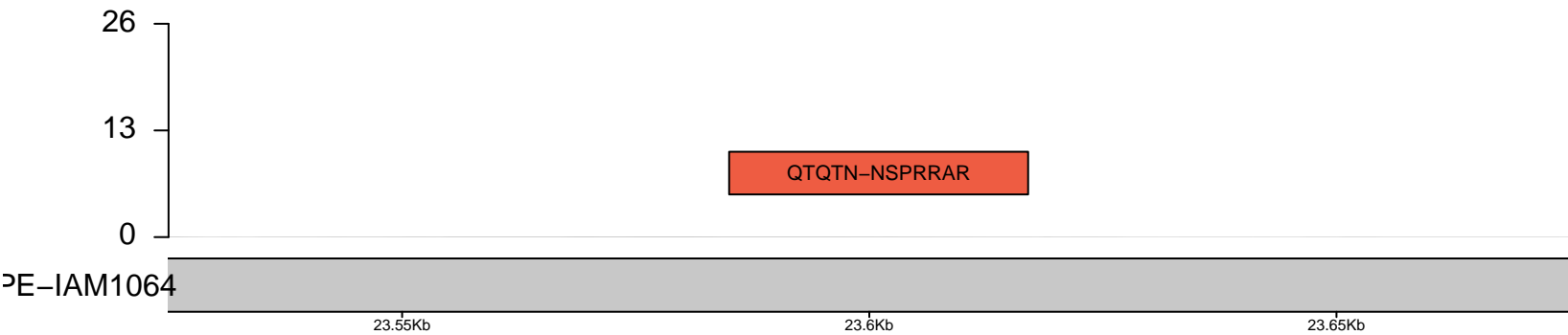

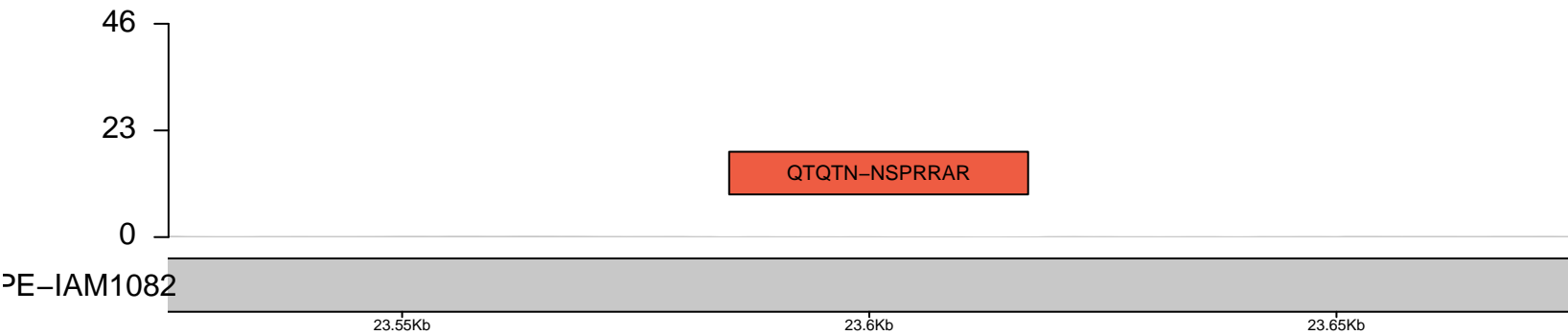

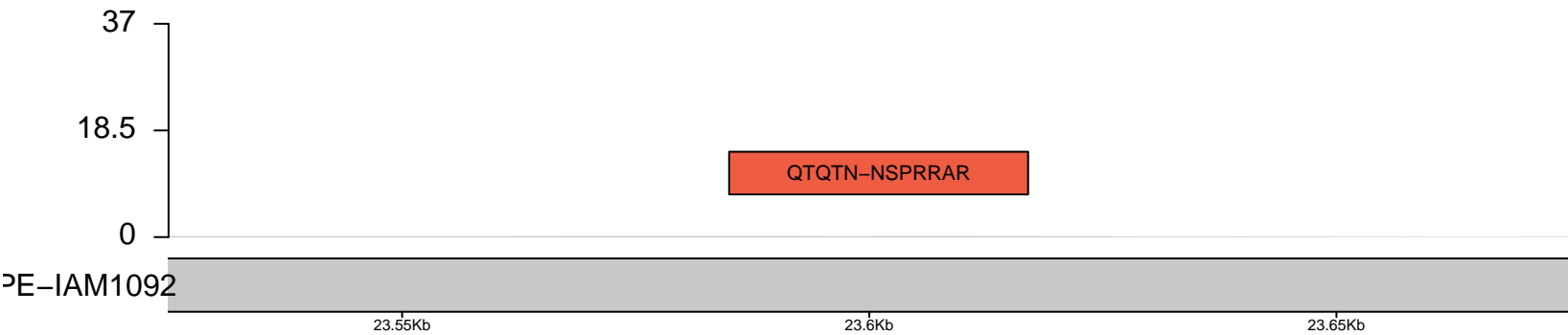

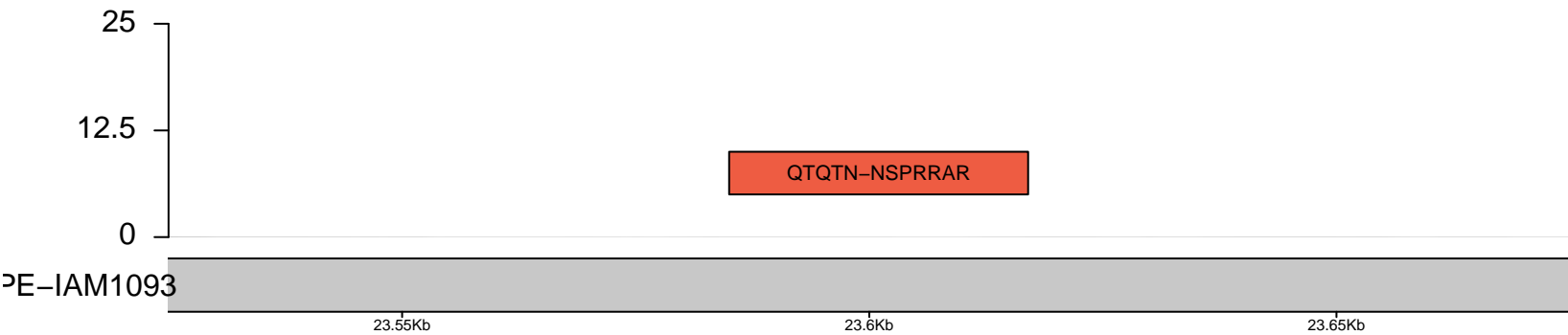

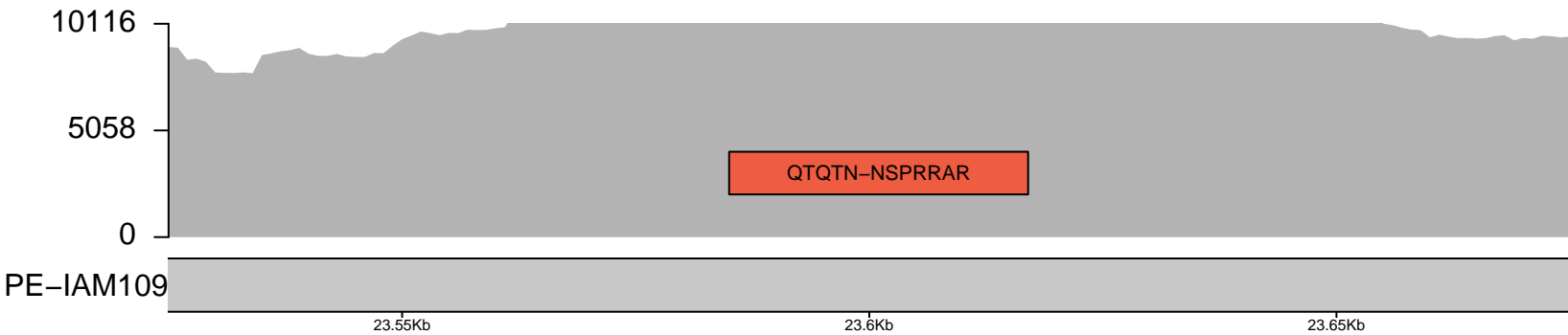

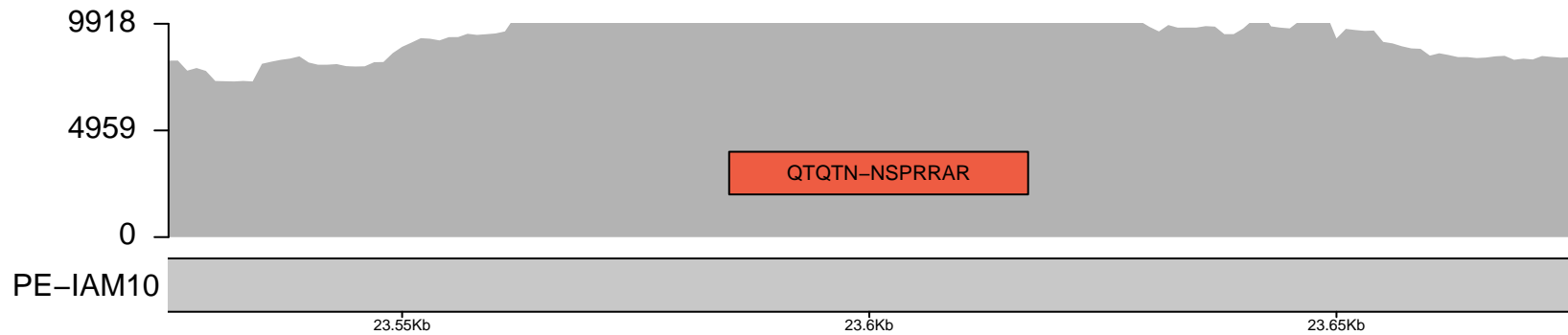

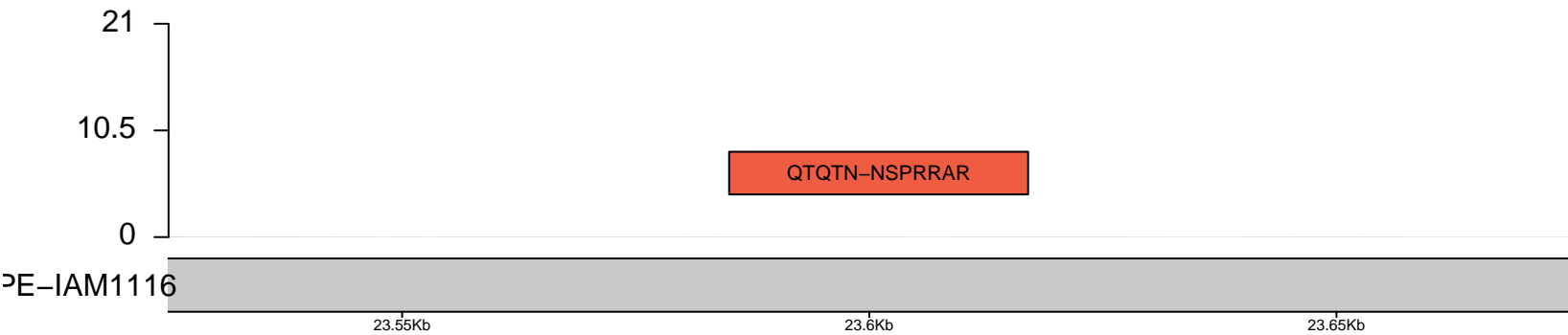

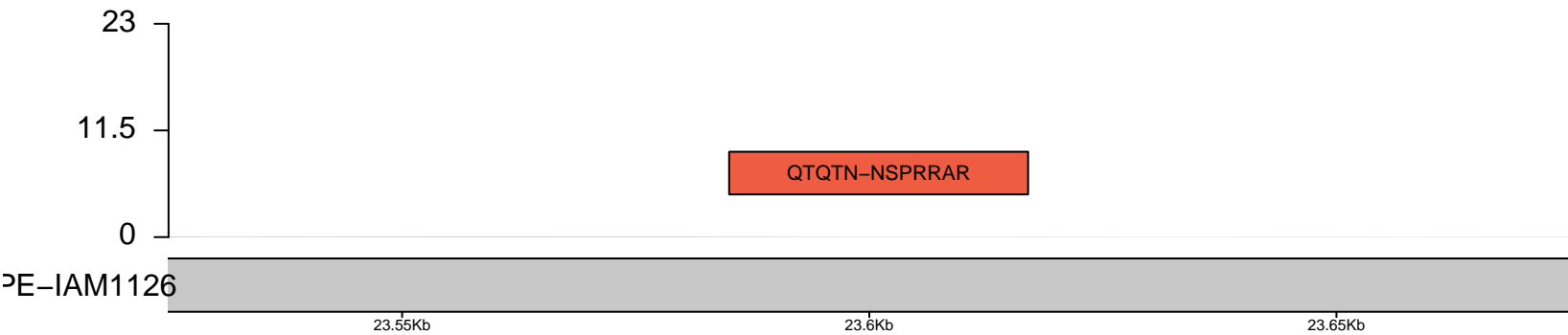

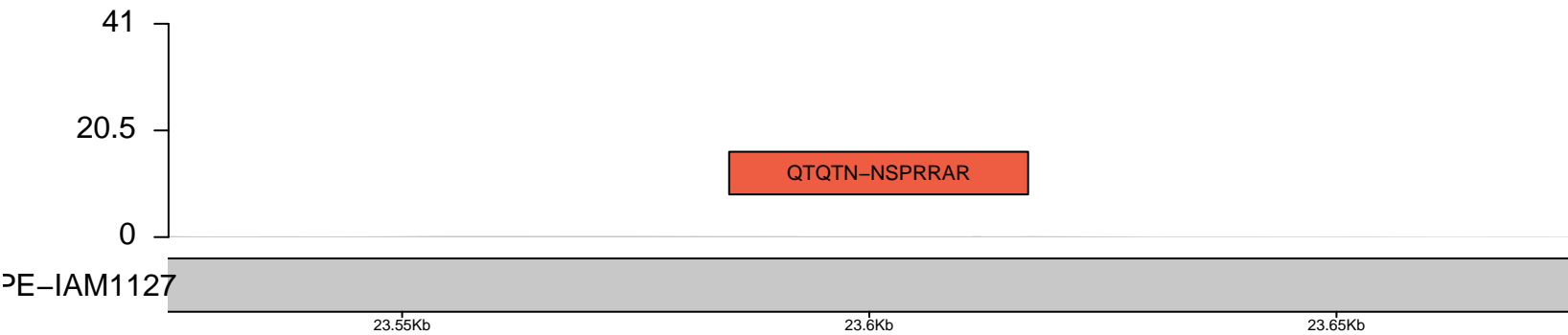

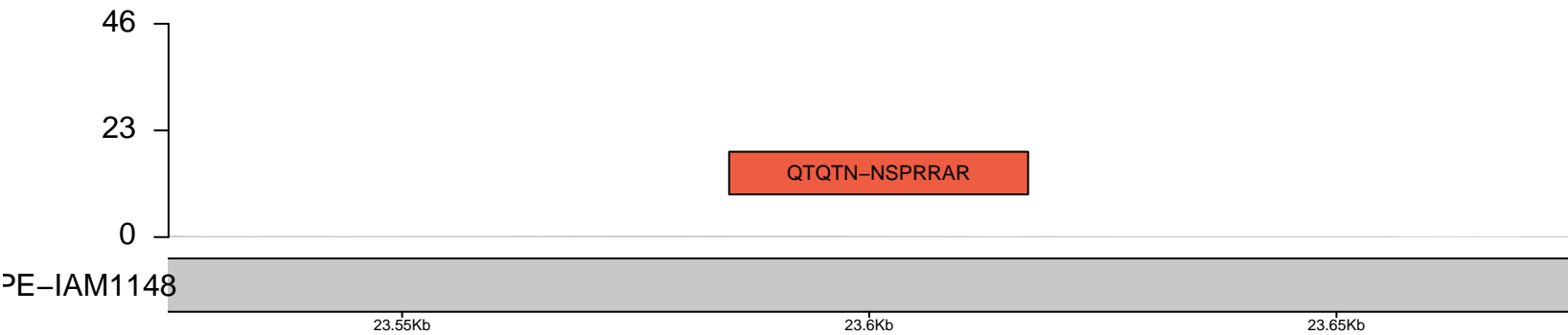

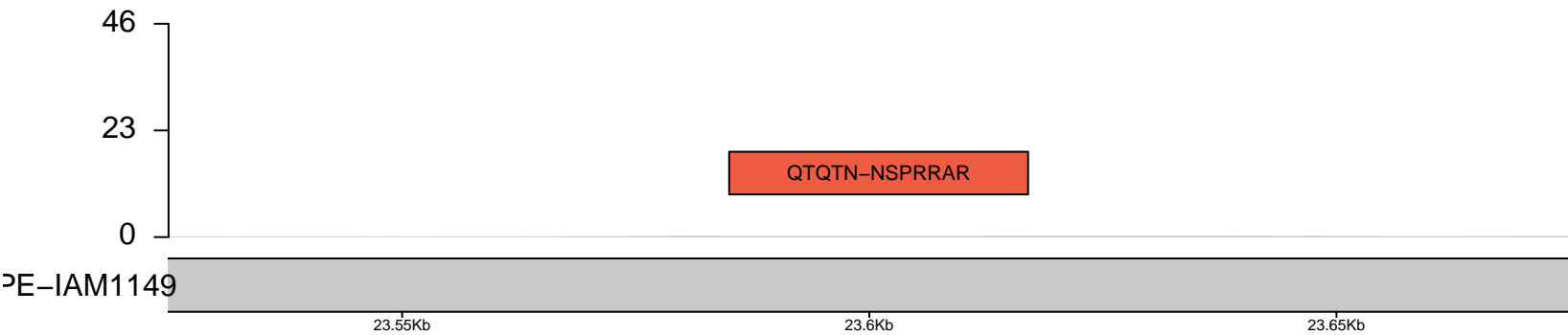

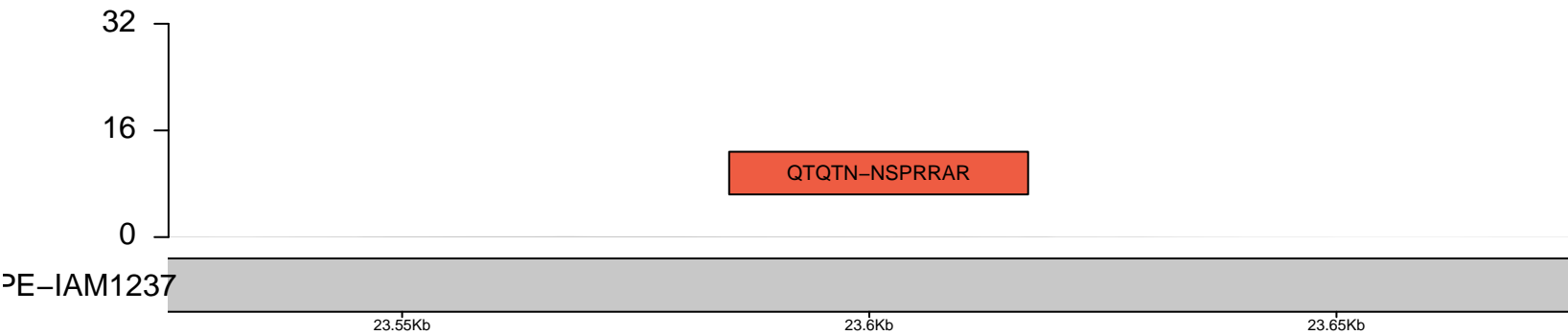

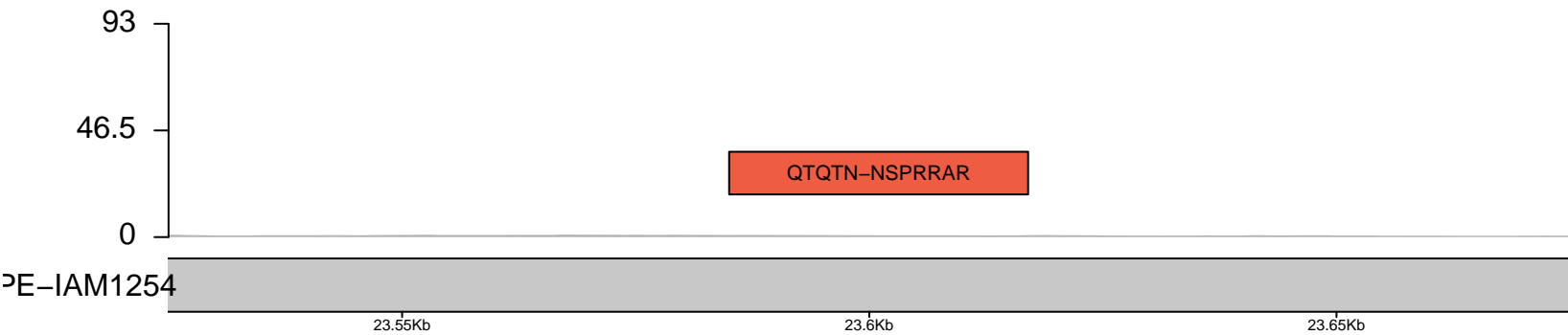

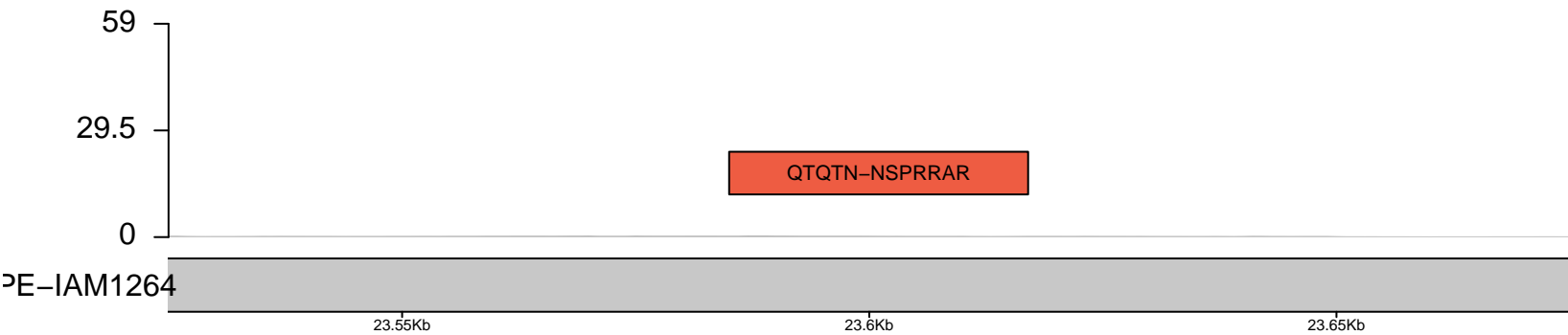

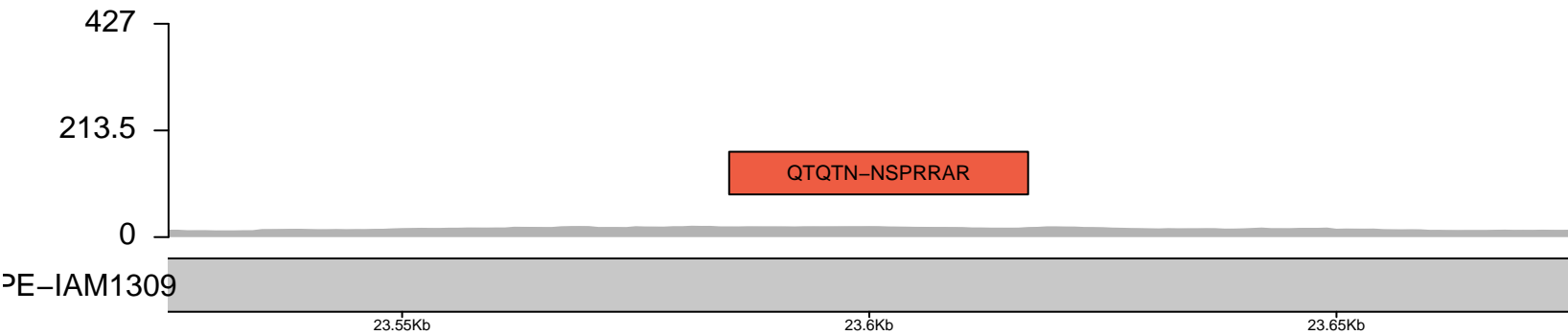

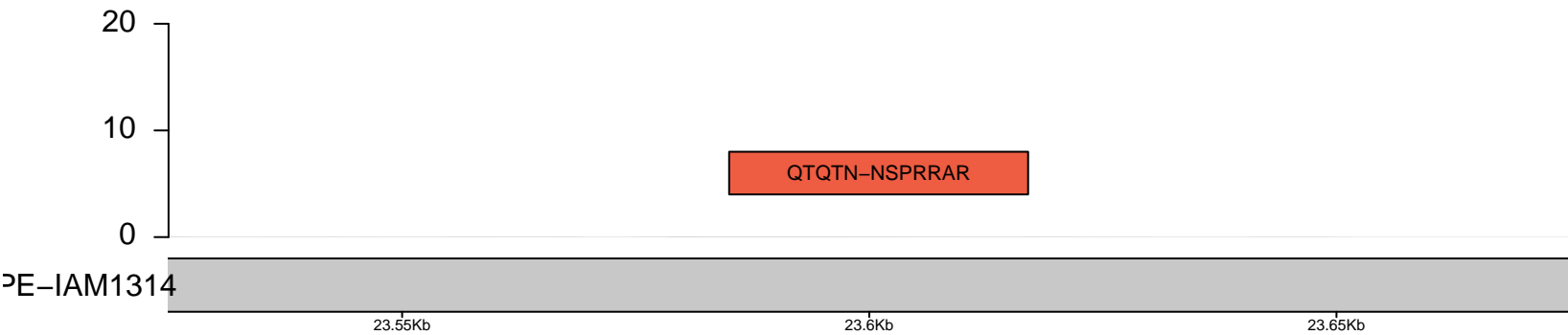

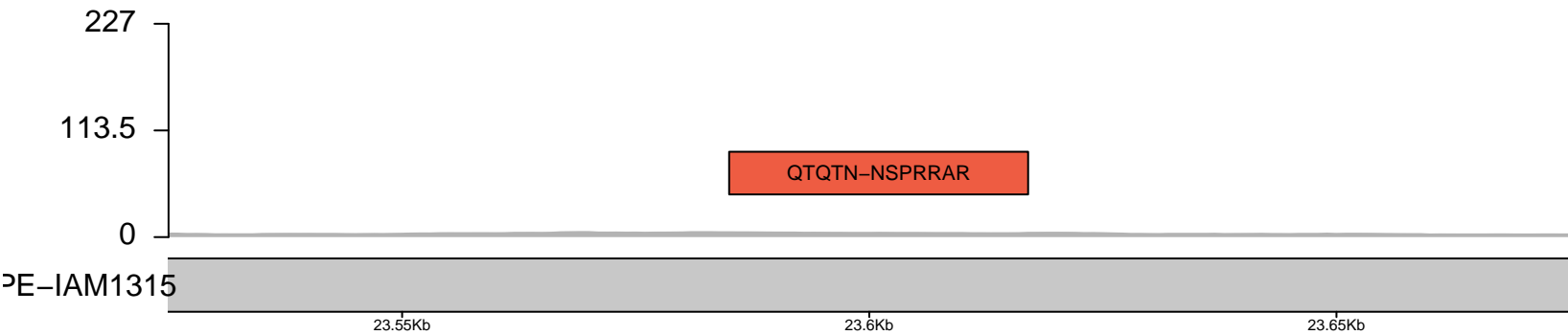

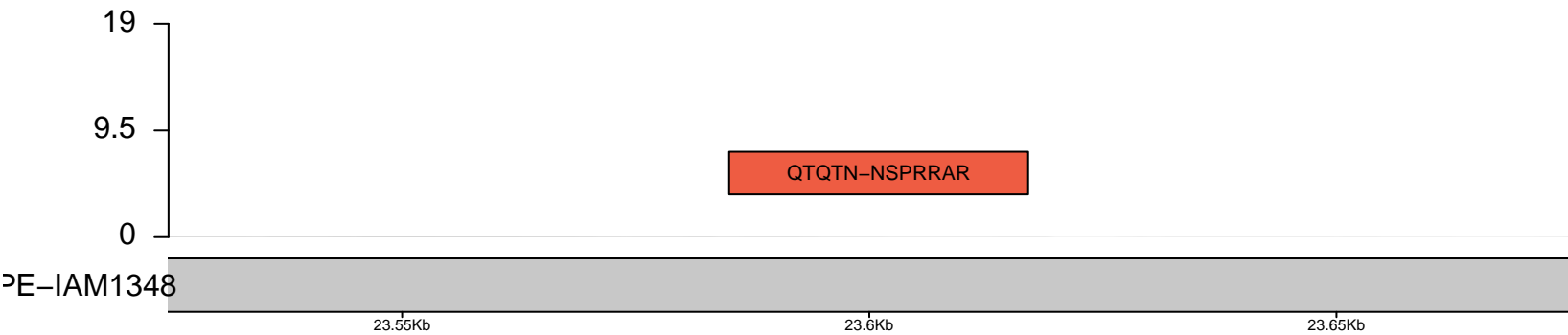

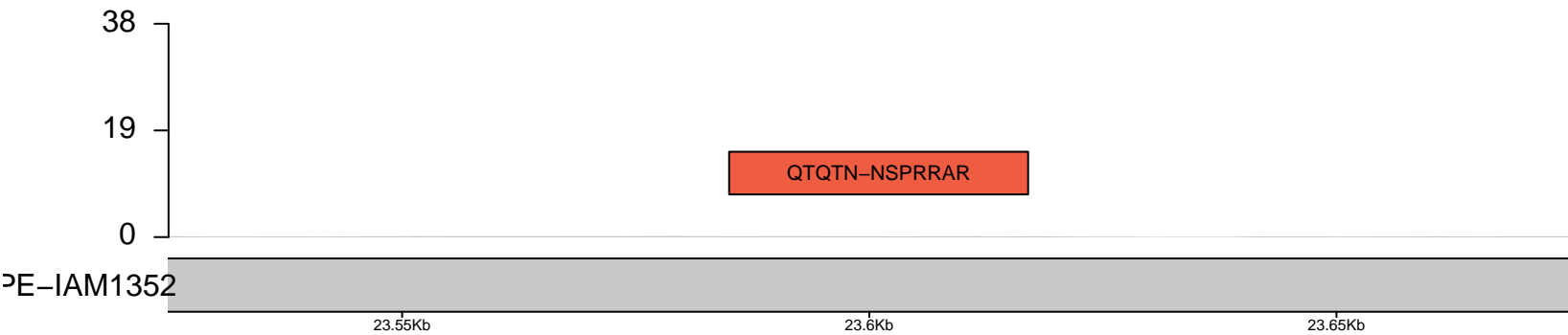

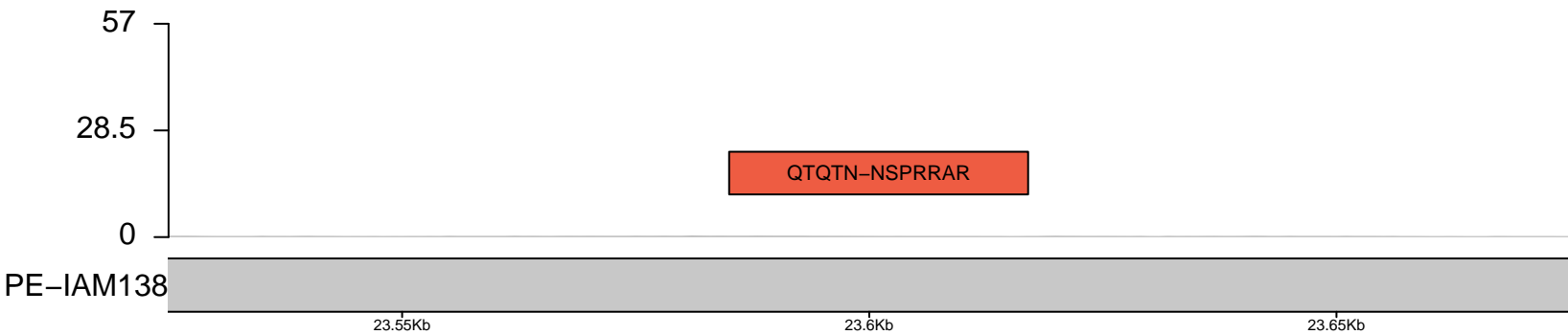

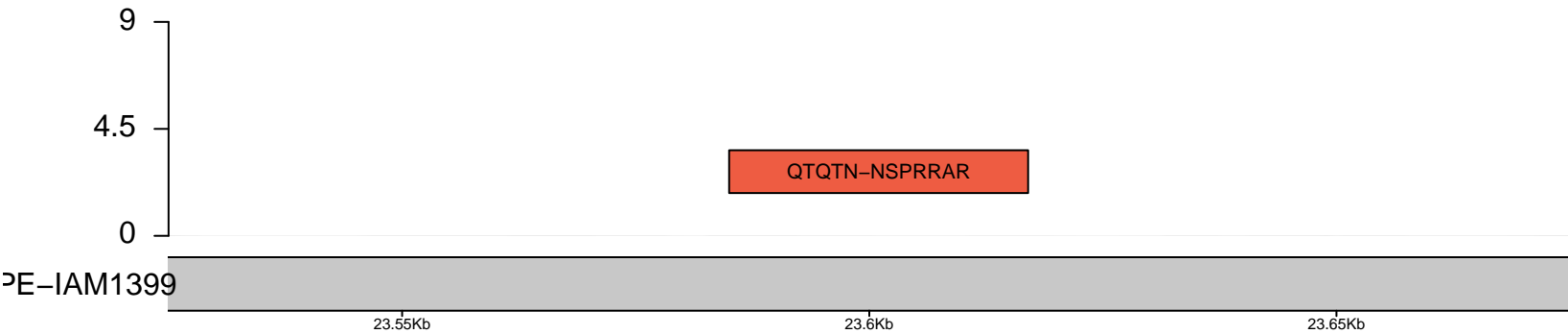

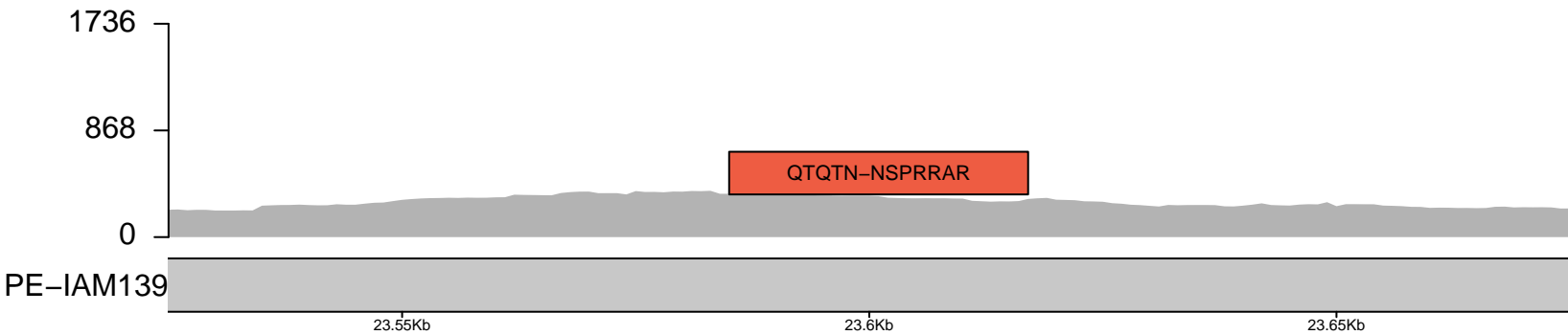

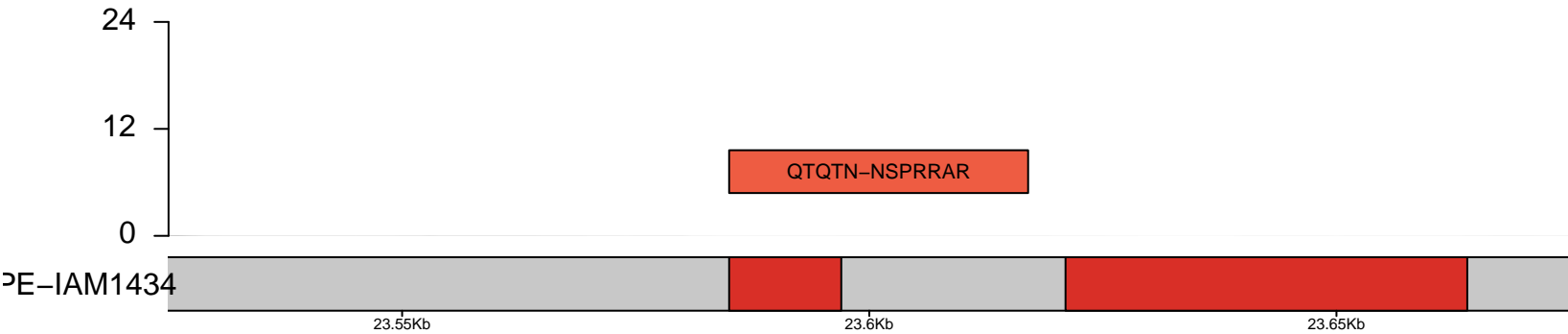

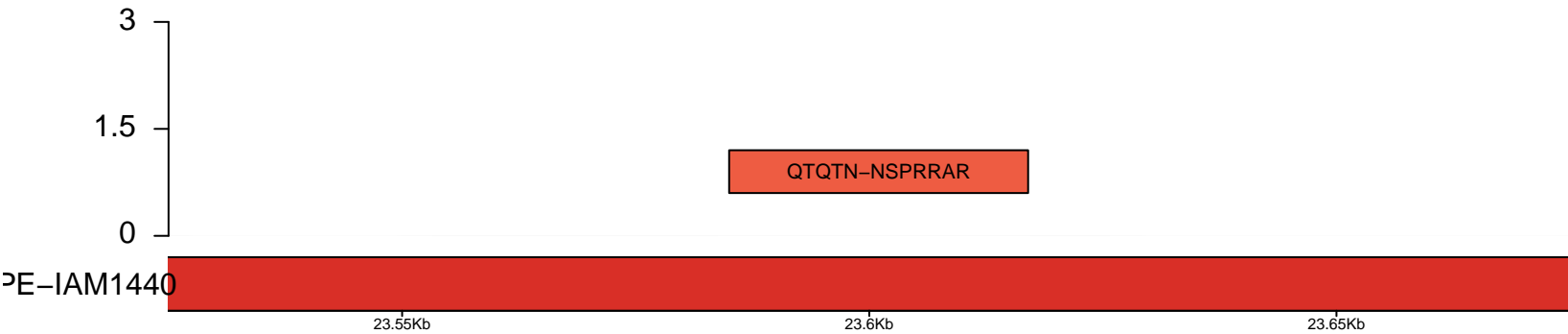

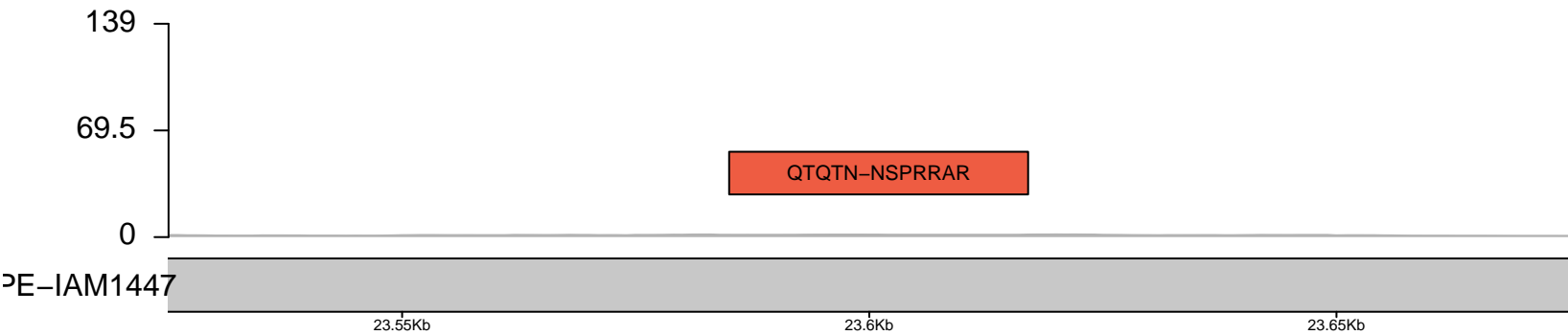

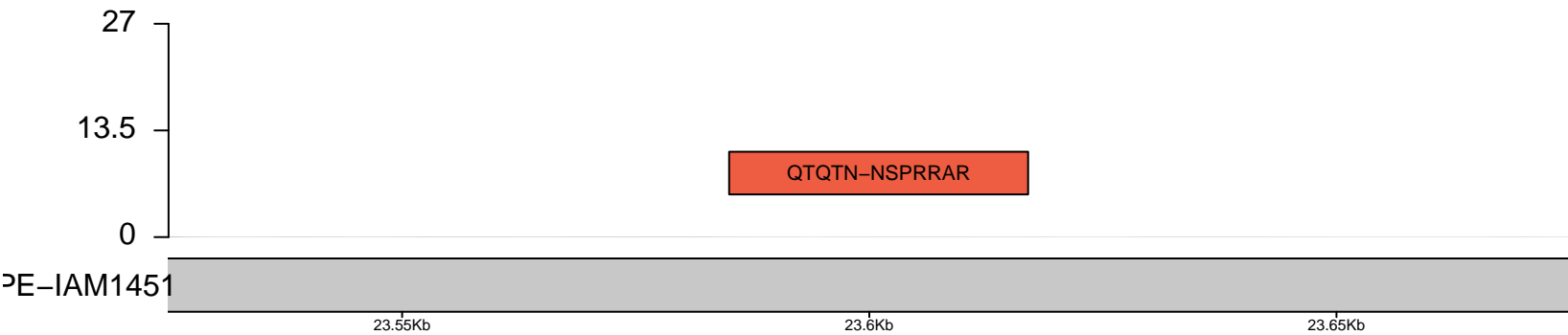

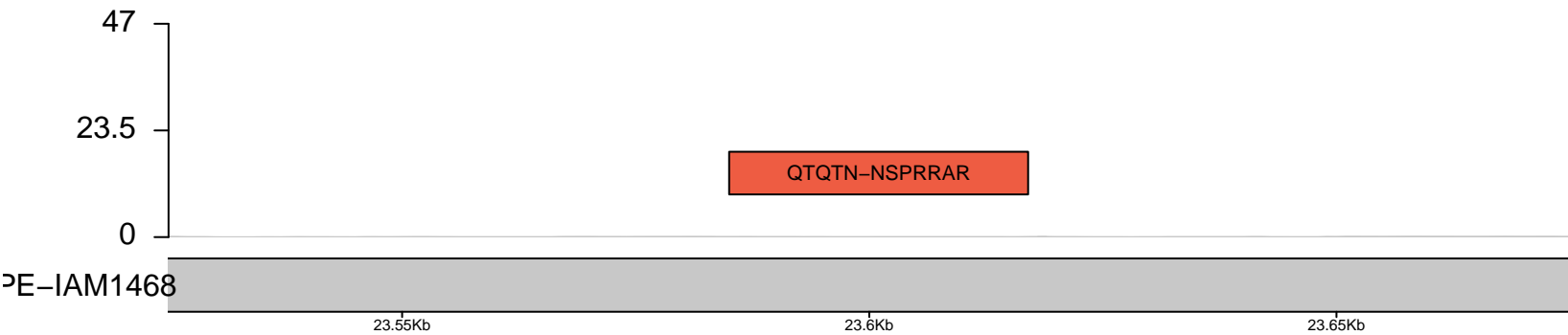

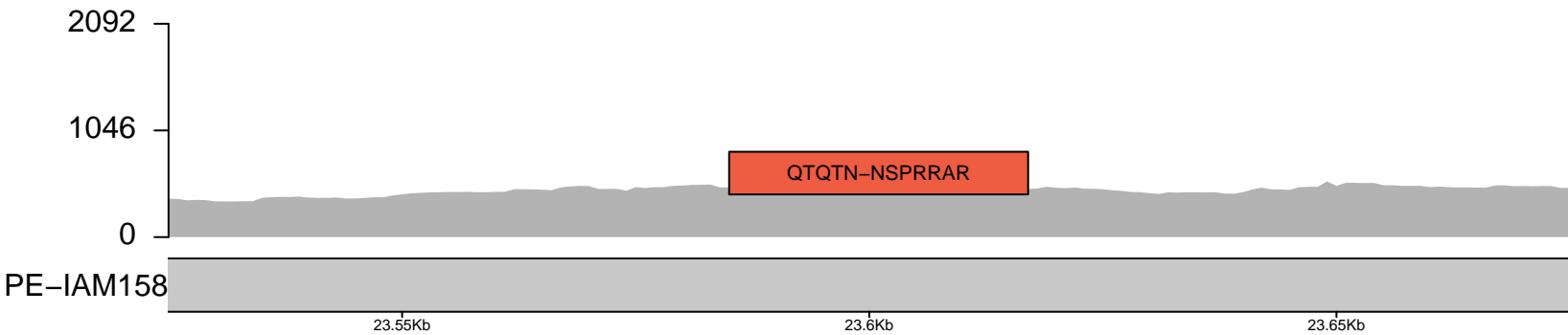

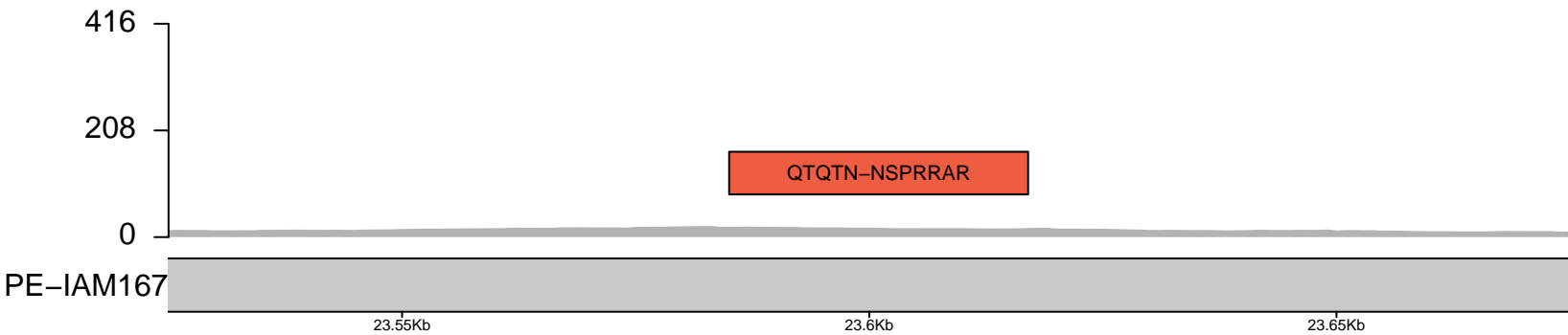

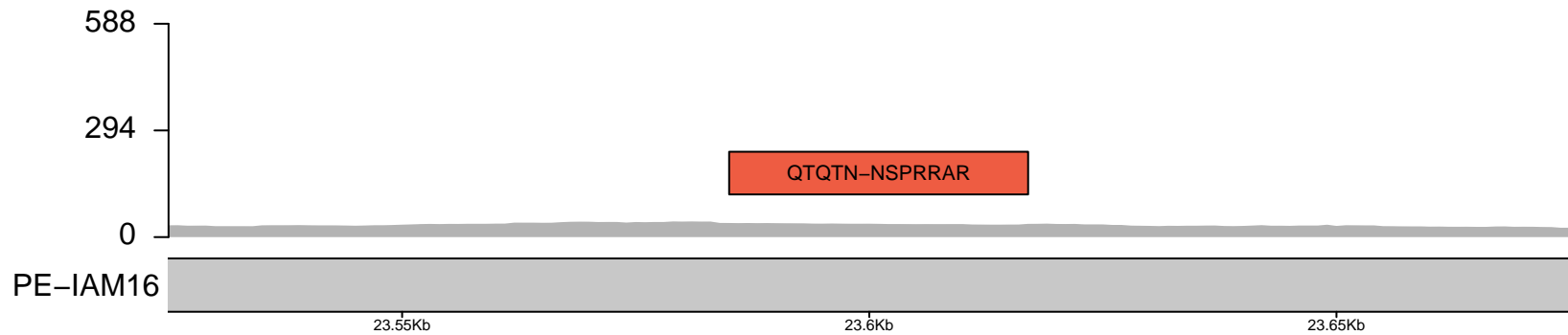

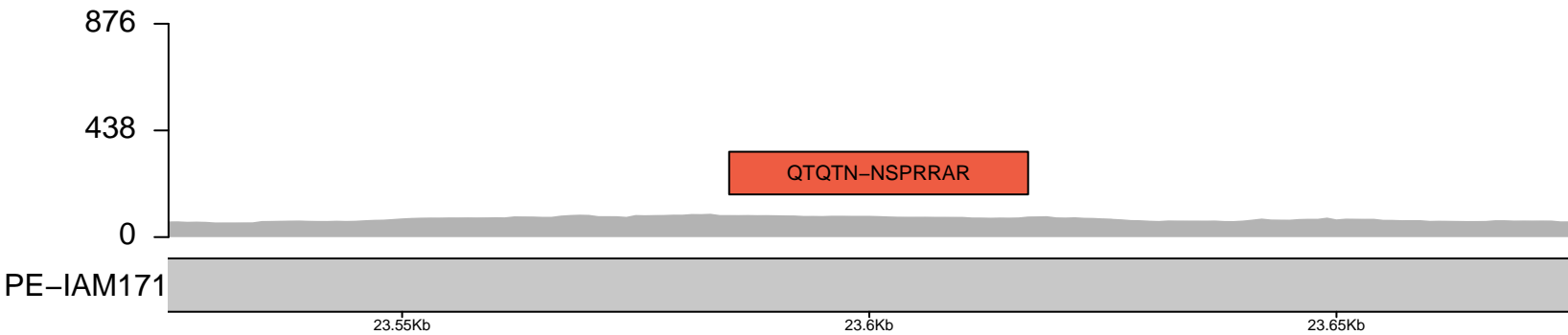

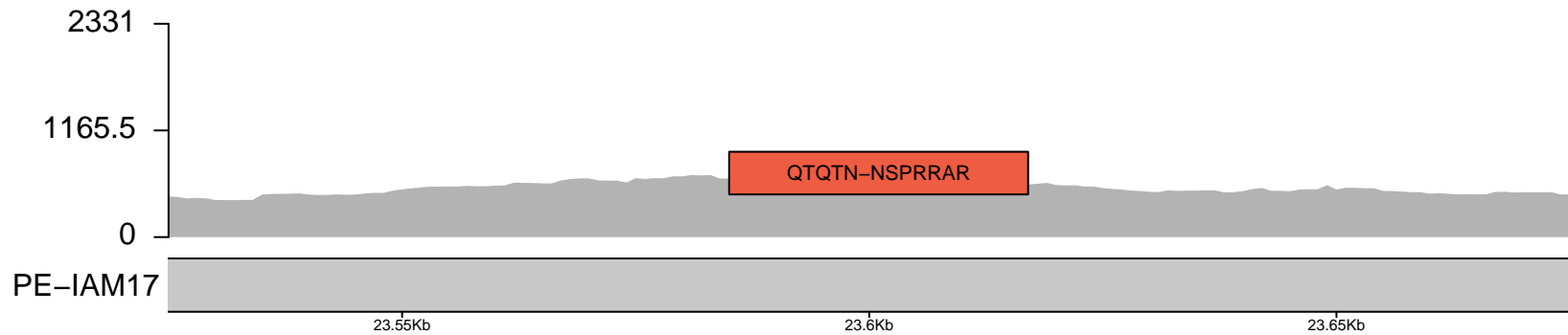

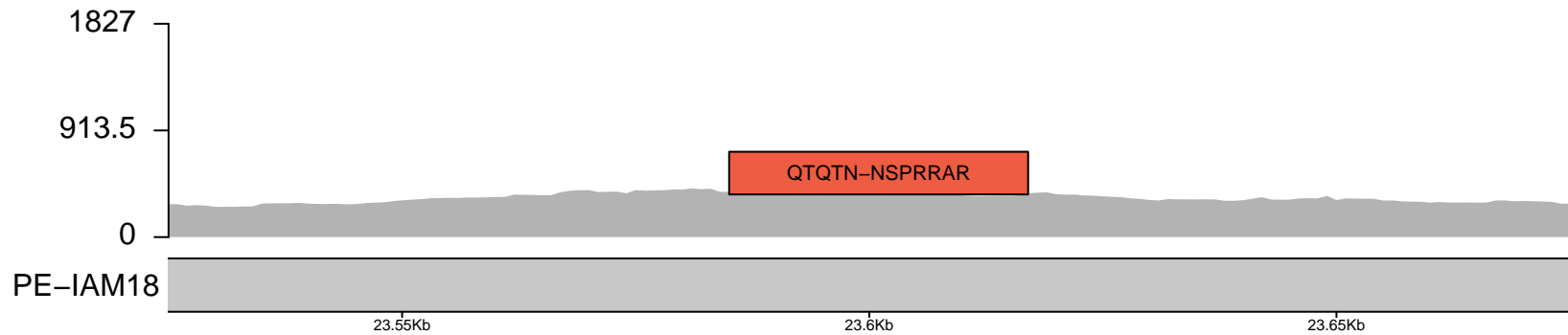

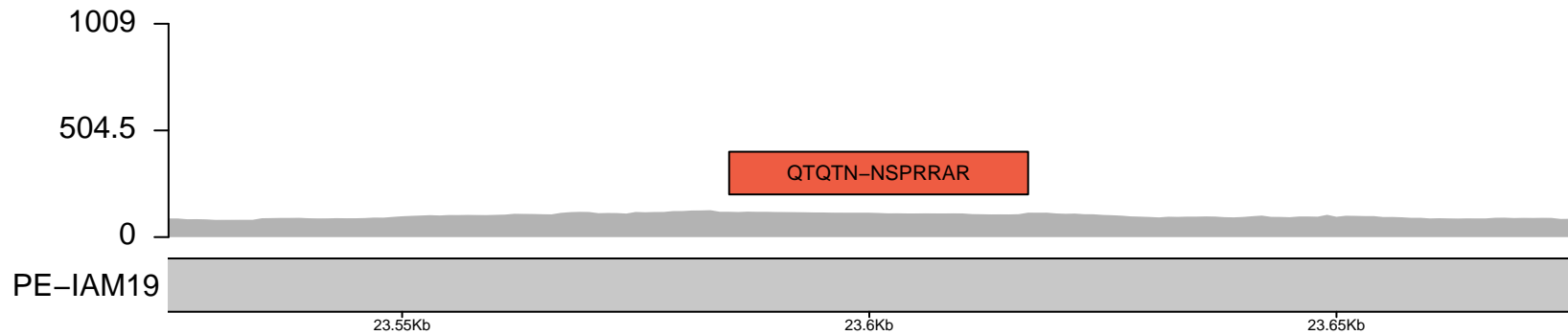

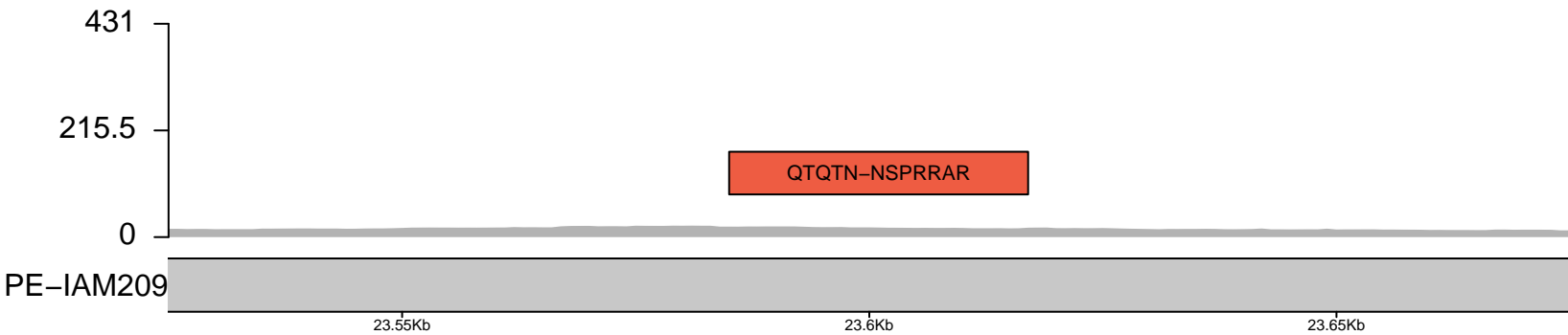

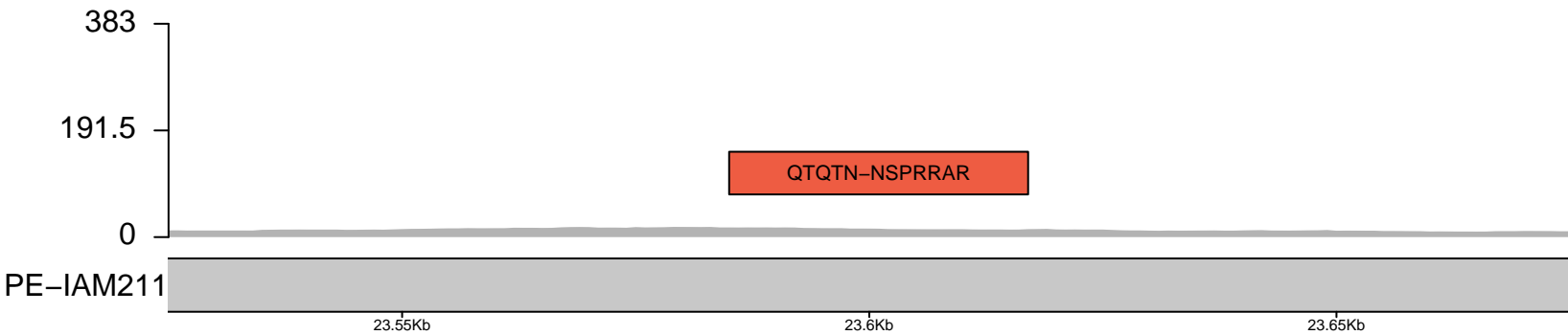

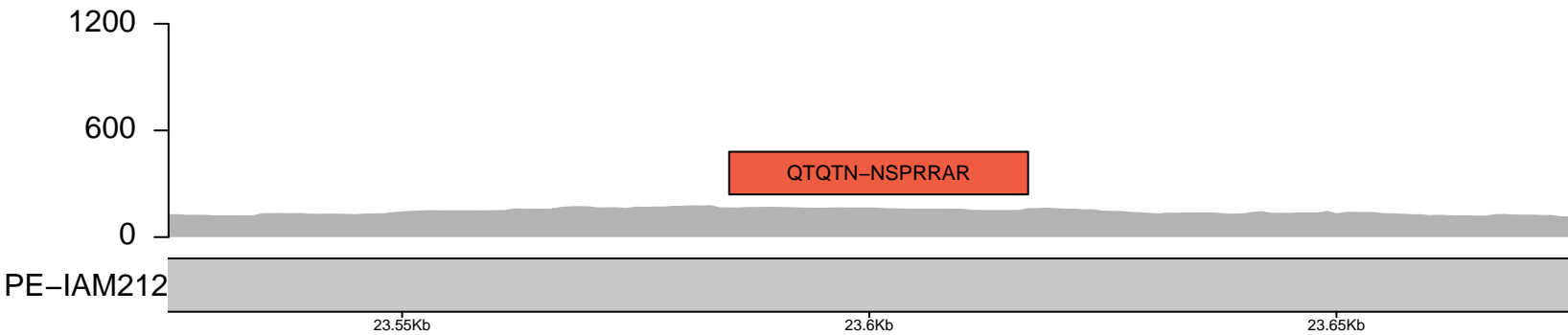

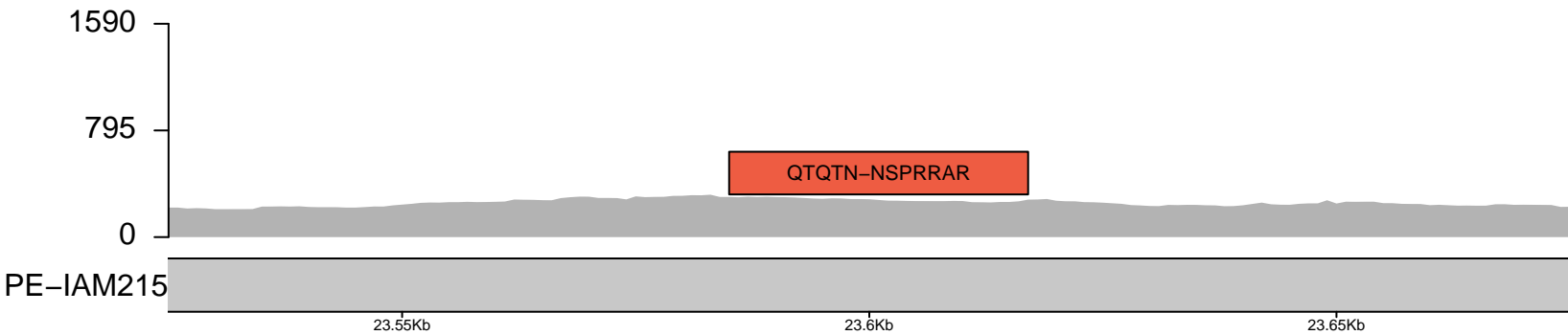

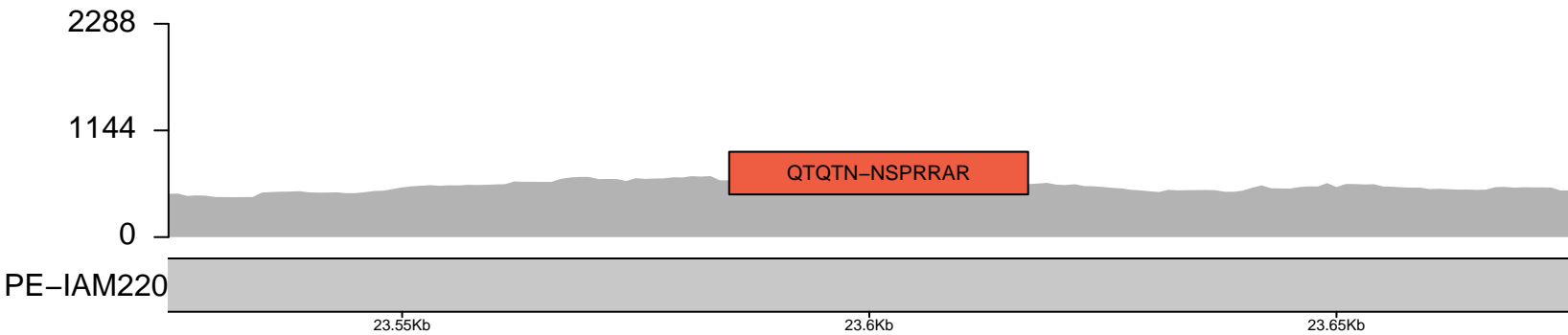

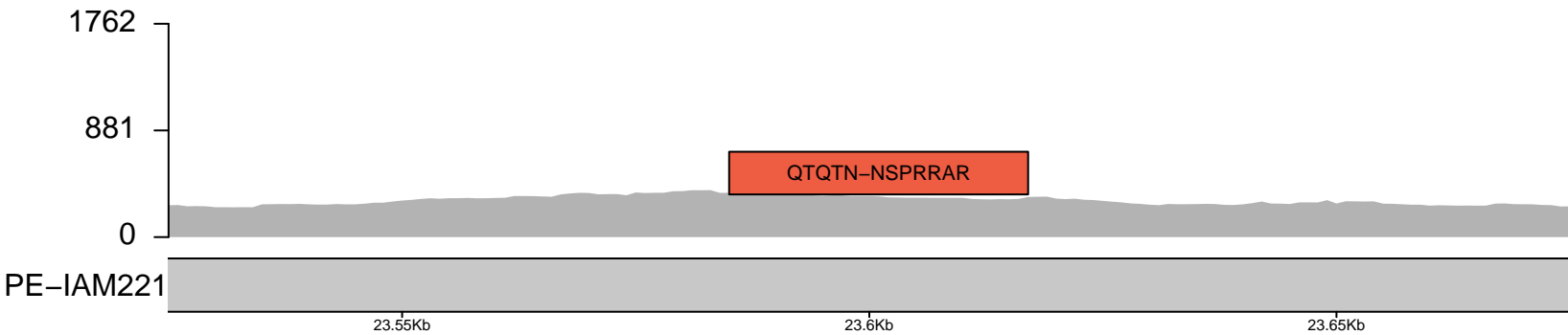

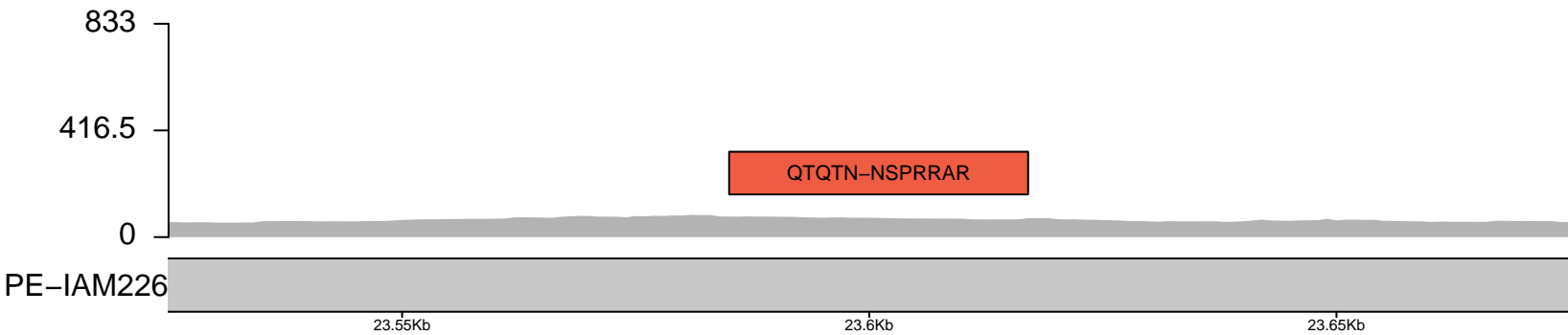

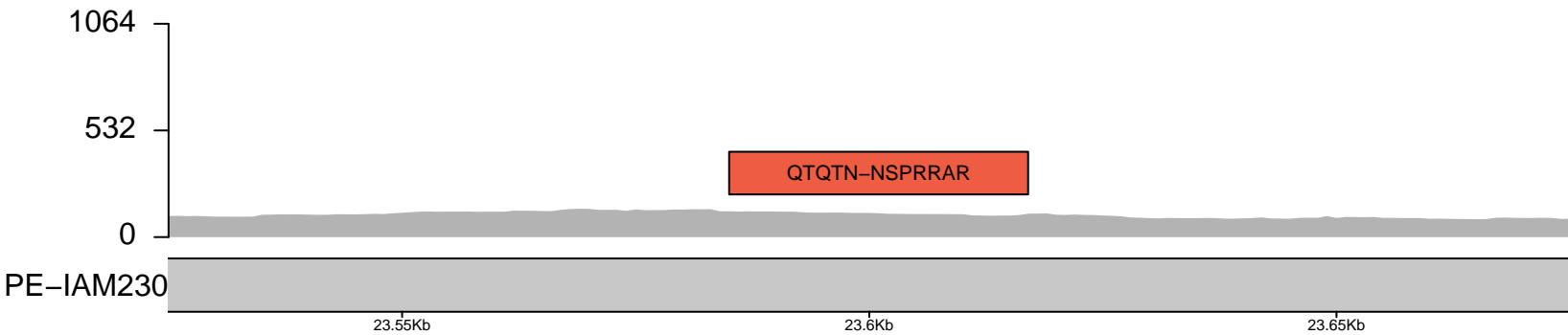

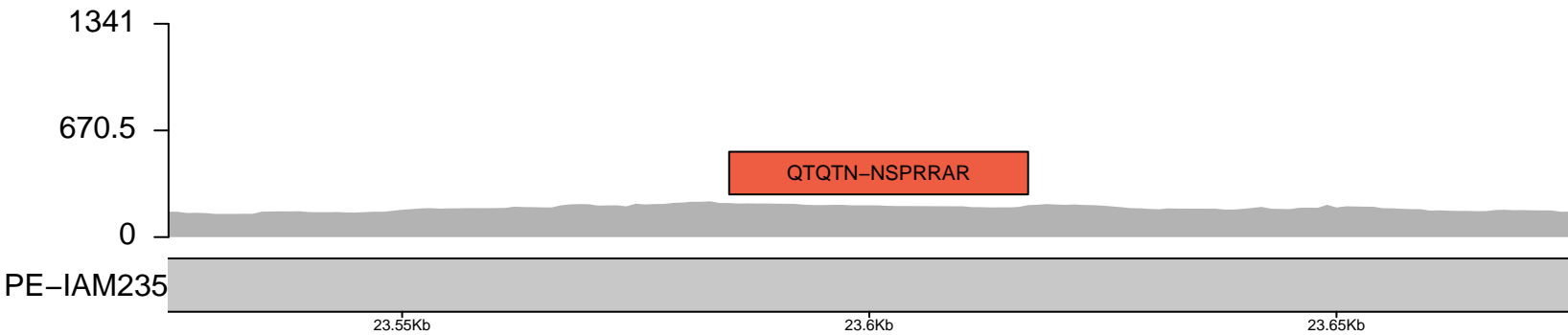

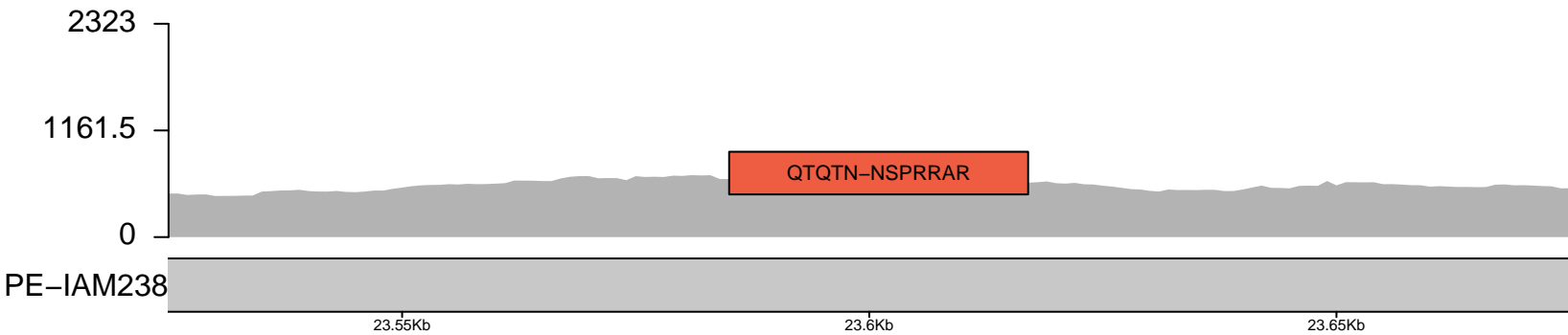

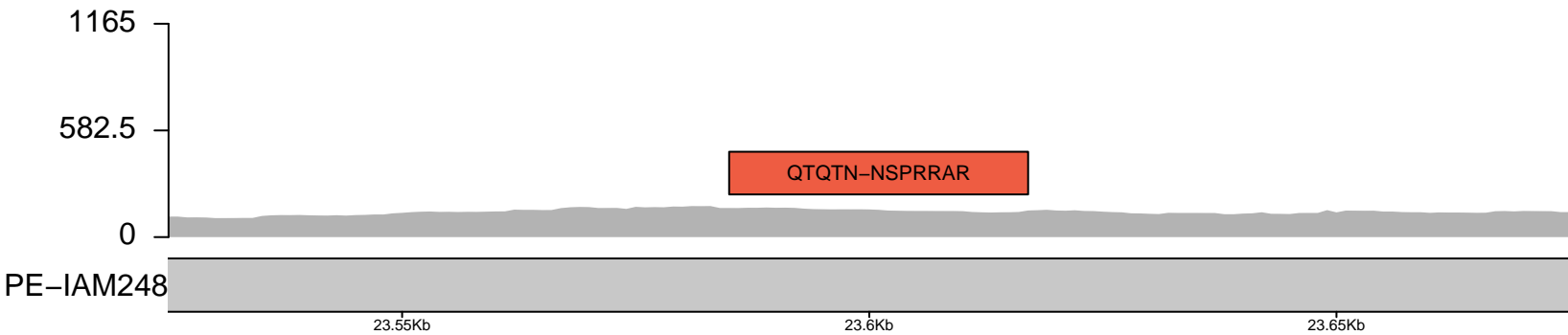

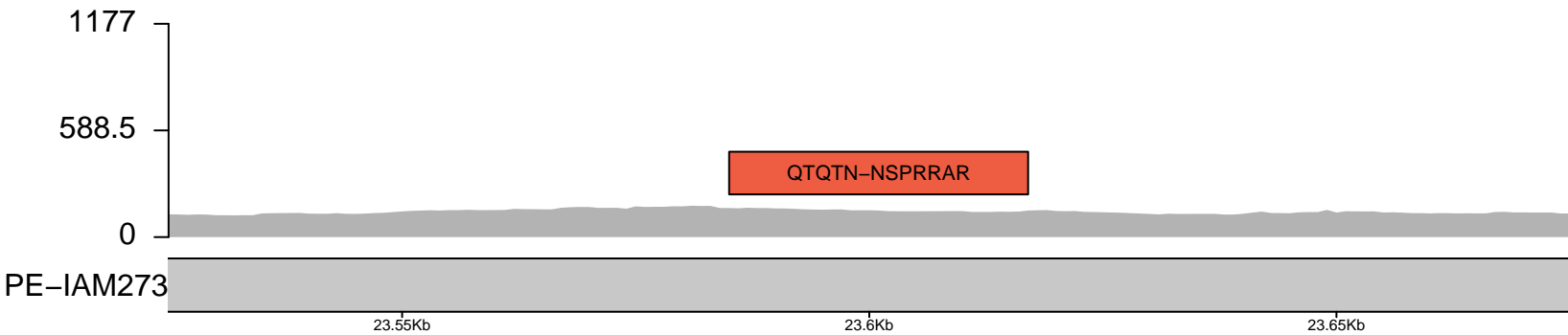

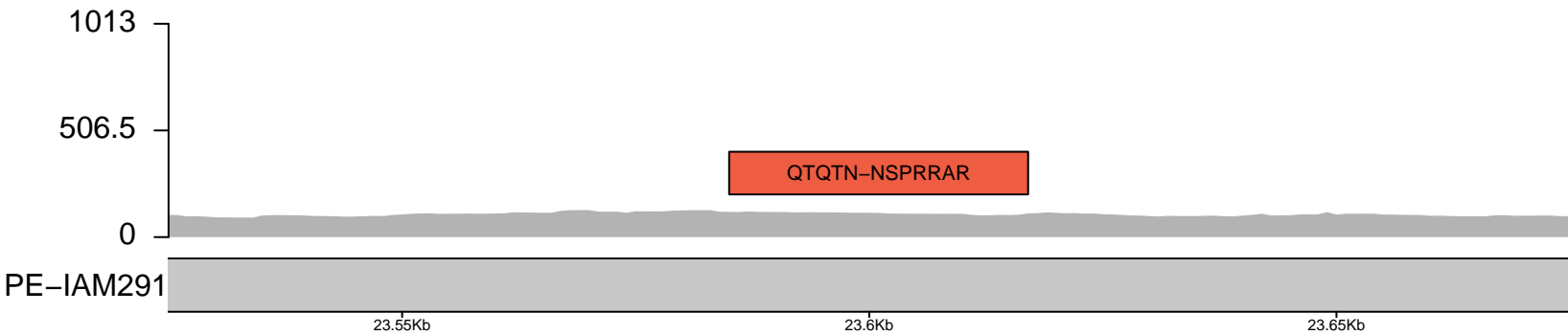

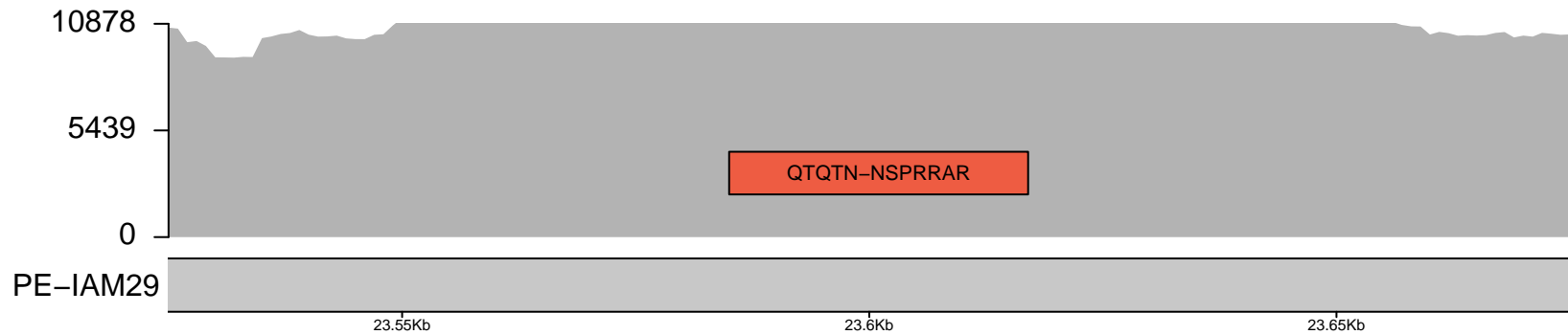

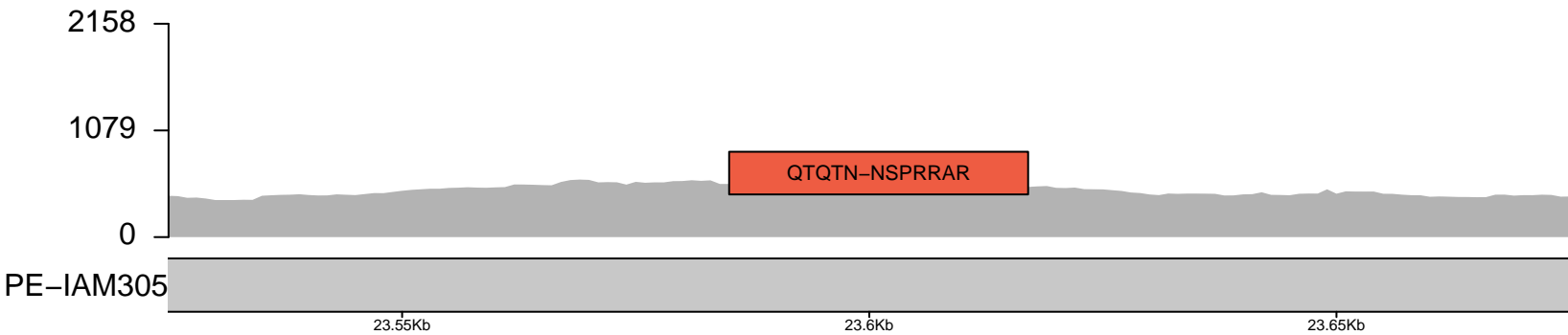

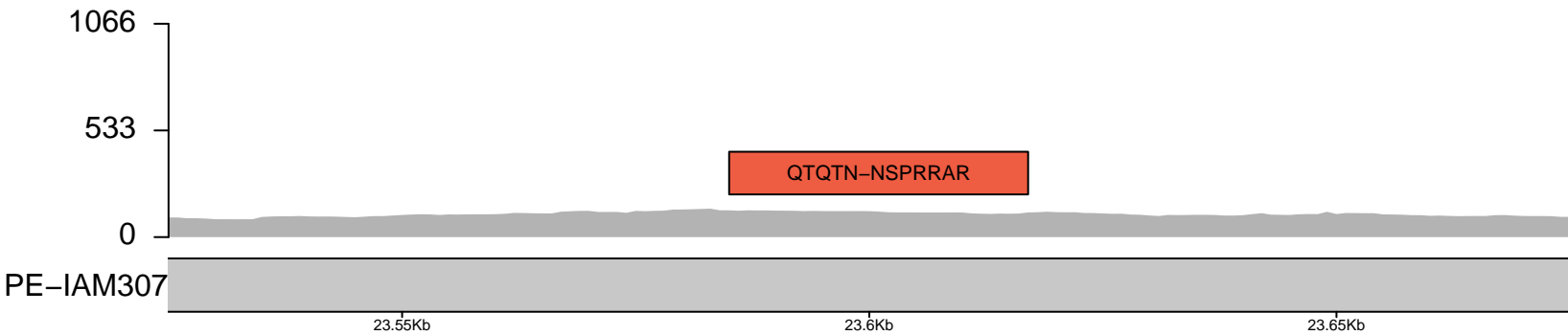

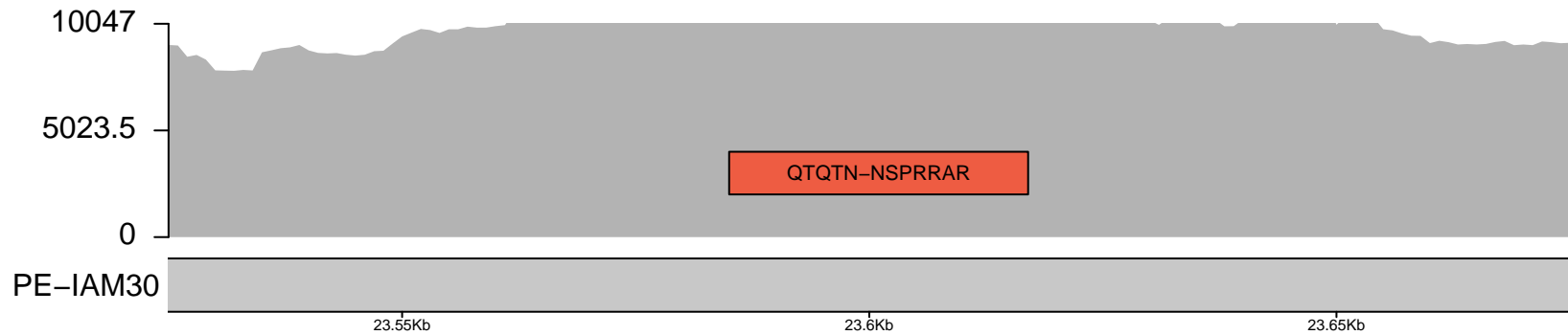

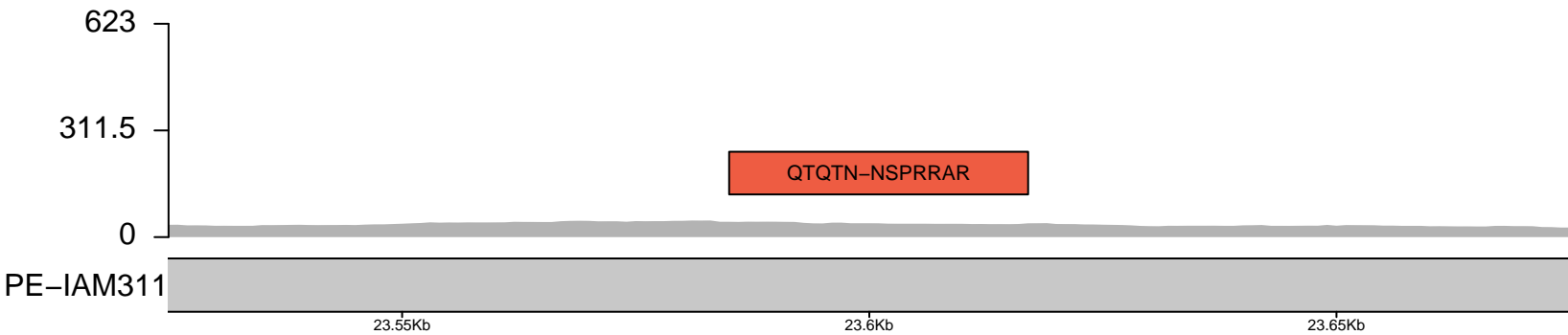

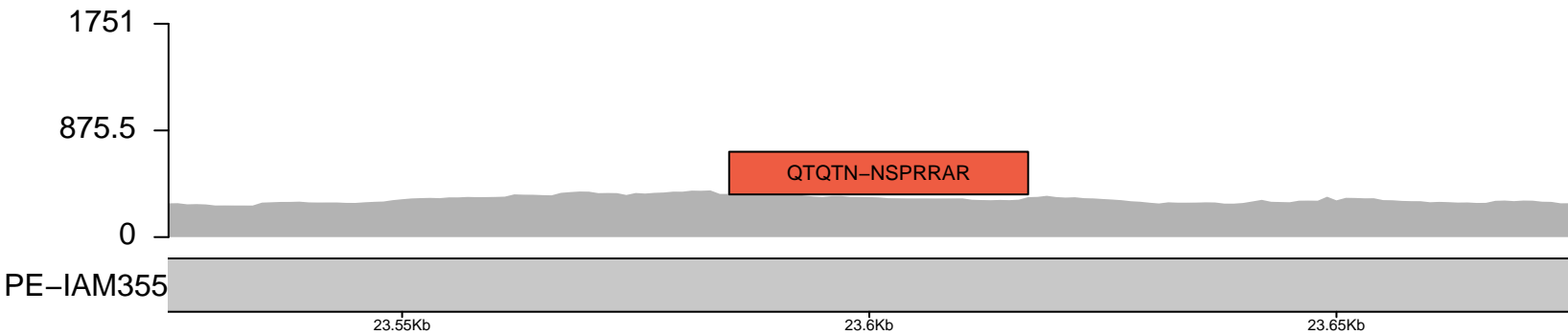

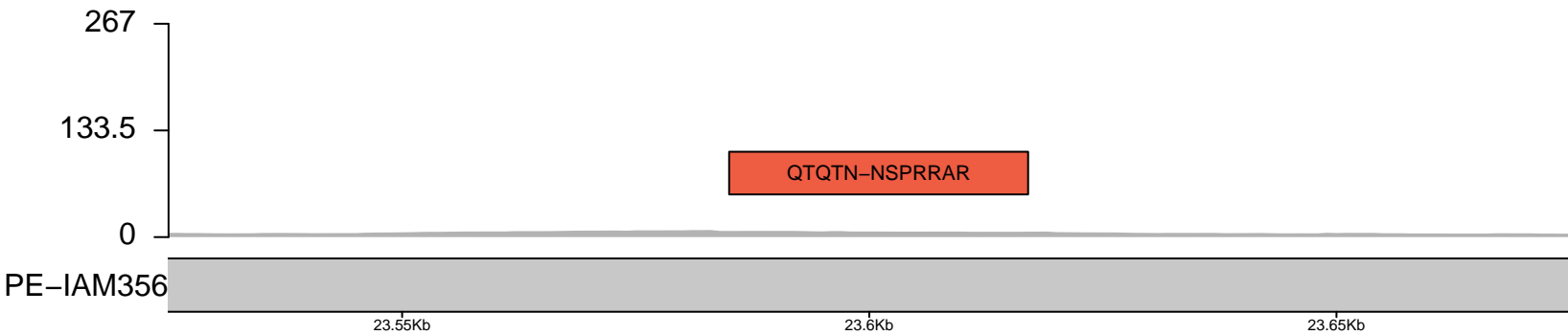

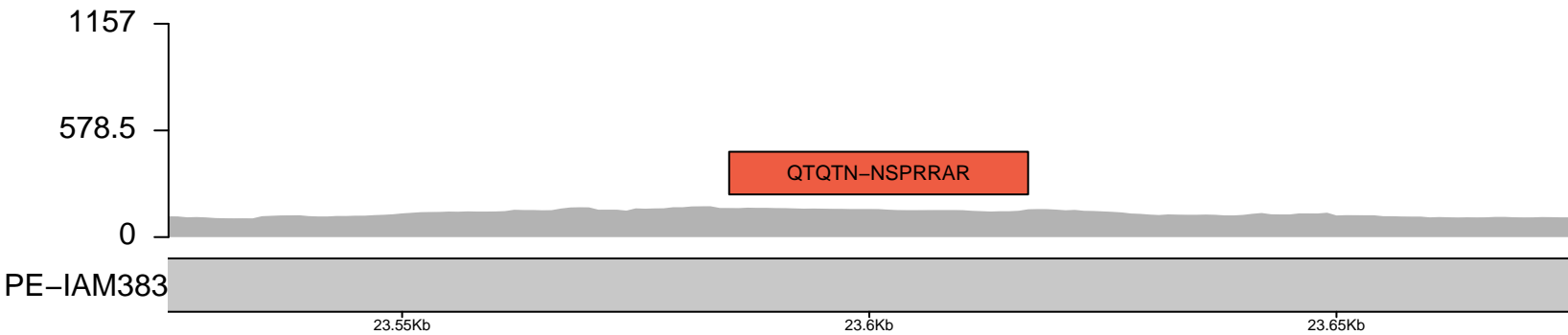

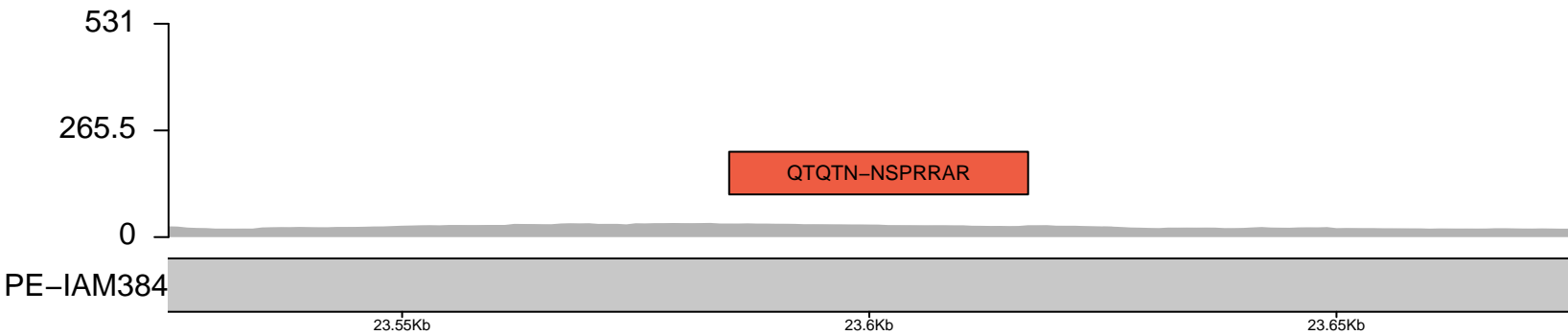

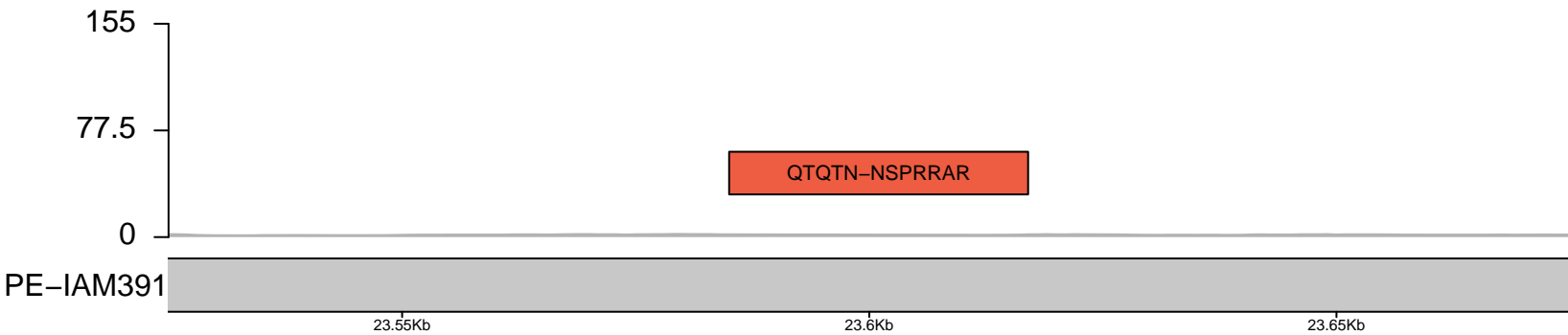

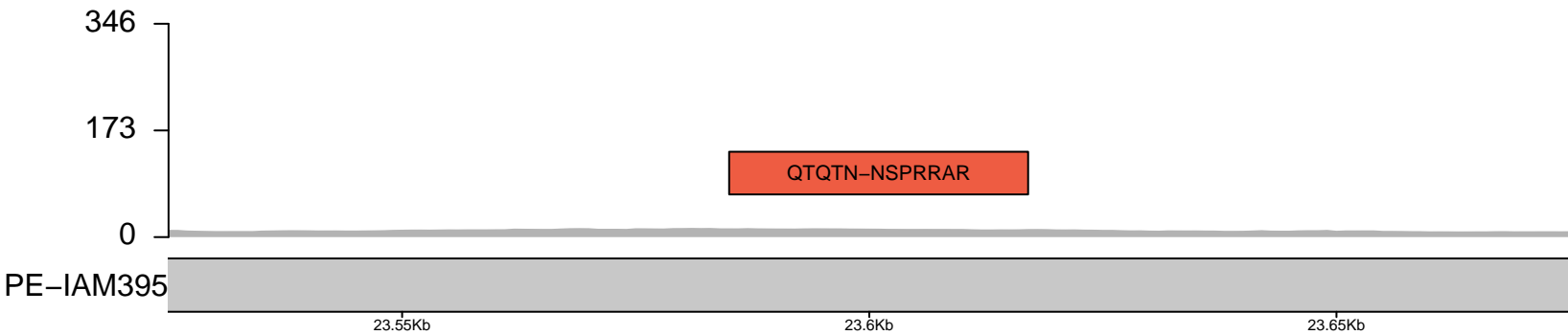

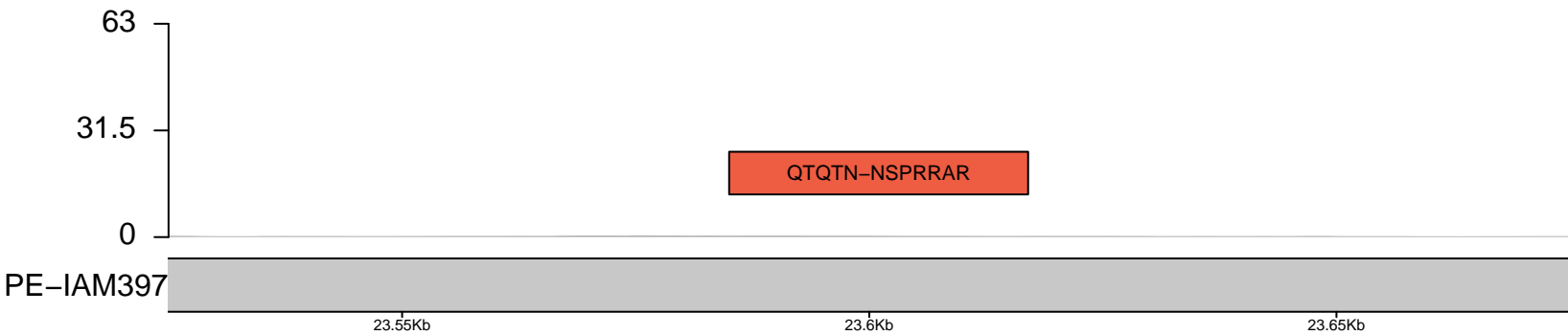

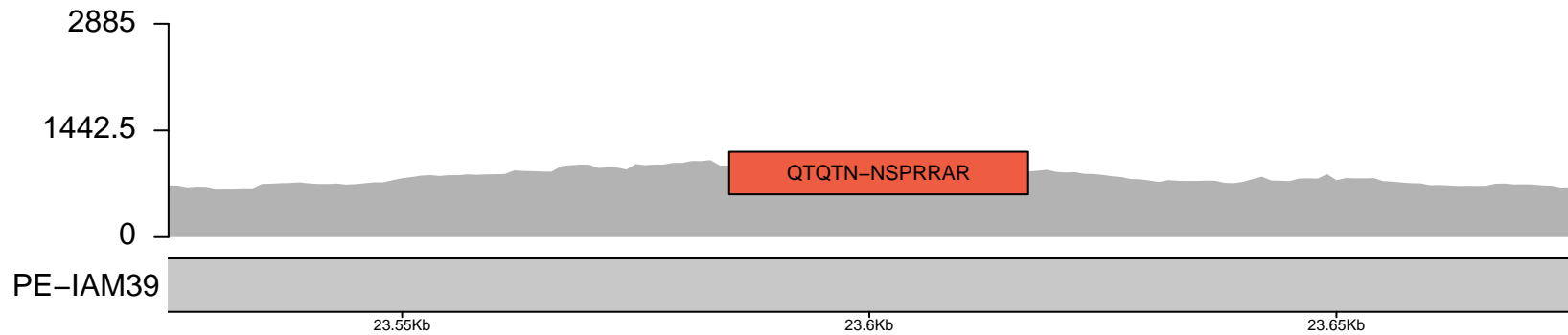

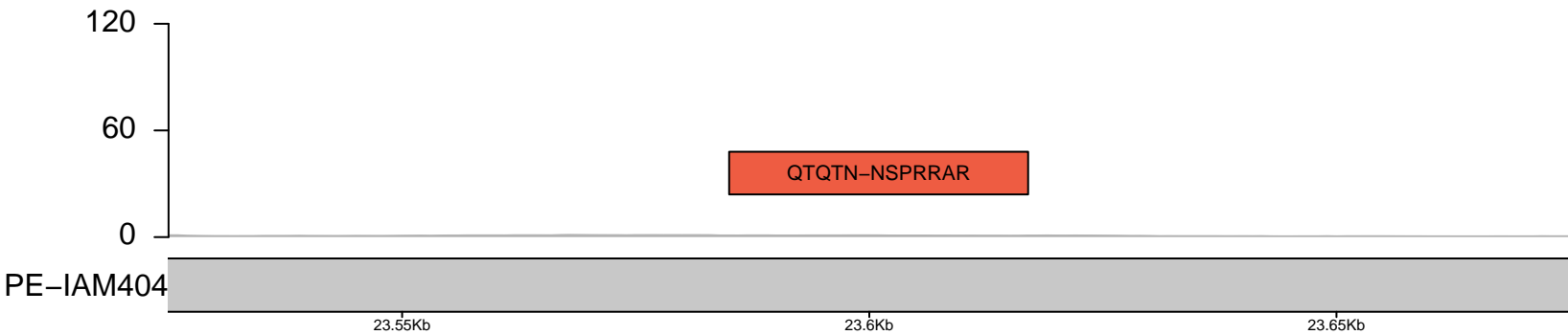

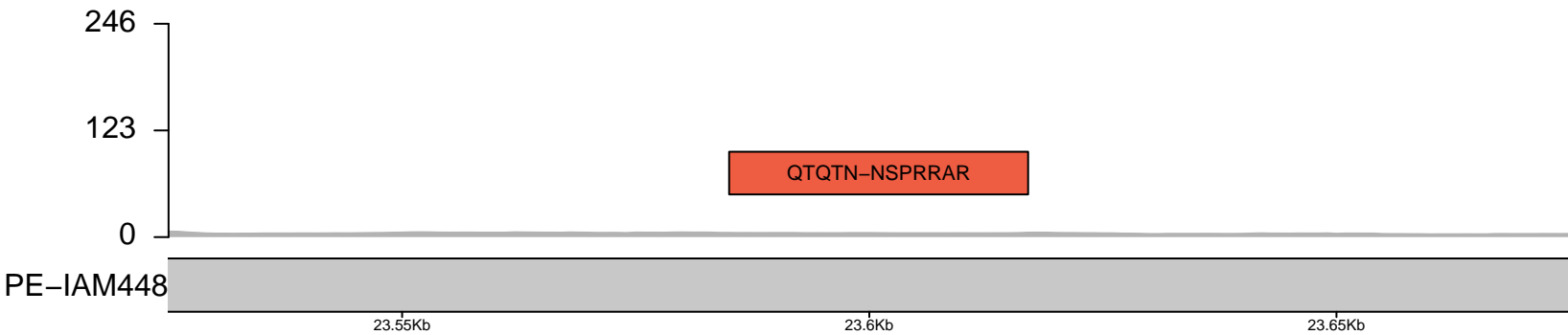

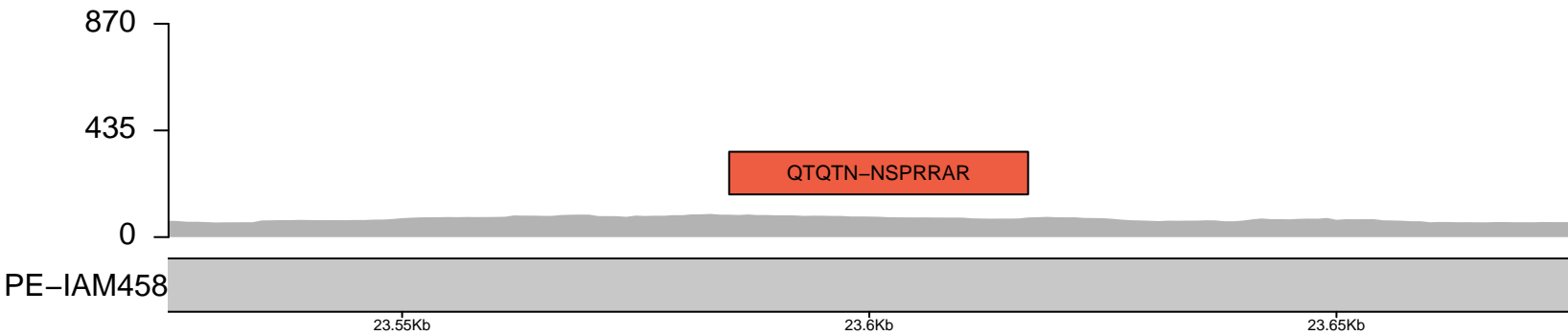

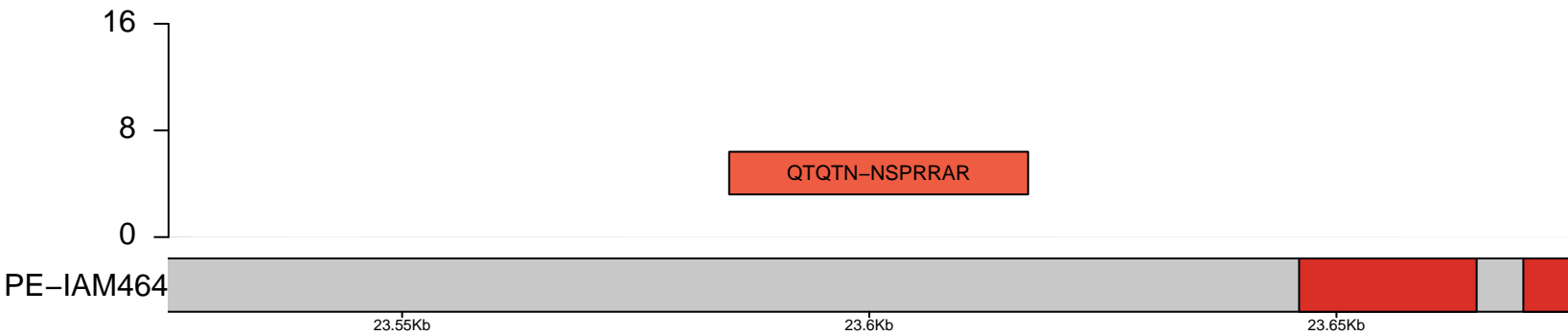

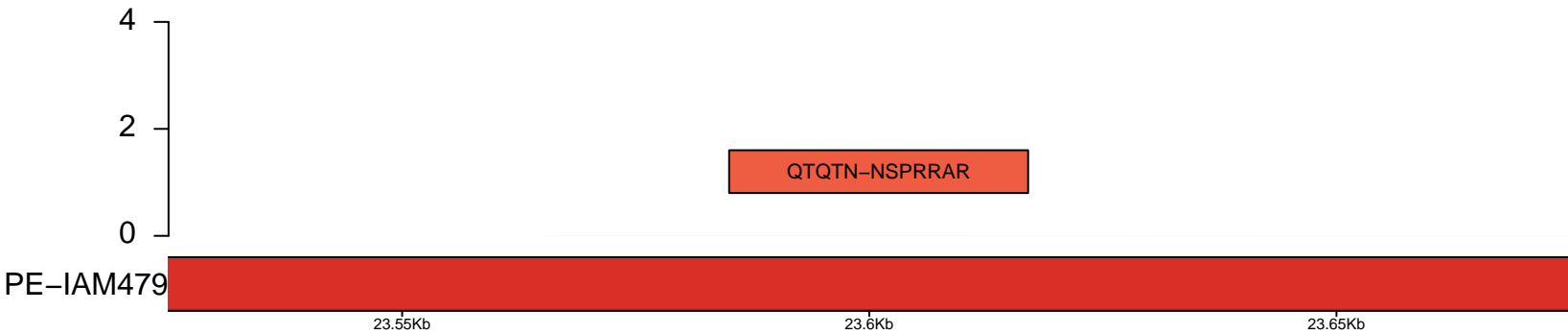

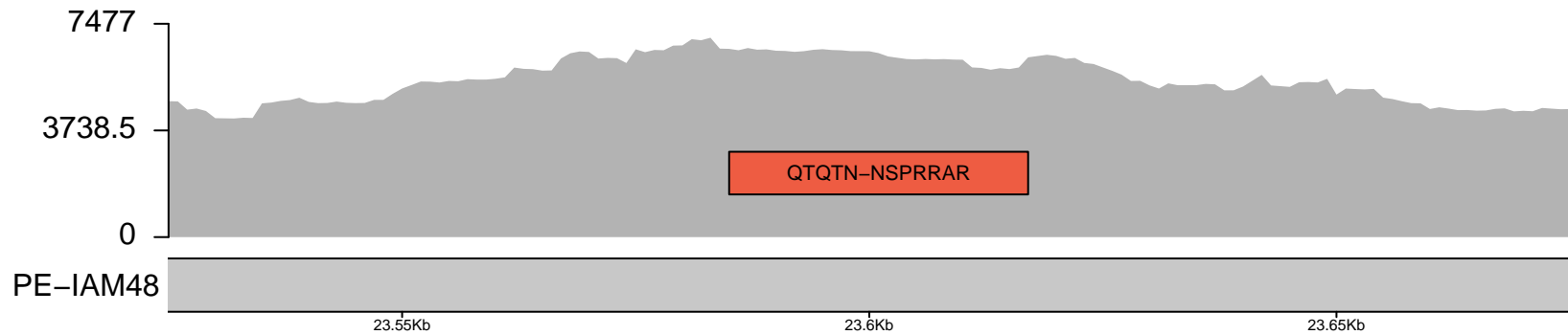

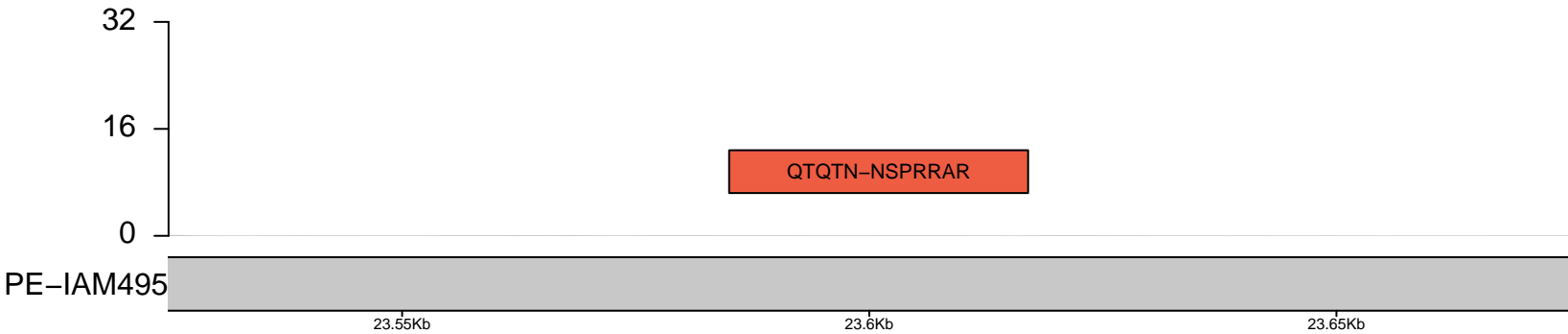

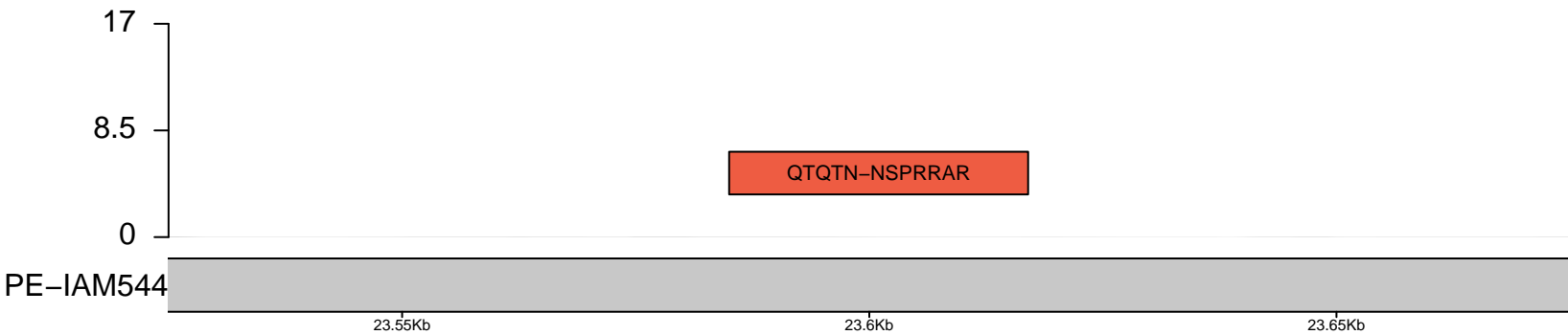

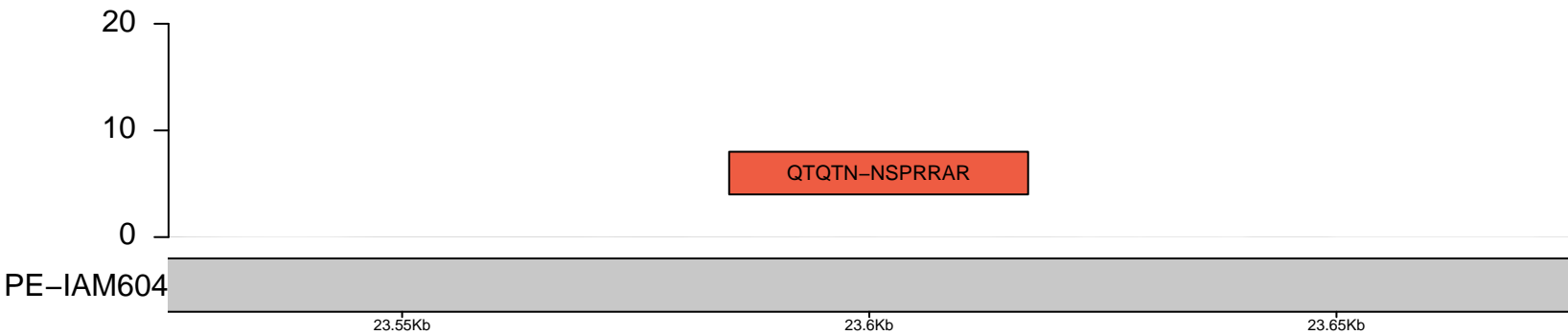

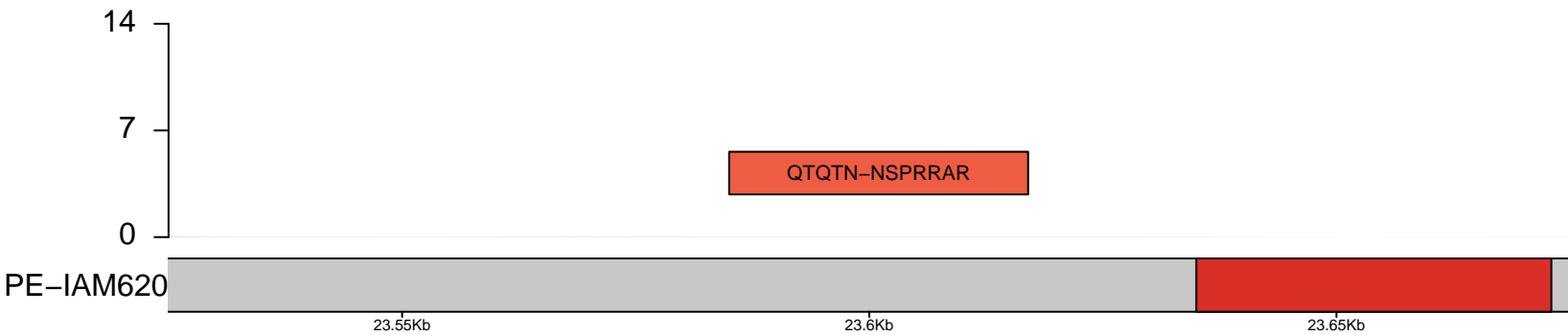

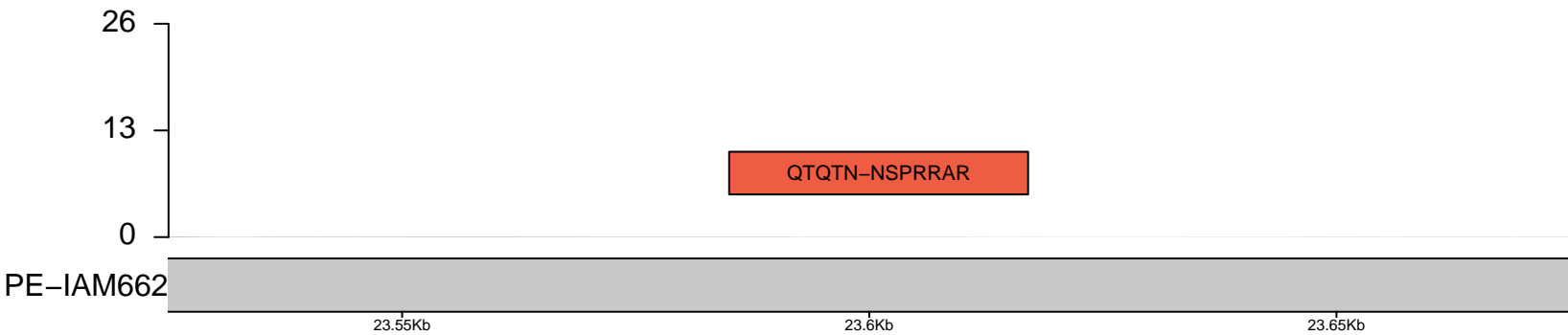

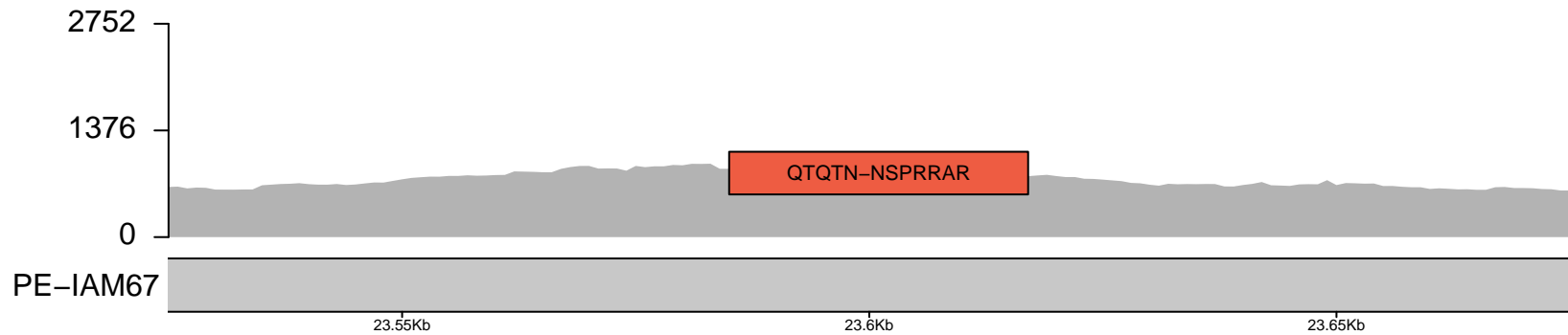

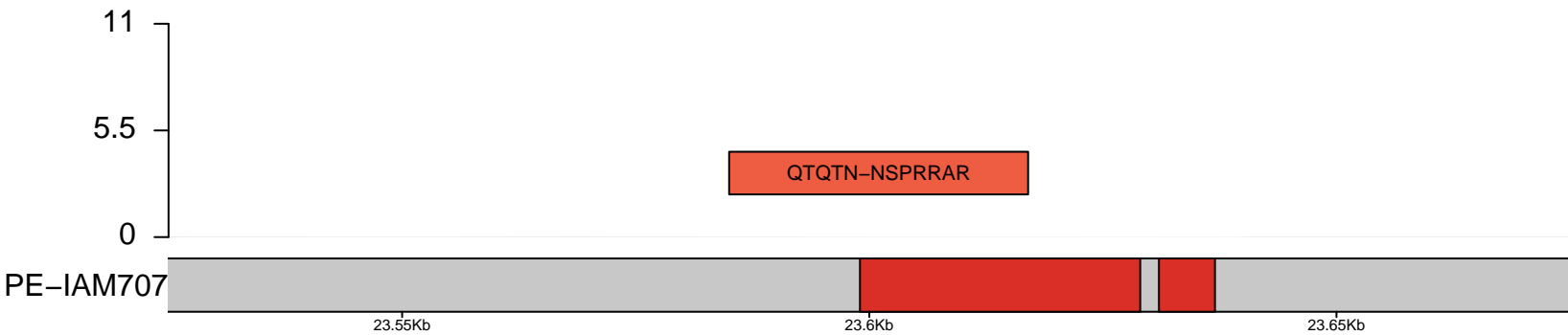

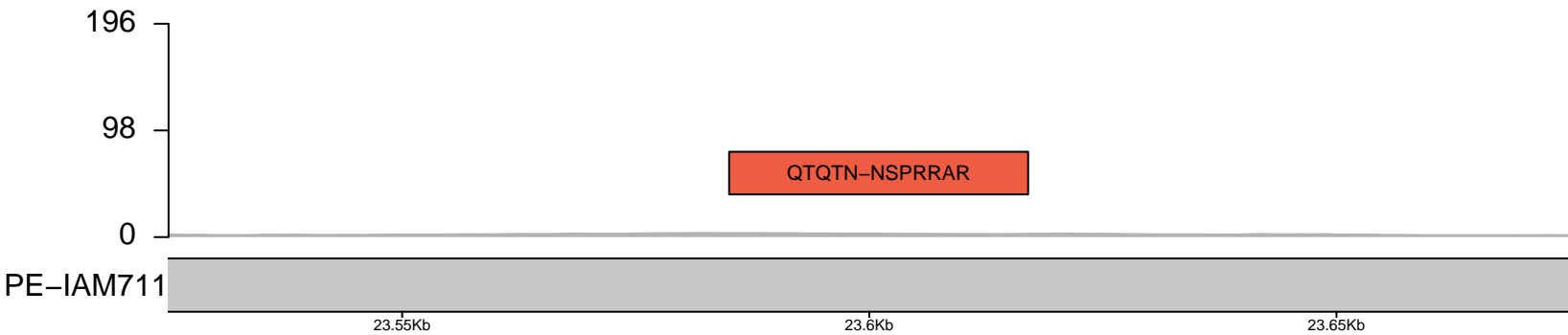

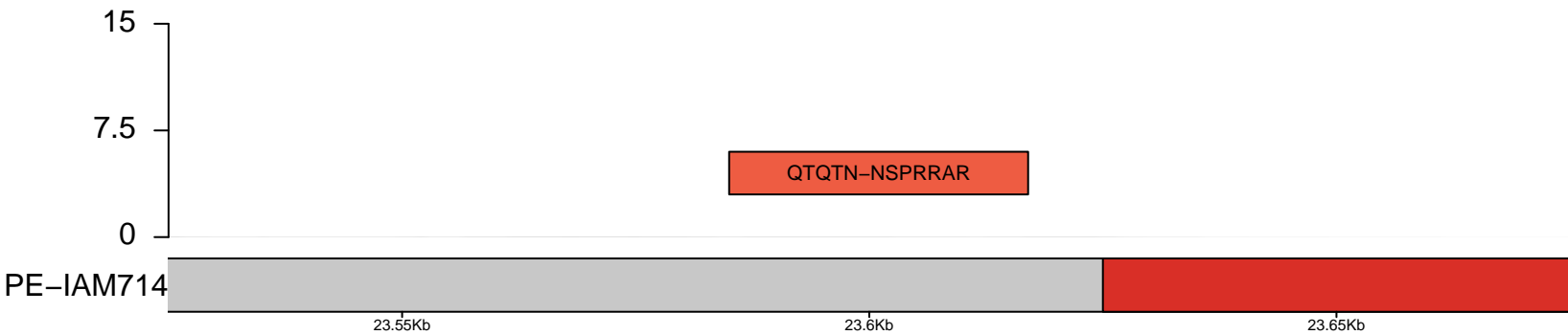

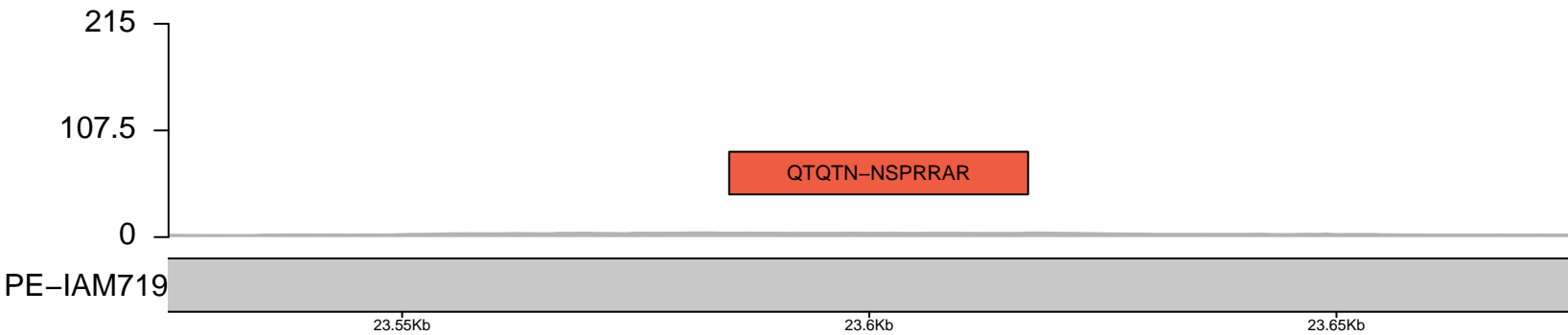

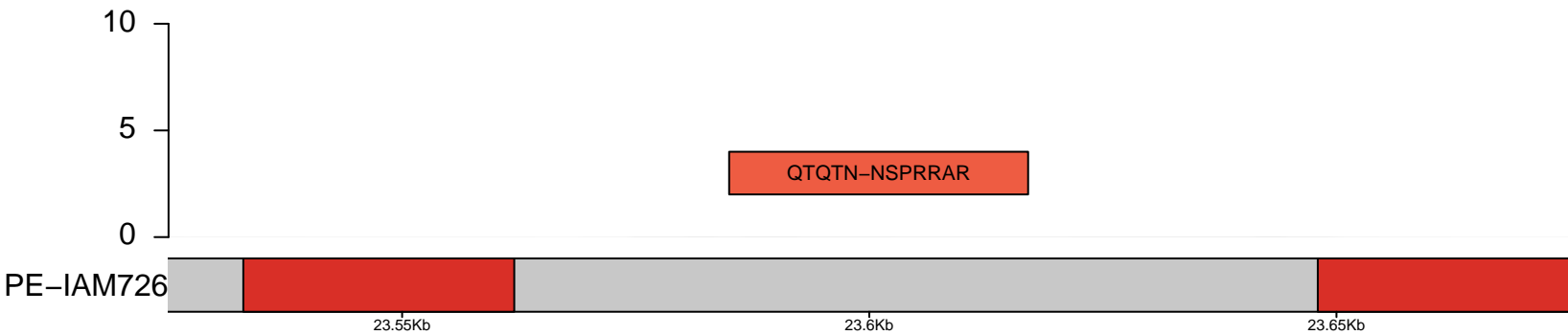

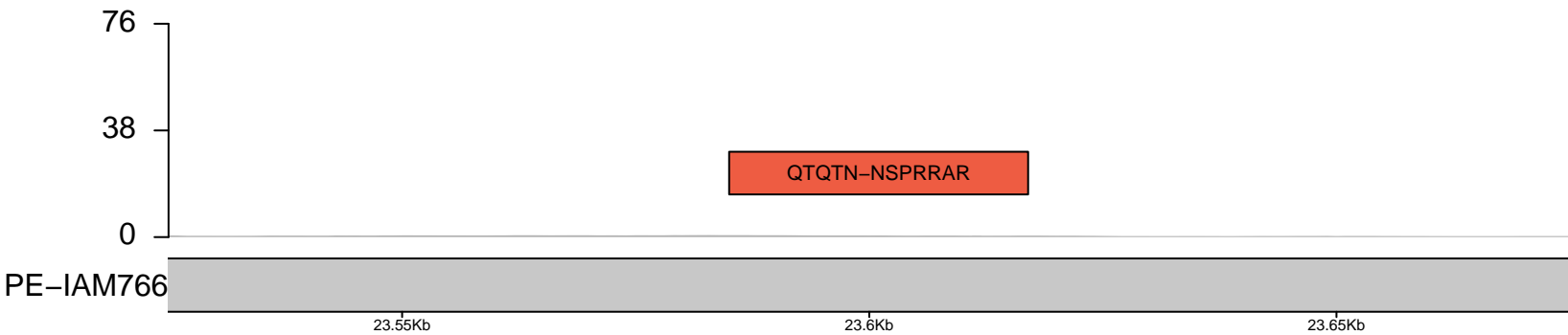

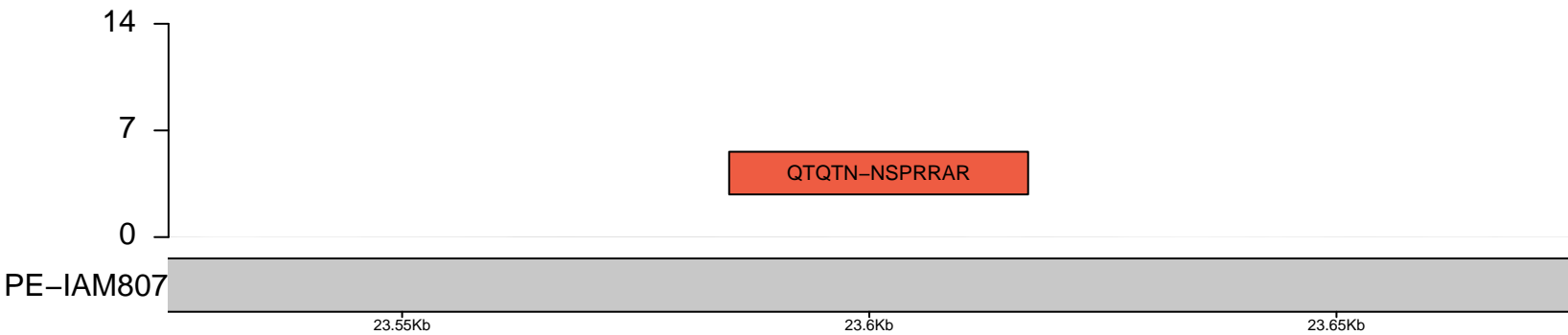

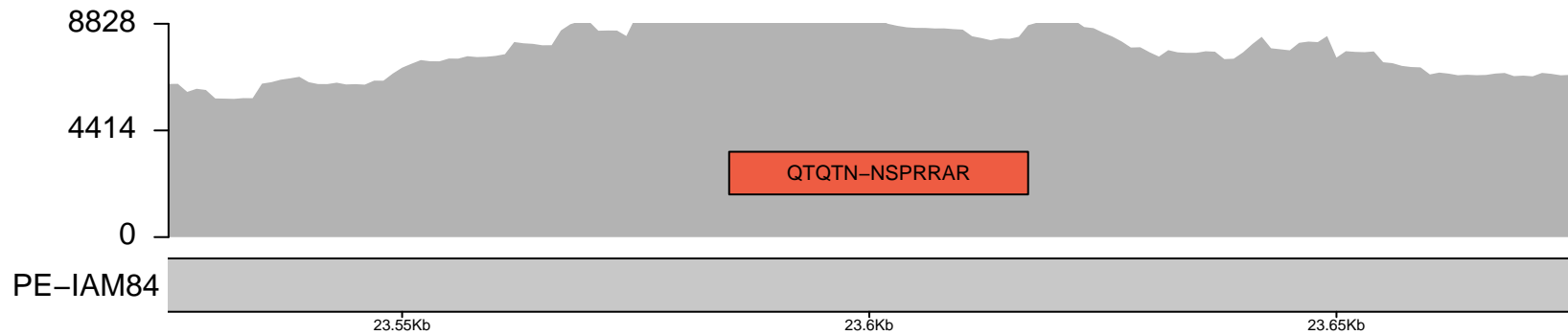

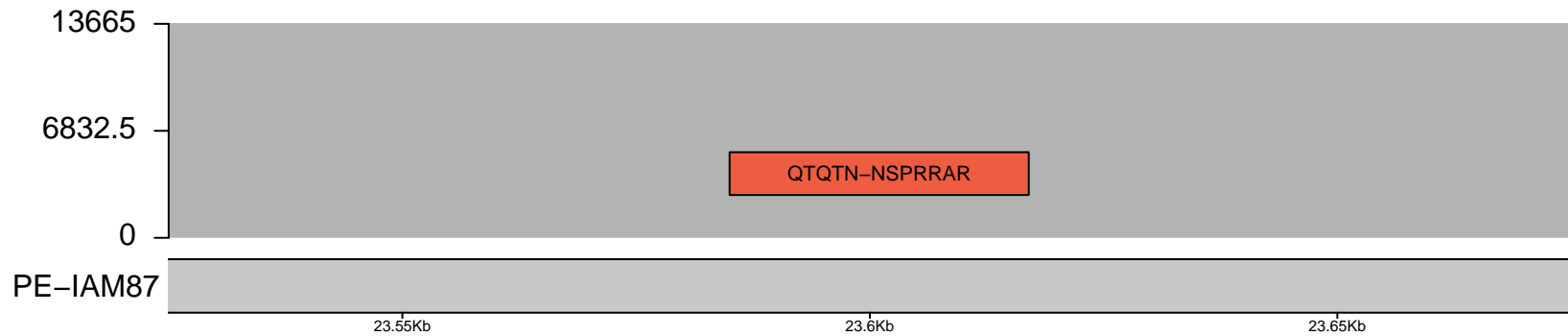

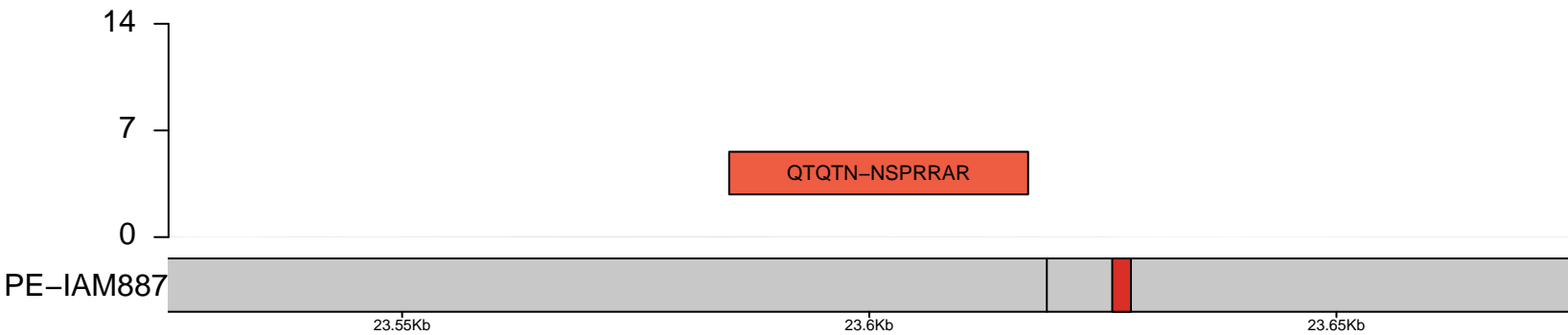

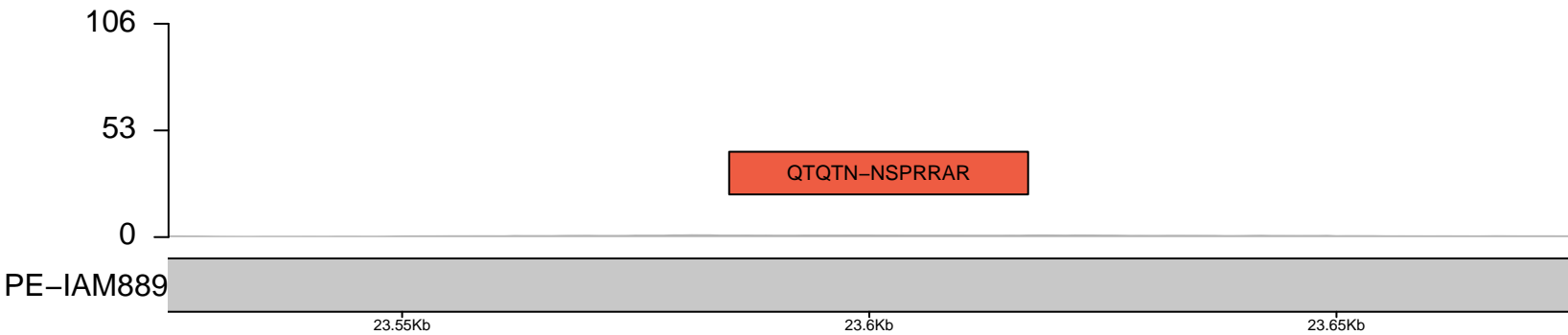

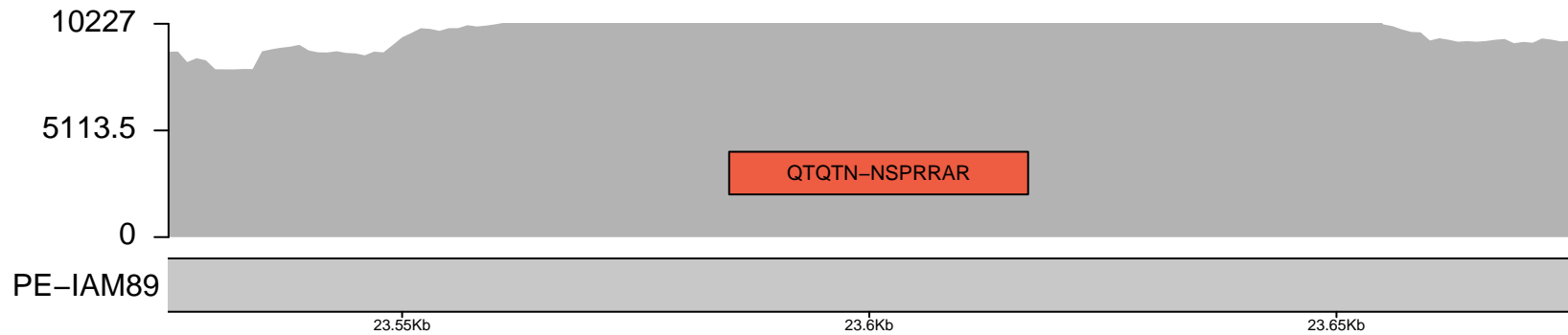

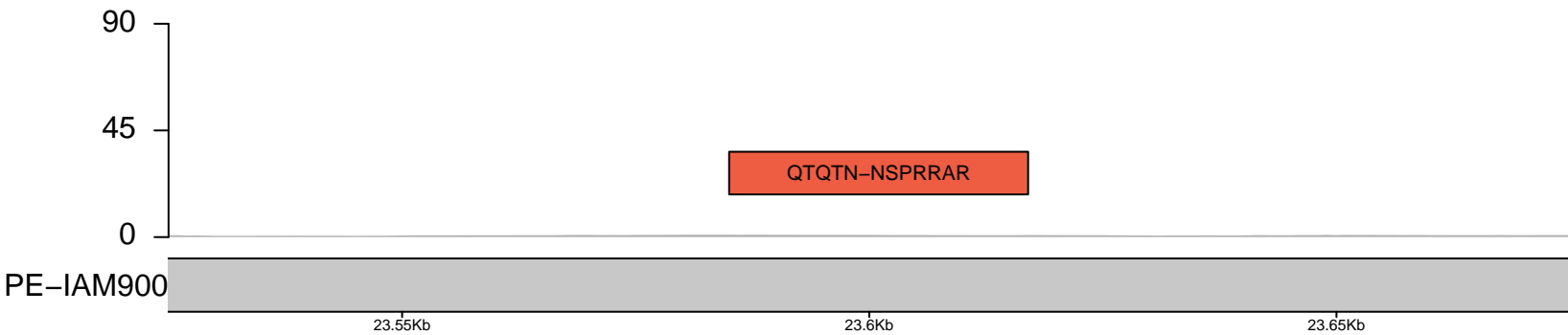

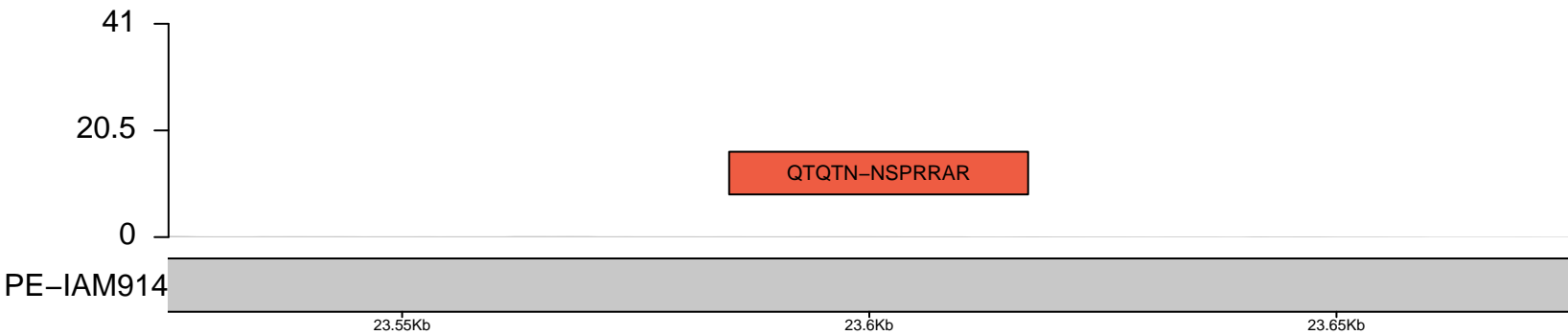

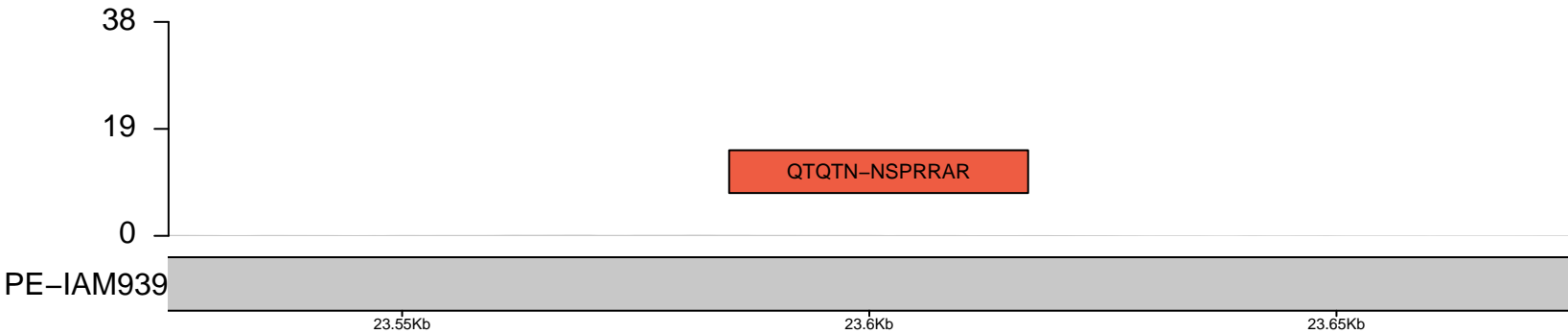

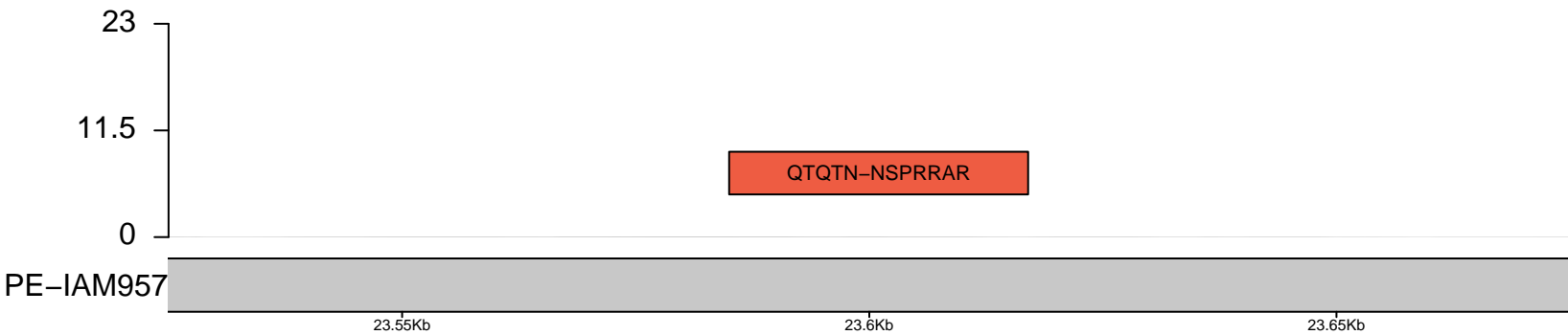

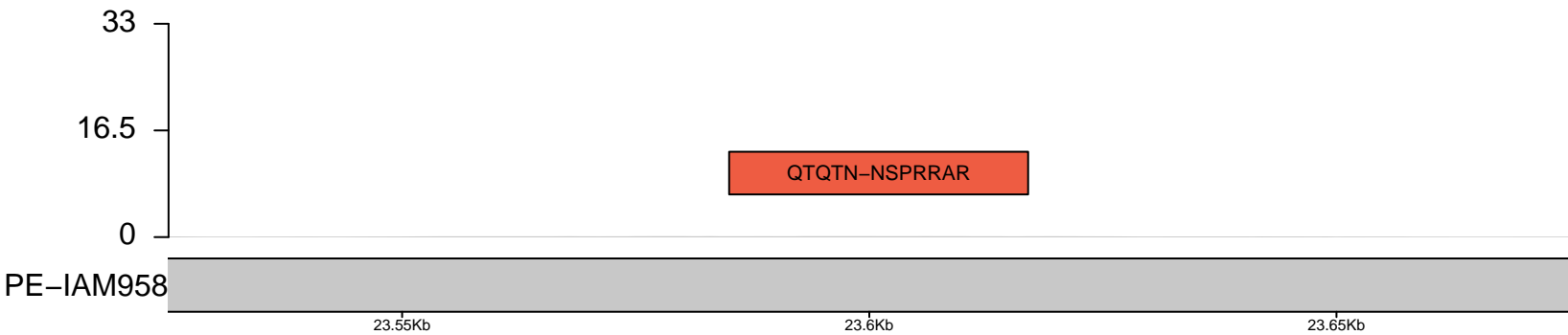

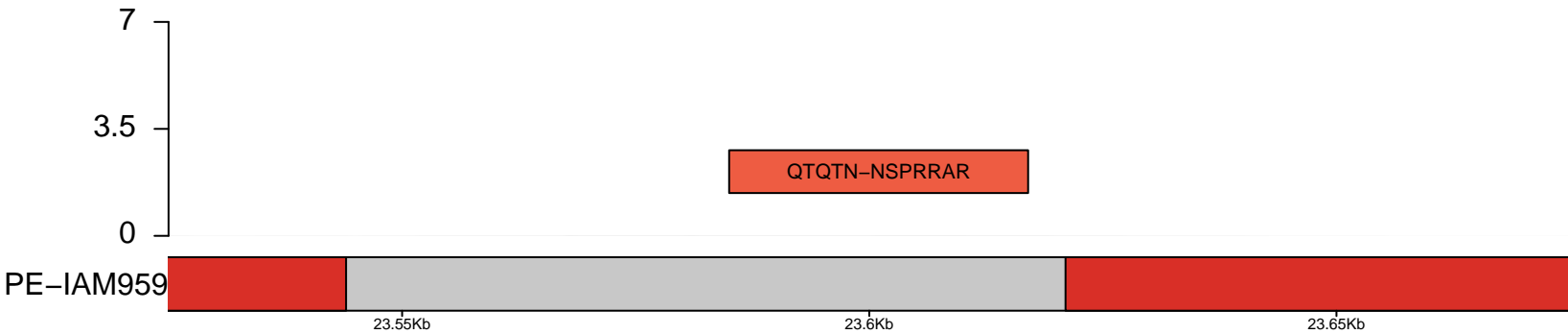

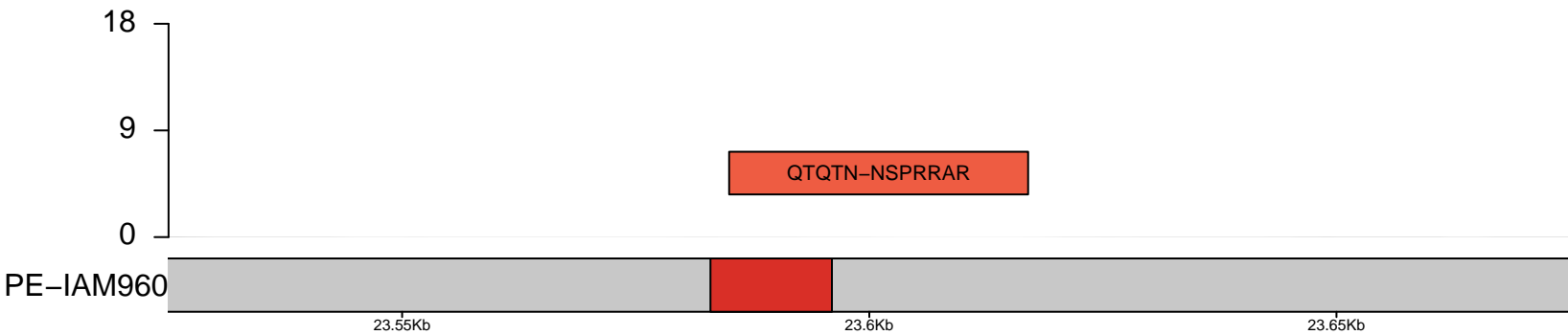

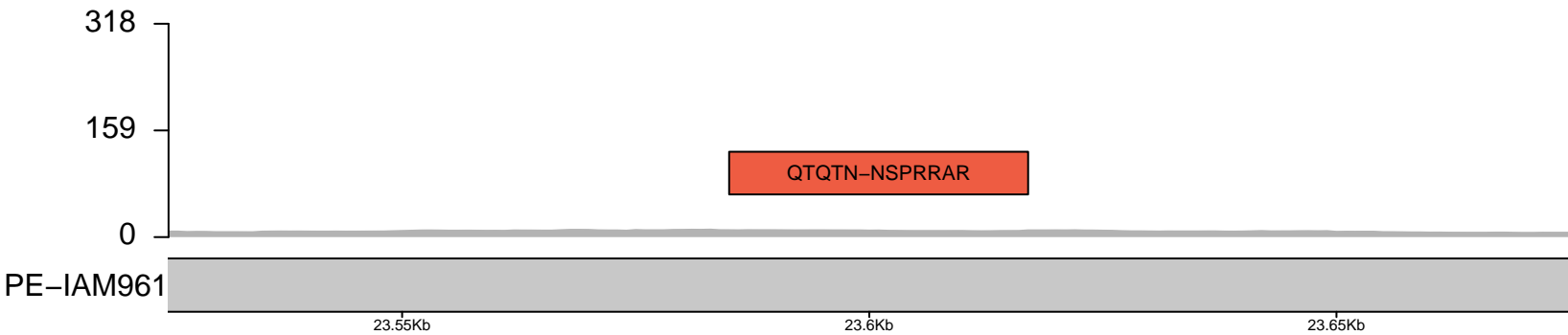

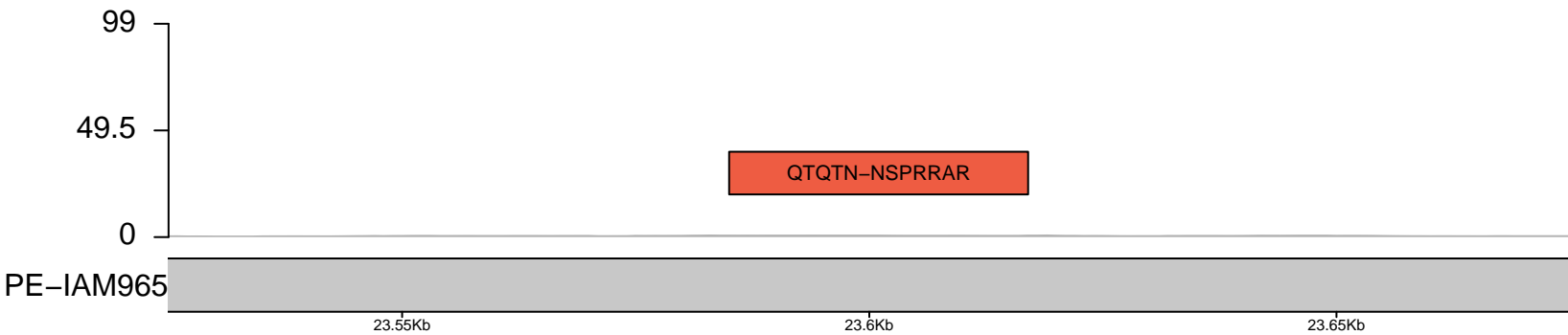

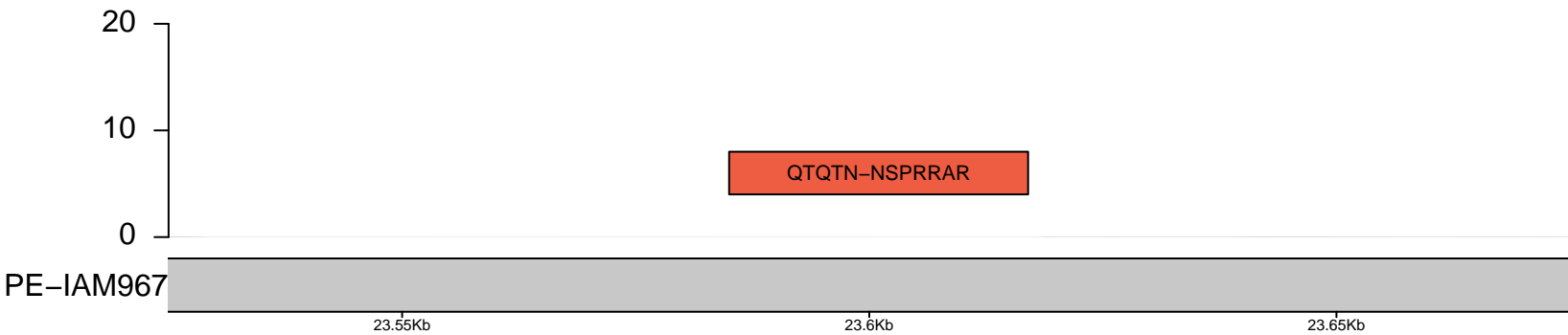

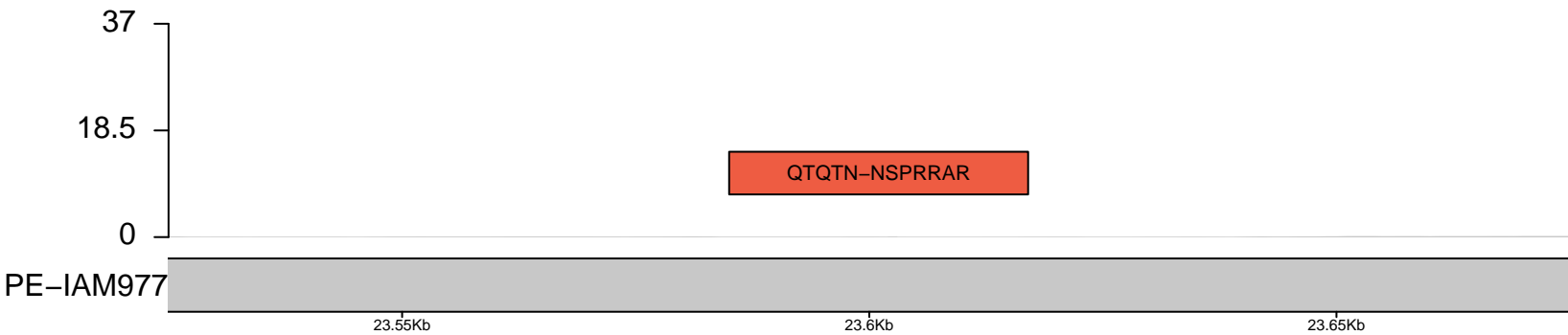

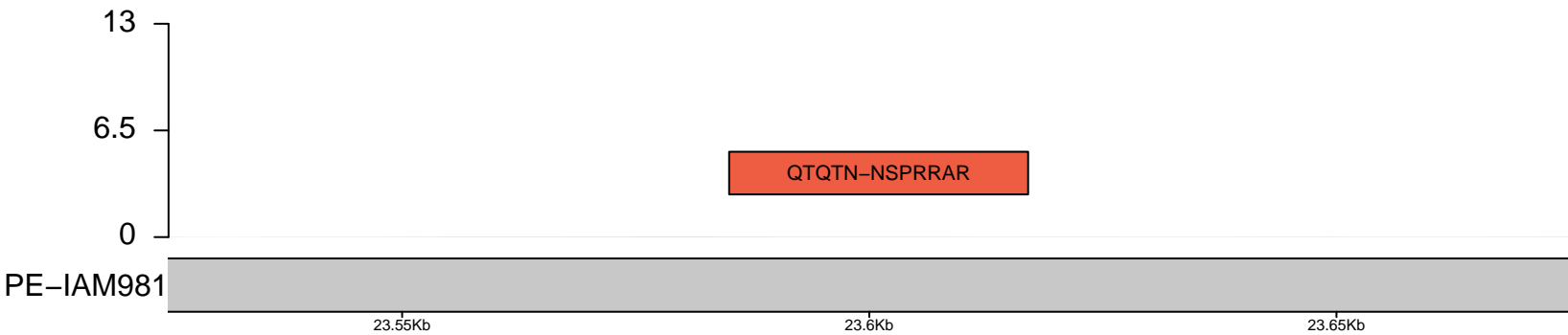

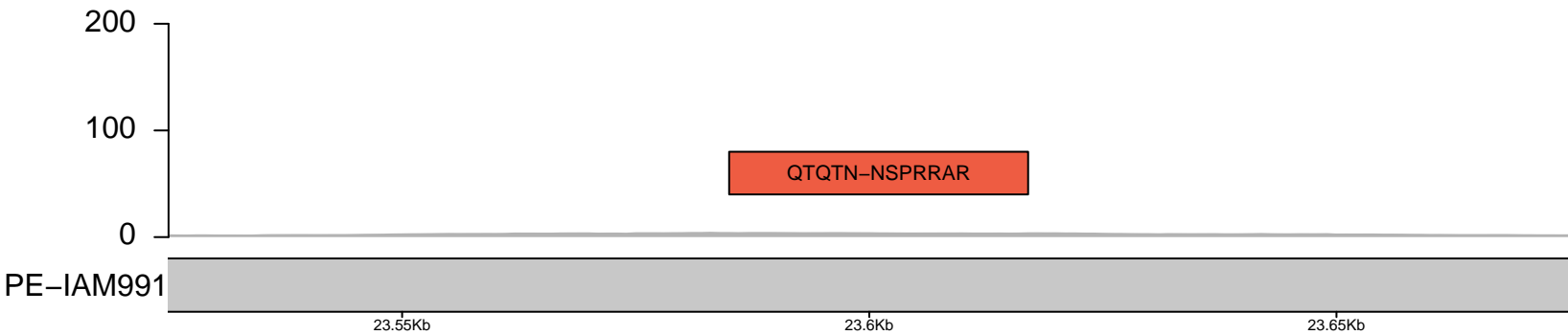

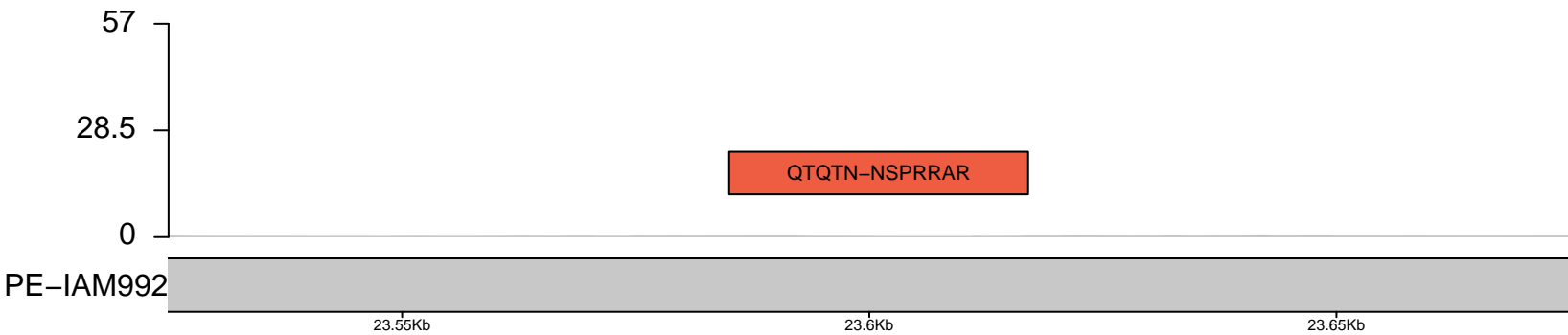

Supplement: Supplementary file 1 [file viruses-12-01414-s001.zip › Supplementary_Material/Supplementary_File_2.pdf]

8000  
4000  
0

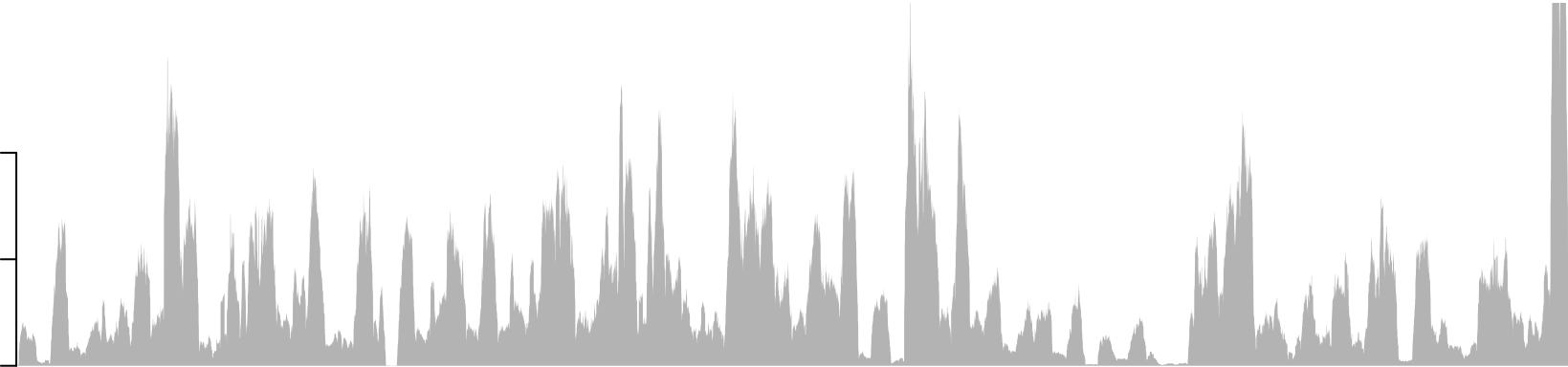

PE-IAM08

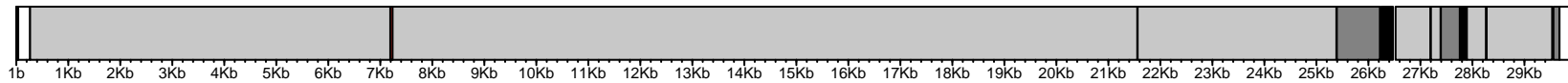

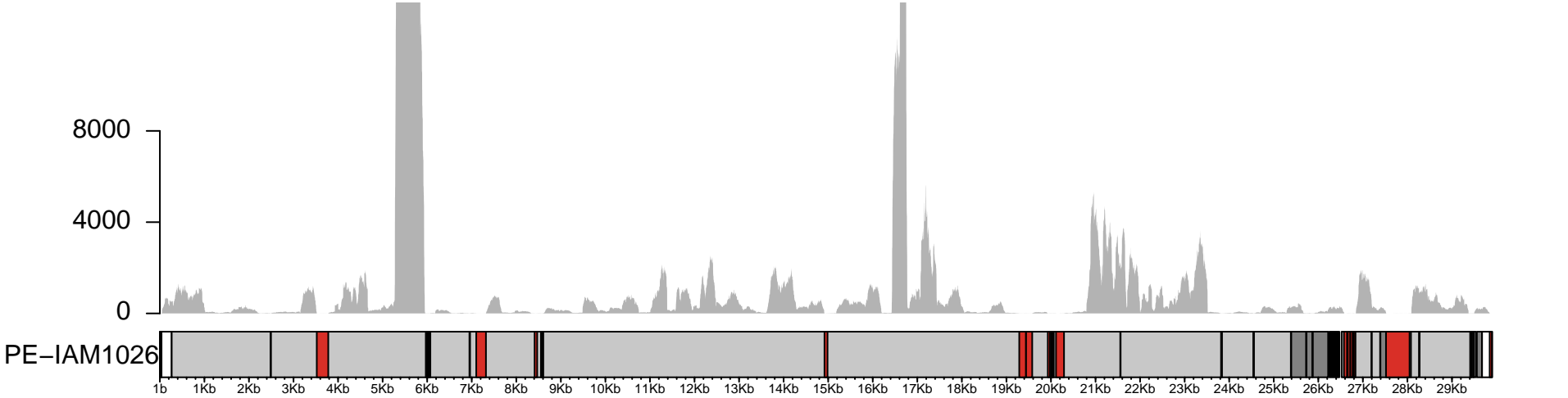

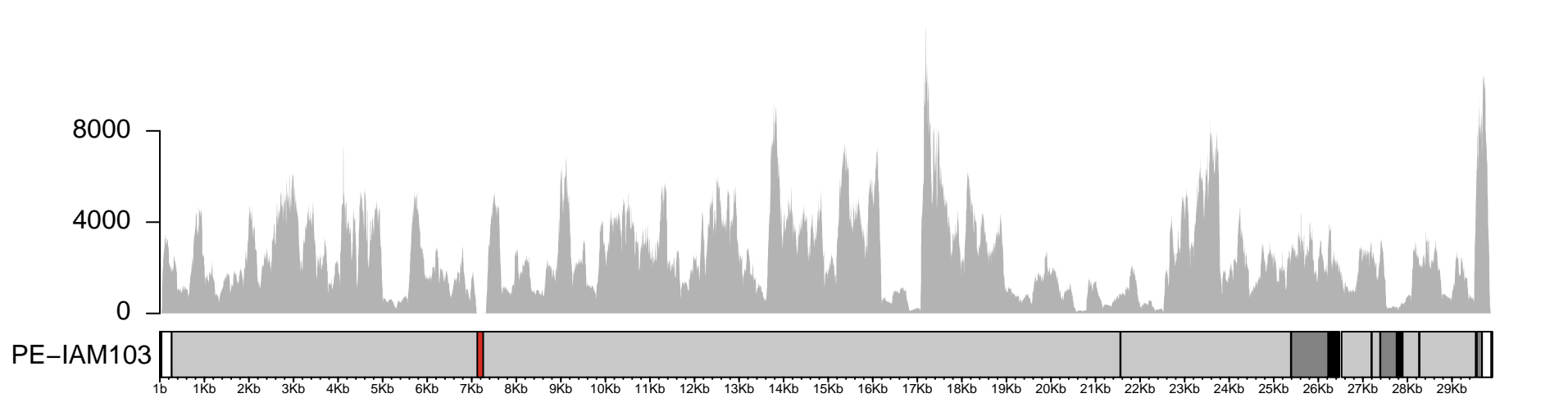

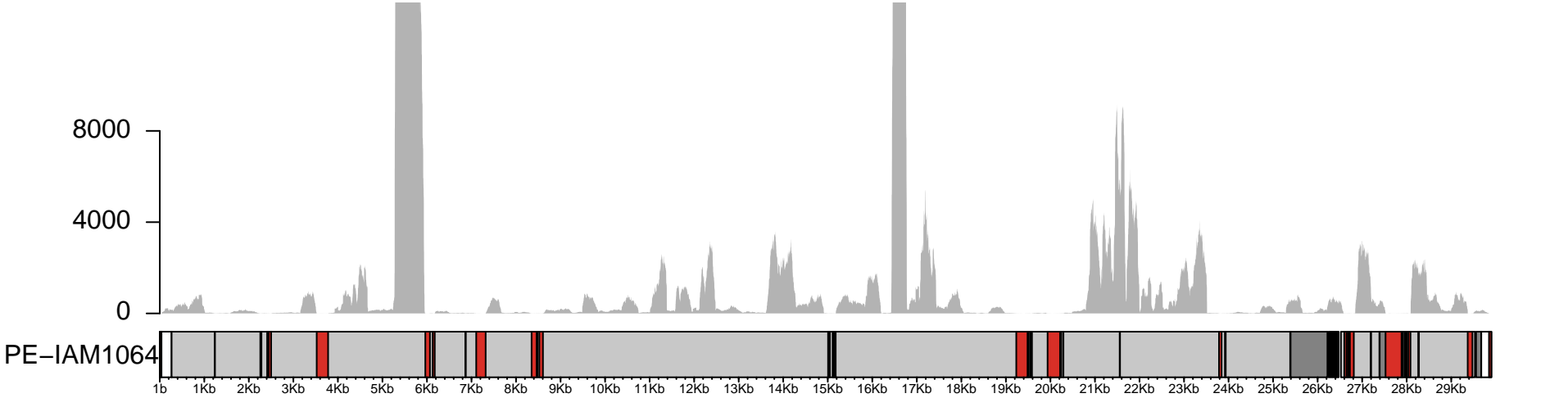

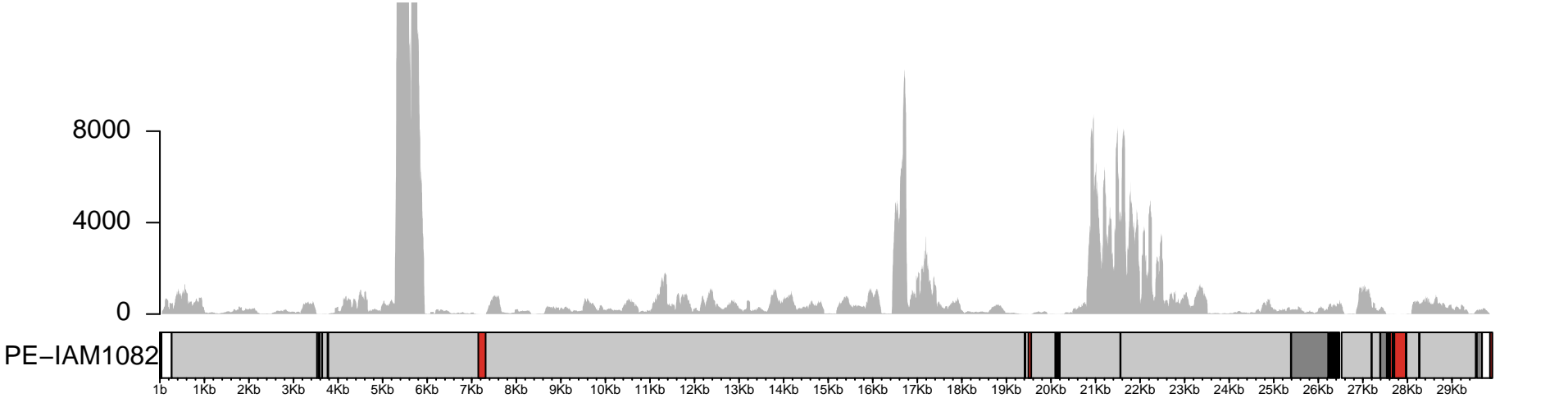

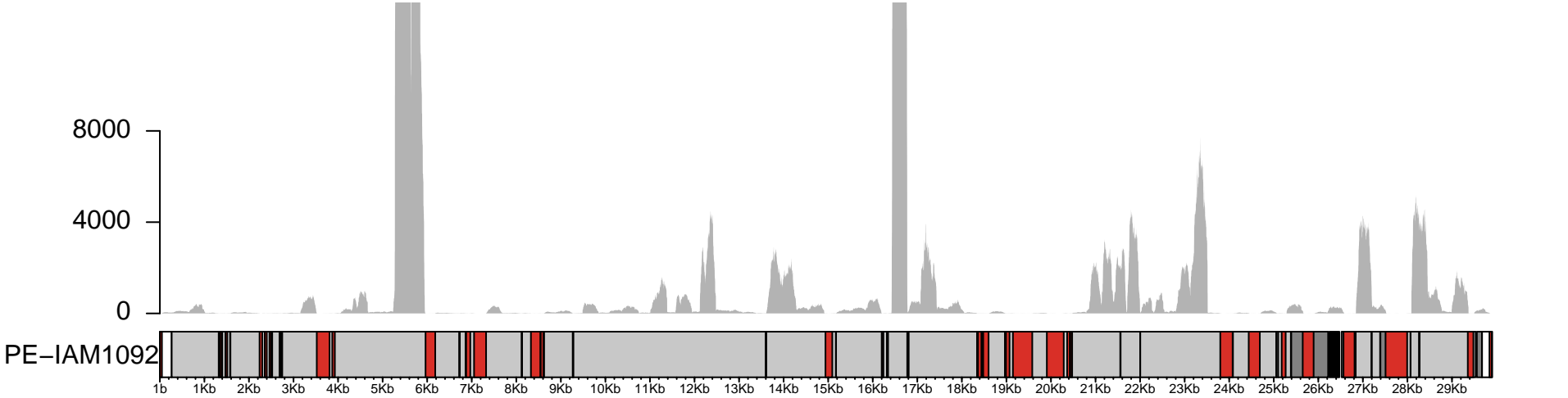

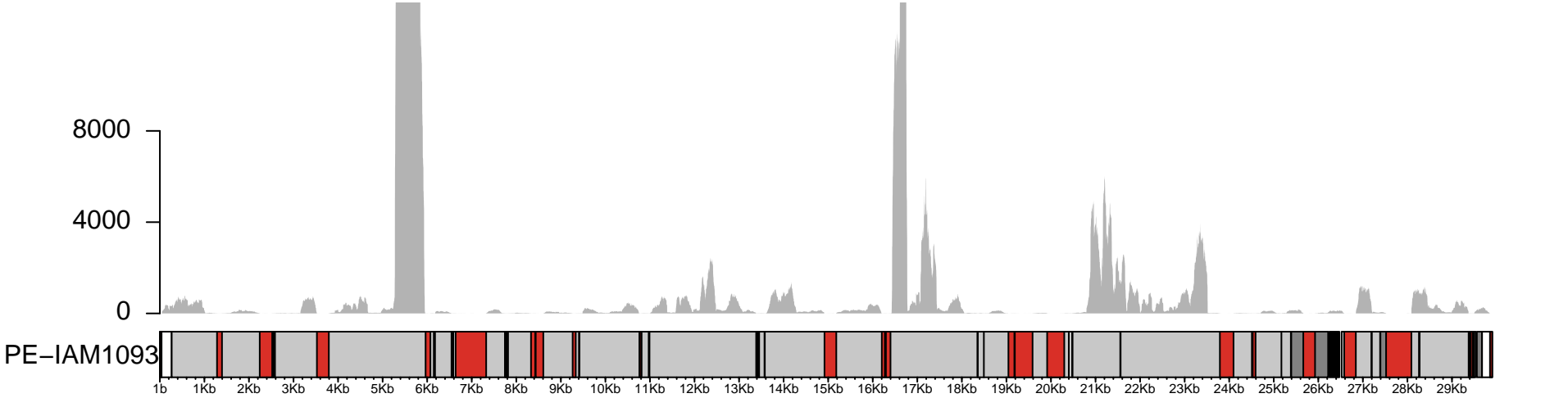

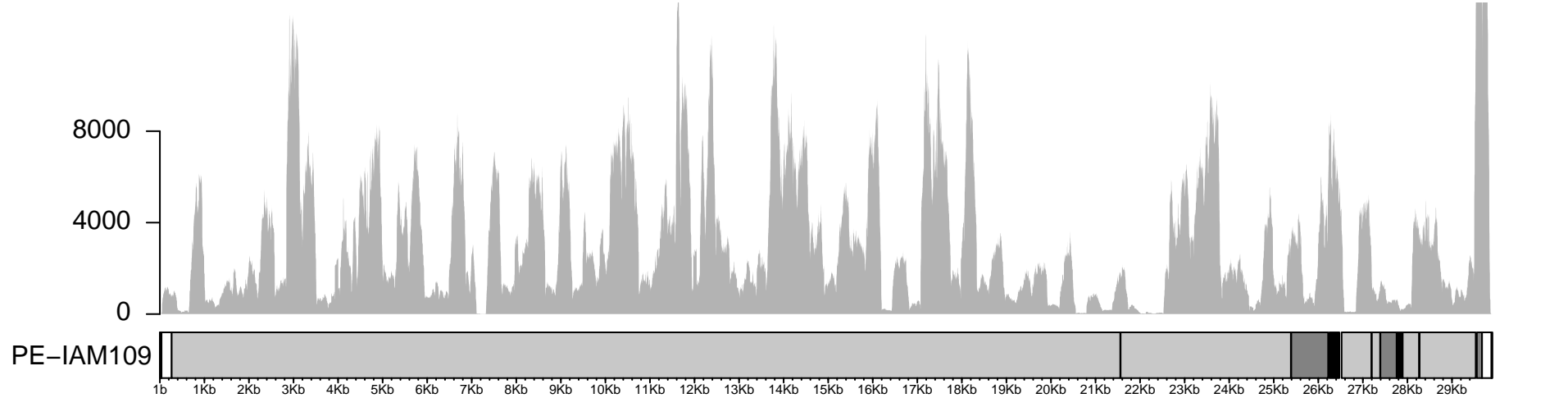

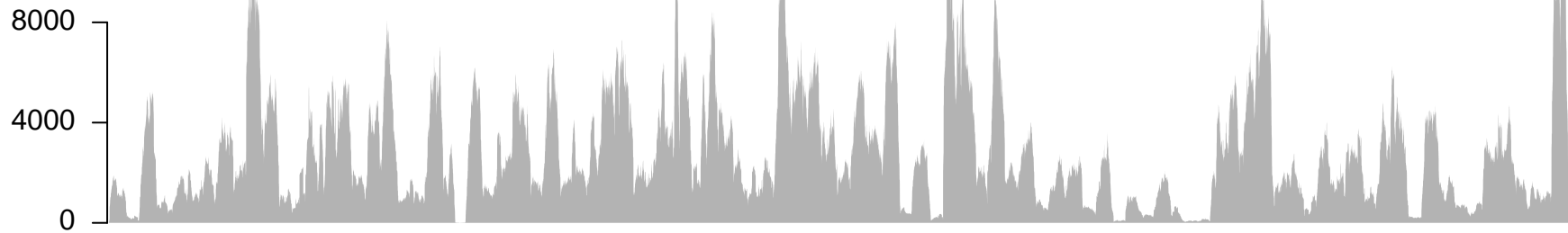

PE-IAM10

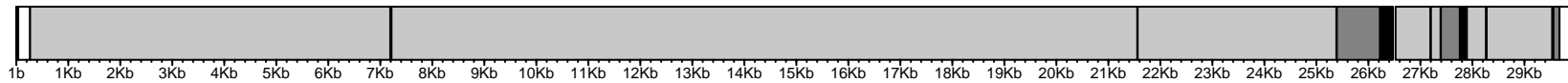

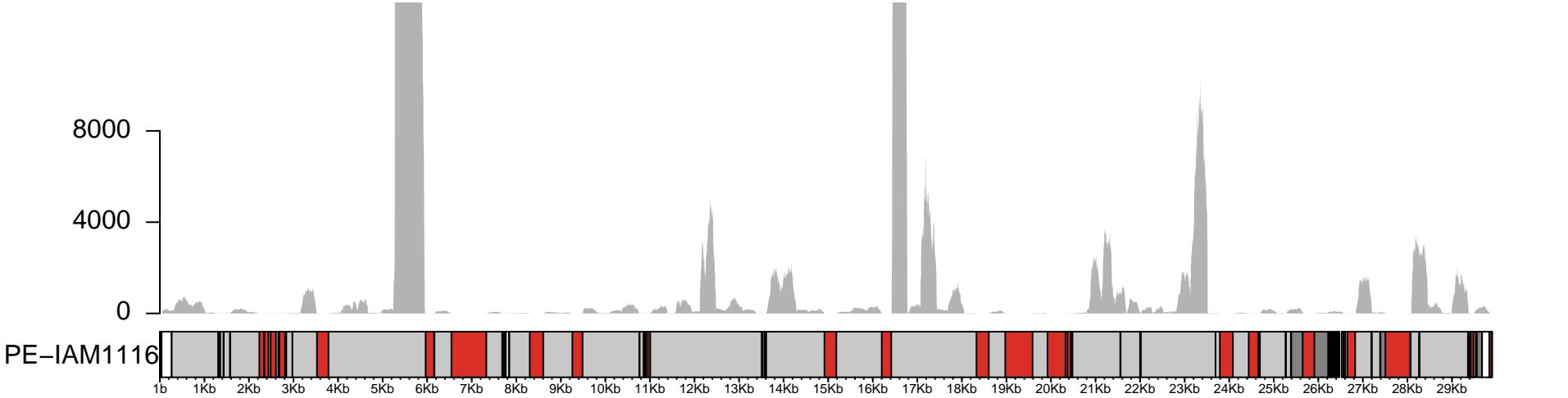

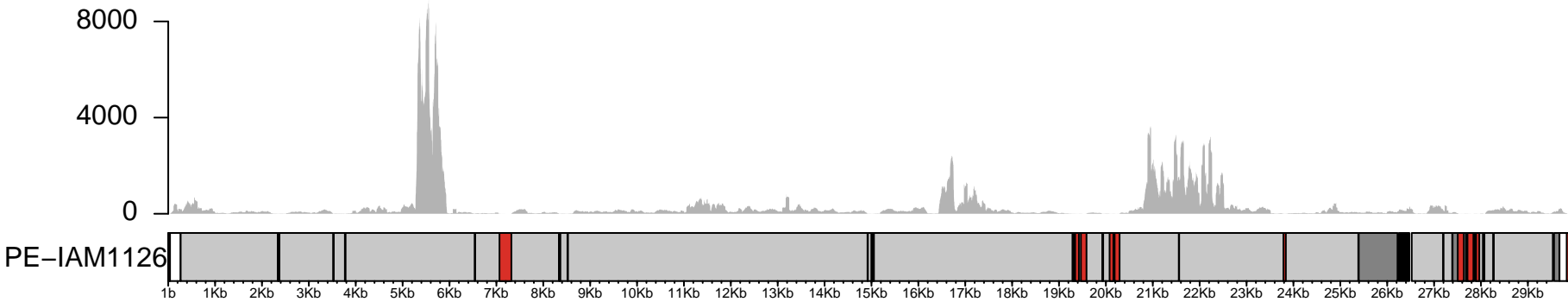

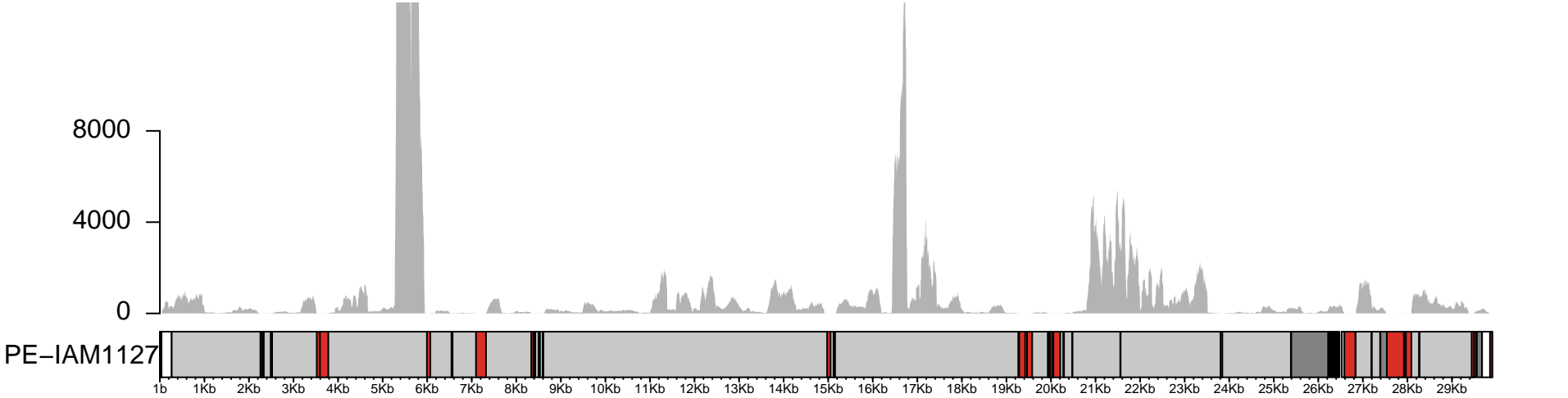

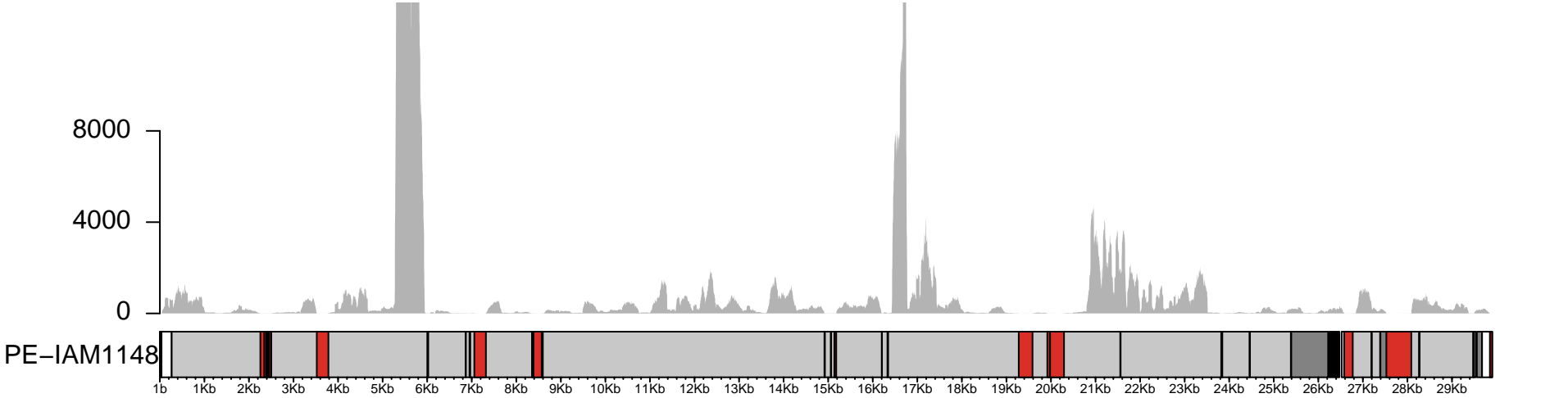

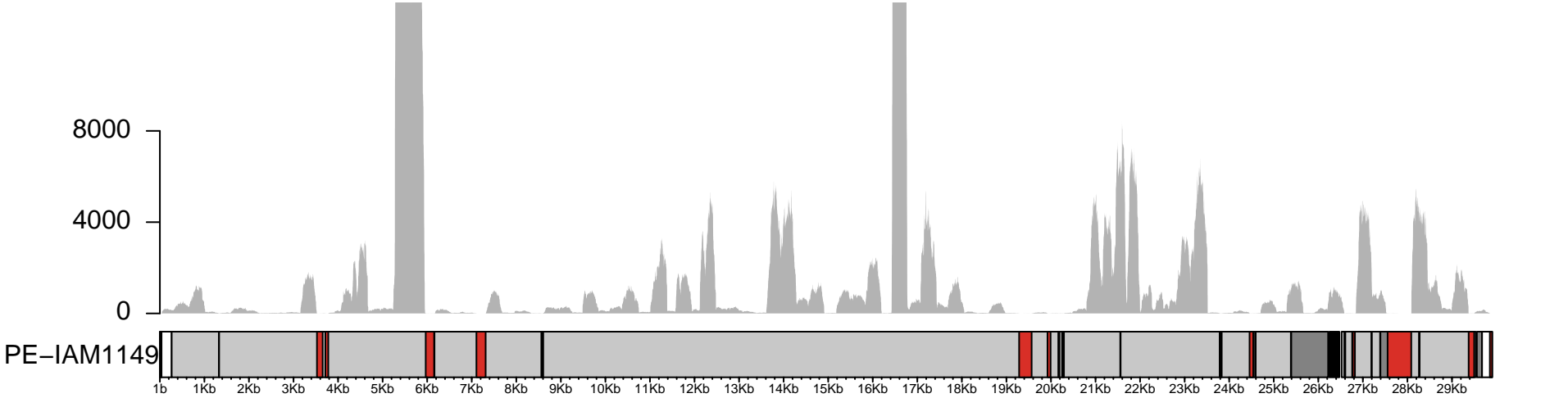

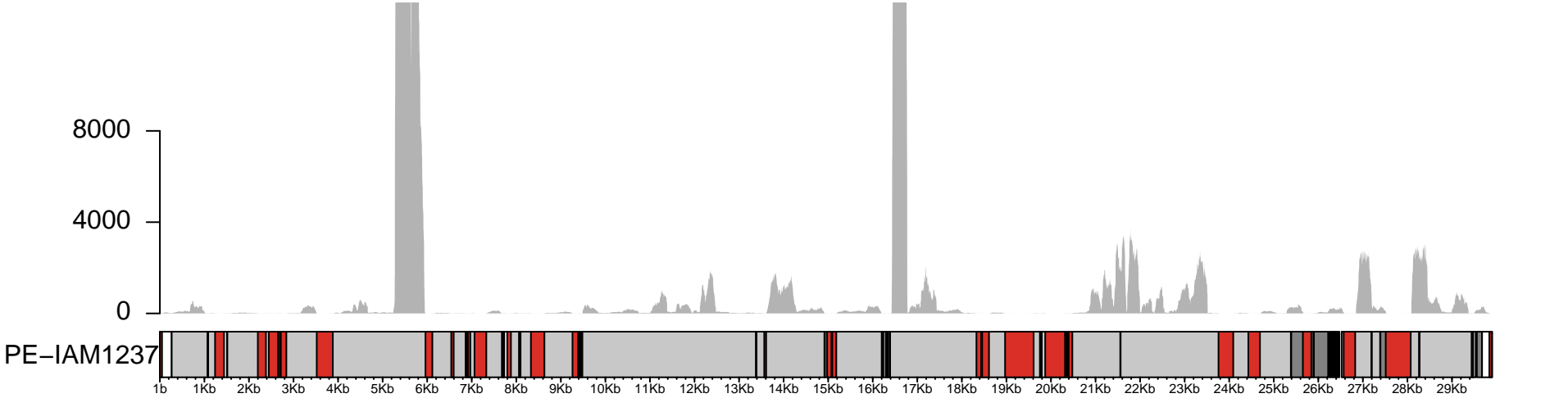

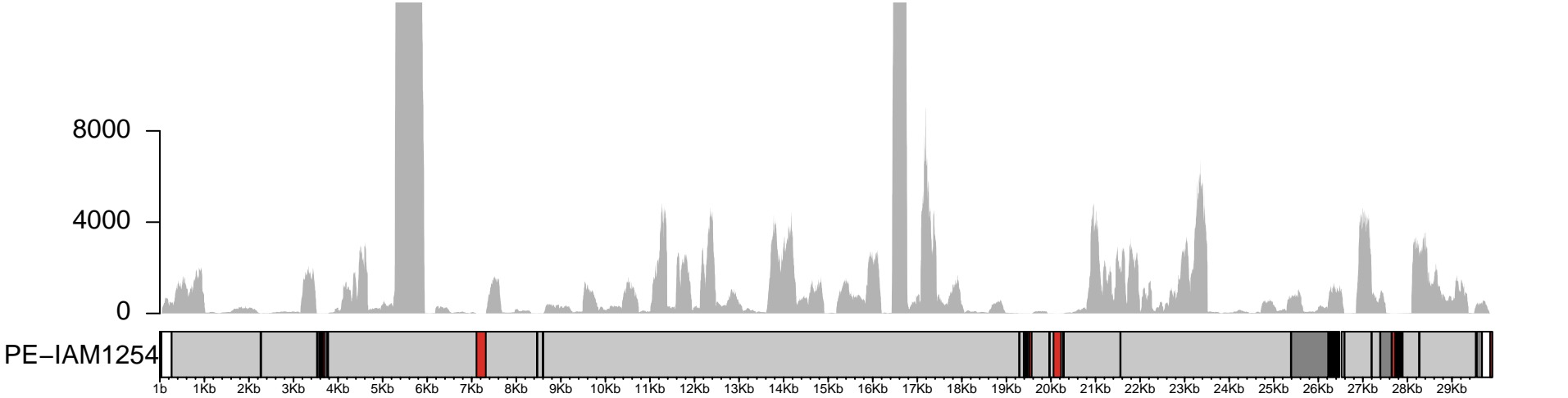

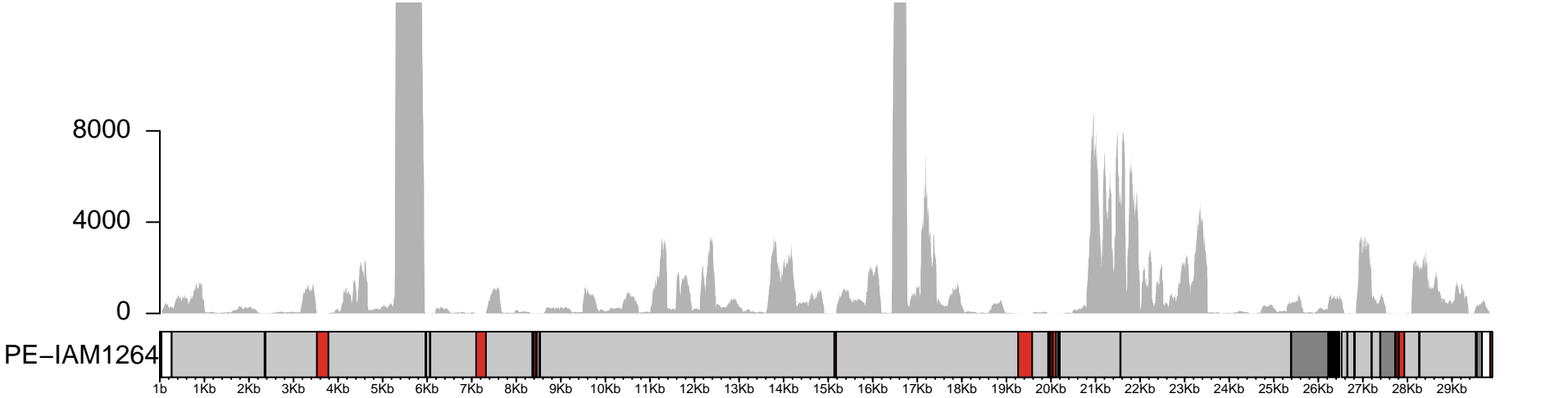

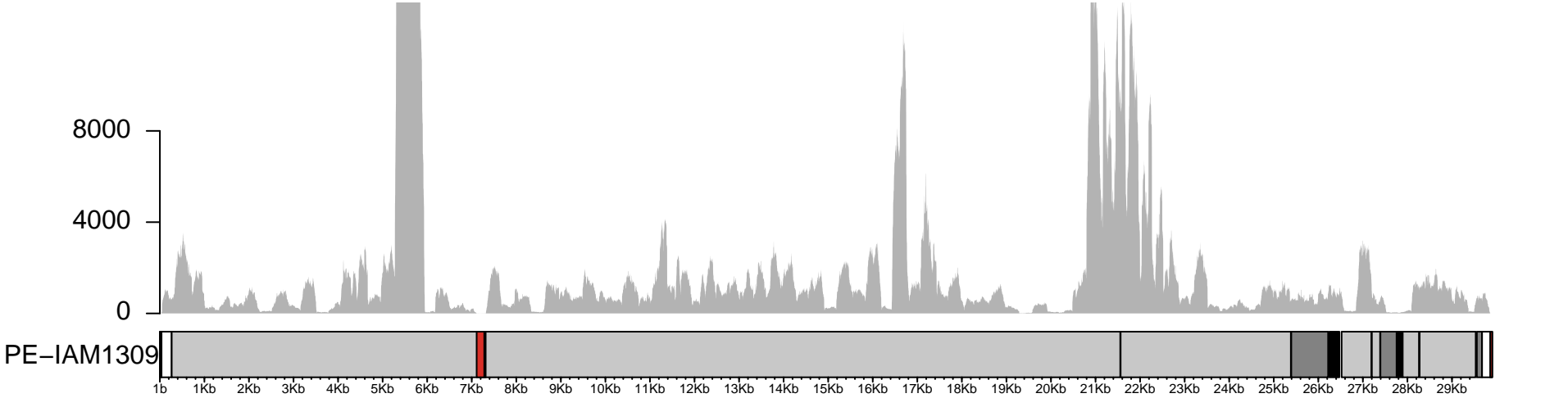

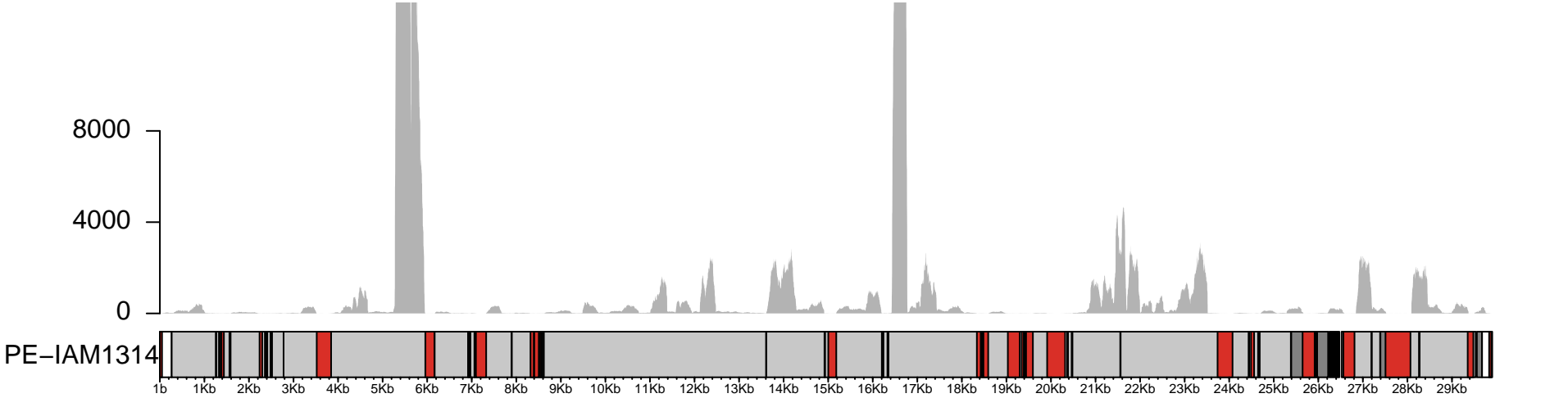

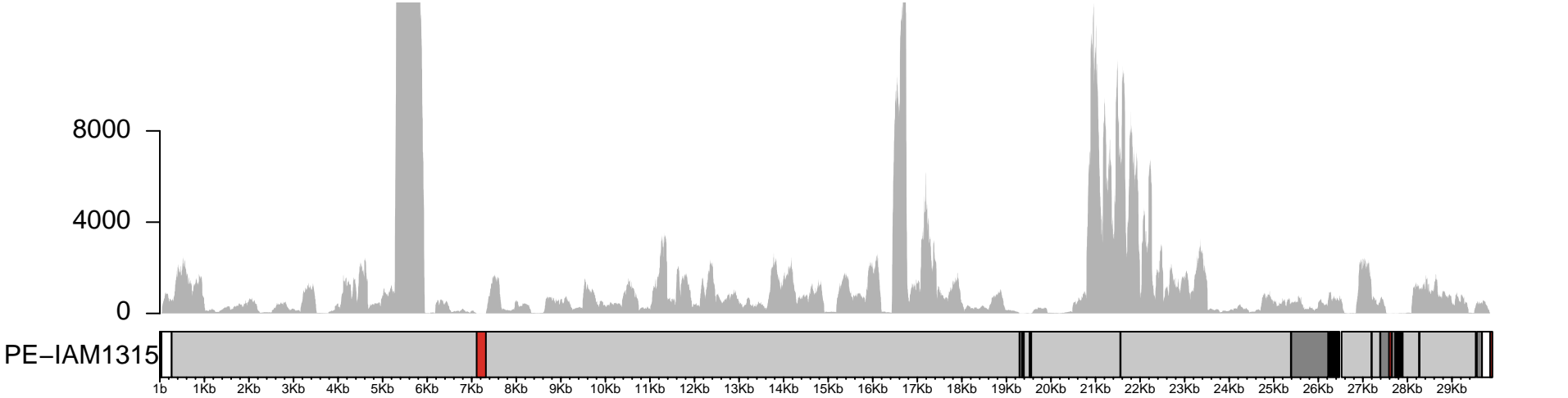

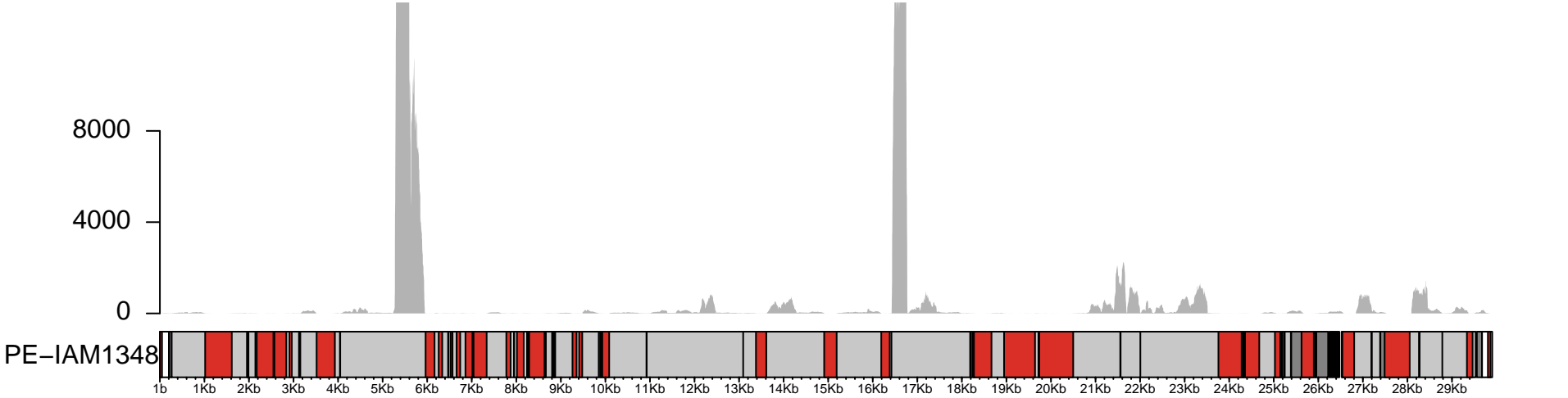

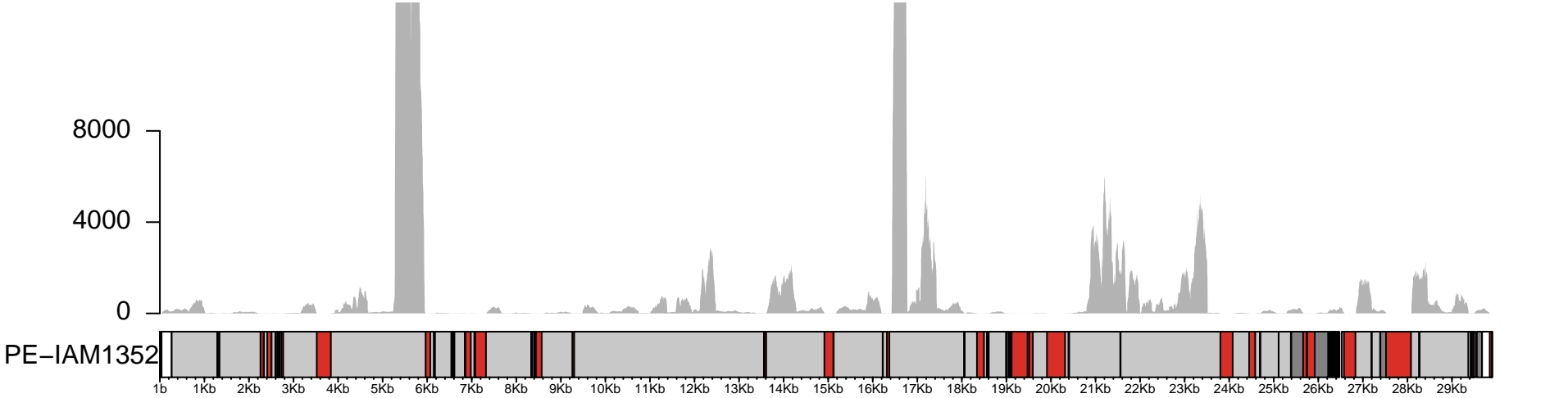

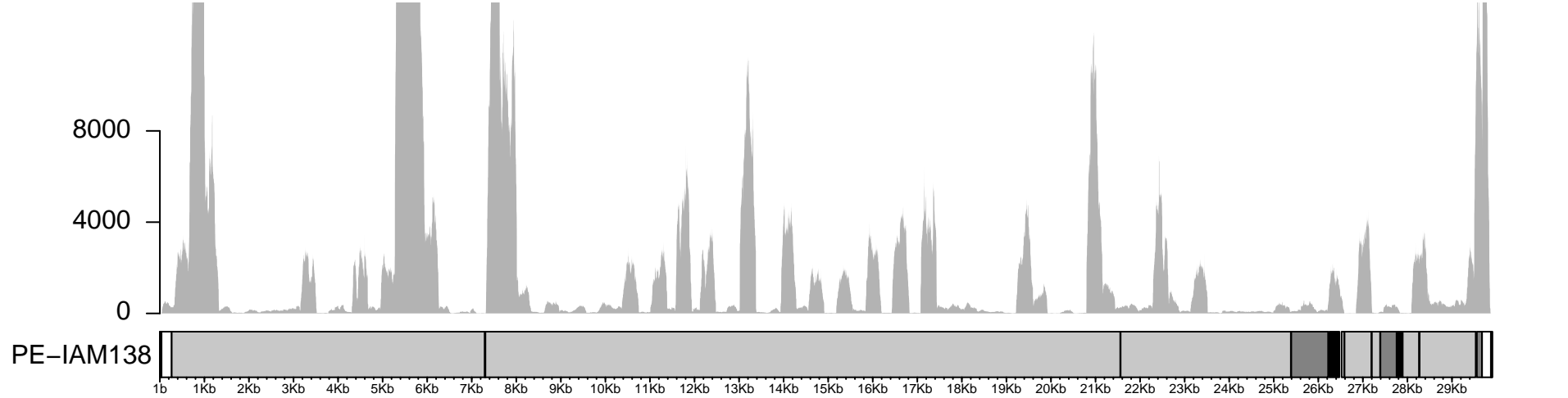

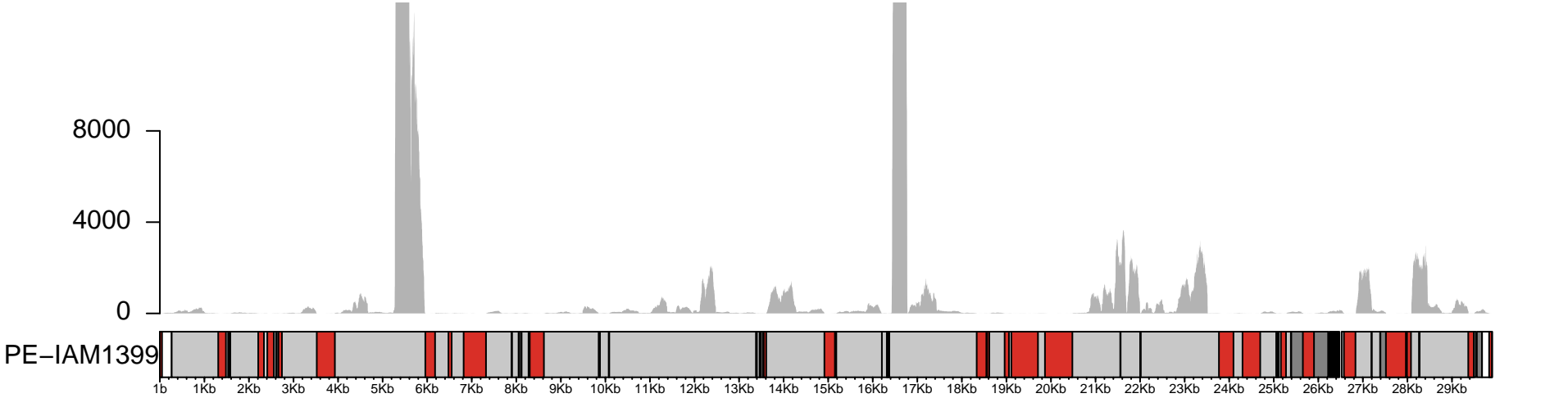

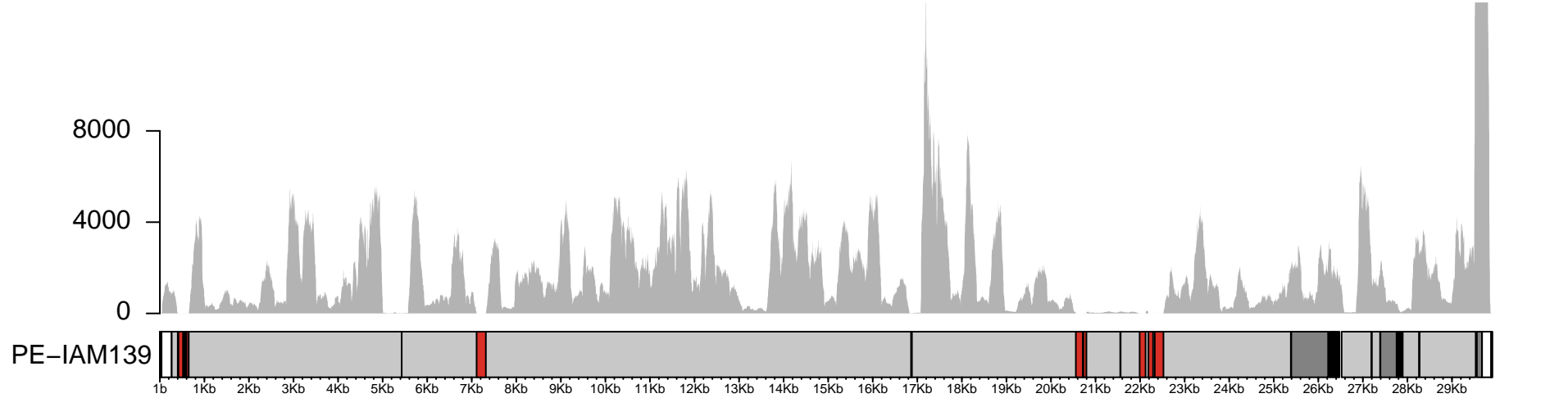

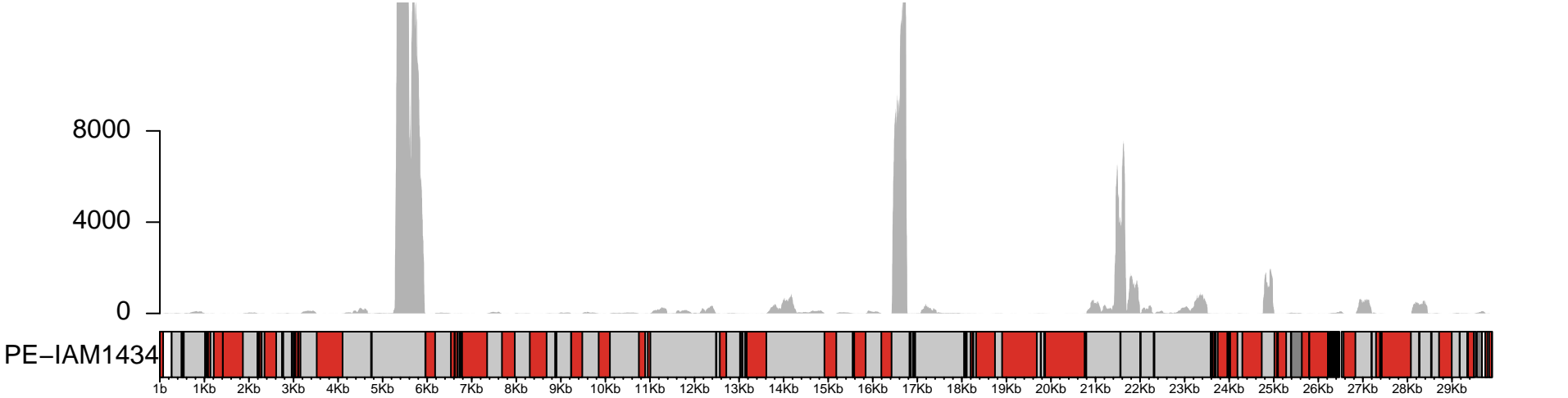

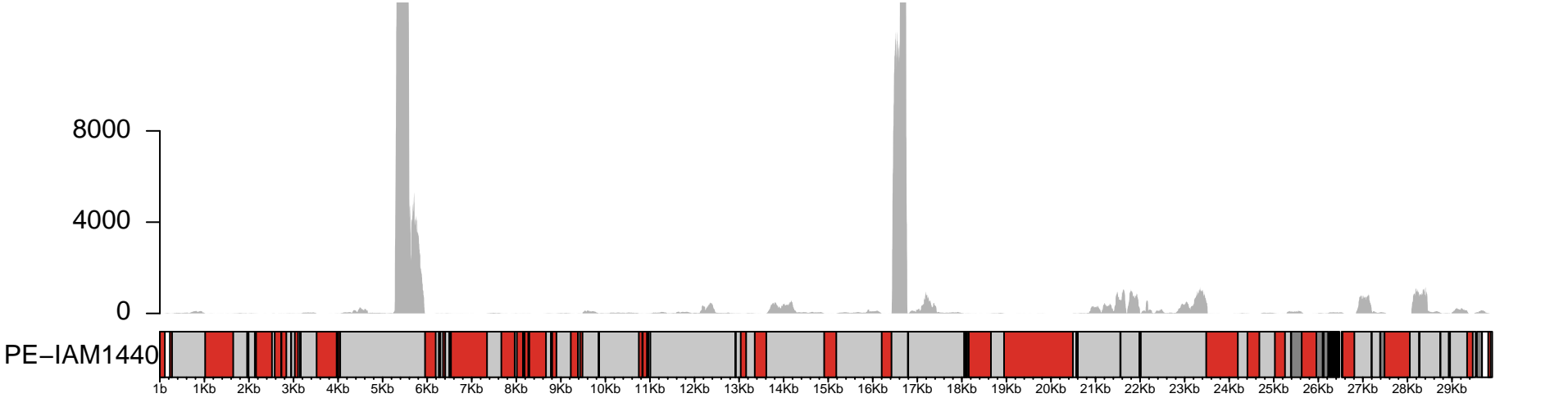

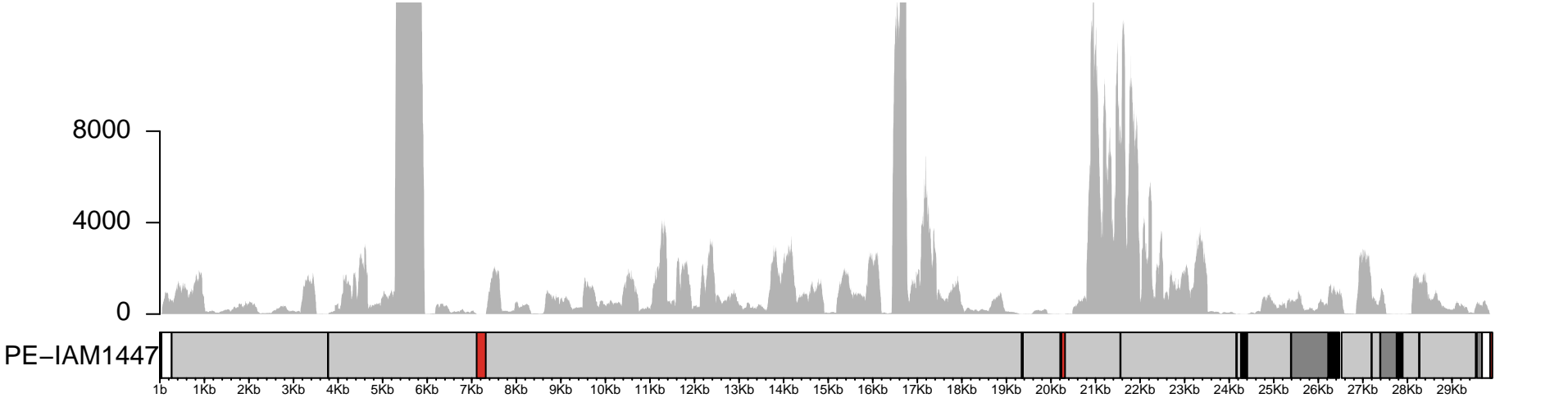

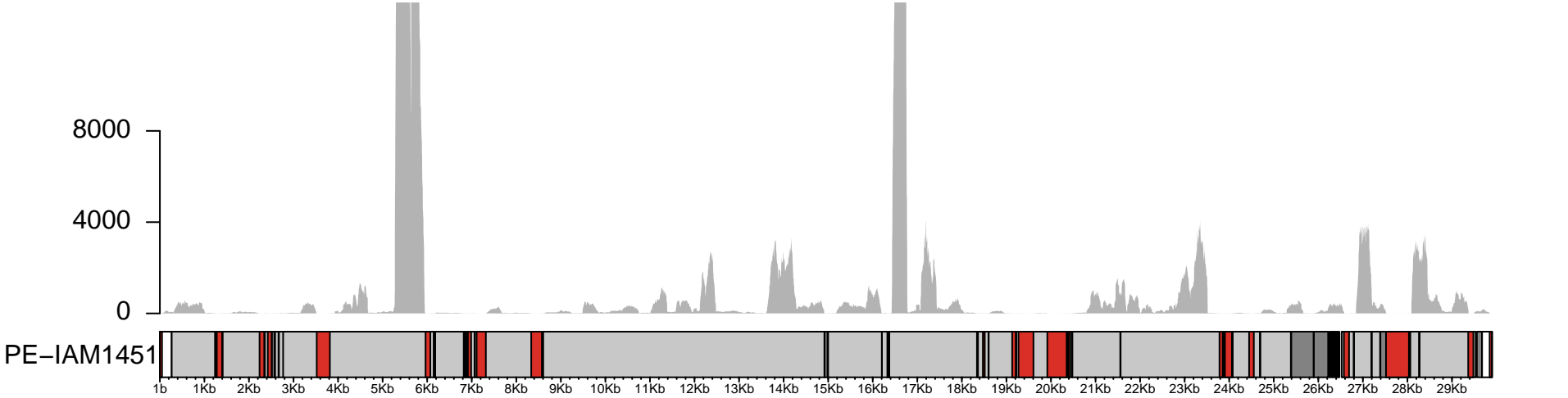

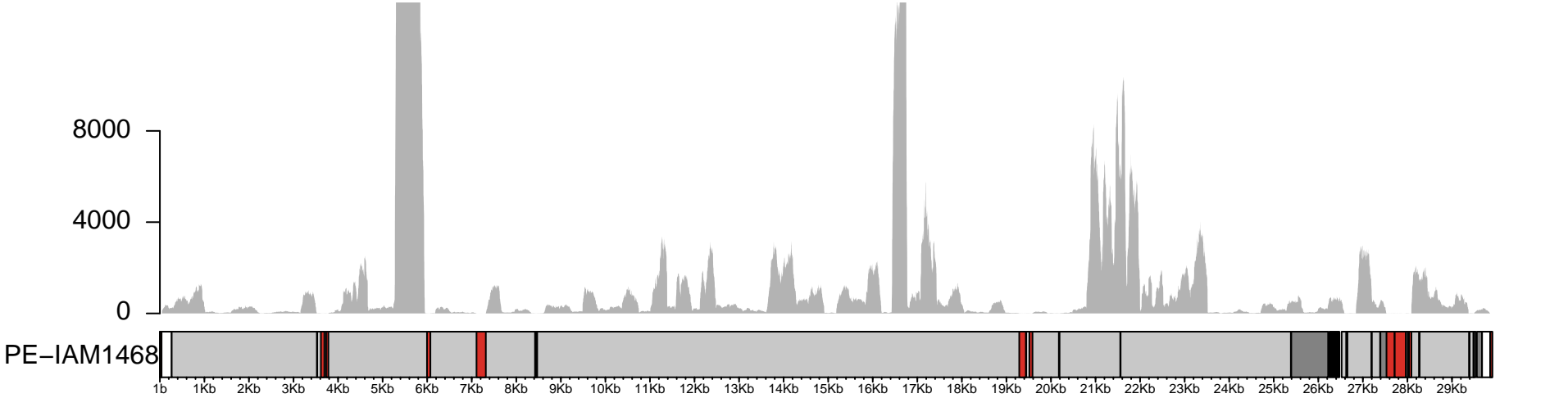

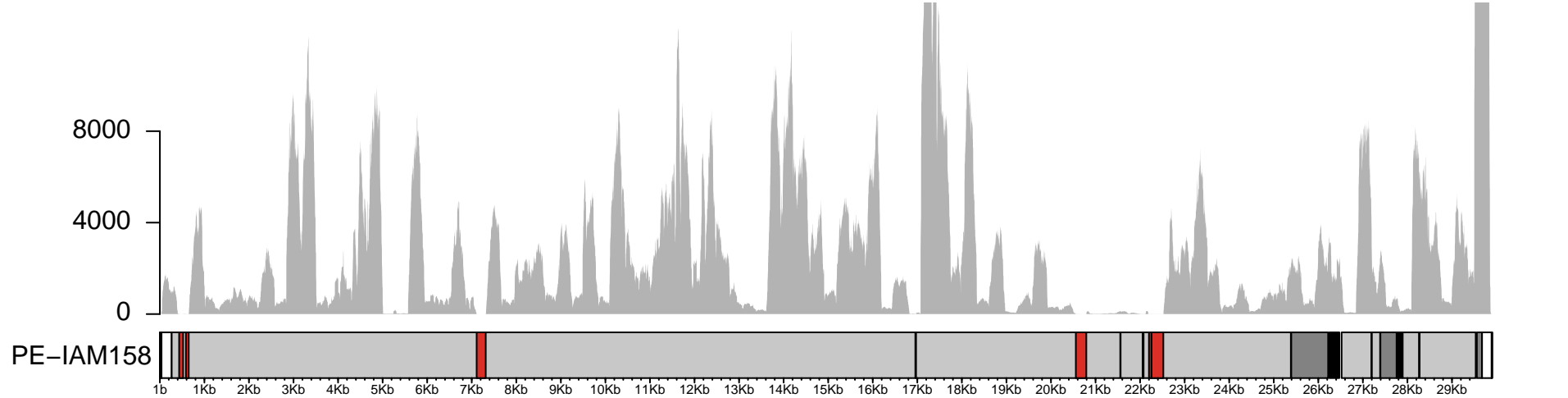

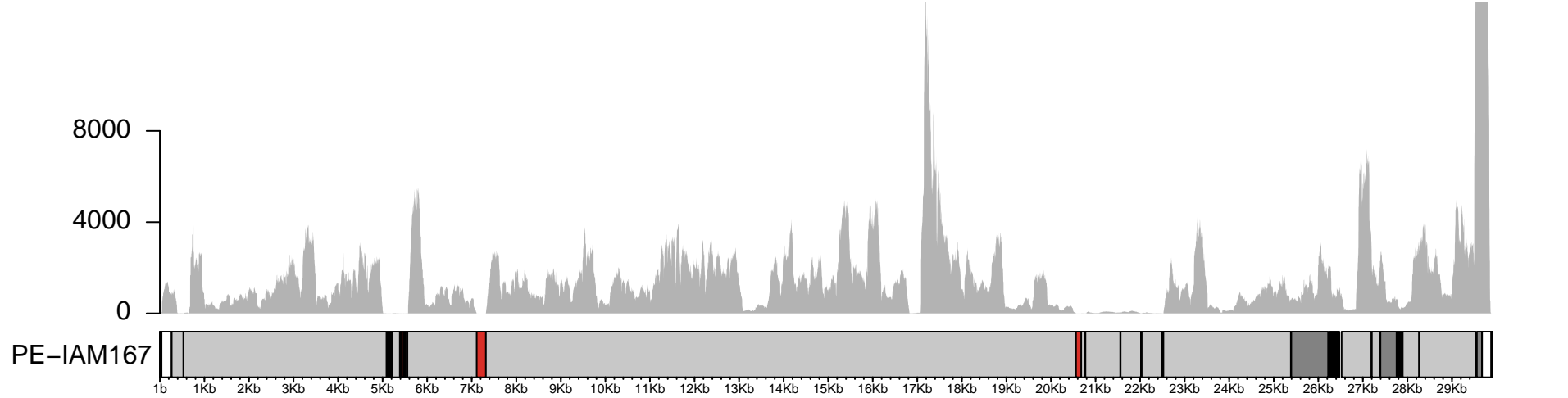

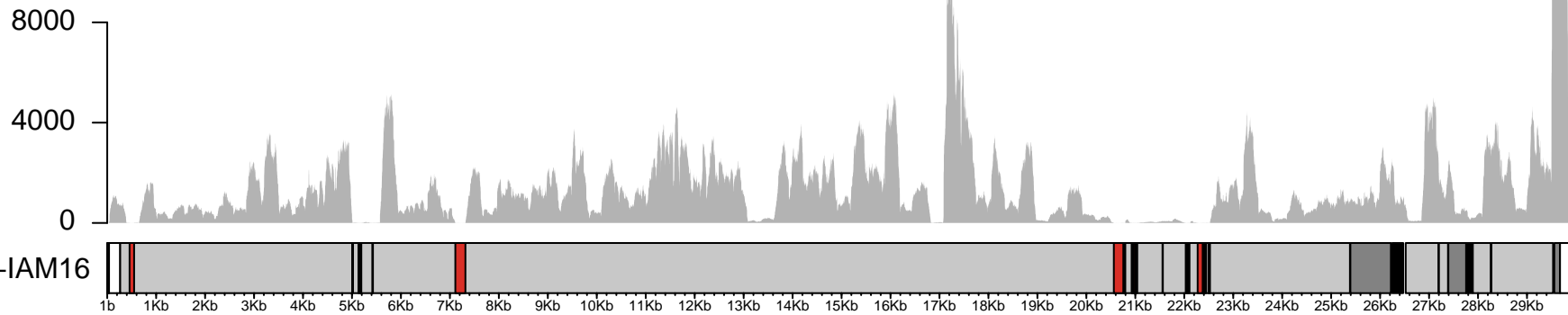

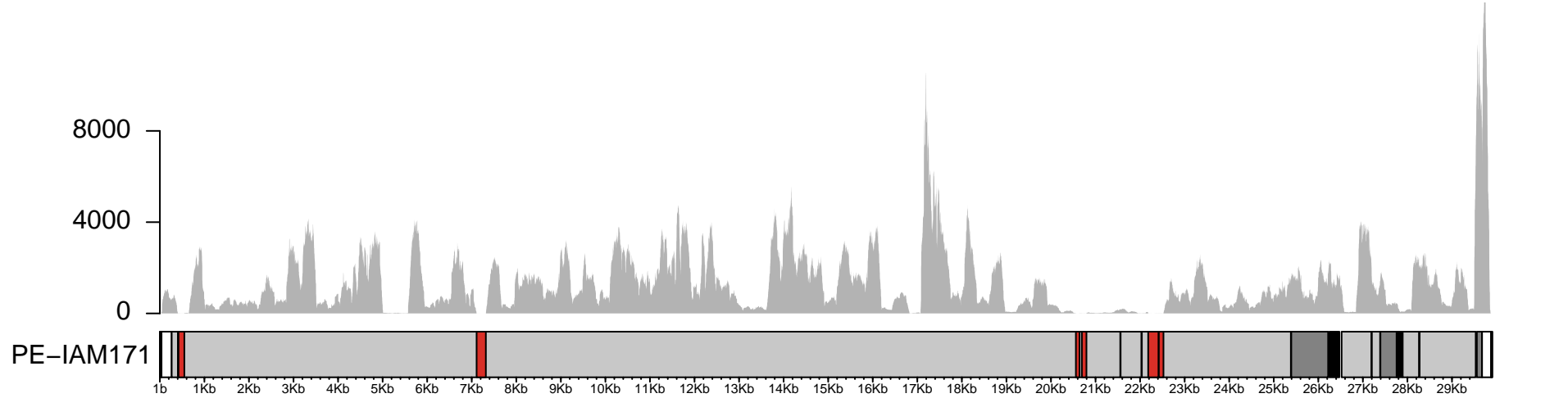

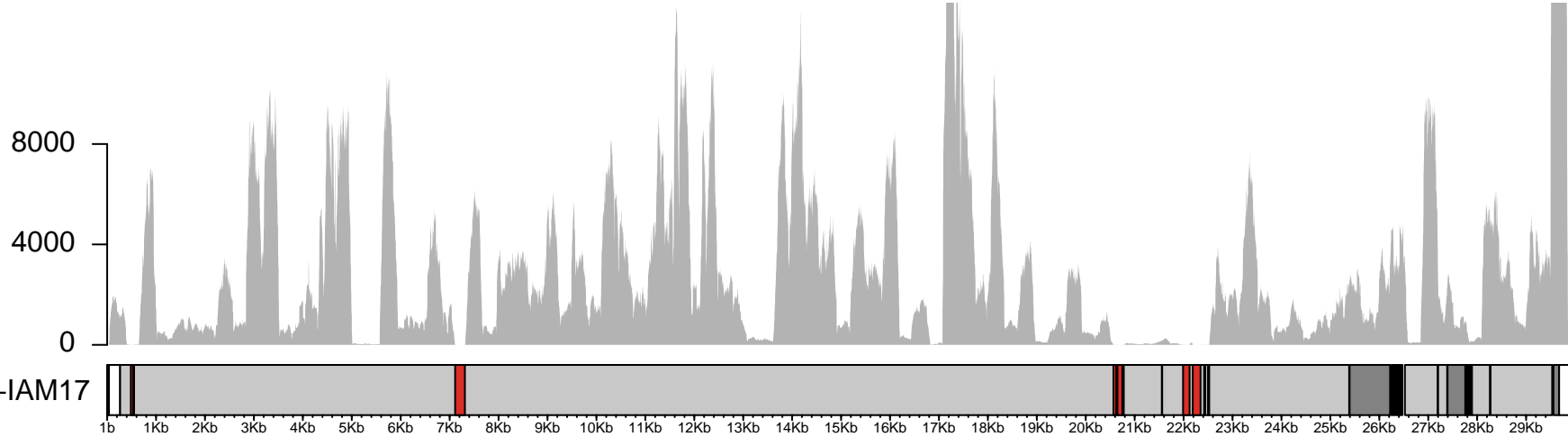

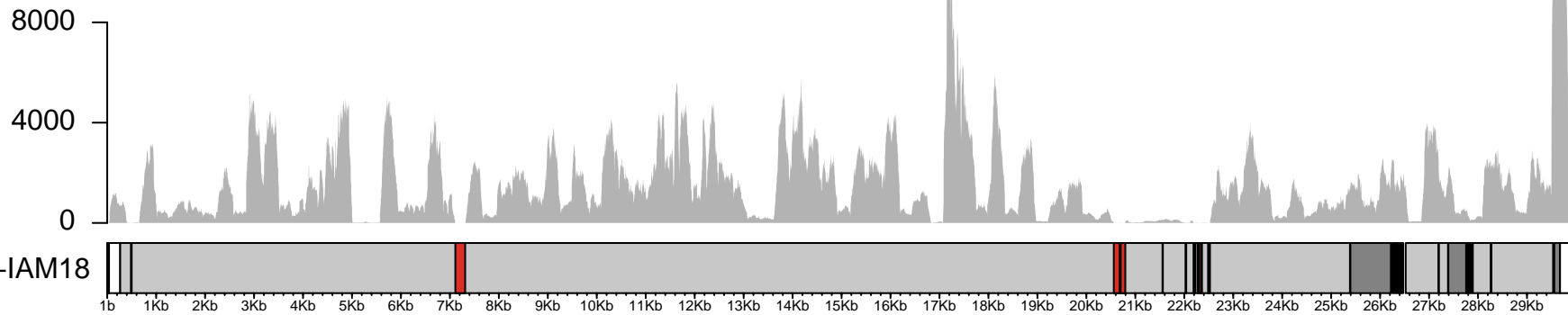

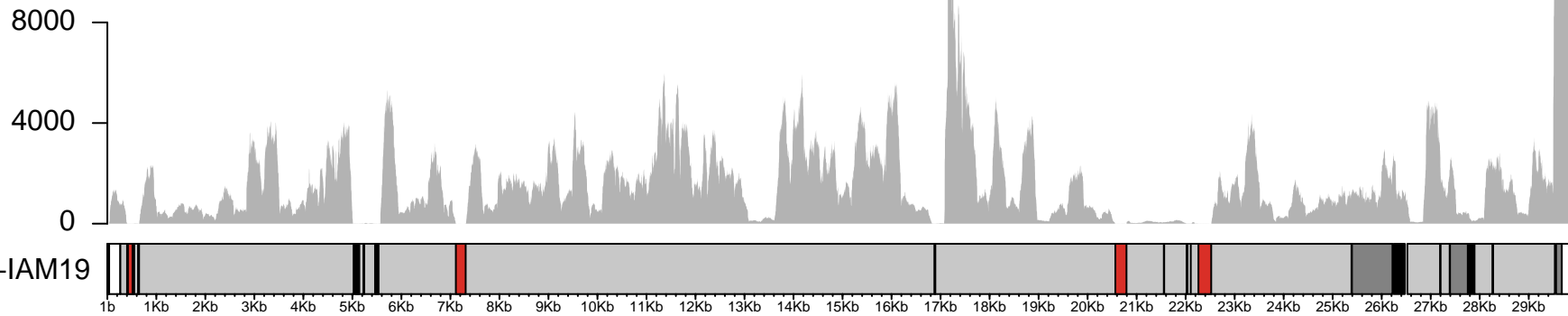

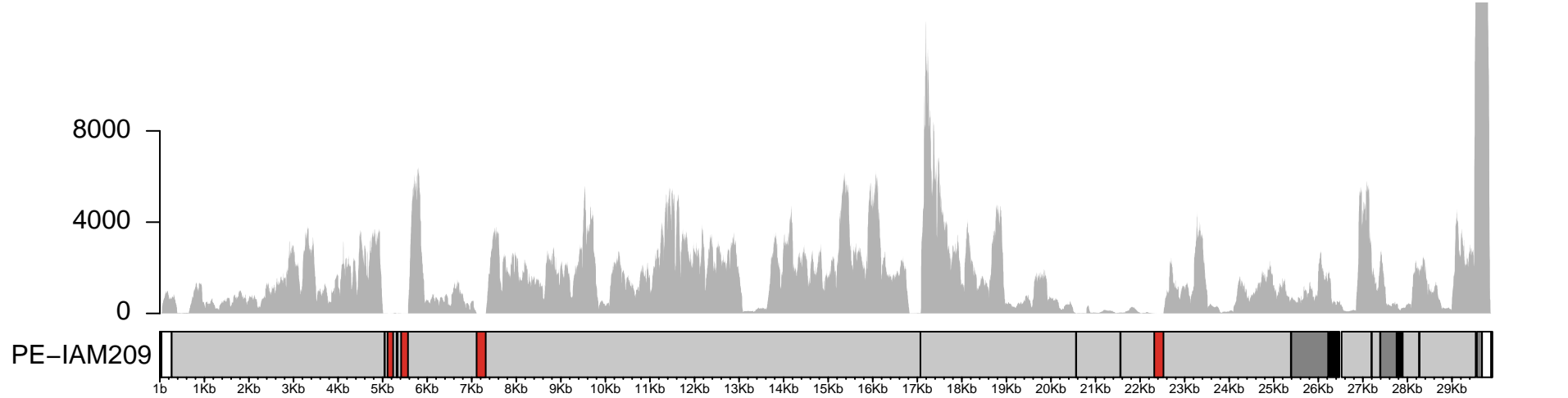

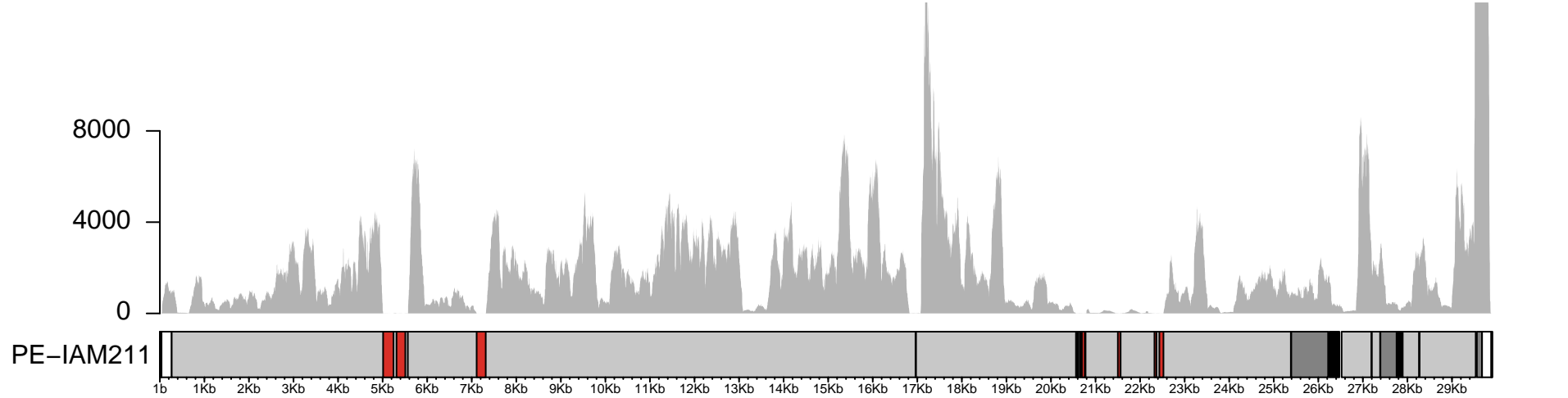

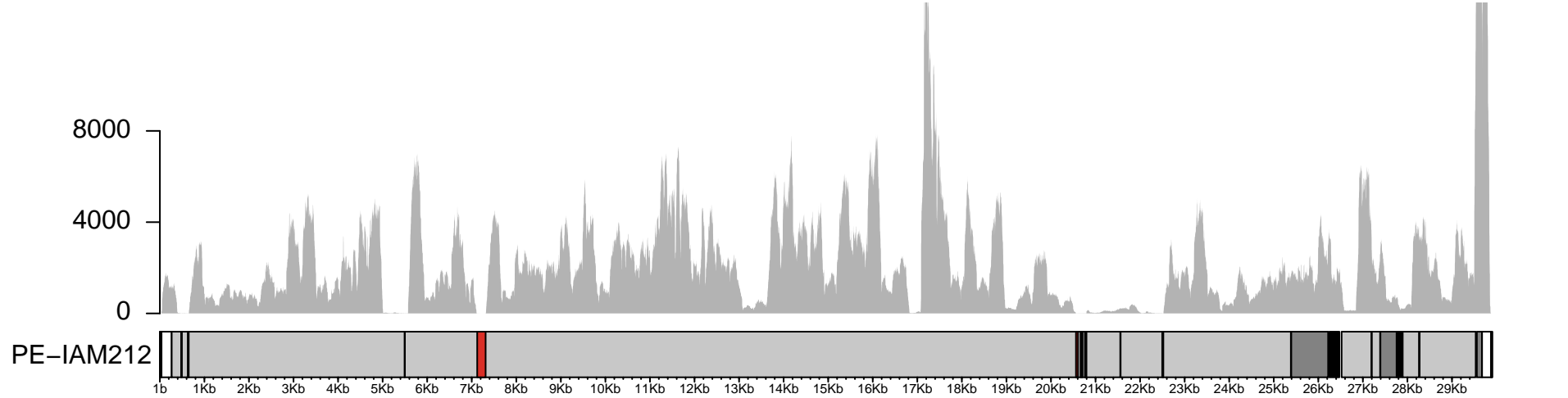

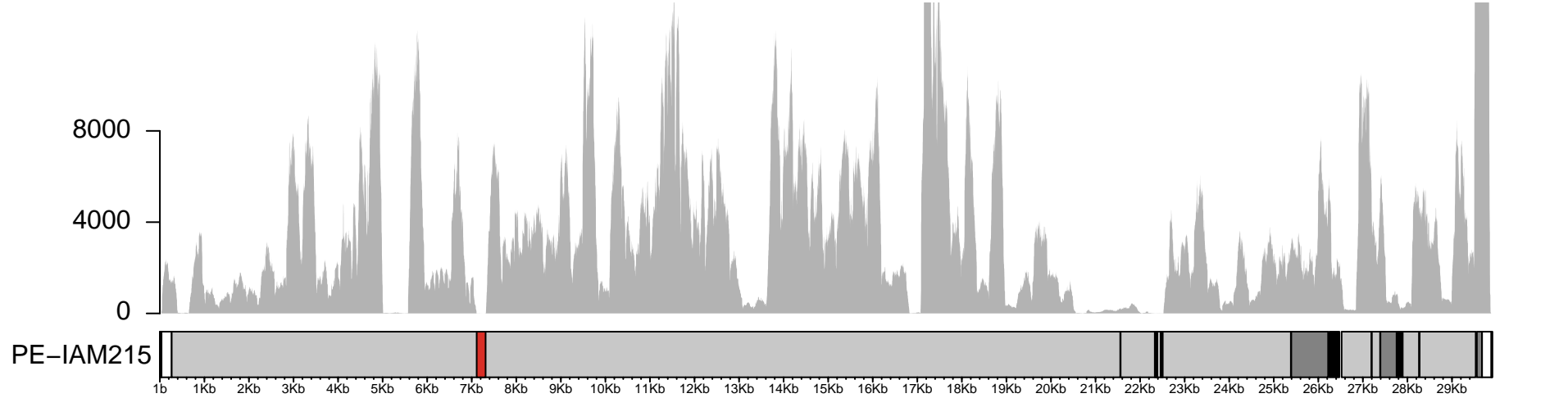

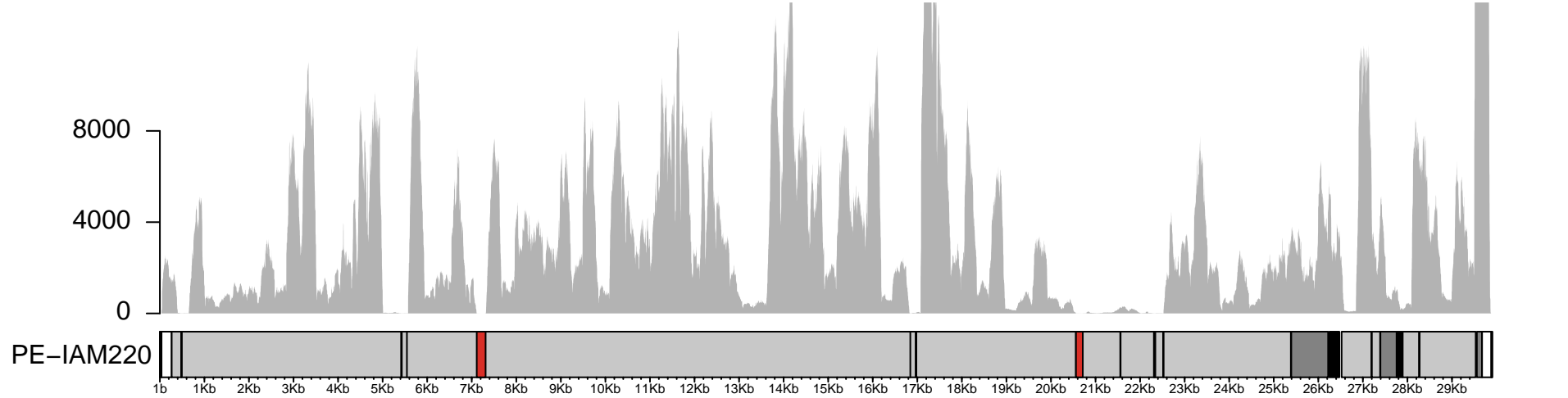

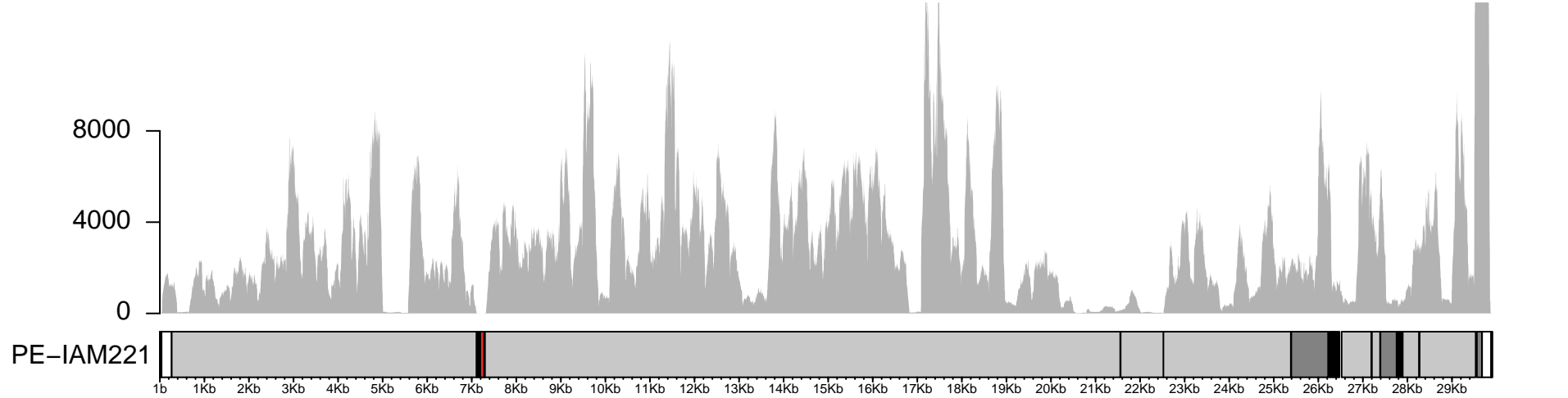

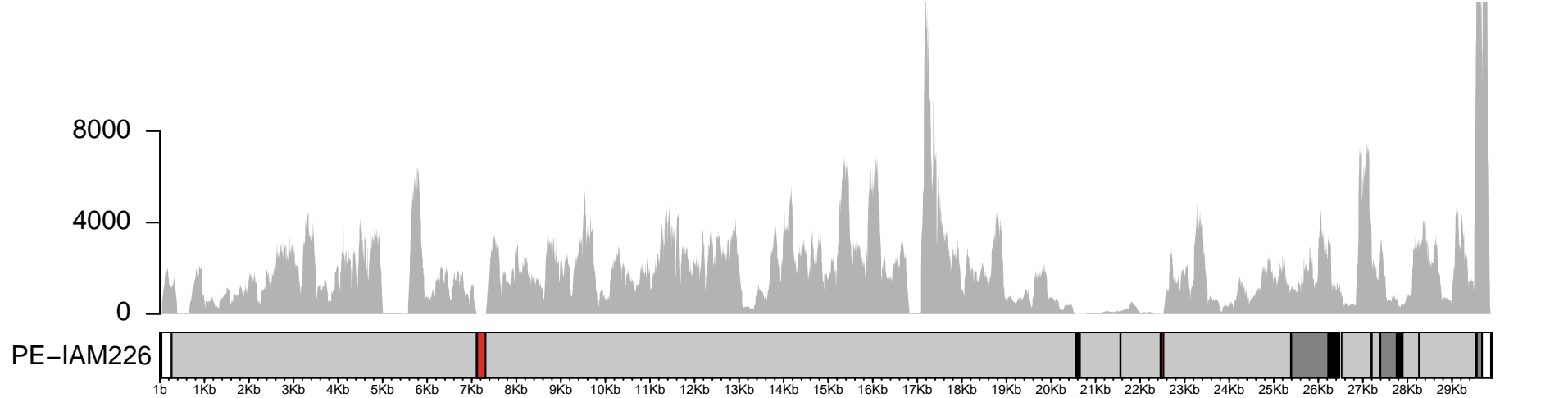

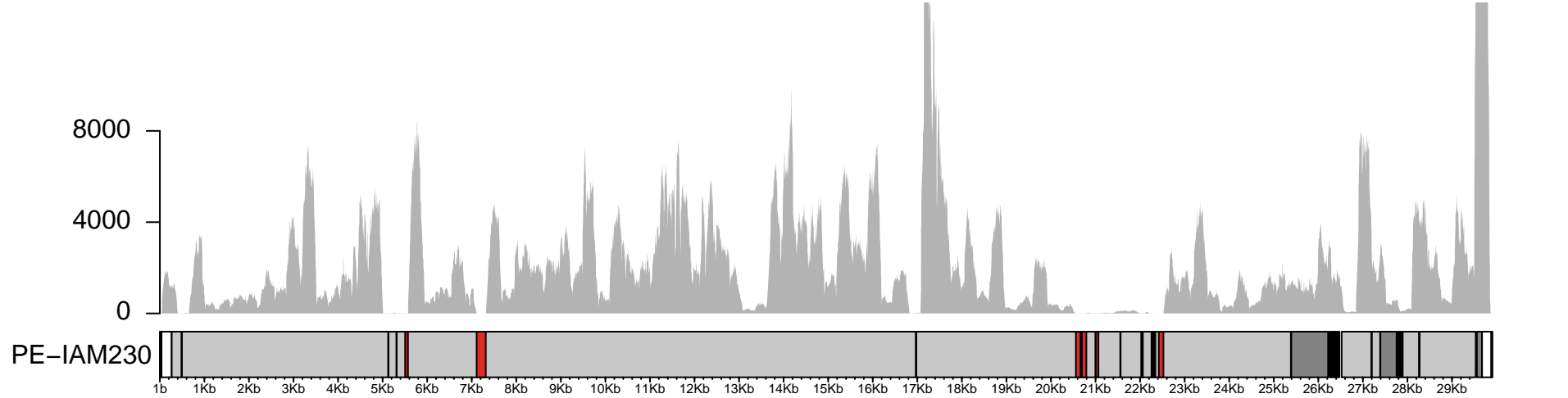

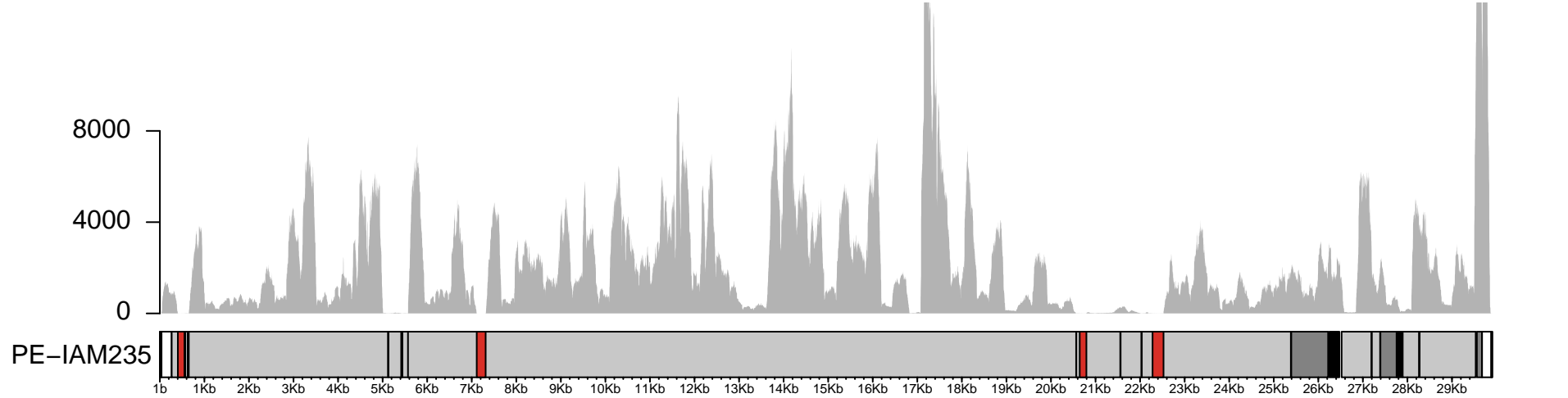

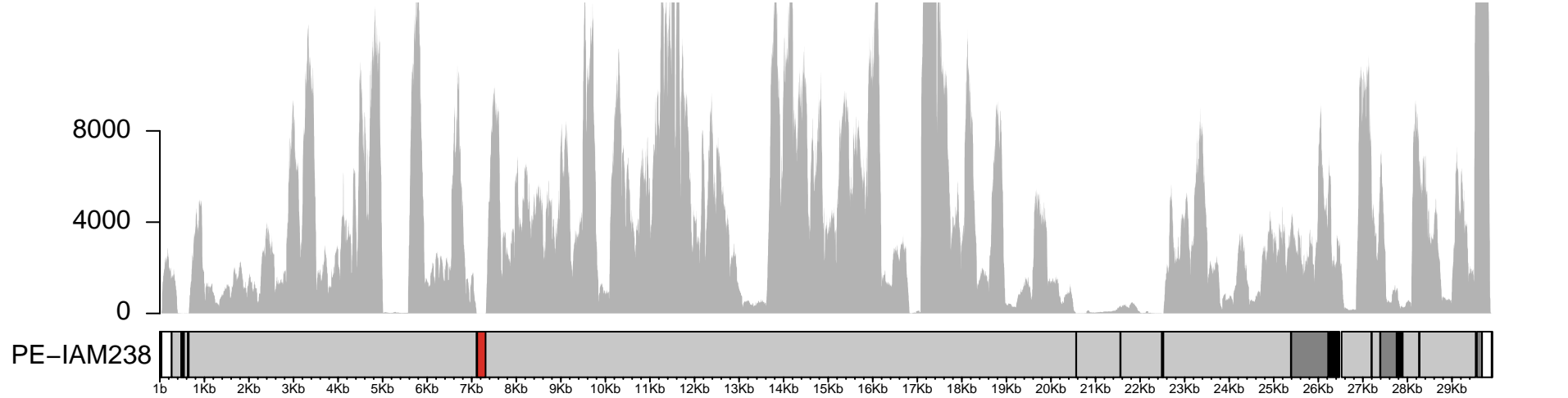

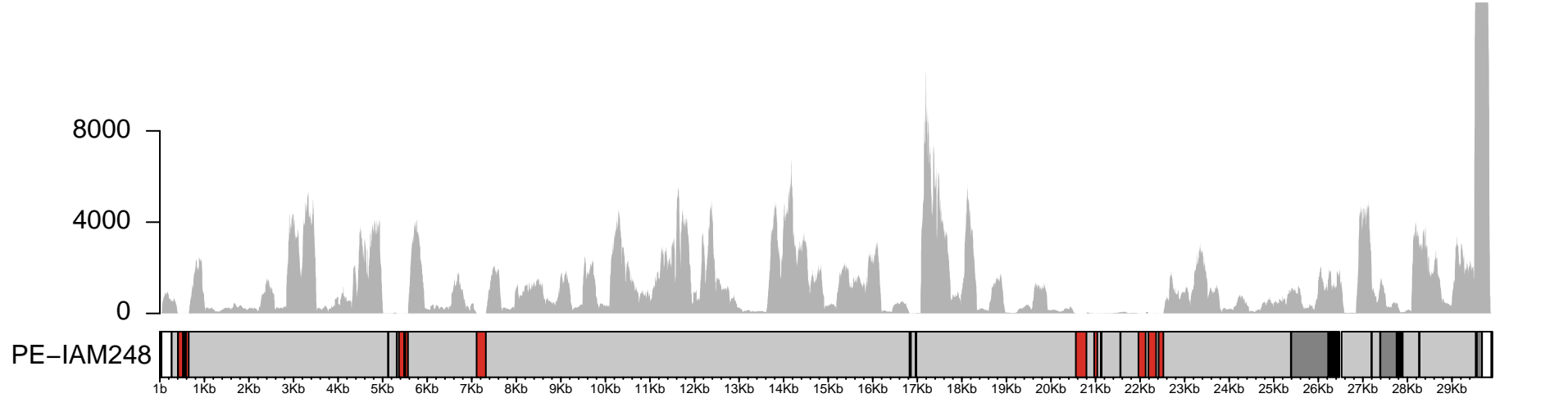

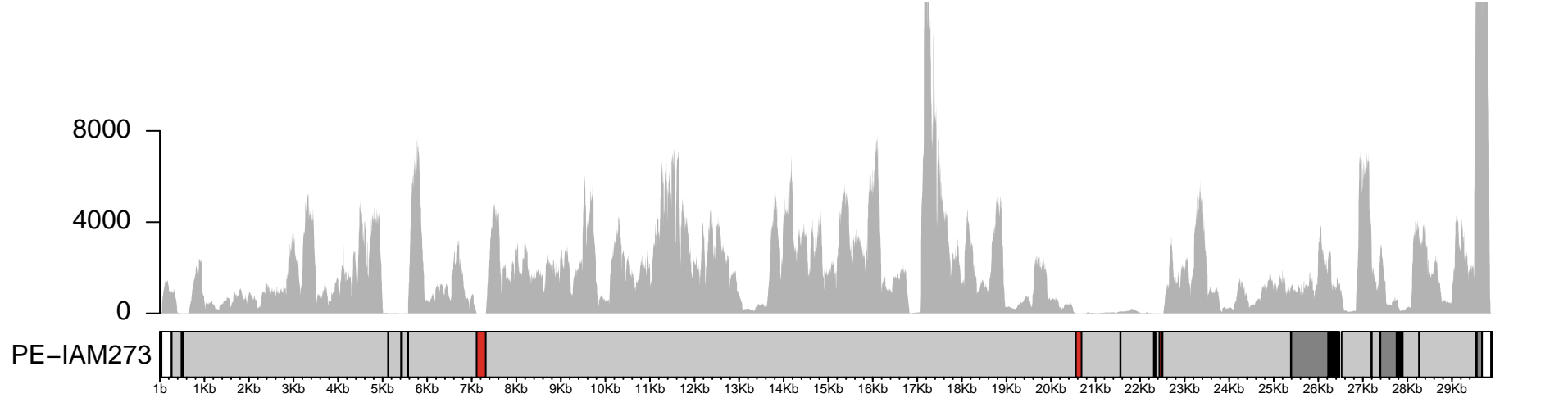

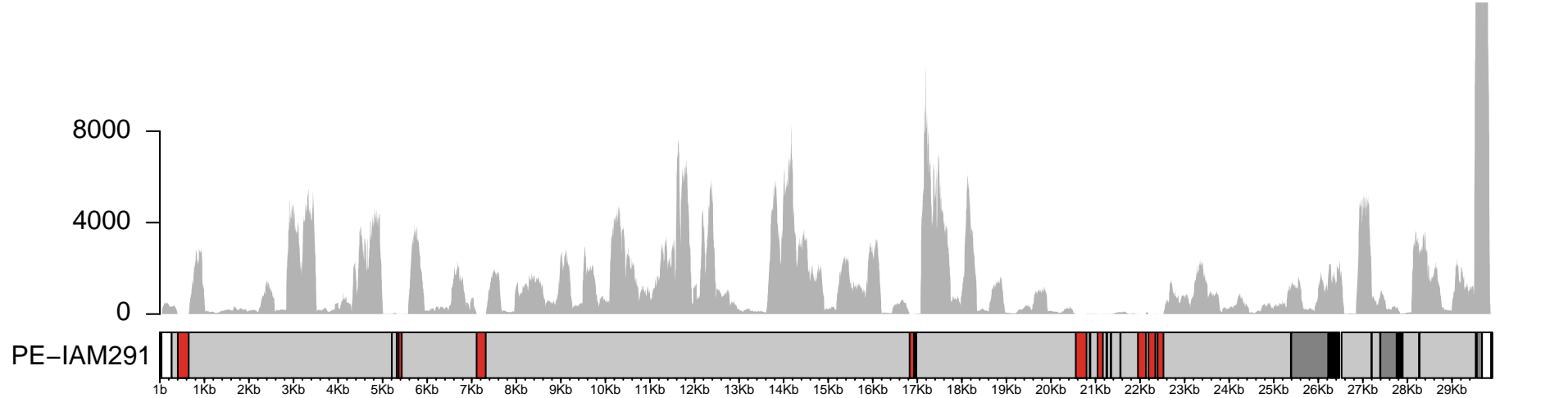

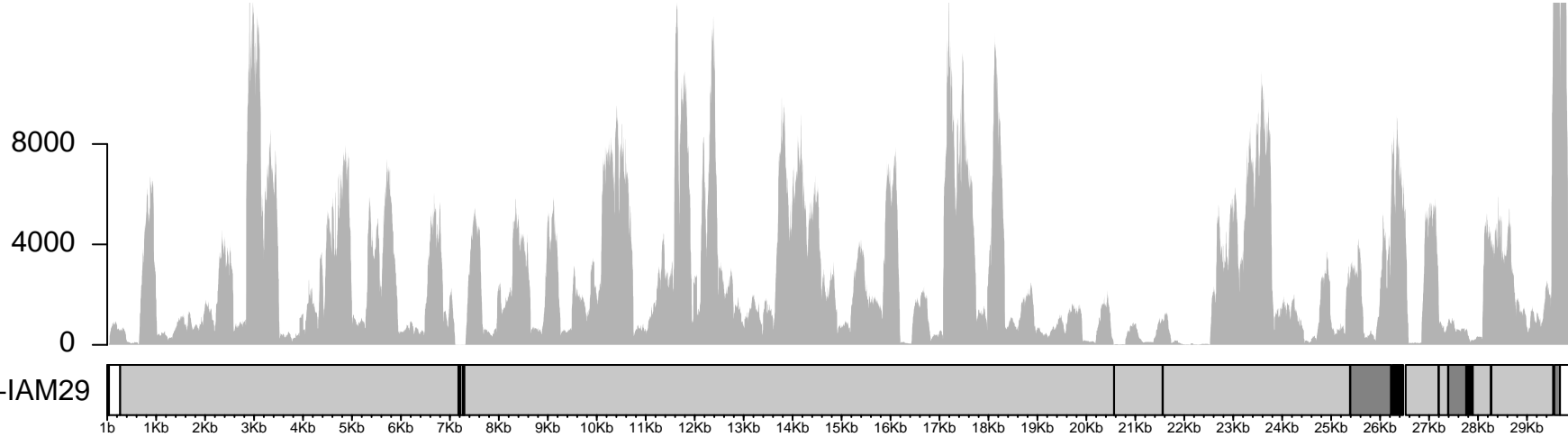

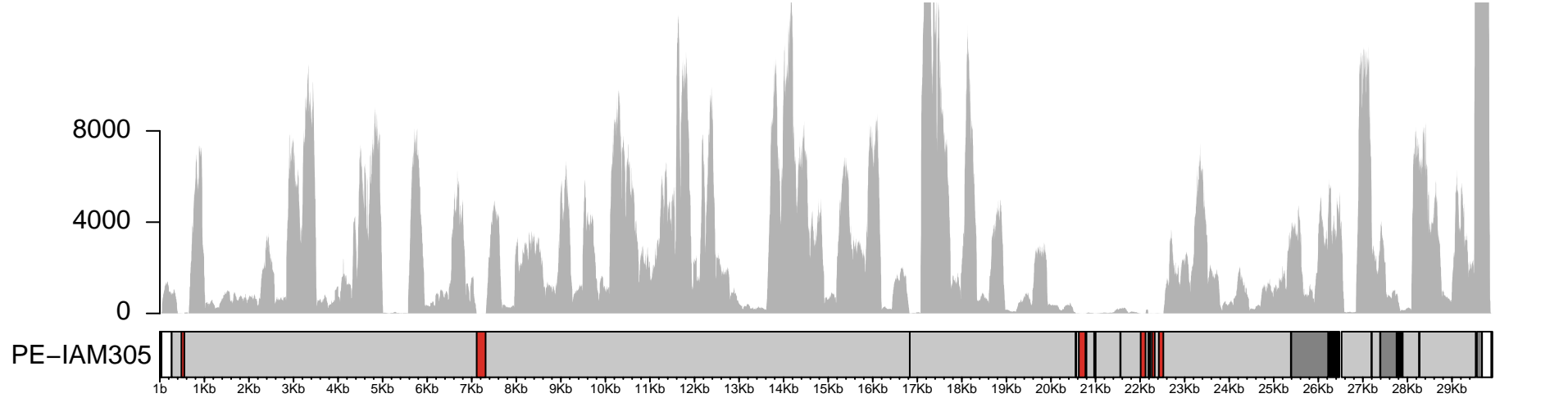

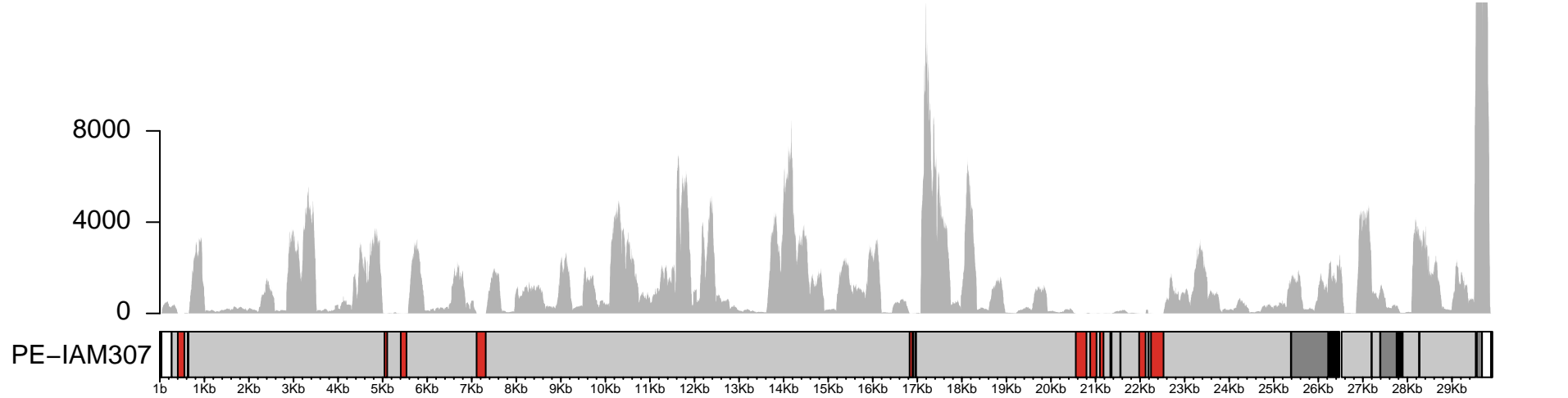

8000  
4000  
0

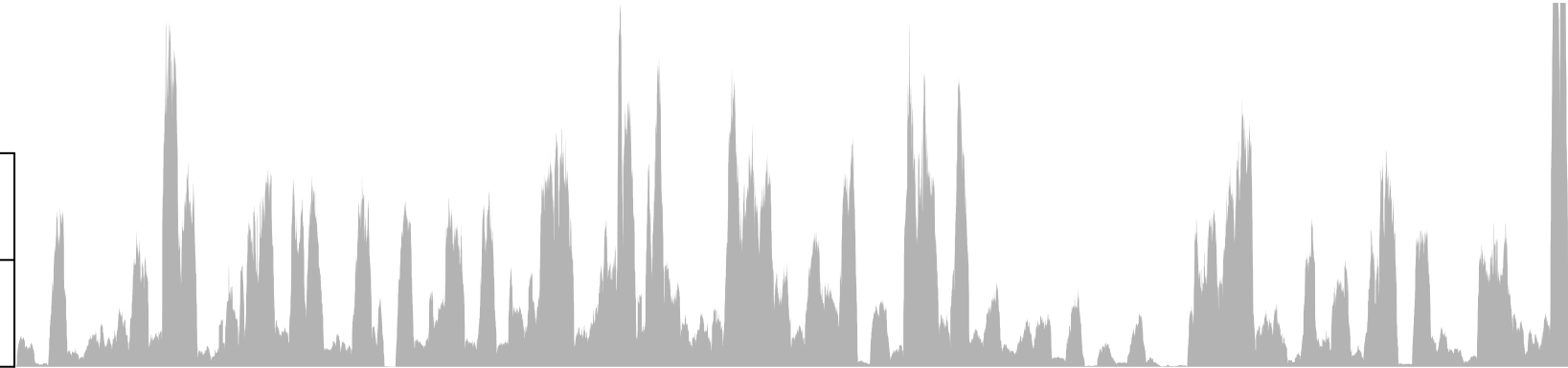

PE-IAM30

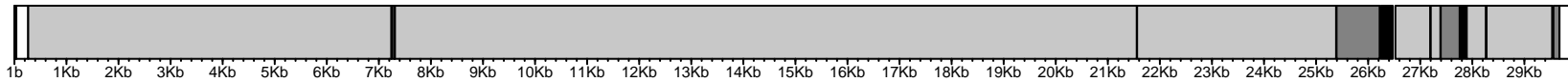

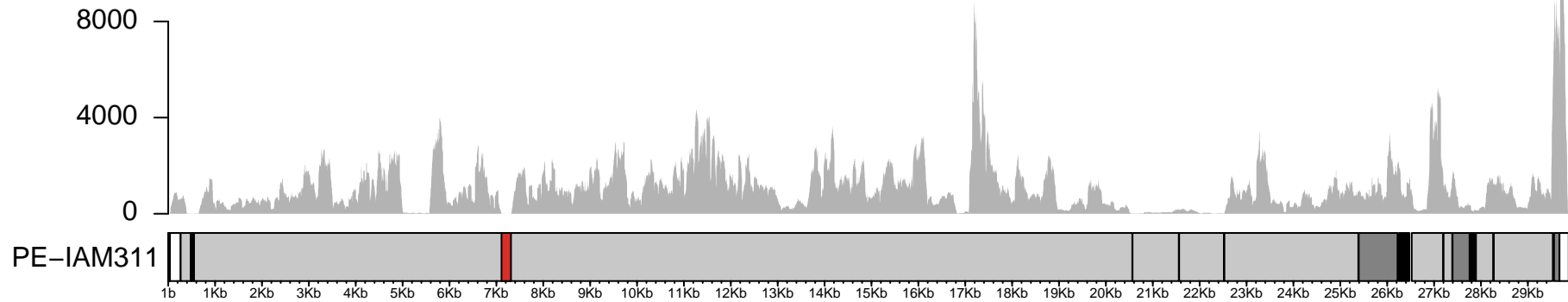

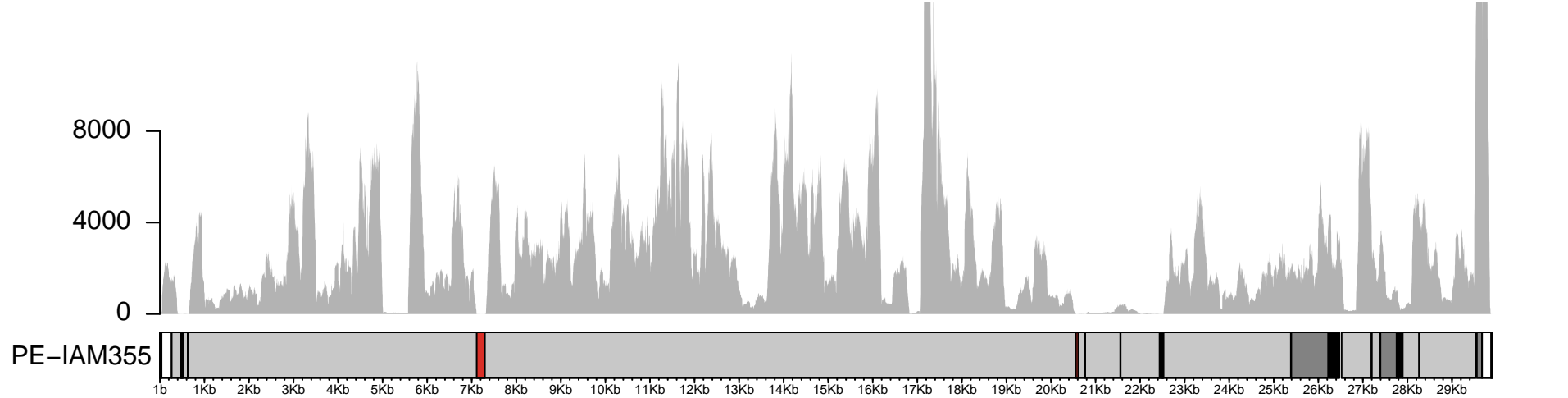

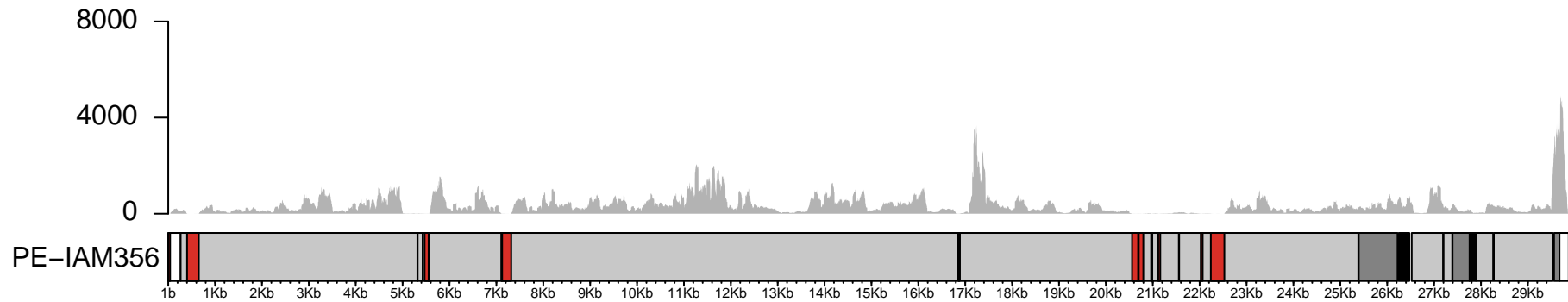

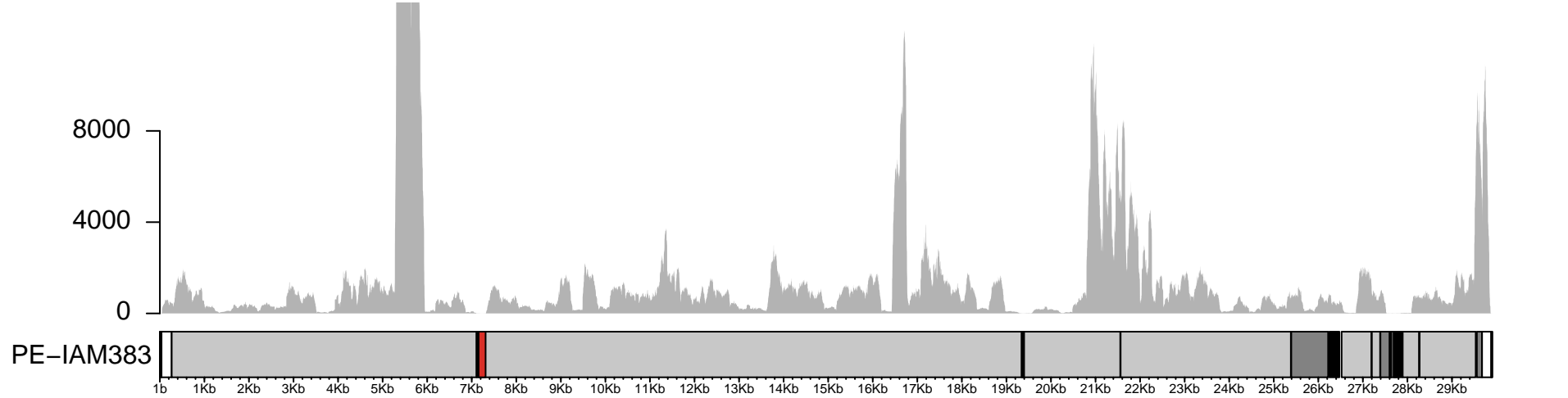

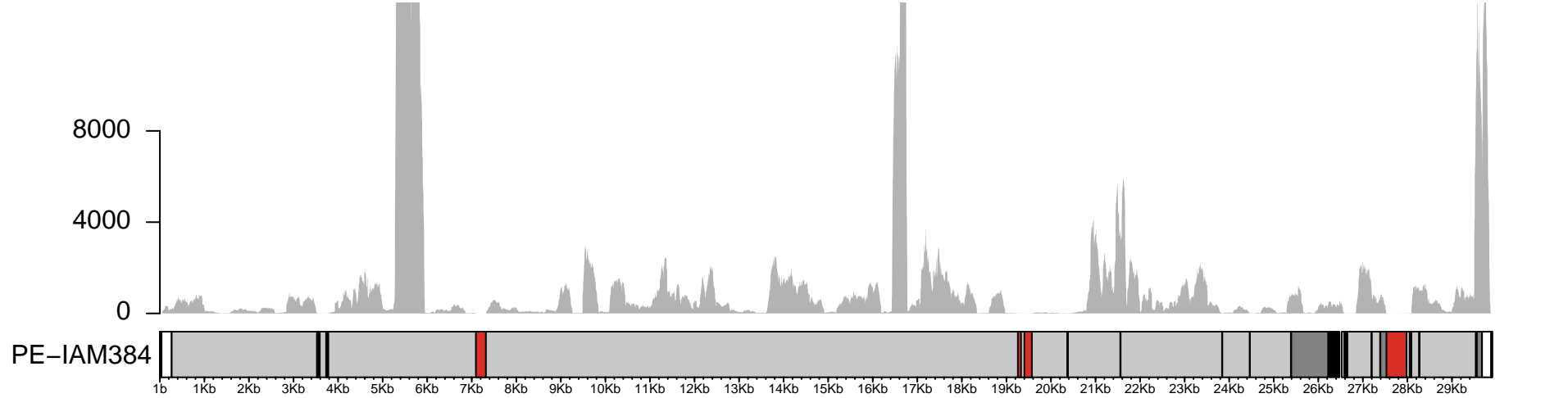

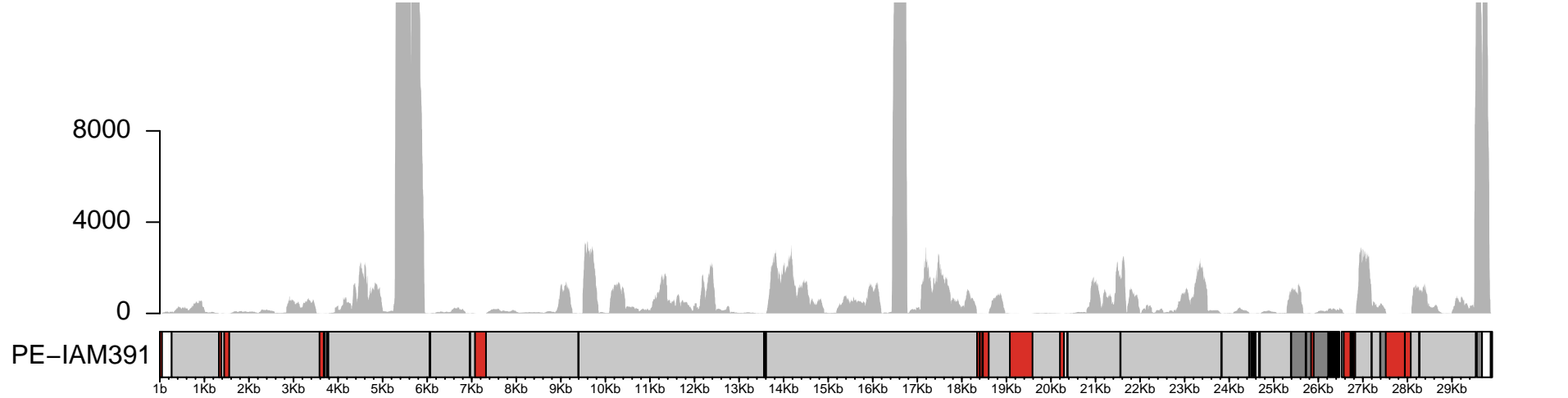

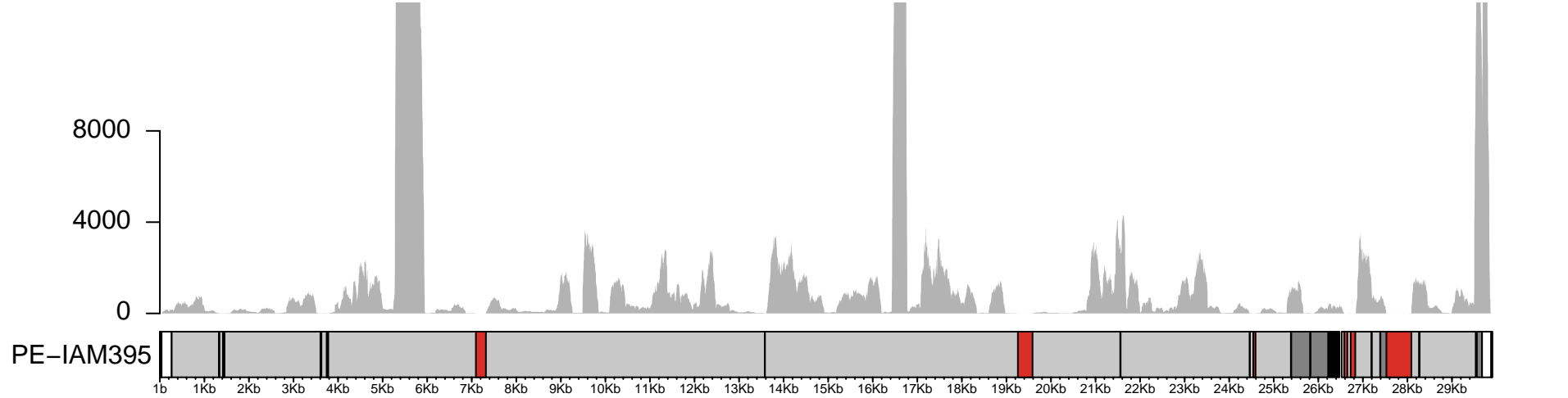

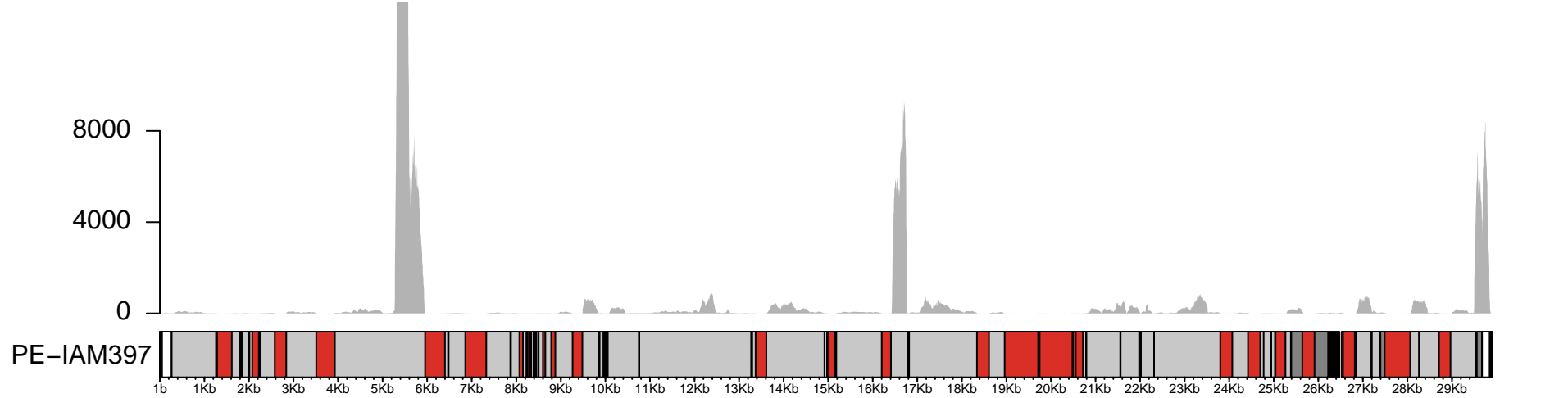

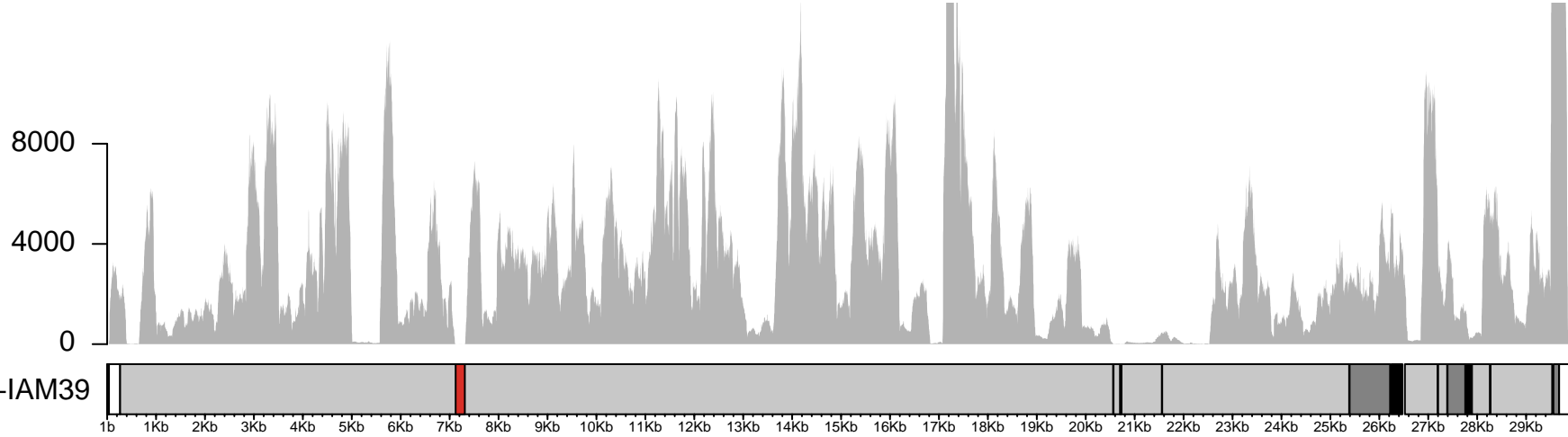

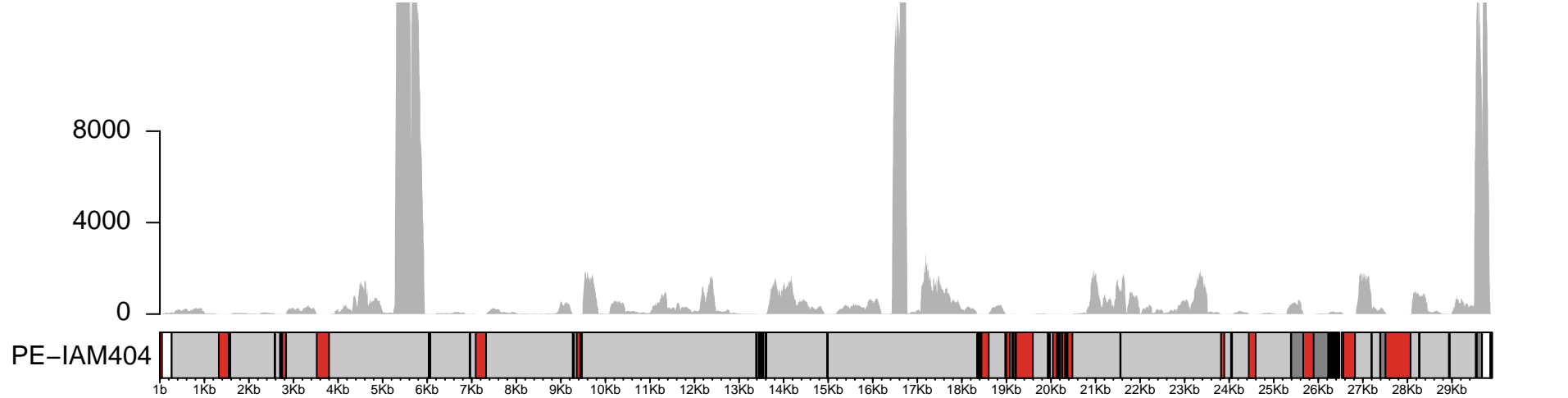

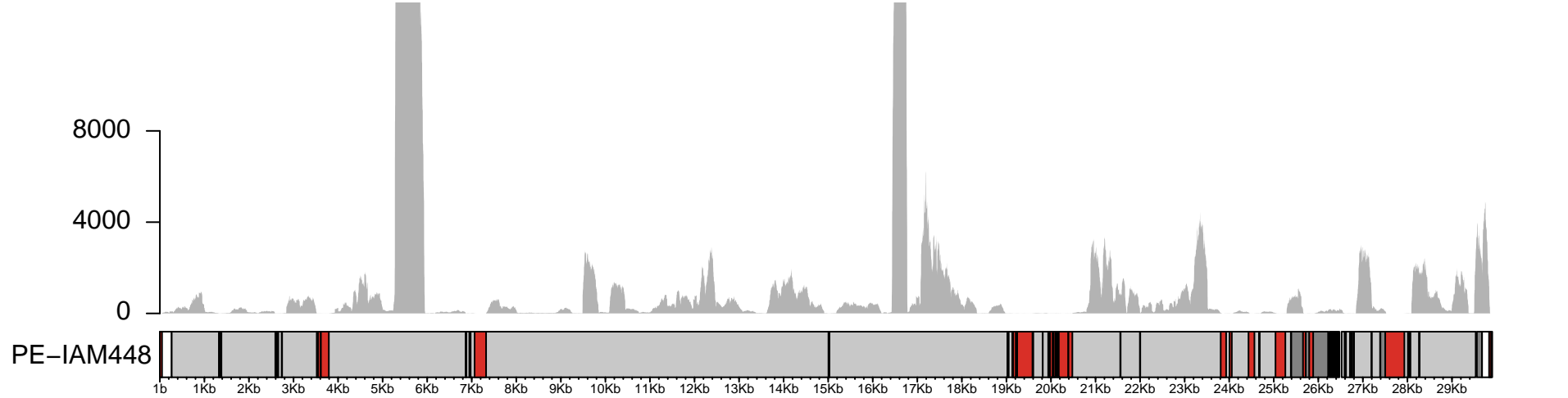

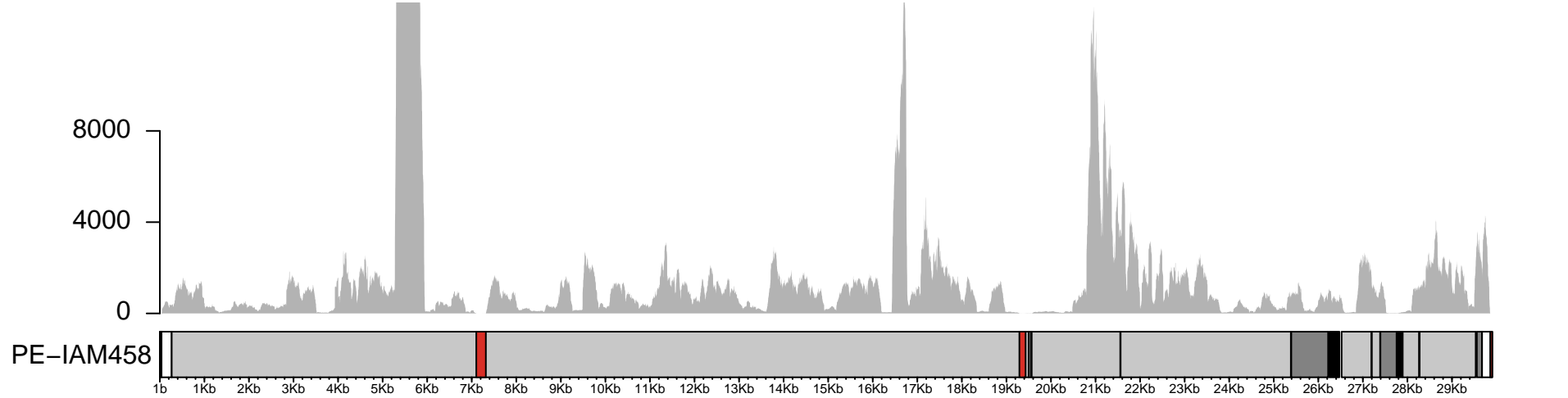

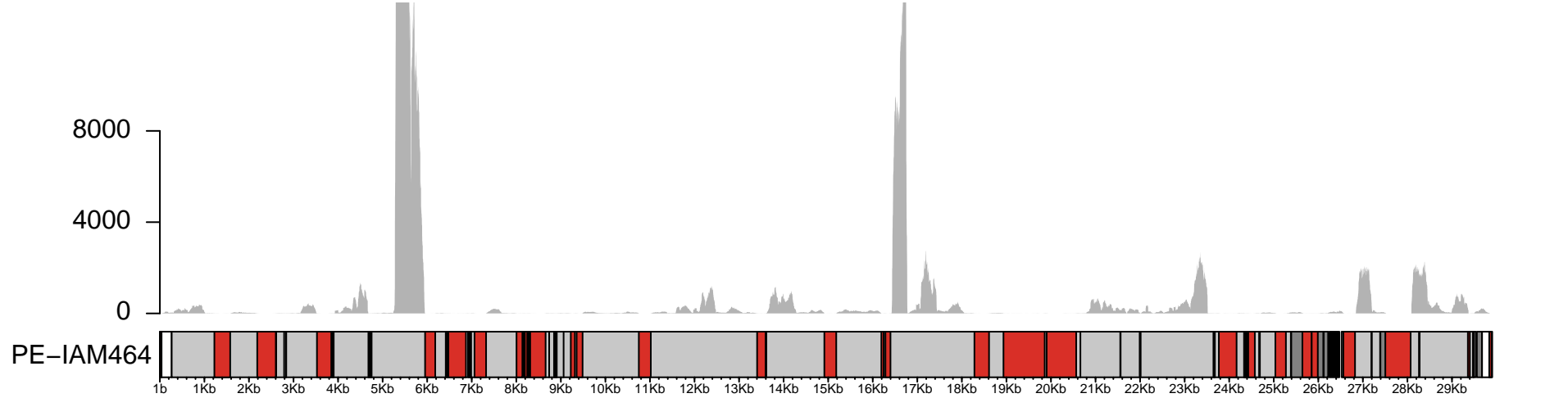

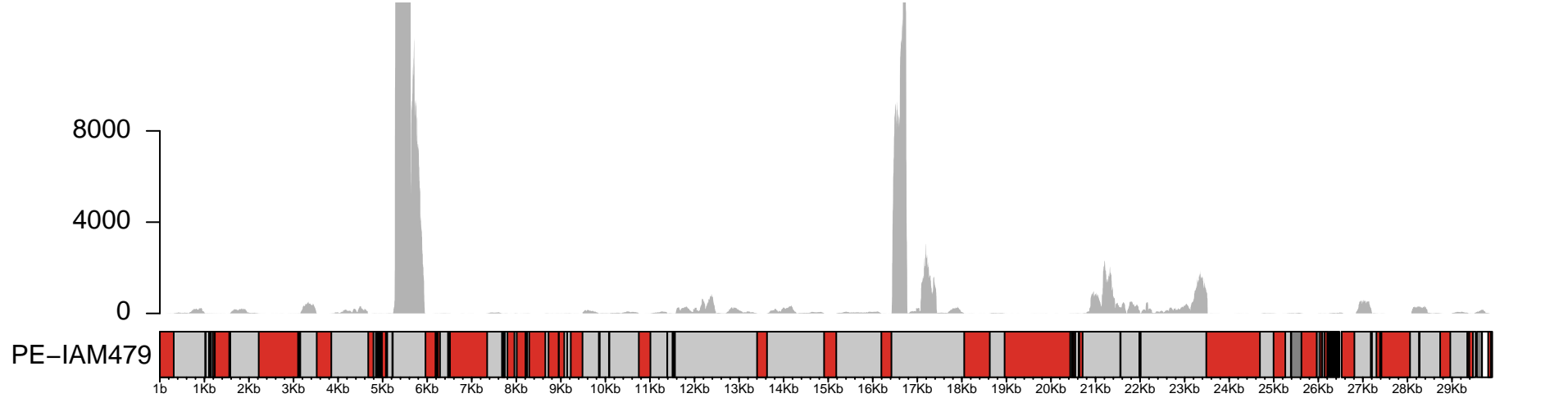

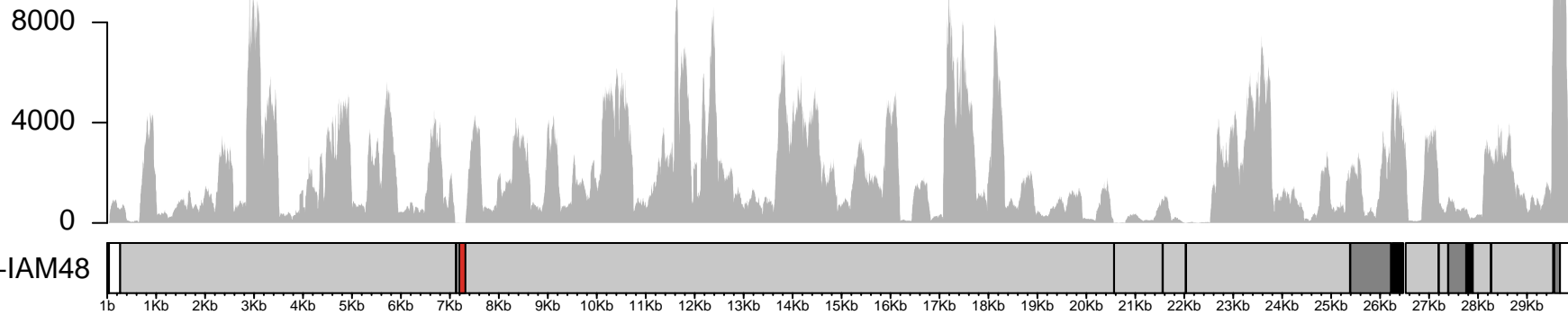

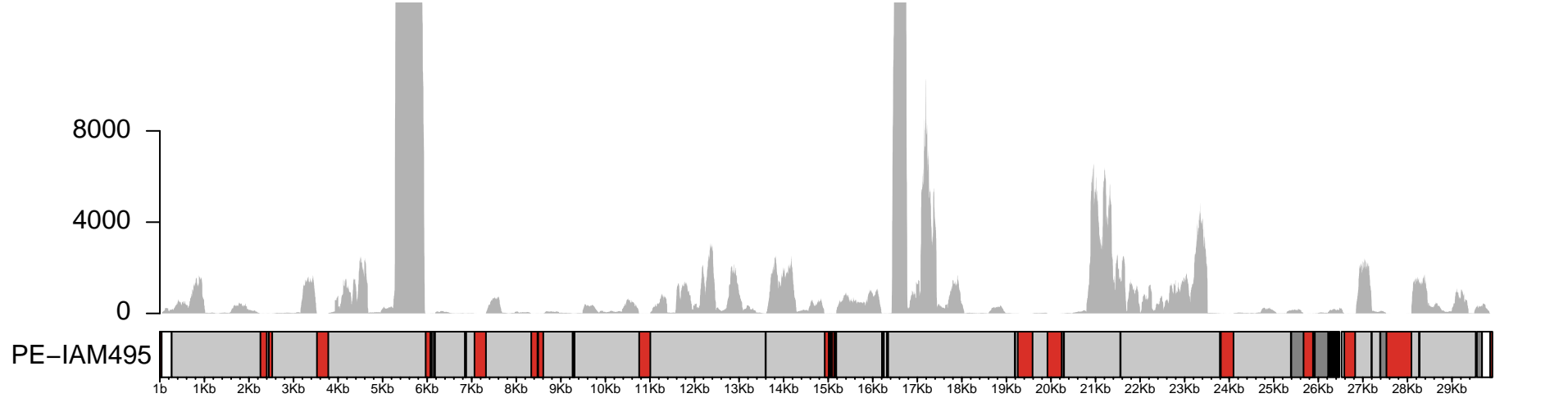

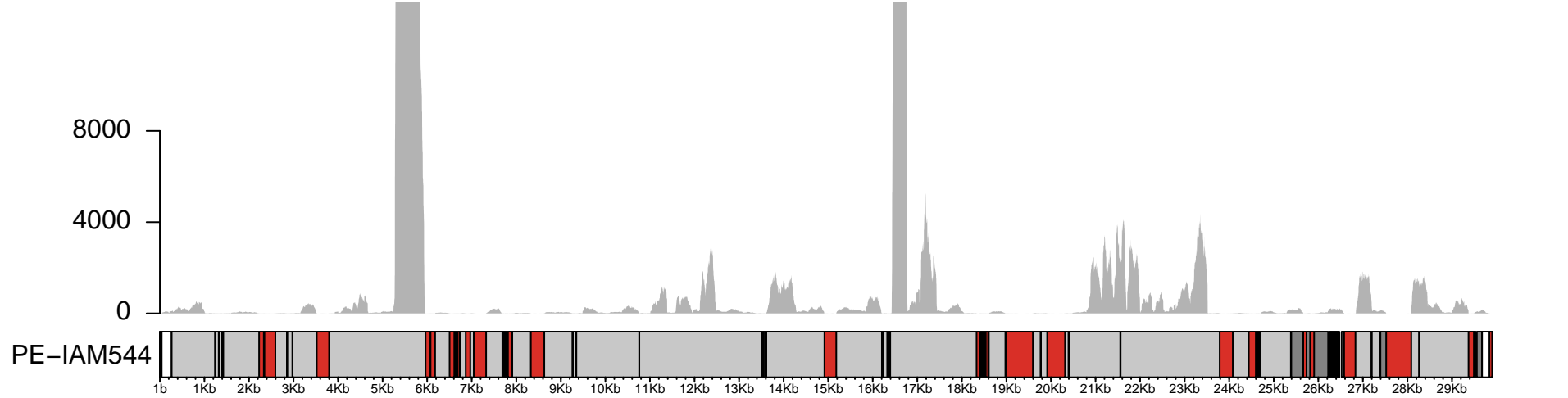

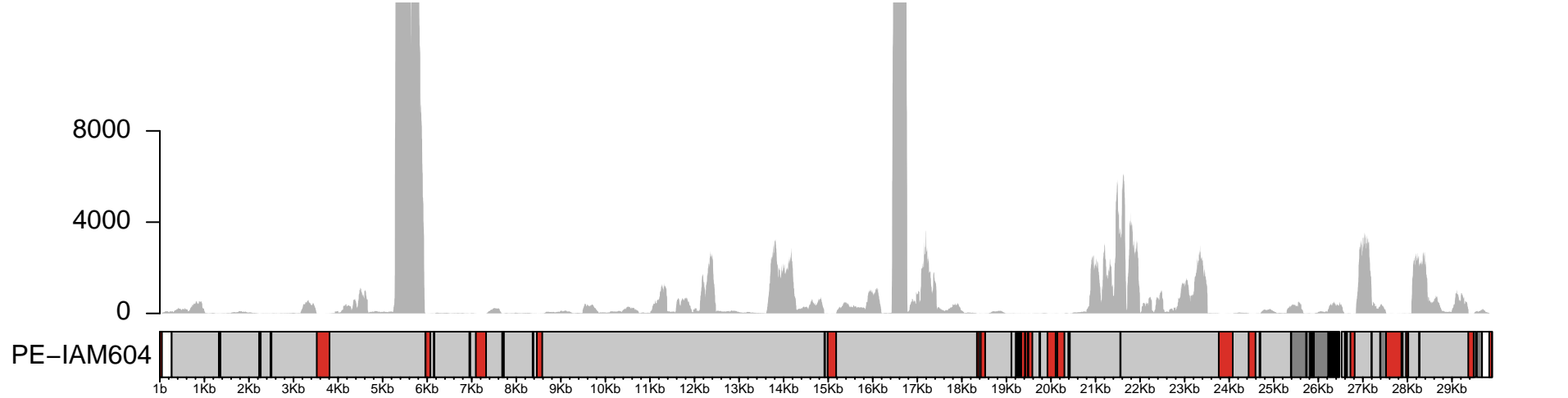

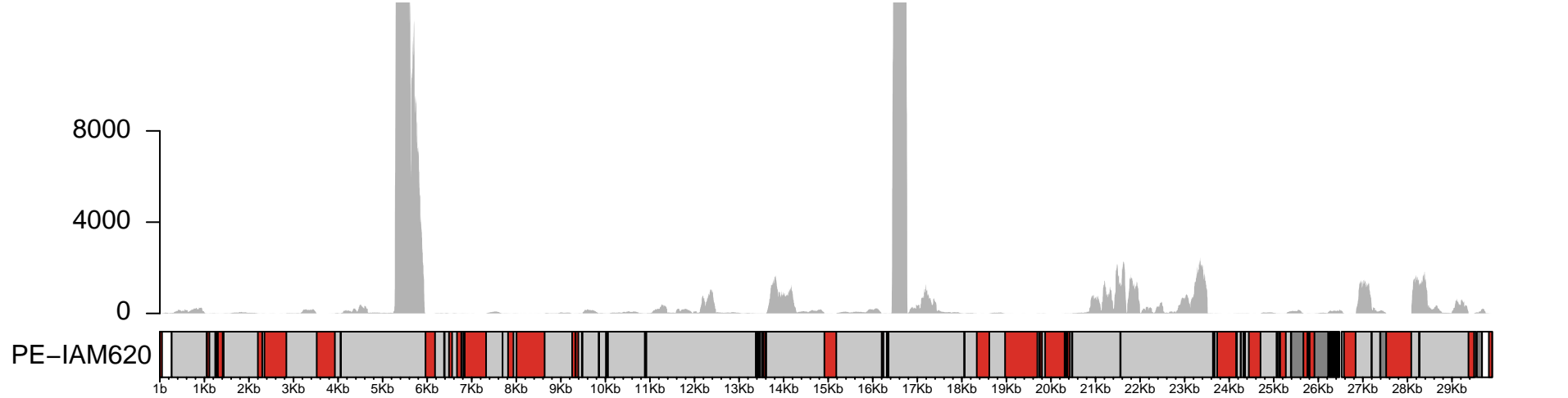

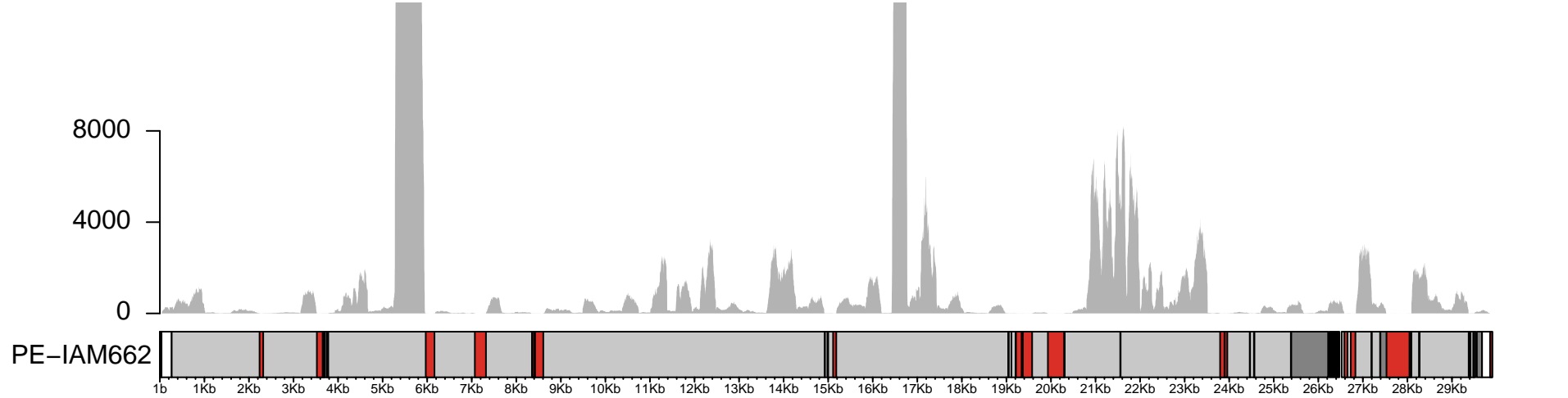

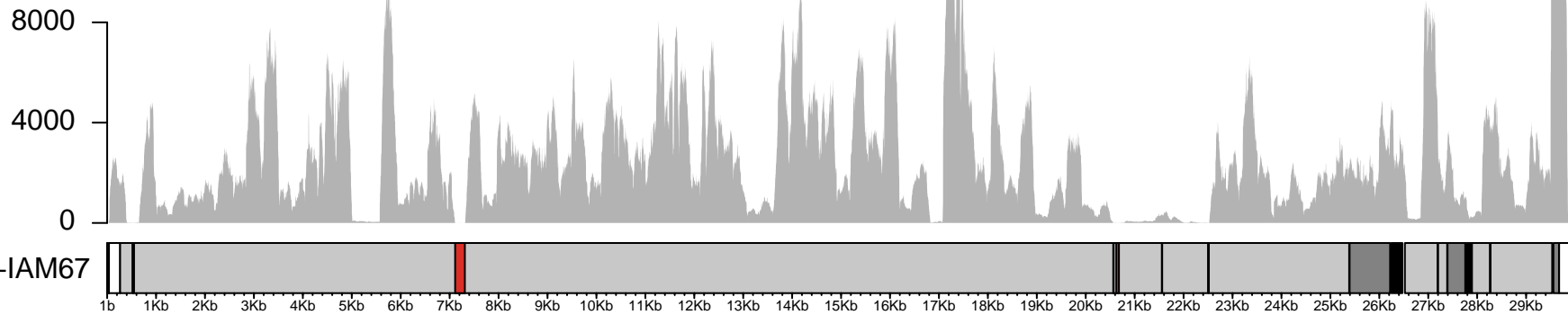

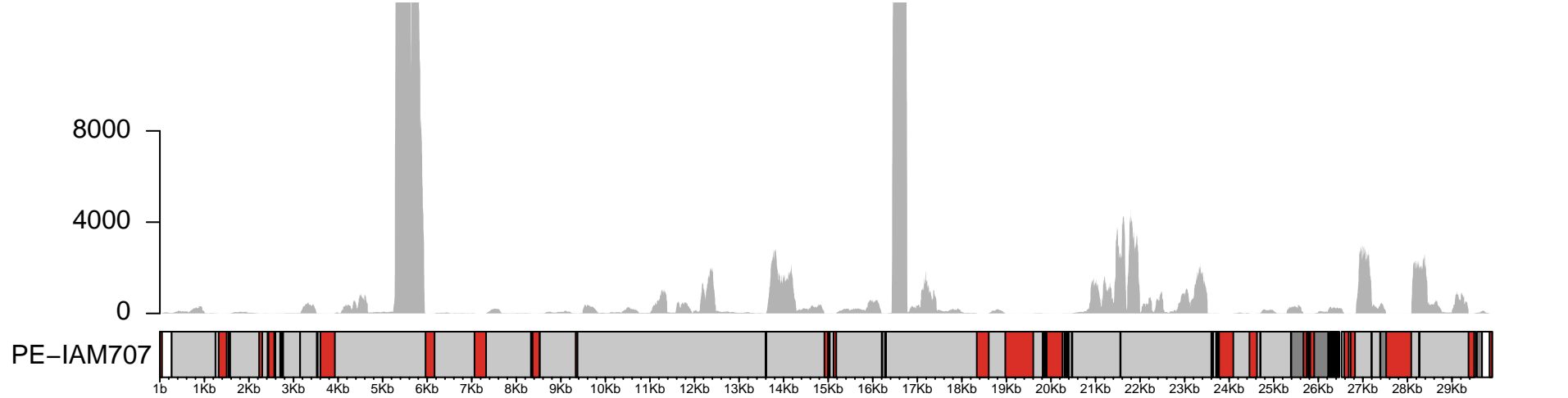

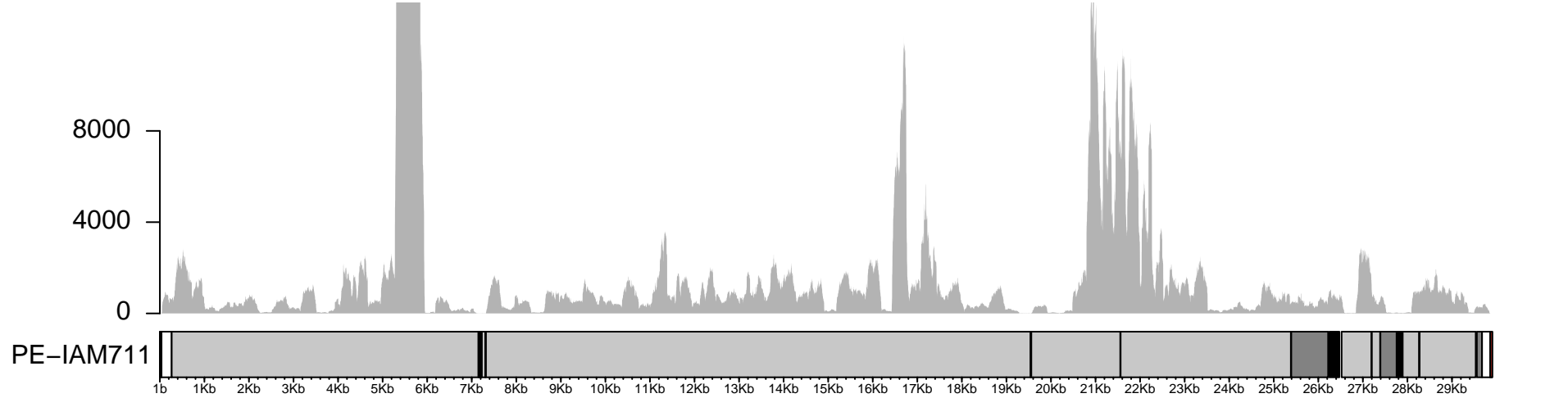

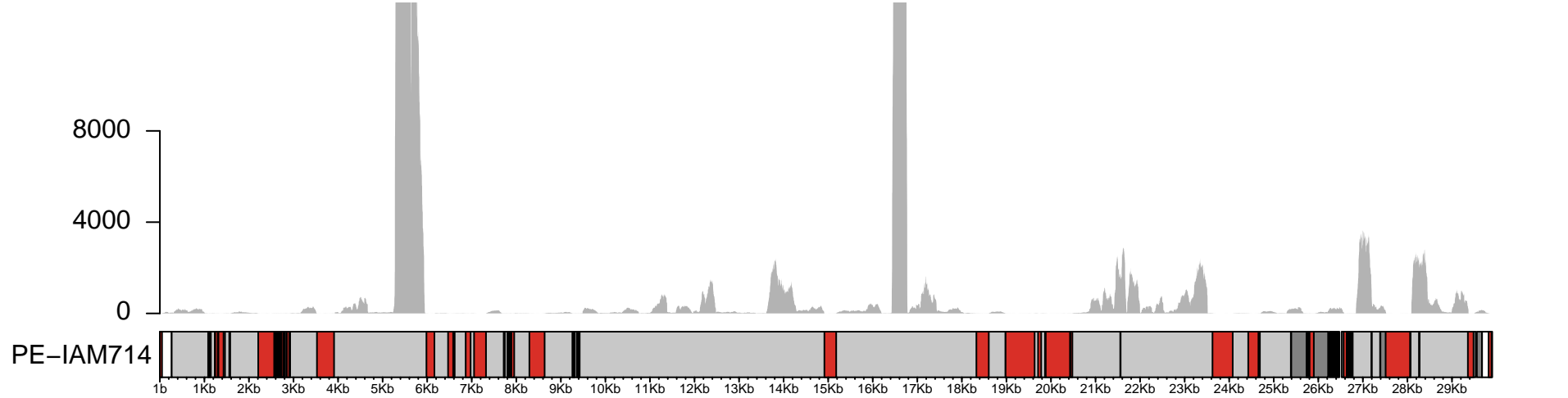

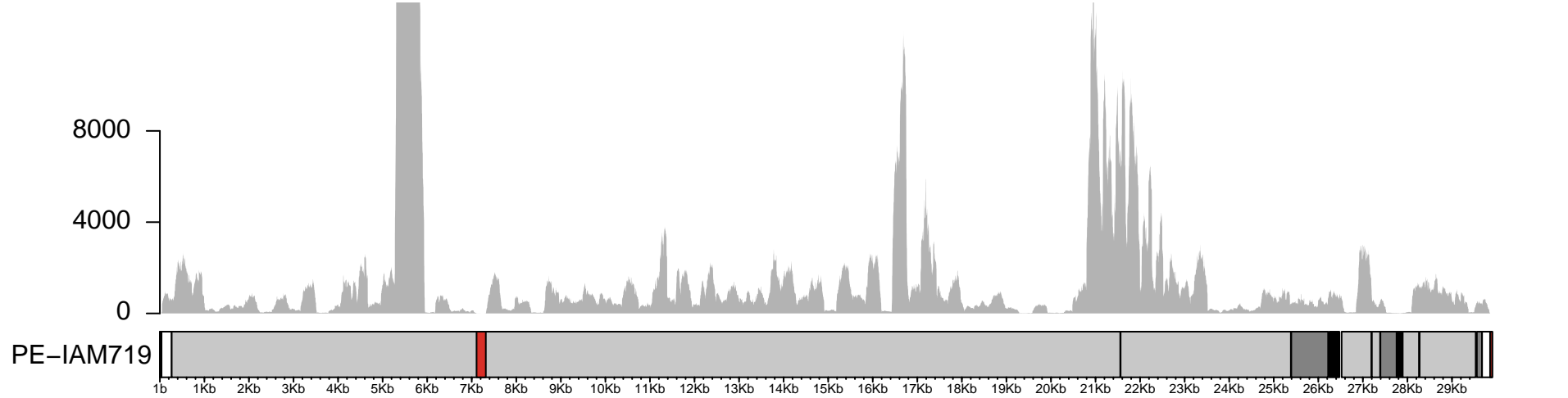

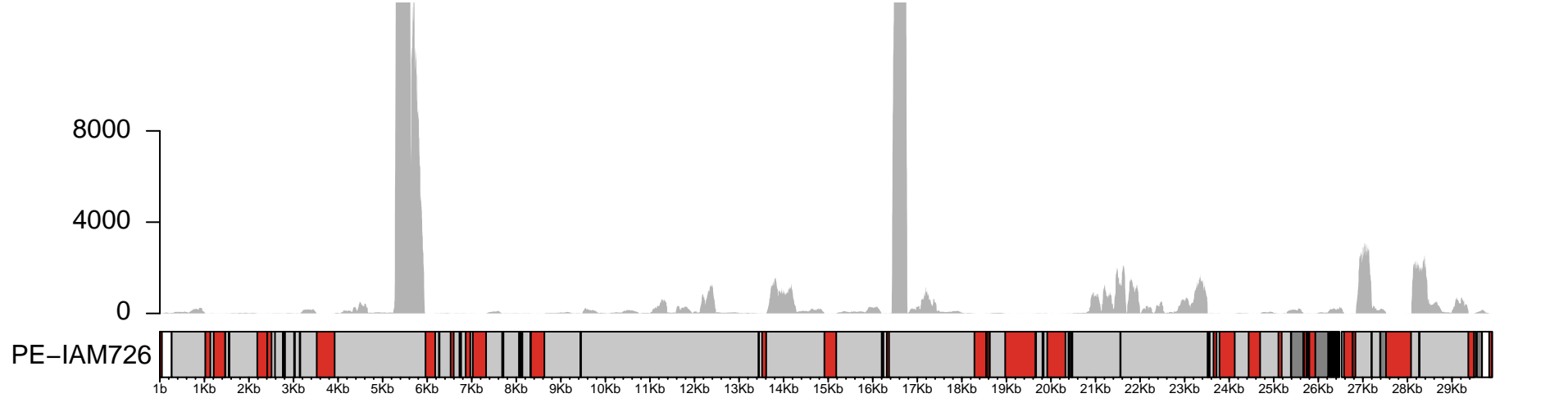

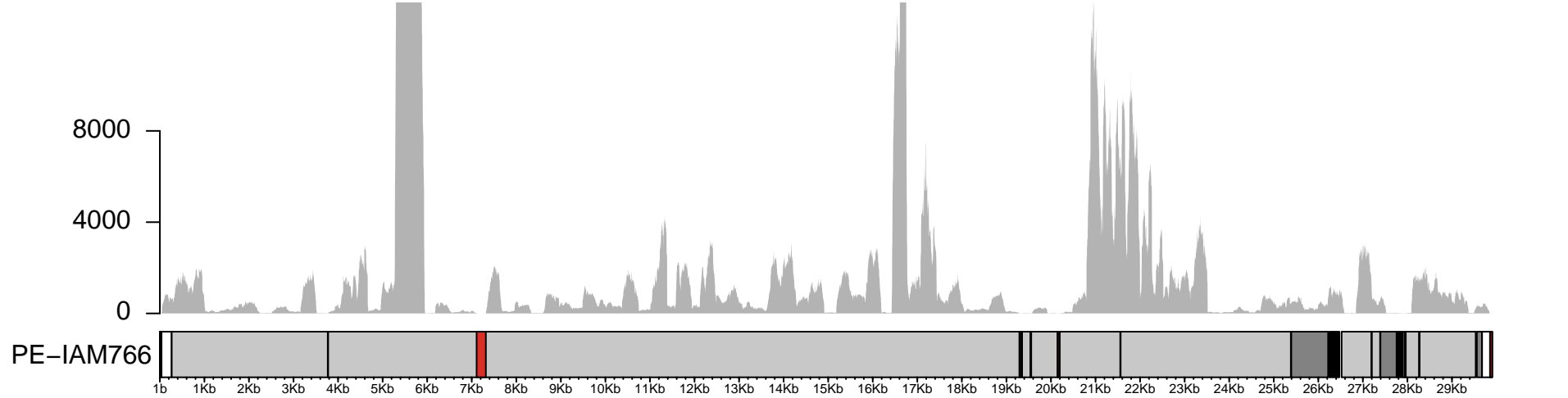

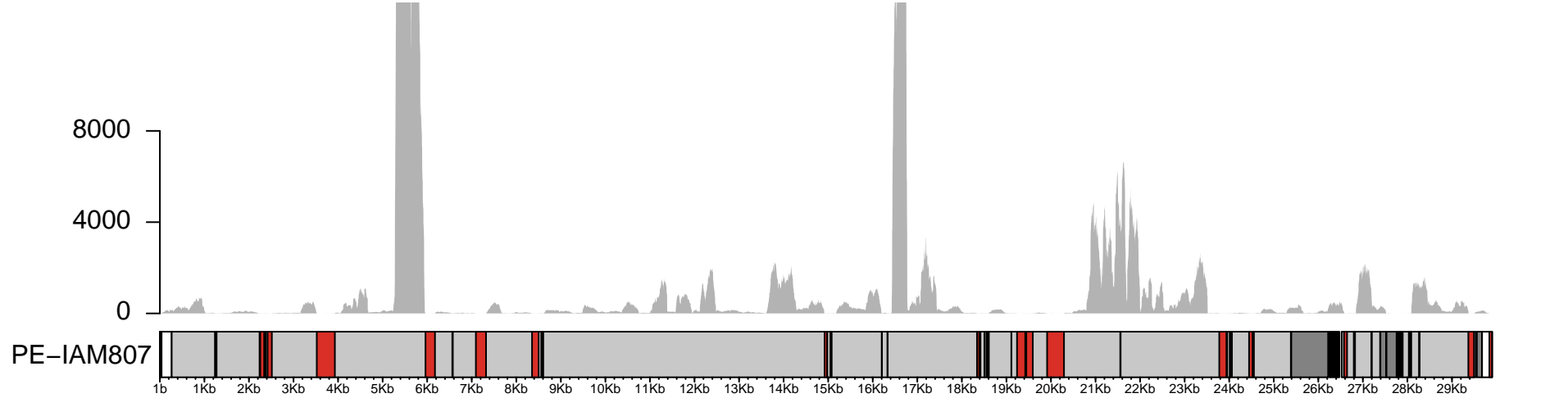

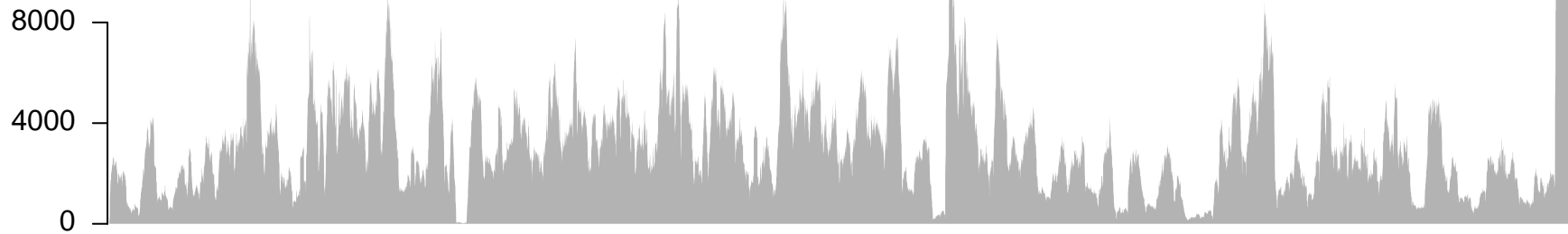

PE-IAM84

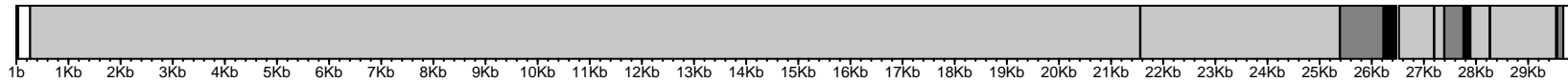

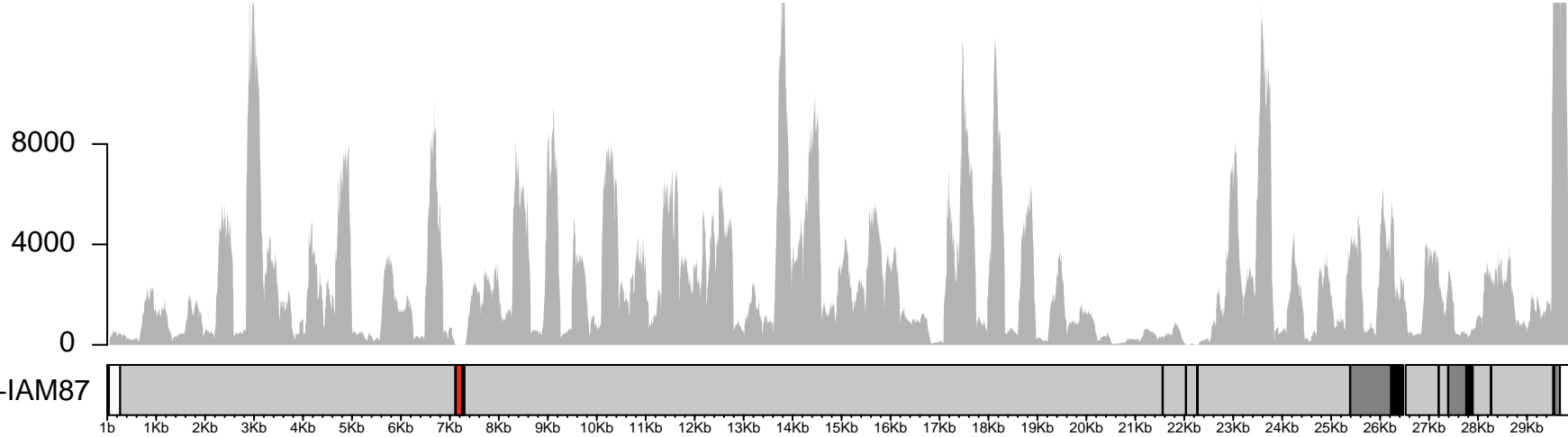

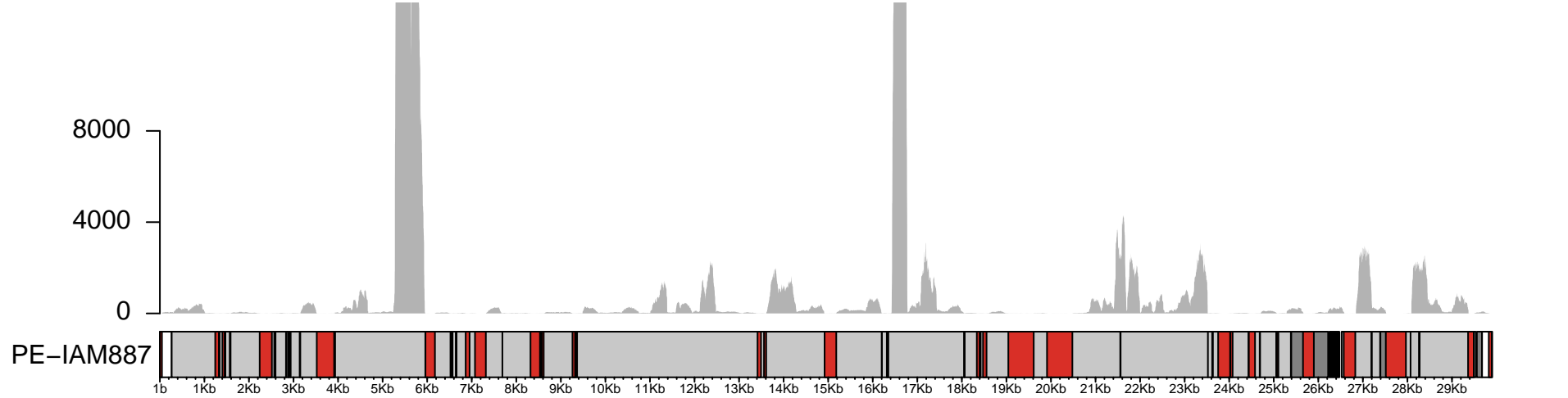

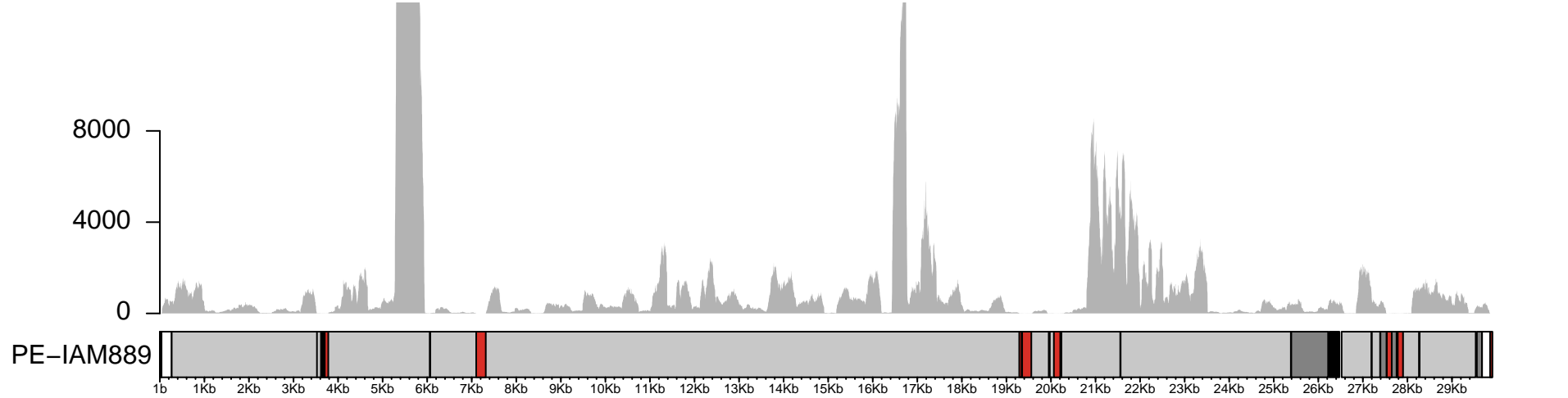

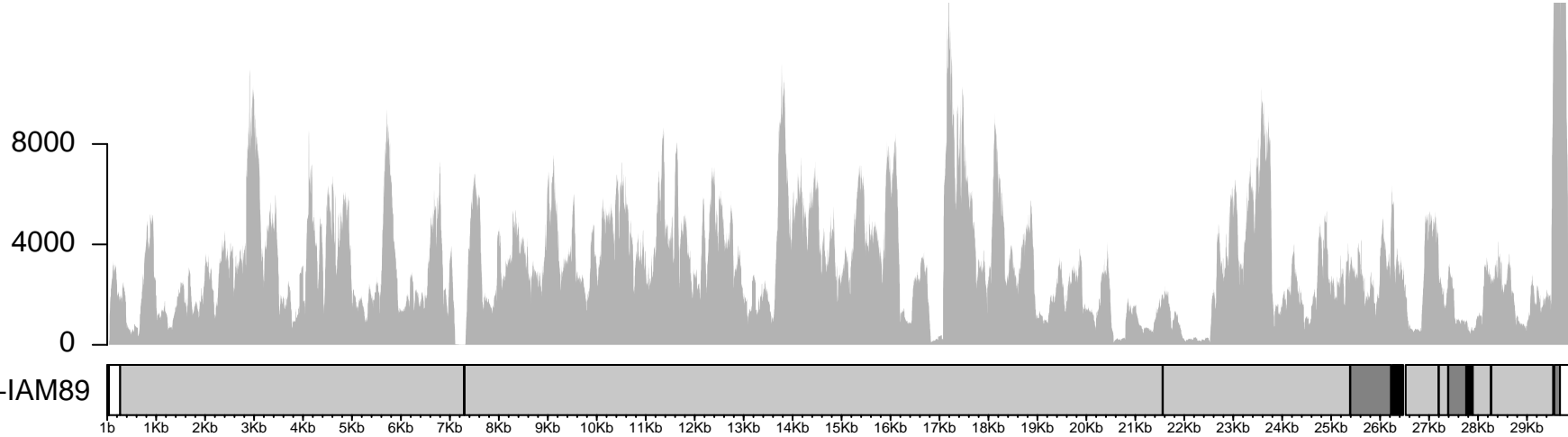

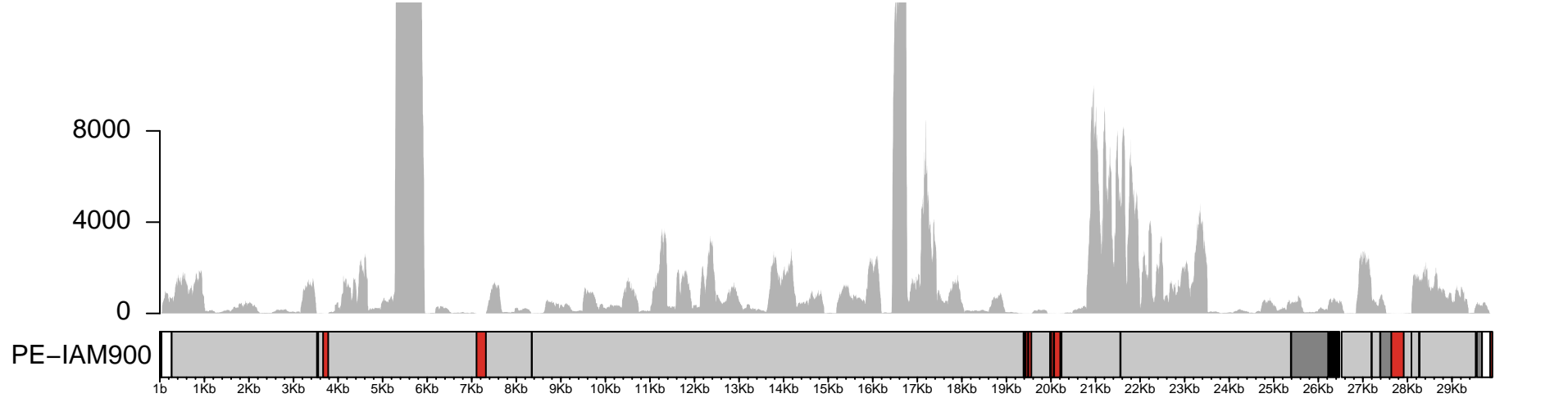

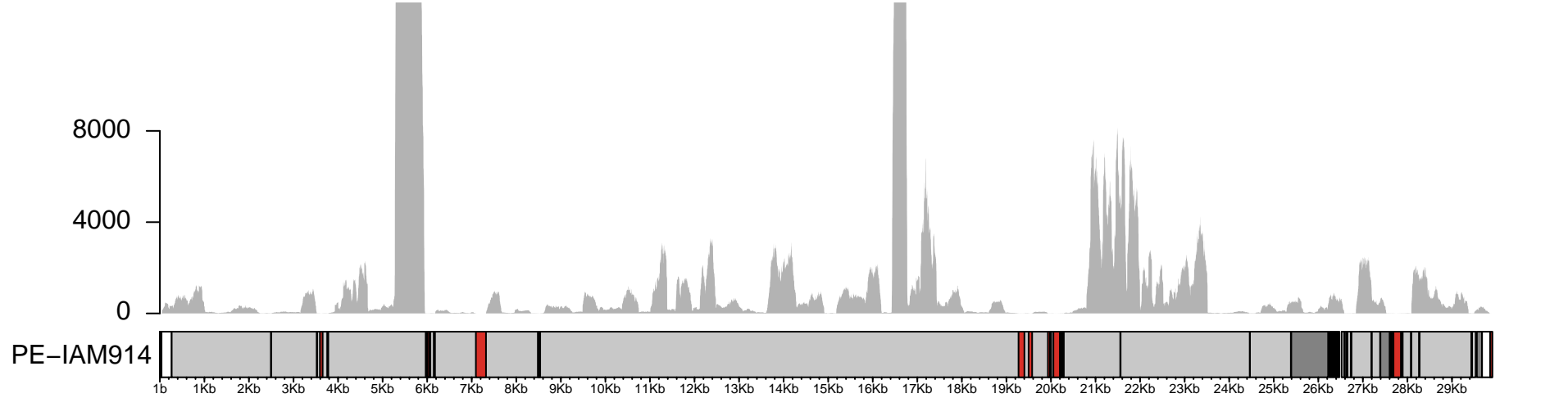

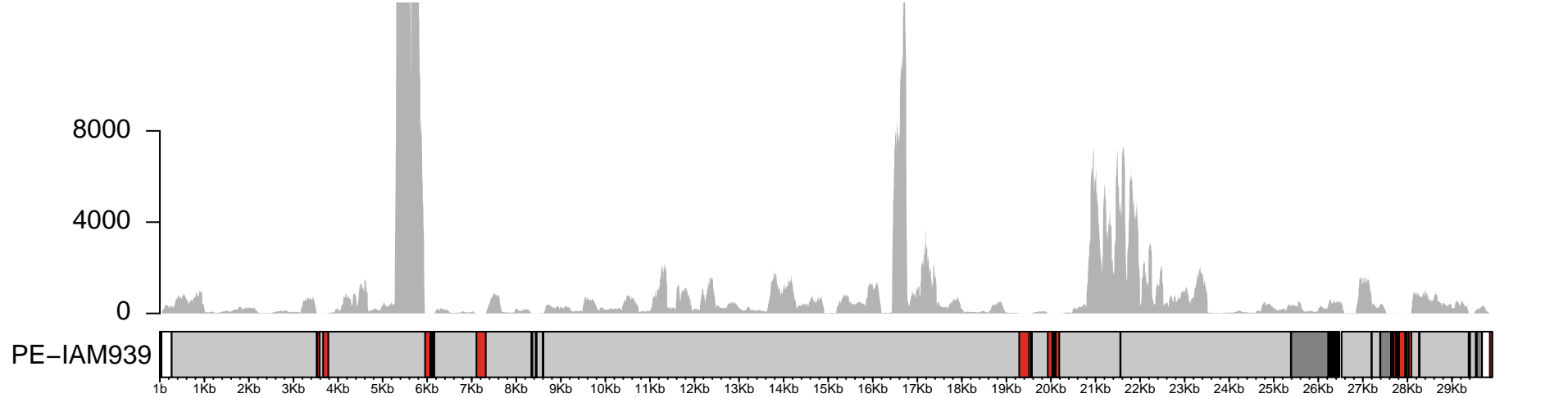

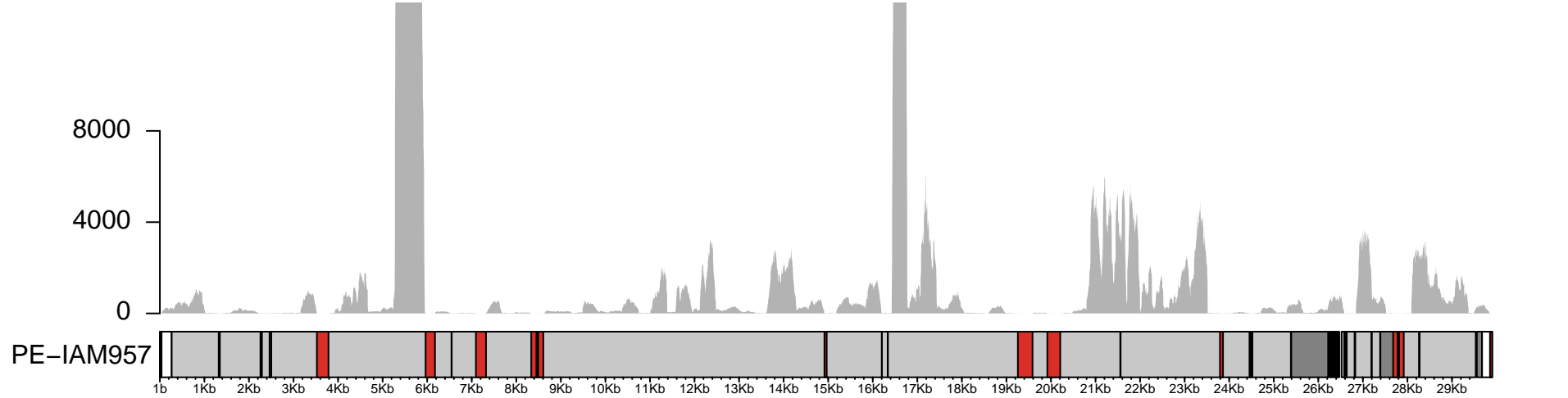

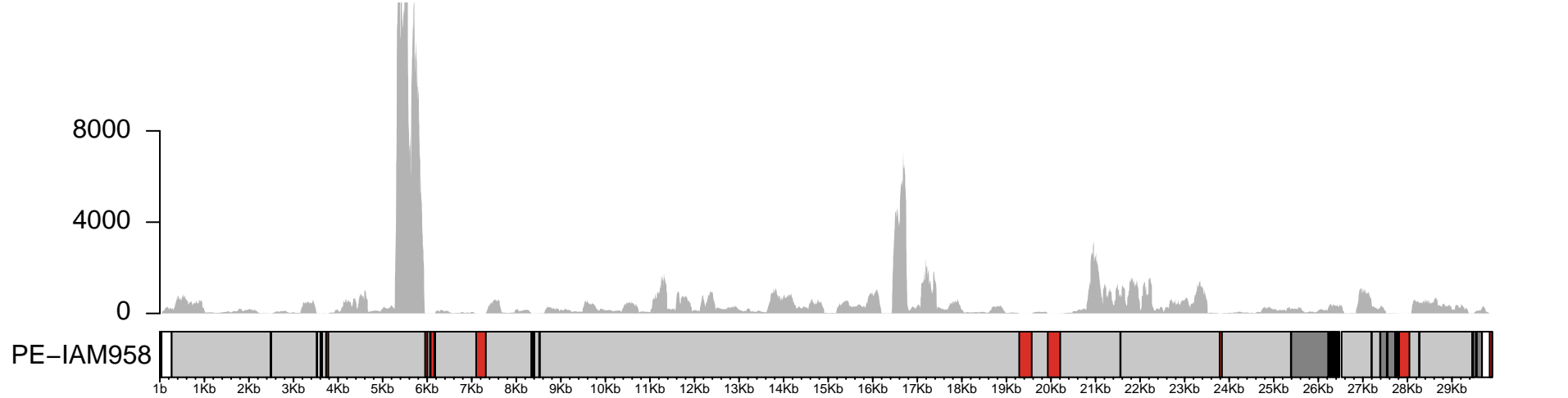

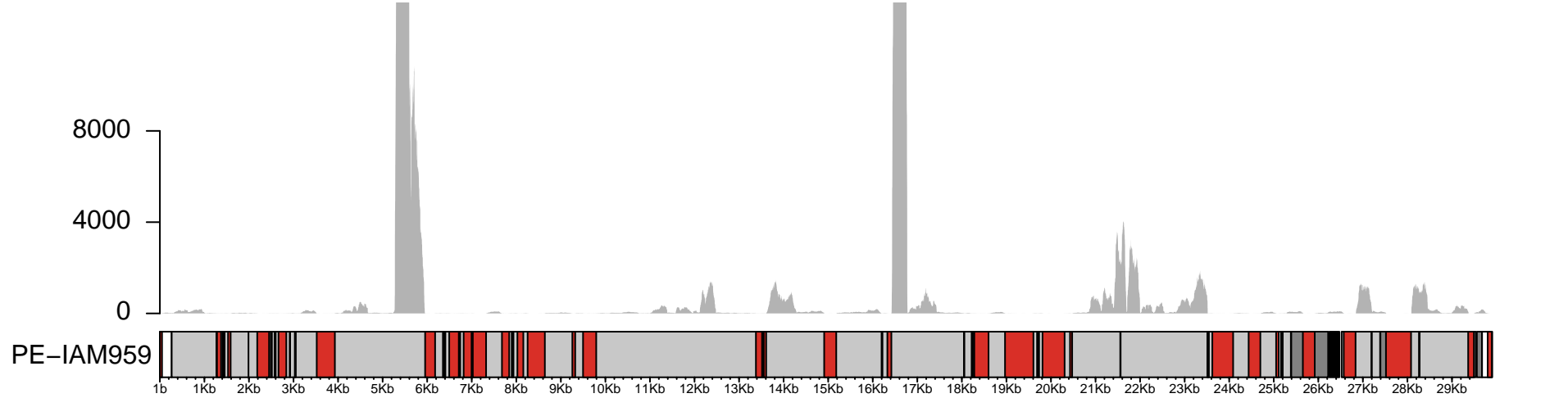

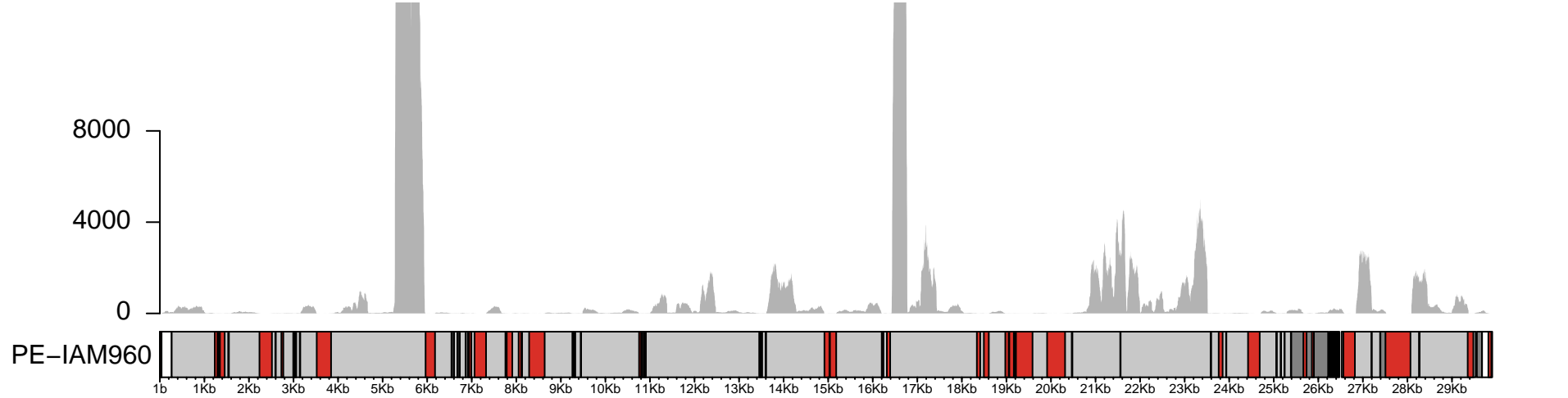

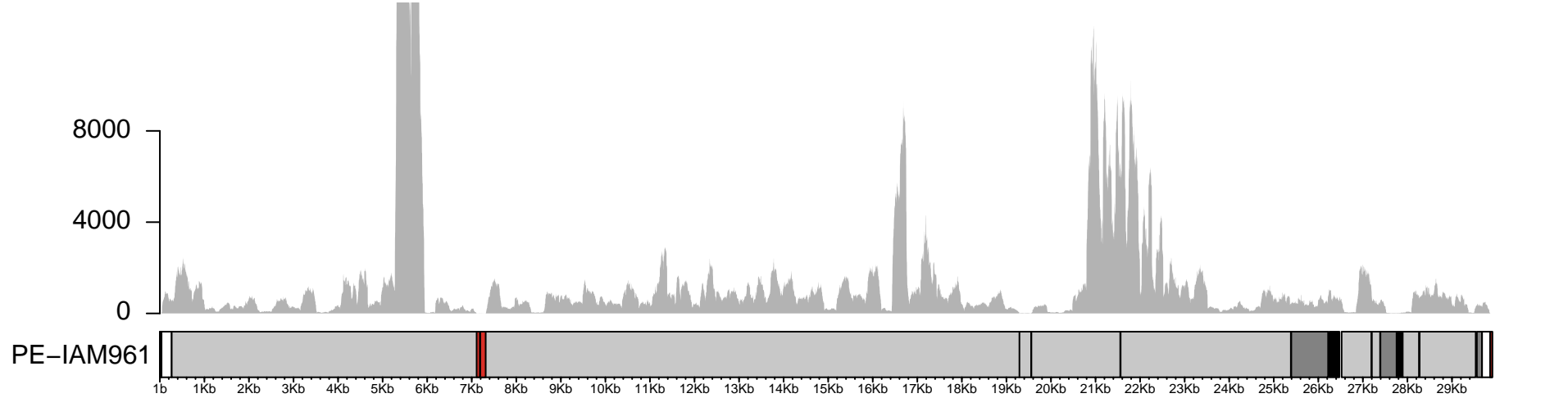

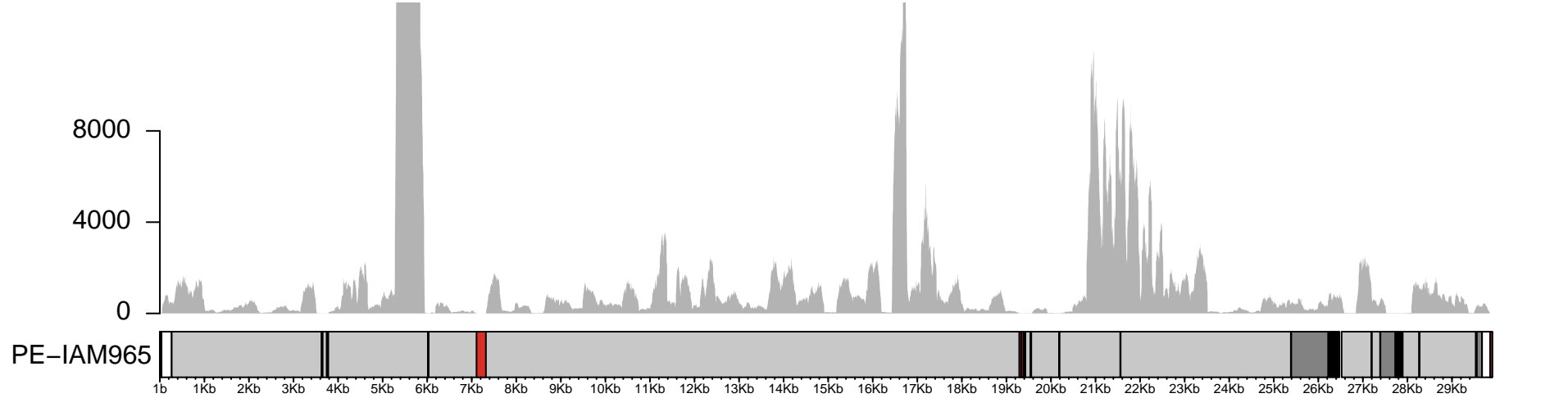

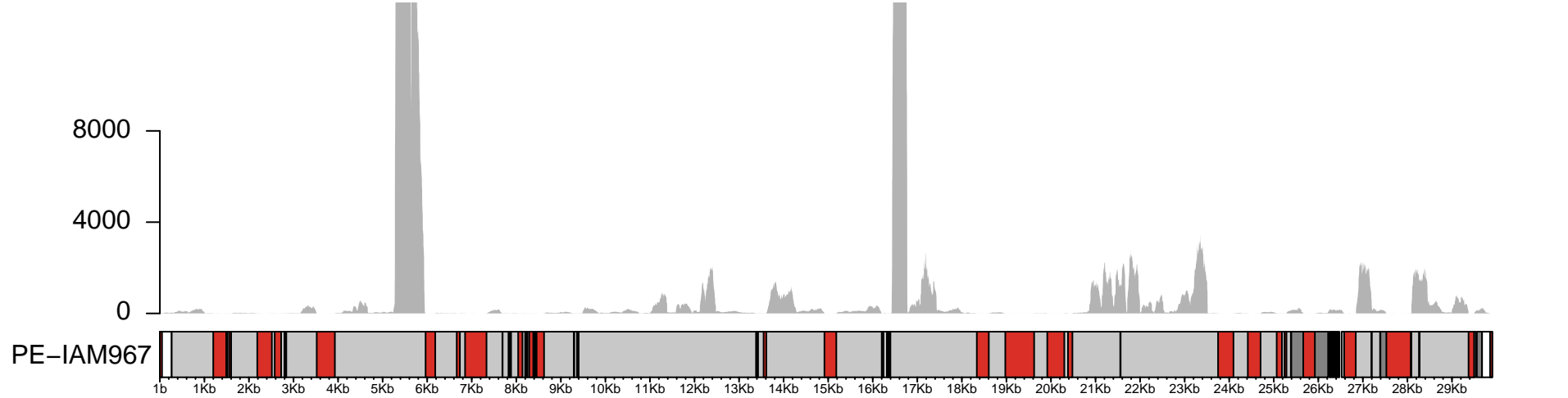

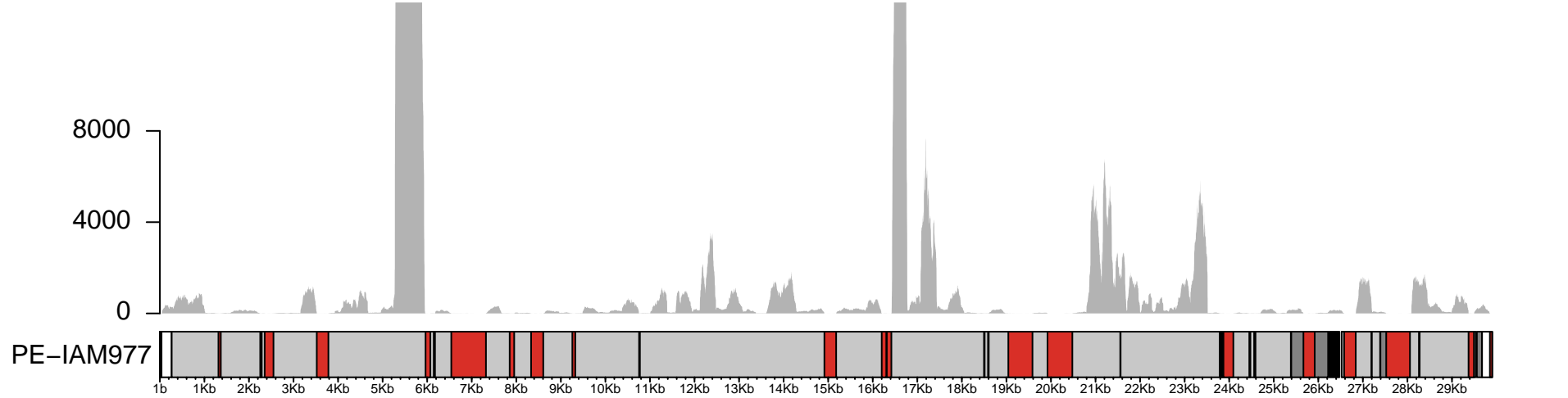

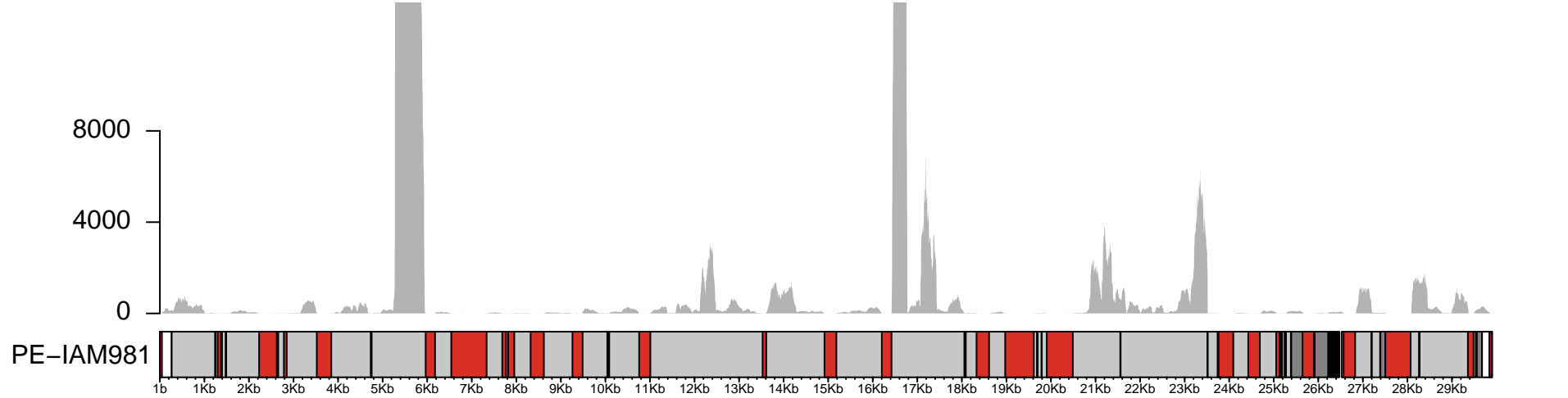

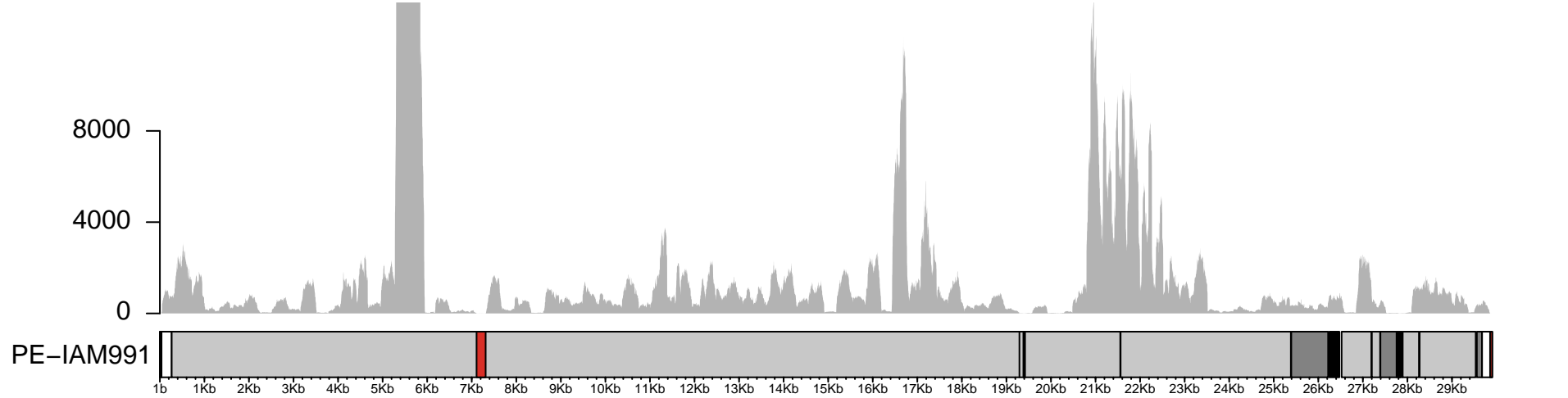

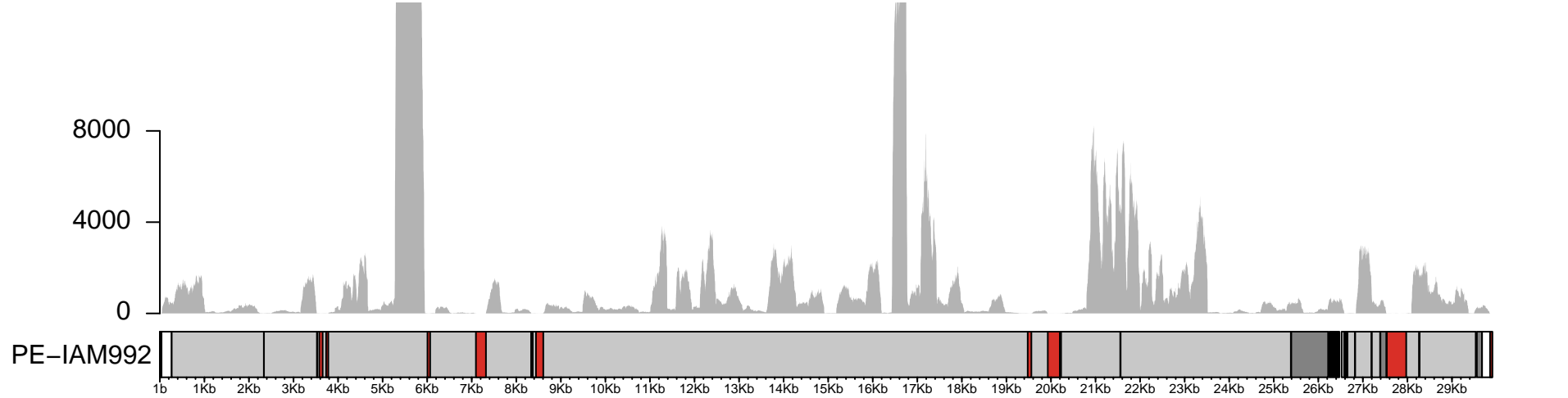

Supplement: Supplementary file 1 [file viruses-12-01414-s001.zip › Supplementary_Material/Supplementary_File_1.pdf]
